# Effect of genomic variations in severe fever with thrombocytopenia syndrome virus on the disease lethality

**DOI:** 10.1080/22221751.2022.2081617

**Published:** 2022-06-20

**Authors:** Zi-Niu Dai, Xue-Fang Peng, Jia-Chen Li, Jing Zhao, Yong-Xiang Wu, Xin Yang, Tong Yang, Shao-Fei Zhang, Ke Dai, Xiu-Gang Guan, Chun Yuan, Zhen-Dong Yang, Ning Cui, Qing-Bin Lu, Yong Huang, Hang Fan, Xiao-Ai Zhang, Geng-Fu Xiao, Ke Peng, Lei-Ke Zhang, Wei Liu, Hao Li

**Affiliations:** a State Key Laboratory of Pathogen and Biosecurity, Beijing Institute of Microbiology and Epidemiology, Beijing, People’s Republic of China; b College of Life Sciences, Fujian Agriculture and Forestry University, Fuzhou, People’s Republic of China; c The People's Liberation Army 990 Hospital, Xinyang, People’s Republic of China; d School of Public Health, Peking University, Beijing, People’s Republic of China; e State Key Laboratory of Virology, Wuhan Institute of Virology, Chinese Academy of Sciences, Wuhan, People’s Republic of China

**Keywords:** Emerging infectious diseases, tick-borne infectious diseases, viral infections, severe fever with thrombocytopenia syndrome, genetic diversity, case fatality rate, inflammatory response

## Abstract

Severe fever with thrombocytopenia syndrome virus (SFTSV), an emerging tick-borne bunyavirus, causes mild-to-moderate infection to critical illness or even death in human patients. The effect of virus variations on virulence and related clinical significance is unclear. We prospectively recruited SFTSV-infected patients in a hotspot region of SFTS endemic in China from 2011 to 2020, sequenced whole genome of SFTSV, and assessed the association of virus genomic variants with clinical data, viremia, and inflammatory response. We identified seven viral clades (I-VII) based on phylogenetic characterization of 805 SFTSV genome sequences. A significantly increased case fatality rate (32.9%) was revealed in one unique clade (IV) that possesses a specific co-mutation pattern, compared to other three common clades (I, 16.7%; II, 13.8%; and III, 11.8%). The phenotype-genotype association (hazard ratios ranged 1.327-2.916) was confirmed by multivariate regression adjusting age, sex, and hospitalization delay. We revealed a pronounced inflammation response featured by more production of CXCL9, IL-10, IL-6, IP-10, M-CSF, and IL-1β, in clade IV, which was also related to severe complications. We observed enhanced cytokine expression from clade IV inoculated PBMCs and infected mice. Moreover, the neutralization activity of convalescent serum from patients infected with one specified clade was remarkably reduced to other viral clades. Together, our findings revealed a significant association between one specific viral clade and SFTS fatality, highlighting the need for molecular surveillance for highly lethal strains in endemic regions and unravelled the importance of evaluating cross-clade effect in development of vaccines and therapeutics.

## Introduction

Severe fever with thrombocytopenia syndrome virus (SFTS) is an emerging tick-borne virus haemorrhagic fever that was first reported in 2009 [1], endemic to China [2,3], South Korea [4], Japan [5], Vietnam [6], and Myanmar [7]. Multiple transmission routes, including tick bite [8], human-to-human via direct contact with patient’s blood and/or human body fluid [9,10], and potential sex transmission [11], in addition to the high mortality and lack of vaccine and effective therapeutic treatment, have raised a global health concern about the potential for a pandemic of SFTS.

The causative agent of SFTS, SFTSV virus (SFTSV), belongs to the genus *Bandavirus* in the family *Phenuiviridae* of the order *Bunyavirales*. Similar to other bunyaviruses, SFTSV has a three single-stranded RNA genomes: L-segment encoding for the RNA-directed RNA polymerase (RdRp), M-segment encoding for the envelope glycoproteins (GPs) Gn and Gc that play important roles in receptor binding and entrance into cells, and S-segment encoding for both a nucleocapsid protein (Np) and a nonstructural protein (NS) that interacts with interferon signalling pathways and plays an important role in evading innate immunity [1,12]. Like other tri-segmented RNA viruses, the evolutionary characteristics of SFTSV rendered high substitution rates and recombinant and reassorted strains, leading to widespread geographical co-circulation of viral strains [13,14]. Previous studies grouped SFTSV strains into the Chinese lineage including at least four genotypes and the Japanese lineage including three genotypes [13,15], suggesting a degree of sequence diversity that shows a geographic pattern.

In clinical practice, a wide spectrum of SFTSV infections was observed that ranged from asymptomatic infection to severe illness, with multiple organ failures or death. As shown in previous clinical studies, the case fatality rate (CFR) of SFTS differed among Japan, South Korea, and China [3–6], and within China ranged from 5.3% to 16.2% across provinces [16]. Whether there are high virulent SFTSV strains that were responsible for this divergence were rarely investigated in previous clinical studies, thus the potential genotype-to-phenotype association and its application for the public health and clinical measures or available diagnostics, and therapeutics remained to be clarified. Here, we used whole-genome sequences of SFTSV from human patients to investigate the role of SFTSV genetic diversity in determining disease severity and eliciting antibody-induced immunity, and to assess the phylogeny, evolution and spatial transmission of the virus.

## Materials and methods

### Study site and participants

This prospective observational study was performed in Xinyang City, Henan Province, China, the hotspot of SFTS endemic located at the centre of Dabie mountain [3,17]. From 2011 to 2020, we have consecutively collected serum samples from patients with SFTS at the People’s Liberation Army (PLA) 990 Hospital, the local designated hospital for treating SFTS. All laboratory-confirmed SFTSV infection had been diagnosed by a standard criterion released by the Chinese Ministry of Health, with detailed information seen in the Supplementary Methods. A group of trained research staff performed questionnaire and medical record reviews to collect data about demography, medical history, clinical signs and symptoms, laboratory test results, treatment regimens, and outcomes. The patients who had been discharged from the hospital after discontinuation of therapy due to adverse clinical progression were followed up by phone calls or home visits within two weeks to determine their final outcome (fatal or survival). The research protocol was approved by the human ethics committee of the institute in accordance with the medical research regulations of China (AF/SC-08/02.114). All participants provided written informed consent to have their samples and information collected.

### Full-length viral genome sequencing on clinical specimens

Viral RNA was isolated from a serum sample using a QIAamp viral RNA Mini Kit (Qiagen) and subjected to quantitative reverse-transcription polymerase-chain-reaction (qRT-PCR) targeting the S segment of SFTSV [17]. Nested RT–PCR assays were performed on viral RNA with CT values of qRT-PCR ≤ 32, to obtain a full-length genome sequence of SFTSV. In brief, a total of 19 pairs of primers covered whole genomic sequences of SFTSV were used for the first round of amplification with the PrimeScript One-Step RT–PCR Kit (Takara), and 19 pairs of primers were used for the second round of amplification with the DreamTaq Green PCR Master Mix (Thermo Scientific). Detailed information on primer sequences was shown in Table S1. PCR amplicons were purified and sequenced on a 3730 DNA Sequencer (Applied Biosystems).

### Sequence analysis

Sequences were assembled using SeqMan Pro v7.1.0 in the Lasergene (DNASTAR) and aligned using CLC Workbench v9.0 (Bio-Qiagen), with SFTSV strain HN6 used as the reference sequence (GenBank accession numbers: HQ141595, HQ141596, and HQ141597 for L, M, and S segments). Phylogenetic trees were constructed based on S, M, and L segments of SFTSV using the Maximum-likelihood model in the software MEGA X. Sequences from the Heartland virus were used as outgroups. The confidence of the phylogenetic tree was tested using 1000 bootstrap replications. In case of inconsistent genotypes determined from three segments for the same strain, a potential genetic reassortment event was considered and tested. Seven different recombination methods (RDP, GENECONV, Bootscan, Maxchi, Chimaera, SiSscan, and 3Seq) were performed by using the software RDP v4.101, based on which reassortment events were considered only when four or more methods yielded *p*-values <0.05. The co-mutation pattern was constructed by analysing pairwise co-occurrence of amino acids within a single SFTSV protein or between two different SFTSV proteins as previously described [18]. Complete genome sequences of SFTSV identified in this study were deposited to GenBank with accession numbers listed in Table S2. In addition, a total of 424 SFTSV S-segment sequences from human patients reported in China were available from GenBank and used to investigate viral diversity at a national level (Table S3).

### Immune mediator detection

Levels of 48 specific immune mediators including inflammatory cytokines, chemokines, and growth factors were quantified in serial serum samples from patients using the Bio-Plex Pro Human Cytokine Screening Panel (Bio-Rad) with Luminex xMAP technology (detailed in Appendix Methods).

### SFTSV-specific antibody detection

Titres of IgM and IgG to SFTSV NP were examined in serial serum samples using commercial ELISA kits (Nanjing Immune-Detect Biotech). Serum specimens were serially diluted starting with 1:10 and 1:100 for detection of IgM and IgG antibodies, respectively, and analysed in duplicate. Neutralization titres of serum samples from convalescent SFTS patients collected at three months post disease onset were determined against four SFTSV genotypes (clades I–IV) using an enzyme-linked immunosorbent assay (ELISA)-based conventional microneutralization test. Serum samples were serially diluted two-fold starting from 1:20 to 1:320 and analysed in duplicate (detailed in Appendix Methods).

### SFTSV culture in Vero cells and infection in PBMCs

SFTSV strains HBMC16_human_2015 and WCH, representing clades III and I, were obtained from China Centre for General Virus Culture Collection. SFTSV strains HNXY2017-66 and HNXY2017-50, representing clades II and IV, were isolated from the serum of patients in the PLA 990 Hospital. The growth kinetics of SFTSV was examined by seeding in Vero cells with four viral clades at a multiplicity of infection (MOI) of 1, and intracellular virus RNA levels and supernatant virus titres were tested at 12, 24, 36, 48, and 72 h post infection with qRT-PCR and immunological focus assay [19], respectively (detailed in Appendix Methods). Peripheral blood mononuclear cells (PBMCs) isolated from healthy donors were inoculated by the same four virus strains with MOI of 1. After 24 h post infection, the intracellular expression and supernatant concentrations of IL-1β, IL-6, and IL-10 were tested using qRT-PCR and ELISA, respectively [20].

### Animal study

Five-week-old female C57BL/6 mice were treated with anti-interferon alpha/beta receptor 1 (IFNAR1) IgG antibody (BioXCell) by intraperitoneal injection (each 1.7 mg) one day prior to infection. Mice were intraperitoneally inoculated with 10^3^ FFU of SFTSV in 100 μL of DMEM at day 0 (*n* = 10 for each virus strain), or the same volume of DMEM (vehicle group, *n* = 5), and then monitored for 10 days. For the examination of virus titre and IL-1β in the spleen, mice were infected as above described (*n* = 4 for each virus strain). Spleen samples were collected after sacrifice. Virus titres in the spleen were determined using immunological focus assay. Concentrations of IL-1β in the spleen were determined by ELISA and immunohistochemistry staining as previously described [20].

### Statistical analysis

Continuous variables were summarized as means and standard deviations (SD) or as medians and interquartile range (IQR). Categorical variables were summarized as frequencies and proportions. Independent *t* test, chi-square test, Fisher’s exact test, ANOVA test, or nonparametric test were used as appropriate, to determine the difference between groups.

For effect analysis of virus variations on disease severity, the Kaplan–Meier method was used to analyse time-to-event data using the log-rank test; hazard ratio (HR), and 95% confidence interval (CI) were calculated using Cox regression models. Generalized estimating equation (GEE) model was used to analyse serially measured viral loads and antibody titres. Associations of virus nucleotide or amino acid mutations with fatal outcomes were assessed using univariate and multivariate (by adjusting age, sex, and delay from symptom onset to hospital admission) logistic regression models. Odds ratio (OR) and the 95% CI were estimated using maximum-likelihood methods. A two-sided *p* value of <0.05 was considered statistically significant. All statistical analyses were performed using SPSS software, version 19.0 (Statistical Product and Service Solutions, Chicago, IL, USA).

## Results

### Genomic diversity of SFTSV from patients with SFTS in a hotspot region

A total of 1149 patients who were hospitalized from 1 April 2011 to 31 October 2020 that yielded CT values of ≤32 for SFTSV were subjected to whole-genome sequencing. The nearly full-length genome sequences of SFTSV were obtained from 805 patients, who had similar mean (SD) age (63.0 ± 11.8 vs. 63.0 ± 11.7 years), female proportion (60.6% vs. 60.9%), and median (IQR) of hospitalization delay (5 [4–7] vs. 5 [4–7]) with the total tested 1149 patients (Table S4). About 12.2% (98/805) had reported a history of the tick bite, and none had potential human-to-human transmission. Clinical manifestations recorded before hospitalization, underlying diseases, laboratory parameters tested on admission, and the 30-day case fatality rate (CFR) were highly comparable between the 805 patients and the total tested patients (Tables S4 and S5). These data suggest that the 805 patients included in the analysis are well representative of the whole tested population of patients with SFTS.

**Figure 1.**
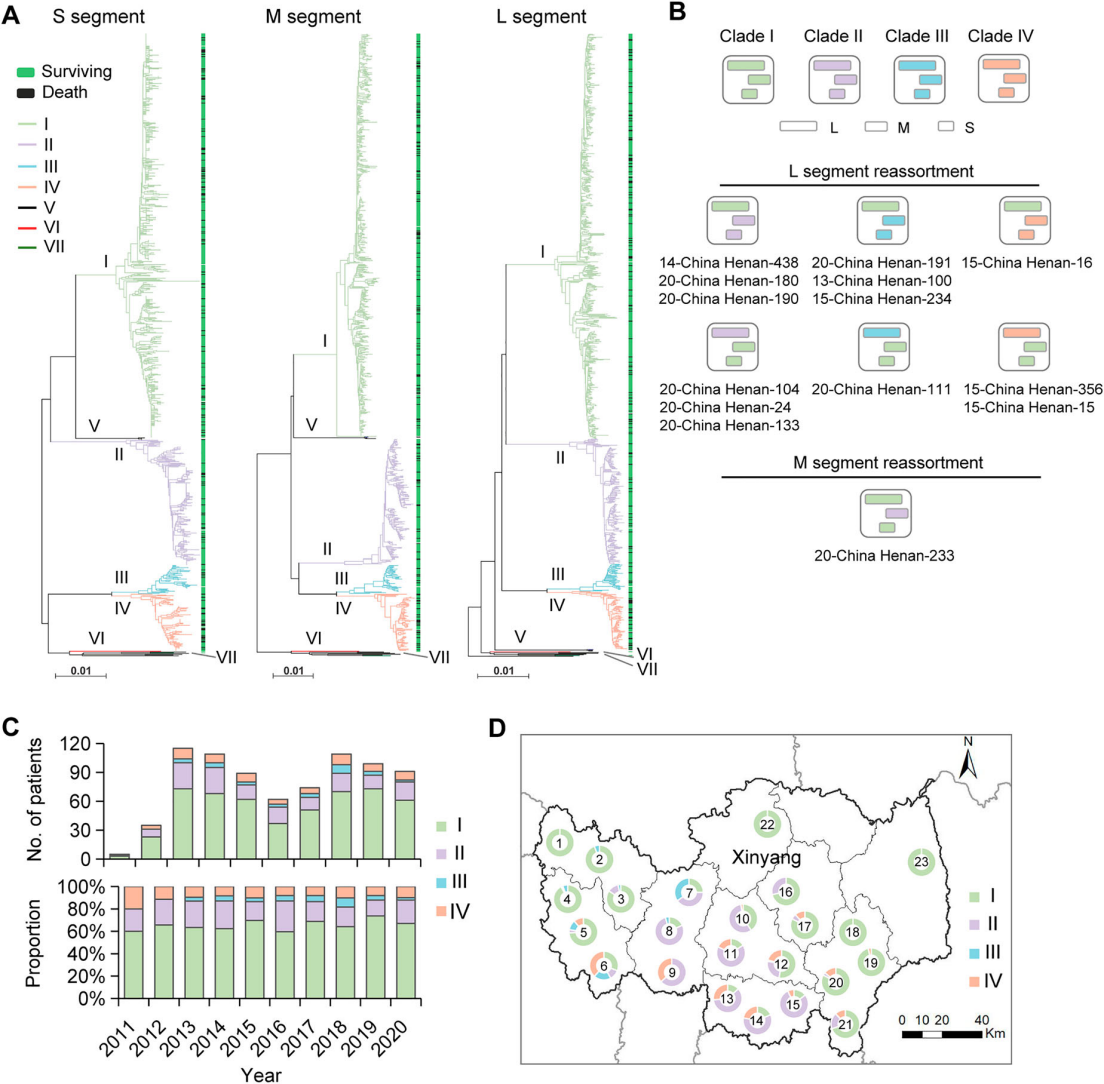
Phylogenetic characterization of SFTSV S, M, and L segments sequenced from human patients. Maximum-likelihood trees for SFTSV S, M, and L genome segments (A). Seven viral clades indicated by coloured branch were identified from patients with SFTS in this study. The black branch indicates sequences downloaded from the GenBank. The outcome (surviving or death) of the patient was shown on the right side of the phylogenetic trees. Scale bars indicate a number of substitutions per site. Graphic representation of reassortment events in SFTSV genome sequences obtained from patients (B). The number beneath the reassortment graphics indicates the name of the virus isolates with detailed information in Appendix Table 2. The number and proportion of four common viral clades obtained from patients with SFTS in each studied year (C). Geographic distribution of four common viral clades from patients with SFTS (*n* = 712) residing in Xinyang City (D). The number in the middle of the circle indicates the region number where the patients reside, with detailed data shown in Table S7.

Segment-based phylogenetic analysis with the use of S, M, and L nucleotide sequences revealed seven viral clades, including five in Chinese geographic clades (I, II, III, IV, and V) and two in Japanese geographic clades (VI and VII) (Figure 1(A)). A total of 14 (1.7%) SFTSV strains showed shifted evolutionary positions among the phylogenetic trees, indicating potential reassortment events, with 13 events involving the L segment and one event involving the M segment (Figure 1(B)). Among them, 11 strains possessing reassortment events were further confirmed by using RDP packages (Table S6). Only one of the 14 patients died, resulting in a CFR of 7.1% for infection with reassorted SFTSV strains.

Among the non-reassorted strains, clade I was the most common genotype, taking a proportion of 65.9% (521/791), followed by clades II (160/791, 20.2%), IV (73/791, 9.2%), and III (34/791, 4.3%; Figure 1(C)). Other rare clades included V, VI (a novel clade that most closely related to Japanese geographic clades), and VII, with each of them observed in only one patient and were not included in the association analysis. An obvious region-related pattern was observed, with clade I more frequently identified from patients residing in western and eastern regions, clade II more frequently identified from central regions, and clade IV from southern regions of Xinyang City (Figure 1(D) and Table S7). All four SFTSV clades were identified during the study period, with similar proportions over a 10-year time (Figure 1(C)). The proportion did not differ regarding age or sex, also was comparable among months when the yearly data were combined (Figure S1). Together, these data indicate that multiple genomic variations of SFTSV continuously circulate and cause human infections in the hotspot endemic region.

### Association of SFTSV genomic variants with disease severity in patients with SFTS

**Figure 2.**
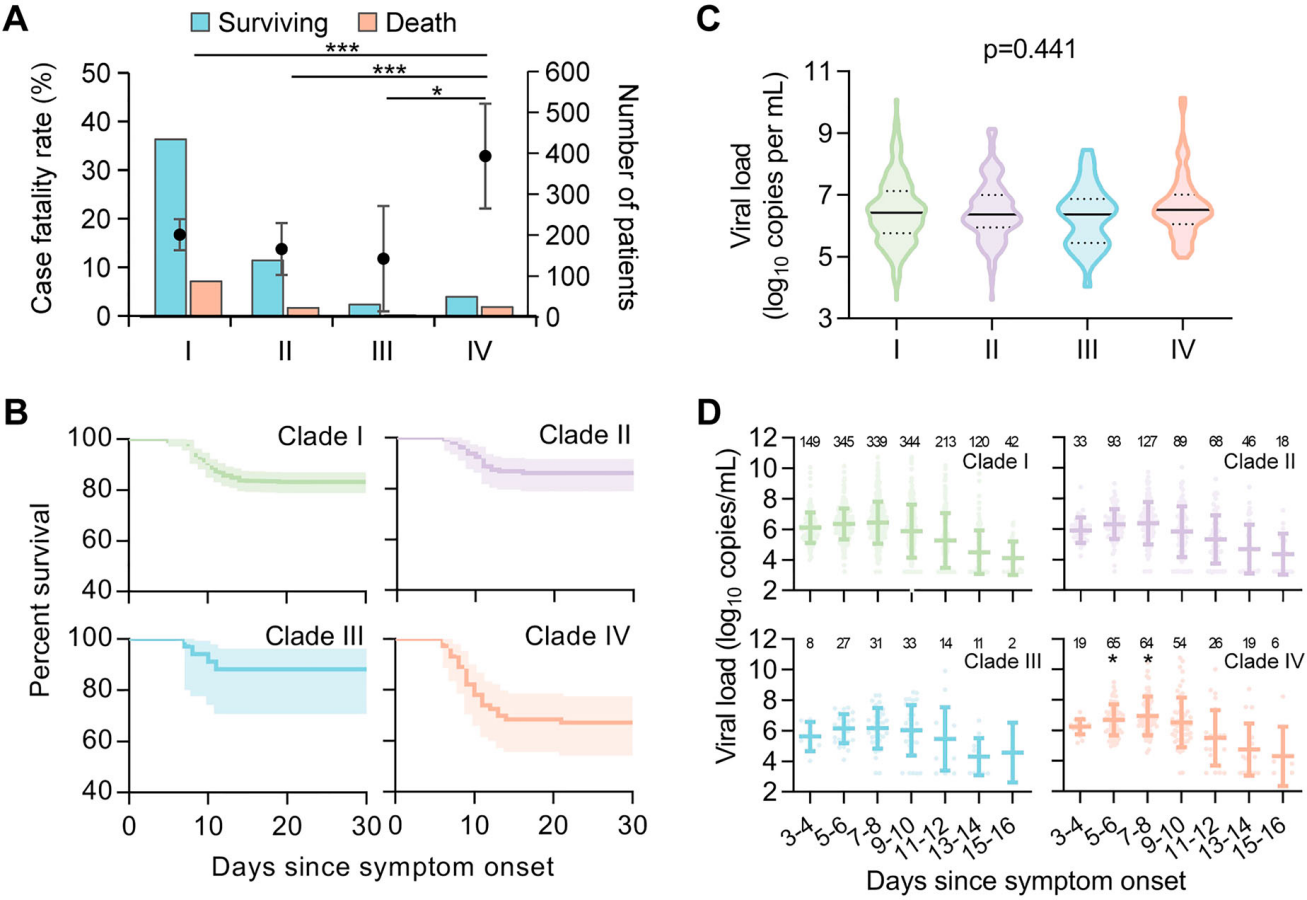
CFRs and viral loads of patients infected with four viral clades. Numbers and CFRs of patients (detailed in Table S8) infected with four viral clades were shown (A). Datapoints show CFRs; error bars show 95% CIs. Chi-square test or Fisher’s exact test was used to determine the difference of case fatality rate between groups. Survival curves of patients infected with four viral clades were shown (B). The Kaplan–Meier method was used to analyse time-to-event data, and statistical analysis results were shown in Table S9. Viral loads in patients infected with four SFTSV clades were shown (C). Horizontal and dotted lines indicate the mean value and IQR. ANOVA test was used as appropriate, to determine the difference between groups. Kinetics of viral loads in patients infected with four viral clades was shown (D). The number of patients who had serum viral loads tested was shown above each dot collection. The star indicates the statistical significance (*p* < 0.05) of the comparison of serum viral loads among the patients infected with four SFTSV clades determined by the ANOVA test. The dot indicates the exact value of the viral load, the horizontal line indicated mean value, and the bar indicates SD. CFR, case fatality rate; ns, no significance.

At follow-up, 139 of the 805 patients with SFTS had died from the current episode of SFTSV infection, giving an average 30-day CFR of 17.3% (Table S1). All four common SFTSV clades (I–IV) were determined in fatal patients, with the highest CFR observed in the patients infected with clade IV (32.9%, 95% CI 22.1–43.7%), followed by clade I (16.7%), clade II (13.8%), and clade III (11.8%) (Figure 2(A) and Table S8). The Kaplan–Meier survival analysis showed significantly increased CFRs related to clade IV, with the HRs (95% CIs) ranging from 1.292 (1.111–1.502) compared with clade I to 3.091 (1.072–8.911) compared with clade III (Figure 2(B) and Table S9). When further considering the influence of confounding factors, the association with high CFR remained for clade IV by performing Cox regression analysis after adjusting for age, sex, and hospitalization delay (Table S9).

Severe complications that were thought to be related to the fatal outcome of SFTS [3] were assessed, among which dyspnoea (*p* = 0.007), disseminated intravascular coagulation (DIC; *p* = 0.046), and neurological symptoms (*p* < 0.001) were all observed with the highest frequencies in patients infected with clade IV (Table S10). These findings collectively revealed a significant association between one specific viral variant (clade IV) and the fatality and severity of SFTS.

### Levels of viremia, SFTSV-specific antibodies, and inflammatory response in patients infected with different SFTSV genomic variants

The SFTSV viral loads measured on hospital admission were comparable (*p* = 0.441; Figure 2(C)) and showed a similar dynamic trend (*p* > 0.05; Figure 2(D)) among four viral clades, however, with viremia measured at 5–6 and 7–8 days post disease onset significantly elevated in patients infected with clade IV (both *p* < 0.05; Table S11). The longitudinal pattern of SFTSV-specific IgM and IgG antibodies was measured in serum samples from 77 patients (aged 32–74 years, 61.0% female), displaying similar kinetics among patients infected with the four viral clades (both *p* > 0.05; Figure S2). The neutralization assay revealed the induction of cross-reactive neutralizing antibodies among patients infected with four SFTSV clades; however, the convalescent serum samples from patients infected with one specified viral clade showed significantly decreased neutralizing activity against other viral clades (Figure S3). For instance, clades I–III derived neutralizing antibodies showed 1.51 to 3.24-fold reduction in neutralizing activity against clade IV.

**Figure 3.**
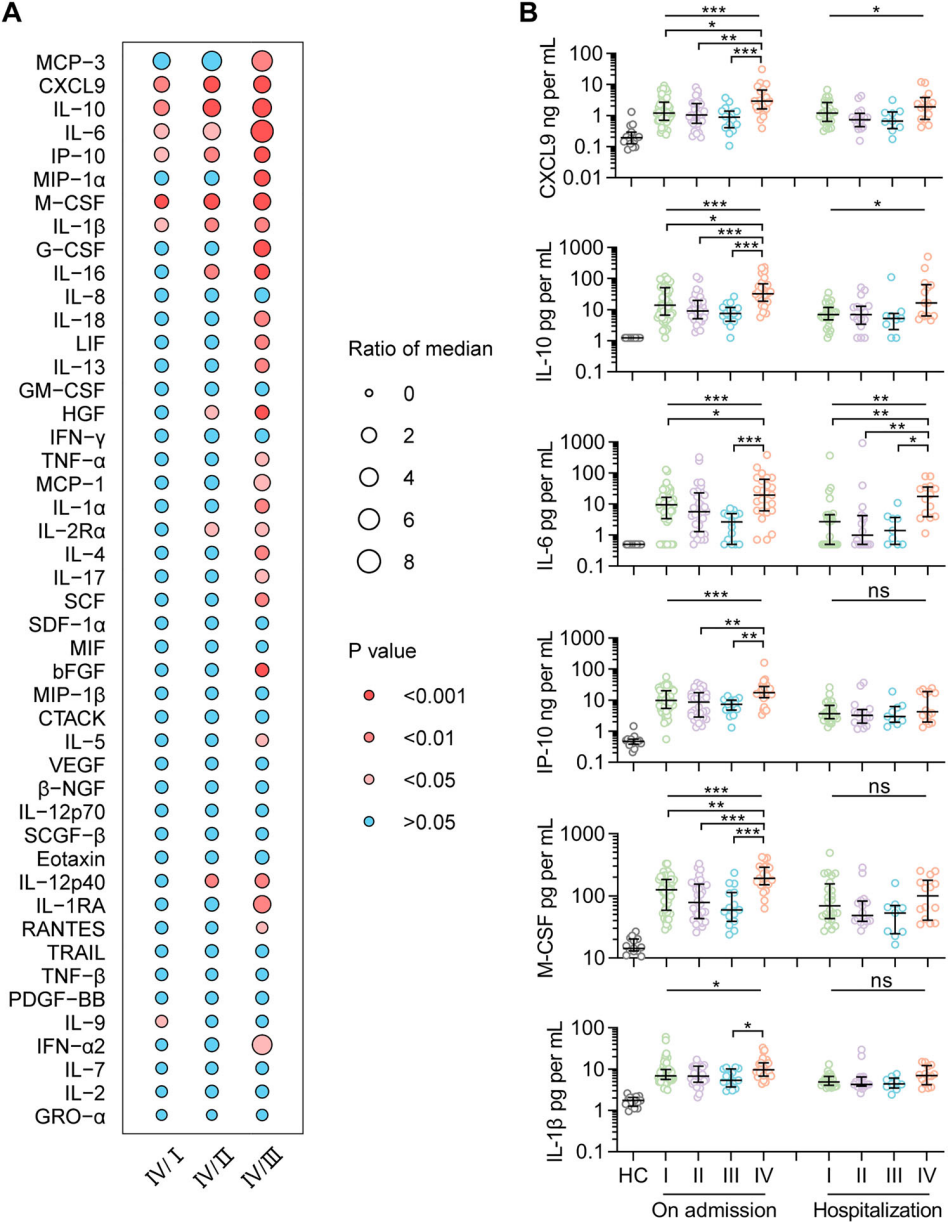
Levels of immune mediators in serum from patients infected with four viral clades. Serum levels of 45 immune mediators were compared between clade IV (*n* = 25) and each of the other three clades (I, *n* = 54; II, *n* = 32; III, and *n* = 15) (A). Comparison of serum concentrations of CXCL9, IL-10, IL-6, IP-10, M-CSF, and IL-1β, among patients infected with four viral clades (B), with detailed information in Table S16. Datapoints show exact values; horizontal lines show median values; error bars show interquartile range. Independent *t*-test and ANOVA test were used to determine the difference between two and multiple groups, respectively. *p < 0.05, **p < 0.01, and ***p < 0.001. HC, healthy control; ns, no significance.

A profile of 48 immune mediators was evaluated in 126 patients (aged 30–76 years, 55.6% female), with six of them, including chemokines CXCL9 and IP-10, pro-inflammatory interleukin (IL)-6 and IL-1β, anti-inflammatory IL-10, and growth factor macrophage colony-stimulating factor (M-CSF), significantly overexpressed in 25 patients infected with clade IV than either of the other three clades (Figure 3(A)). This pattern on hospital admission was confirmed by Kruskal–Wallis test, indicative of the most severe inflammatory responses produced by clade IV, and with three of them, including CXCL9, IL-10, and IL-6, maintaining higher in patients infected with clade IV during their hospitalization (all *p* < 0.05, Figure 3(B)). These results suggest a correlation between SFTSV genomic variants and virus-induced hyperinflammation in patients, which is related to lethal infection.

### Enhanced inflammatory response in SFTSV clade IV infected PBMCs and mouse model

To further confirm the correlation between SFTSV genomic variants and pathogenicity, we first assessed the kinetics of viral replication in Vero cells. SFTSV strains representing the four viral clades did not show the apparent difference in replication rate in cell culture as measured up to 72 h after inoculation (Figure S4A). The *ex vivo* infection of SFTSV in PBMCs from healthy donors demonstrated significantly high expression levels of IL-6, IL-1β, and IL-10 induced from clade IV in either PBMCs or supernatants that were tested at 24 h after inoculation (Figure S4B). Finally, the inflammatory response induced by different SFTSV clades was analysed in a lethal C57BL/6 mouse model with pre-treatment with an anti-IFNAR1 IgG antibody. The high fatality was seen in the mice infected with clade IV (50%) compared with clades I (20%), II (30%), and III (30%), although without statistical significance (Figure S5A). At four days post infection, spleen samples were collected for viral titring by immunological focus assay and IL-1β measurement using ELISA and immunohistochemistry staining. There was no significant difference from virus titres (Figure S5B); however, enhanced production of IL-1β was observed in the spleen of the mice infected with clade IV (Figure S5C, D). These results supported that SFTSV clade IV may result in stronger systemic inflammation in human patients, thus leading to an elevated CFR.

### Characterization of SFTSV genomic variants and the correlation with SFTS fatality

The substitution rate was higher in the S segment (9.3%) than that in M (8.0%) or L (7.4%) segment (*p* = 0.030), and transition substitution was more commonly observed, with a proportion ranging from 79.3% to 82.7% (Table S12). SFTSV clade I showed a wider nucleotide sequence identify the range, while clade III showed a higher amino acid divergence (Figure S6). A total of 247 nucleotide mutations, including 40, 74, and 133 from S, M, and L segments, respectively, were determined to be associated with SFTS fatality by multivariate regression (Table S13). A large number of amino acid mutations were identified, mostly in viral clades II, III, and IV (Figure S7). A total of 25 missense mutations (five in the NS, nine in the Gn, four in the Gc, and seven in the RdRp) were associated with SFTS fatality using multivariate regression analysis after adjusting for sex and hospitalization delay (Table 1). The top five mutations with high ORs included two in the NS (S207P and Q245H), one in the Gn (K371R), one in the Gc (V587I), and one in the RdRp (R1684K).

**Table 1.** Amino acid mutations related to fatal outcomes in patients with SFTS.

Amino acid mutation	Fatal/Total (%)		Univariate regression		Multivariate regression*	
	Wild type	Mutation type	OR (95% CI)	*P*	OR (95% CI)	*p*
NS						
S207P	114/718 (15.9)	24/73 (32.9)	2.595 (1.531–4.399)	<0.001	2.987 (1.699–5.251)	<0.001
I223V	110/681 (16.2)	28/107 (26.2)	1.840 (1.142–2.964)	0.012	2.072 (1.246–3.445)	0.005
D239E	127/758 (16.8)	11/32 (34.4)	2.603 (1.224–5.532)	0.013	2.626 (1.182–5.836)	0.018
Q245H	114/718 (15.9)	24/73 (32.9)	2.595 (1.531–4.399)	<0.001	2.987 (1.699–5.251)	<0.001
Y249H	110/681 (16.2)	28/108 (25.9)	1.817 (1.128–2.925)	0.014	2.061 (1.240–3.424)	0.005
Gn						
D37G	110/685 (16.1)	28/104 (26.9)	1.926 (1.193–3.109)	0.007	2.139 (1.284–3.562)	0.004
G114E	114/716 (15.9)	24/75 (32.0)	2.485 (1.470–4.200)	<0.001	2.876 (1.641–5.038)	<0.001
T273A	110/681 (16.2)	28/109 (25.7)	1.794 (1.115–2.887)	0.016	1.993 (1.201–3.305)	0.008
Q341P	109/681 (16.0)	29/110 (26.4)	1.879 (1.173–3.009)	0.009	2.077 (1.257–3.431)	0.004
K371R	114/717 (15.9)	24/74 (32.4)	2.539 (1.500–4.297)	<0.001	2.963 (1.687–5.205)	<0.001
Q394H	110/684 (16.1)	28/107 (26.2)	1.849 (1.148–2.980)	0.011	2.084 (1.253–3.464)	0.005
I491M	83/508 (16.3)	29/116 (25.0)	1.707 (1.054–2.763)	0.030	1.840 (1.103–3.069)	0.019
M506V	111/685 (16.2)	27/106 (25.5)	1.767 (1.092–2.862)	0.021	1.983 (1.188–3.312)	0.009
R525G	111/681 (16.3)	27/109 (24.8)	1.691 (1.046–2.733)	0.032	1.944 (1.165–3.242)	0.011
Gc						
V587I	114/718 (15.9)	24/73 (32.9)	2.595 (1.531–4.399)	<0.001	2.987 (1.699–5.251)	<0.001
T960I	90/553 (16.3)	24/73 (32.9)	2.520 (1.471–4.315)	<0.001	2.880 (1.620–5.121)	<0.001
S1056F	112/689 (16.3)	26/101 (25.7)	1.786 (1.094–2.915)	0.020	2.034 (1.207–3.428)	0.008
F1058L	115/715 (16.1)	23/76 (30.3)	2.264 (1.335–3.841)	0.002	2.599 (1.478–4.569)	<0.001
RdRp						
Y566F	110/683 (16.1)	28/108 (25.9)	1.823 (1.132–2.935)	0.013	2.068 (1.244–3.436)	0.005
I651V	111/688 (16.1)	27/103 (26.2)	1.847 (1.138-2.996)	0.013	2.123 (1.269–3.551)	0.004
R835K	114/715 (15.9)	24/76 (31.6)	2.433 (1.442–4.107)	<0.001	2.821 (1.613–4.933)	<0.001
S1038T	115/718 (16.0)	23/72 (31.9)	2.461 (1.443–4.198)	<0.001	2.785 (1.577–4.920)	<0.001
T1433A	114/716 (15.9)	24/75 (32.0)	2.485 (1.470–4.200)	<0.001	2.847 (1.626–4.987)	<0.001
I1447V	110/680 (16.2)	28/111 (25.2)	1.748 (1.088–2.810)	0.021	1.932 (1.166–3.201)	0.011
R1684K	114/717 (15.9)	24/73 (32.9)	2.591 (1.528–4.392)	<0.001	2.986 (1.699–5.248)	<0.001

*Adjusting for age, sex, and hospitalization delay. OR = odds ratio; CI = confidence interval. The amino acid mutations with a frequency less than 2% were not included in the association analysis.

**Figure 4.**
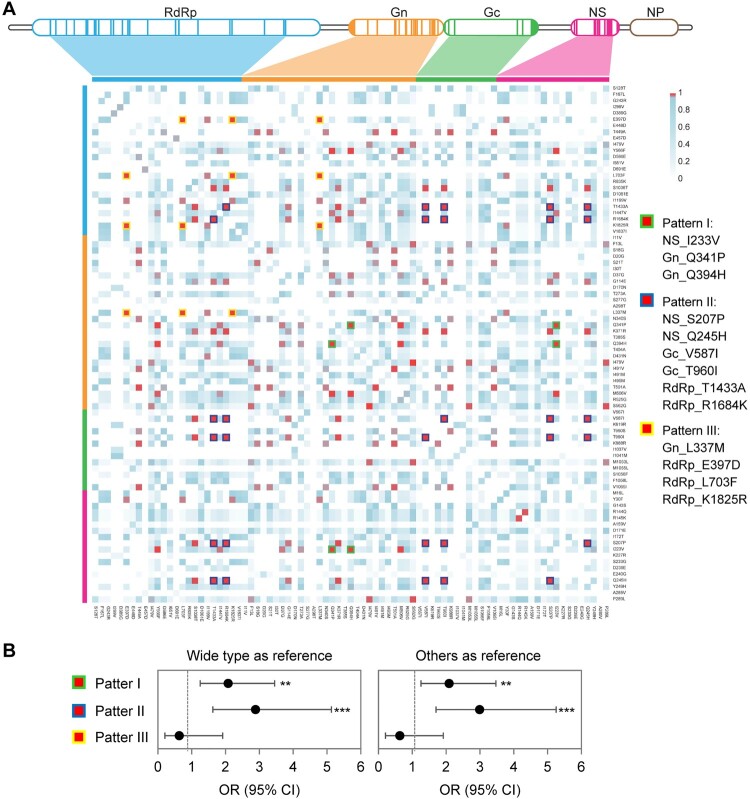
Co-mutation patterns in SFTSV strains from human patients. The matrix representation of co-mutation network for SFTSV strains obtained from patients with SFTS (A). Adjusted ORs for fatal outcomes in patients infected with SFTSV possessing co-mutation patterns (B). ORs were evaluated by using multivariate logistic regression models, with detailed information in Table S15. **p < 0.01, ***p < 0.001; OR = odds ratio.

Three co-mutation patterns were determined among all the 791 non-reassorted SFTSV strains (Figure 4(A)), with patterns I (NS_I233V, Gn_Q341P, and Gn_Q394H) and III (Gn_L337M, RdRp_E397D, RdRp_L703F, and RdRp_K1825R) related to clade III, with patterns I and II (NS_S207P, NS_Q245H, Gc_V587I, Gc_T960I, RdRp_T1433A, and RdRp_R1684K) related to clade IV (Figure S7; Table S14). Patterns I and II were associated with increased risks of fatal outcomes, with pattern II showing a higher OR when either compared to wide type (OR, 2.886; 95% CI, 1.624–5.130) or all other sequences (OR, 2.990; 95% CI, 1.702–5.254; Figure 4(B) and Table S15). Moreover, the strains of pattern II were also related to more severe inflammatory responses compared to wide-type viruses (Figure S8).

### Geographic distribution of SFTSV clade IV in China

To investigate the geographic distribution of SFTSV clade IV, a variant with potentially high pathogenicity, in other regions of China, we performed phylogenetic characterization of SFTSV S-segment sequences from human patients that were available from GenBank. A total of 424 S sequences from nine provinces of China were analysed, which revealed three additional clades, including two (VIII and IX) in the Chinese lineage and one (X) in the Japanese lineage (Figure S9A), besides the seven viral clades identified in Xinyang City. Likewise, clade I was the most common genotype (224/424, 52.8%), followed by clades IV (64/424, 15.1%) and II (54/424, 12.7%). Notably, clade IV was also determined in three other provinces (Hubei, Shandong, and Jiangxi) than Henan, with geographic scope only after clade I, which was distributed in six provinces (Figure S9B).

## Discussion

Empirical studies based on laboratory methods such as a combination of reverse genetics and cell culture and/or animal models had been applied to identify the mutations that affect virulence [21]. *In vitro* studies reported that the R624W and R962S in the GP of SFTSV may affect virus-cell fusion [22, 23], and the N1891K in the RdRp may be important for polymerase activity [24], while their effect on viral pathogenicity and pathogenesis remains unclear. Through functional mutation screening, Choi et al. showed the P102A and K211R in the NS protein could inhibit the tumour progression locus 2-mediated IL-10 production, thereby reducing the mortality in SFTSV-infected mice [25]. However, neither functional mutations in NS had been determined in naturally circulating virus strains, leaving their clinical significance unable to be confirmed.

A very recent study by Yun et al. had characterized 116 SFTSV sequences from Korean patients, determining a specific genotype associated with a higher CFR [26]; however, only a limited number of clinical cases were identified within each viral genotype, without considering the variation from hospitals capacity or treatment bias. Differing from previous studies, the current research bears the advantage of recruiting a large case-cohort at a single centre, where the same standard treatment regimens were followed, and the final outcome of the patients was determined by follow-up, in which manner, CFR could be regarded as an indicator of the virulence of the virus. The sequences have been determined directly from patient specimens, thus not affected by nucleotide mutations that might have occurred during the isolation process. Moreover, a total of 19 amplicons covering the SFTSV genome were obtained by using a high-fidelity and hot-start PCR enzyme-based RT–PCR, which could reduce the potential error rate derived from reverse transcriptase. Thus, in an unbiased way, we determined one unique cluster of SFTSV (clade IV) showing a CFR of 32.9%, which was higher than that of patients infected with other clades, also higher than that of the average level in the same hospital [3]. Survival analysis that controlled for the effect of age, sex, and hospitalization delay disclosed increased hazard ratios between 1.327 and 2.916, which translates to a 33% to 192% increased risk of death.

Further analysis of viral loads and viremia duration revealed no potential biological significance from more rapid viral replication for the clade IV. Interestingly, a pronounced inflammation was produced from the patients and PBMCs with clade IV infection or inoculation, in agreement with the previously determined association between severe SFTS and uncontrolled inflammatory response, which similarly involved elevated levels of CXCL10, granulocyte-CSF (G-CSF), IL-6, IL-10, IL-1β, MIP-1α, and MCP-1 in patients with the severe or fatal disease [17,27,28]. The hyper-inflammation response in SFTSV infection might disturb the coagulation system and promote the development of DIC, which underpins the causative correlation between clade IV and higher odds of death from SFTS [3,27,29].

We demonstrated a co-mutation pattern characterized by the combination of multiple mutations in NS, Gc, and RdRp that corresponded to this specific clade IV. The co-mutation pattern II involving S207P and Q245H in NS was related to a more robust production of cytokines. Considering the critical importance of NS in mediating systematic inflammatory activation [25,30], we postulate that the mutant strain might act by affecting its interaction with host proteins, and most likely responsible for the catastrophic immunopathology of SFTS, which hampers antiviral activity efficacy in the severe SFTS and drives fatal disease progression. Nevertheless, a detailed mechanism is needed to be investigated through further functional studies *in vitro* or in animal models based on reverse genetics. The mutation-related pathogenicity needs to be further investigated in other infectious lethal animal models such as ferret [31], in addition to the anti-IFNAR1-blocking antibody pre-treated mouse model.

Reassortment event is widespread among viruses with segmented genomes, including Bunyavirus [32]. Natural reassortment has been reported for SFTSV of various sources, including human patients, ticks, and animals [13,14]; however, their role in the pathogenicity and transmissibility remained uninvestigated. Here, we likewise demonstrated the occurrence of reassortment events in 1.7% SFTSV strains from human patients, which, however, harboured no correlation with fatality in our analysis.

When deciphered from the epidemiological point, the clade IV was not increasing in frequency along the study years, thus less likely to be a recent emergency event. However, a spatial pattern was indeed shown, with more prevalence of clade IV seen in three hotspots in southern region of Xinyang City, and featured by a disperse from south to north over time (Figure S10). We see no reason why this spatial clustering was specific to these regions. Nonetheless, it can be inferred that individuals exposed to SFTSV infection in the region with clade IV as the predominant strain were at high risk of severe disease or lethal outcomes. Based on the phylogenetic characterization of human patients-related SFTSV genome sequences available from GenBank, we also inferred the existence of clade IV in other endemic regions other than Xinyang (Figure S9), suggesting a wider distribution of this highly lethal clade in China. However, SFTSV clade IV was not found in Japan and South Korea (Figure S11). Likewise, SFTSV genotype B-2 with high pathogenicity prevalent in South Korea identified by Yun et al. [26] was not determined in any of the currently sequenced virus strains. Therefore, the distinct composition of circulated virus genotypes may contribute to the varied mortality in different SFTS endemic regions, and geography-related virus genetic and pathogenic diversity needs further investigation.

It is noteworthy that neutralization activity against one clade by convalescent serum was remarkably reduced by other clades, such as between clades I and IV, and between clades II and IV, thus raising concerns that the variants can evade immune responses elicited by natural infections with the prototype strain. During the process of development of a vaccine, the efficacy of the vaccines should fully consider these clades and the reduced effect of these predominant clades.

The study had several limitations. The association between clade IV and increased fatality was only determined in one region, which warrants further validation from an increased sequencing surveillance effort in wider regions. Second, clade IV was also identified among patients without severe disease, indicating that both host and viral factors influence an individual’s propensity to develop fatal outcomes. How each factor contributes to the clinical sequelae remained to be investigated. Third, potential selection bias might be induced in the *in vitro* and animal studies of SFTSV pathogenicity, due to the small number of virus strains, which warrants an expanded study by using a higher variety of strains.

In conclusion, we found multiple genomic variations of SFTSV continuously circulating and causing human infections in a hotspot endemic region and uncovered one specific viral clade that was related to a higher incidence of fatal outcomes post infection. Viral mutants especially those involving the NS may result in stronger systemic inflammation and pathology in human patients, thus leading to an elevated CFR. The biological significance of this clade and the key mutations related to SFTS fatality need further investigation by exploring its phenotypic modifications in both *in vitro* and animal studies. The clade IV represents 9.1% of the patients reported in the hotspot of SFTS; therefore, molecular surveillance for the highly lethal strain in the endemic region should be alerted in public health and clinical diagnosis. Expanded surveillance and sequencing on the circulating strains with a wide scope might help to identify more genetic variants, thus improving our understanding of their public health impact.

## Supplementary Material

Supplemental Material

## References

[1] Yu XJ, Liang MF, Zhang SY, et al. Fever with thrombocytopenia associated with a novel bunyavirus in China. N Engl J Med. 2011 Apr 21;364(16):1523–1532. 21410387 10.1056/NEJMoa1010095 PMC3113718

[2] Li JC, Wang YN, Zhao J, et al. A review on the epidemiology of severe fever with thrombocytopenia syndrome. Chin J Epidemiol. 2021 Dec 10;42(12):2226–2233. 10.3760/cma.j.cn112338-20210529-00439 34954991

[3] Li H, Lu QB, Xing B, et al. Epidemiological and clinical features of laboratory-diagnosed severe fever with thrombocytopenia syndrome in China, 2011-17: a prospective observational study. Lancet Infect Dis. 2018 Oct;18(10):1127–1137. 30054190 10.1016/S1473-3099(18)30293-7

[4] Kim YR, Yun Y, Bae SG, et al. Severe fever with thrombocytopenia syndrome virus infection, South Korea, 2010. Emerg Infect Dis. 2018 Nov;24(11):2103–2105. 30334706 10.3201/eid2411.170756 PMC6199997

[5] Takahashi T, Maeda K, Suzuki T, et al. The first identification and retrospective study of severe fever with thrombocytopenia syndrome in Japan. J Infect Dis. 2014 Mar;209(6):816–827. 24231186 10.1093/infdis/jit603 PMC7107388

[6] Tran XC, Yun Y, Van An L, et al. Endemic severe fever with thrombocytopenia syndrome, Vietnam. Emerg Infect Dis. 2019 May;25(5):1029–1031. 31002059 10.3201/eid2505.181463 PMC6478219

[7] Win AM, Nguyen YTH, Kim Y, et al. Genotypic heterogeneity of Orientia tsutsugamushi in scrub typhus patients and thrombocytopenia syndrome co-infection, Myanmar. Emerg Infect Dis. 2020 Aug;26(8):1878–1881. 32687023 10.3201/eid2608.200135 PMC7392420

[8] Zhuang L, Sun Y, Cui XM, et al. Transmission of severe fever with thrombocytopenia syndrome virus by Haemaphysalis longicornis ticks, China. Emerg Infect Dis. 2018 May;24(5):868–871. 10.3201/eid2405.151435 PMC5938789 29664718

[9] Tang X, Wu W, Wang H, et al. Human-to-human transmission of severe fever with thrombocytopenia syndrome bunyavirus through contact with infectious blood. J Infect Dis. 2013 Mar 1;207(5):736–739. 23225899 10.1093/infdis/jis748

[10] Jiang XL, Zhang S, Jiang M, et al. A cluster of person-to-person transmission cases caused by SFTS virus in Penglai, China. Clin Microbiol Infect. 2015 Mar;21(3):274–279. 25687766 10.1016/j.cmi.2014.10.006

[11] Koga S, Takazono T, Ando T, et al. Severe fever with thrombocytopenia syndrome virus RNA in semen, Japan. Emerg Infect Dis. 2019 Nov;25(11):2127–2128. 31625854 10.3201/eid2511.190061 PMC6810197

[12] Mendoza CA, Ebihara H, Yamaoka S. Immune modulation and immune-mediated pathogenesis of emerging tickborne Banyangviruses. Vaccines (Basel). 2019 Sep 20;7(4):125. 10.3390/vaccines7040125 PMC6963857 31547199

[13] Yoshikawa T, Shimojima M, Fukushi S, et al. Phylogenetic and geographic relationships of severe fever with thrombocytopenia syndrome virus in China, South Korea, and Japan. J Infect Dis. 2015 Sep 15;212(6):889–898. 25762790 10.1093/infdis/jiv144

[14] Wu X, Li M, Zhang Y, et al. Novel SFTSV phylogeny reveals new reassortment events and migration routes. Virol Sin. 2021 Apr;36(2):300–310. 32960400 10.1007/s12250-020-00289-0 PMC8087752

[15] Shi J, Hu S, Liu X, et al. Migration, recombination, and reassortment are involved in the evolution of severe fever with thrombocytopenia syndrome bunyavirus. Infect Genet Evol. 2017 Jan;47:109–117. 27884653 10.1016/j.meegid.2016.11.015

[16] Zhan J, Wang Q, Cheng J, et al. Current status of severe fever with thrombocytopenia syndrome in China. Virol Sin. 2017 Feb;32(1):51–62. 28251515 10.1007/s12250-016-3931-1 PMC6598917

[17] Liu W, Lu QB, Cui N, et al. Case-fatality ratio and effectiveness of ribavirin therapy among hospitalized patients in China who had severe fever with thrombocytopenia syndrome. Clin Infect Dis. 2013 Nov;57(9):1292–1299. 23965284 10.1093/cid/cit530

[18] Deng L, Liu M, Hua S, et al. Network of co-mutations in Ebola virus genome predicts the disease lethality. Cell Res. 2015 Jun;25(6):753–756. 25976404 10.1038/cr.2015.54 PMC4456622

[19] Li H, Zhang LK, Li SF, et al. Calcium channel blockers reduce severe fever with thrombocytopenia syndrome virus (SFTSV) related fatality. Cell Res. 2019 Sep;29(9):739–753. 31444469 10.1038/s41422-019-0214-z PMC6796935

[20] Li S, Li H, Zhang YL, et al. SFTSV infection induces BAK/BAX-dependent mitochondrial DNA release to trigger NLRP3 inflammasome activation. Cell Rep. 2020 Mar 31;30(13):4370–4385.e7. 32234474 10.1016/j.celrep.2020.02.105

[21] Stern A, Yeh MT, Zinger T, et al. The evolutionary pathway to virulence of an RNA virus. Cell. 2017 Mar 23;169(1):35–46.e19. 28340348 10.1016/j.cell.2017.03.013 PMC5787669

[22] Tsuda Y, Igarashi M, Ito R, et al. The amino acid at position 624 in the glycoprotein of SFTSV (severe fever with thrombocytopenia virus) plays a critical role in low-pH-dependent cell fusion activity. Biomed Res. 2017;38(2):89–97. 28442665 10.2220/biomedres.38.89

[23] Tani H, Kawachi K, Kimura M, et al. Identification of the amino acid residue important for fusion of severe fever with thrombocytopenia syndrome virus glycoprotein. Virology. 2019 Sep;535:102–110. 31299486 10.1016/j.virol.2019.06.014

[24] Noda K, Tsuda Y, Kozawa F, et al. The polarity of an amino acid at position 1891 of severe fever with thrombocytopenia syndrome virus L protein is critical for the polymerase activity. Viruses. 2020 Dec 27;13(1):33. 10.3390/v13010033 PMC7823514 33375489

[25] Choi Y, Park SJ, Sun Y, et al. Severe fever with thrombocytopenia syndrome phlebovirus non-structural protein activates TPL2 signalling pathway for viral immunopathogenesis. Nat Microbiol. 2019 Mar;4(3):429–437. 30617349 10.1038/s41564-018-0329-x PMC6548314

[26] Yun SM, Park SJ, Kim YI, et al. Genetic and pathogenic diversity of severe fever with thrombocytopenia syndrome virus (SFTSV) in South Korea. JCI Insight. 2020 Jan 30;5(2):e129531. 10.1172/jci.insight.129531 PMC7098714 31877113

[27] Li H, Li X, Lv S, et al. Single-cell landscape of peripheral immune responses to fatal SFTS. Cell Rep. 2021;37(8):110039. 34818556 10.1016/j.celrep.2021.110039

[28] Kwon JS, Jin S, Kim JY, et al. Viral and immunologic factors associated with fatal outcome of patients with severe fever with thrombocytopenia syndrome in Korea. Viruses. 2021 Nov 23;13(12):2351. 34960620 10.3390/v13122351 PMC8703577

[29] Liu Q, He B, Huang SY, et al. Severe fever with thrombocytopenia syndrome, an emerging tick-borne zoonosis. Lancet Infect Dis. 2014 Aug;14(8):763–772. 24837566 10.1016/S1473-3099(14)70718-2

[30] Sun Q, Jin C, Zhu L, et al. Host responses and regulation by NFκB signaling in the liver and liver epithelial cells infected with a novel tick-borne bunyavirus. Sci Rep. 2015 Jul 2;5:11816. 26134299 10.1038/srep11816 PMC4488873

[31] Park SJ, Kim Y, Park A, et al. Ferret animal model of severe fever with thrombocytopenia syndrome phlebovirus for human lethal infection and pathogenesis. Nat Microbiol. 2019 Mar;4(3):438–446. 30531978 10.1038/s41564-018-0317-1 PMC6548318

[32] Briese T, Calisher CH, Higgs S. Viruses of the family Bunyaviridae: are all available isolates reassortants? Virology. 2013 Nov;446(1–2):207–216. 24074583 10.1016/j.virol.2013.07.030

