# Supplementary material for: Effect of genomic variations in severe fever with thrombocytopenia syndrome virus on the disease lethality
Source: Emerg Microbes Infect. 2022 Jun 20;11(1):1672–82. doi: 10.1080/22221751.2022.2081617 (PMC9225783; doi:10.1080/22221751.2022.2081617)
Supplement: Supplemental Material [file TEMI_A_2081617_SM4785.docx]

**Appendix**

**Methods**

**Criterion of SFTS diagnosis**

A laboratory-confirmed patient with SFTSV infection was defined as meeting one or more of the following criteria: (1) isolation of SFTSV in cell culture (2) detection of SFTSV RNA by a molecular method (3) seroconversion or ≥4 fold increase of antibody titers between two serum samples collected at least 2 weeks apart.

**Immune mediator detection by Luminex xMAP technology**

Serum samples serially collected from the patients at admission and during hospitalization were used to determine levels of immune mediators by using Bio-Plex Pro Human Cytokine Screening Panel (Bio-Rad) with Luminex xMAP technology. Data analysis was conducted with the Bio-Plex Manager 6.1.1 software (Bio-Rad). A total of 48 immune mediators were tested, including bFGF, Eotaxin, G-CSF, GM-CSF, IFN-γ, IL-1β, IL-RA, IL-1α, IL-2Rα, IL-3, IL-12p40, IL-16, IL-2, IL-4, IL-5, IL-6, IL-7, IL-8, IL-9, GRO-α, HGF, IFN-α2, LIF, MCP-3, IL-10, IL-12p70, IL-13, IL-15, IL-17A, IP-10, MACP-1, MIG, β-NGF, SCF, SCGF-β, SDF-1α, MIP-1α, MIP-1β, PDFG-BB, RANTES, TNF-α, VEGF, CTACK, MIF, TRAIL, IL-18, M-CSF, and TNF-β.

**Neutralization assay**

The neutralization titers of serum samples from convalescent SFTS patients collected at three months post symptom onset was determined against four SFTSV genotypes (clades I-IV) using an enzyme-linked immunosorbent assay (ELISA)-based microneutralization test (EMNT). Heat inactivated (30 mins at 56 °C) serum samples were serially diluted two-fold starting from 1:20 to 1:320, and then mixed with equivalent volume of 100 × TCID_50_ viral aliquots separately. The mixtures were incubated for 1.5 h at 37 °C, transferred to Vero cells in 96-well plates for 1.5 h at 37 °C. The plates were washed 1 time by PBS. Eight wells contained 50 μl of diluted virus, 50 μl diluent and 100 μl of Vero cells were set as positive controls and Eight wells contained 100 μl diluent and 100 μl of Vero cells as were set as negative controls. The plates were then incubated for 72 hours at 37 °C and 5% CO_2_. The infected cells were fixed with pre-cold 80% acetone. The anti-SFTSV NP mouse monoclonal antibody was used at a 1: 2000 dilution in blocking buffer (PBST, 1% bovine serum albumin) per well for 1 h at 37 °C. The plates were washed five times with PBST. Then, HRP-conjugated goat anti-mouse IgG was used at 1: 2000 dilution in blocking buffer per well for 0.5 h at 37 °C. The plates were washed five times with PBST again. Followed detecting antibody in the presence of the 3,3',5,5'-tetramethylbenzidine (TMB) substrate for 10 mins. The reaction was stopped by the addition of stop solution. The absorbance was measured by absorbance microplate reader (BMG LABTECH) at 450 (A450) and 630 (A630) nm. The virus neutralization titers were calculated as following: A = A450 - A630; X = [(average A_virus positive control wells_) - (average A_cell negative control wells_)]/2 + (average A_cell negative control wells_). All sample dilutions were tested in duplicate and considered positive for neutralizing activity if either A_serum_ was less than X.

**SFTSV culture**

The growth kinetics of SFTSV was examined by seeding in Vero cells at a density of 1 × 10^5^ cells per well in 24-well plates. Cells were infected with virus strains of four clades at a multiplicity of infection (MOI) of 1. Cells were harvested and the total cellular mRNA was extracted using the QIAamp Viral RNA Mini Kit (Qiagen), according to the manufacturer’s instructions. Relative quantitation by real-time reverse-transcription PCR based on SFTSV L segment and monkey GAPDH gene was performed using One-Step TB green Primescript RT-PCR Kit (Takara) as previously described.^1^ Virus titer was examined from the culture supernatant serially harvested at 12, 24, 36, 48, and 72 hours post infection by using immunological focus assay as previously described.^2^

**Appendix Figure Legend**

**Appendix Figure 1. Number and proportion of four common SFTSV clades in patients with SFTS.**

The number (up) and proportion (down) of four viral clades were show in patients with SFTS in regarding to the month (A), age (B) and sex (C). The month indicate the symptom onset time of the patient. The monthly proportion of the four viral clades was assessed using the yearly combined data across the study period.





**Appendix Figure 2. Titers of IgM and IgG antibodies in patients infected with four virus clades based on S, M, and L segments.**

Dynamic changes in titers of IgM (A) and IgG antibodies (B) in patients infected with four viral clades. Datapoints show exact values; horizontal lines show median values; error bars show interquartile-range.





**Appendix Figure 3. Neutralization of four SFTSV viral clades in convalescent serum samples.**

The reciprocal NAb titers against four SFTSV strains representing four viral clades were determined in 40 convalescent serum samples collected from patients infected with SFTSV clade I (n=10) (A), clade II (n=10) (B), clade III (n=10) (C), and clade IV (n=10) (D), respectively. The mean value and fold reduction of neutralizing antibody titers against original virus and other tested clades were shown above the graph. NAb=neutralization antibody.

**
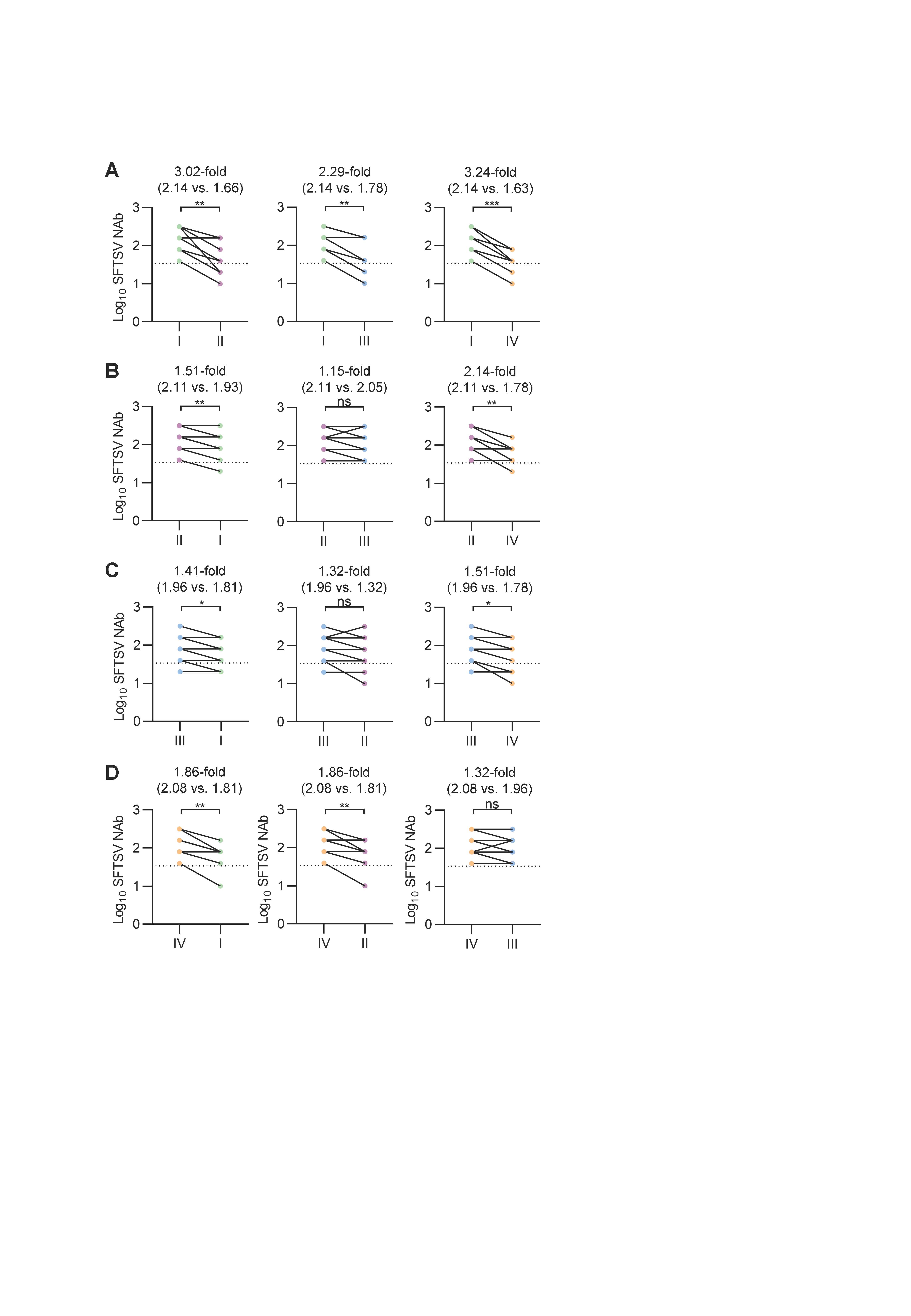
**

**Appendix Figure 4. *In vitro* study on viral replication and cytokine induction of different SFTSV clades.**

Kinetics of viral replication of SFTSV in Vero cells (left: supernatant viral titers; right: intracellular viral RNA levels) (A). Induction of cytokines (IL-1β, IL-6, and IL-10) in PBMCs from healthy individuals after inoculated with different SFTSV clades (B; up: intracellular cytokine levels; down: supernatant cytokine levels). ANOVA test was used for the comparison among multiple groups.





**Appendix Figure 5. Animal study on viral replication and cytokine induction of different SFTSV clades.**

Analysis of survival probability in anti-IFNAR1 IgG antibody treated mice (n=10 for each virus strain) after infection with four SFTSV clades (A). SFTSV titers in the spleen samples (n=4 for each virus strain) determined by immunological focus assay (B). Concentrations of IL-1β in the spleen samples determined by ELISA (C) and immunohistochemistry staining (D). ANOVA test was used for the comparison among multiple groups.





**Appendix Figure 6.** Comparison of nucleotide and amino acid sequence identity of SFTSV strains.

Pairwise sequence comparison of SFTSV strains within the same clade (A). Normalized sequence identities of each clade SFTSV strains to the reference strain HN6/China/2010 (B). The upper and lower bars represent the maximum and minimum values, respectively. The box shows identities ranging from 25% to 75% of the total values, and the solid line in the middle indicates the median.





**Appendix Figure 7. Amino acid mutations in each protein of SFTSV strains.**

The matrix represents amino acid mutations in 791 non-reassorted SFTSV strains obtained from patients with SFTS. The amino acid mutations with a frequency less than 2% were not shown.


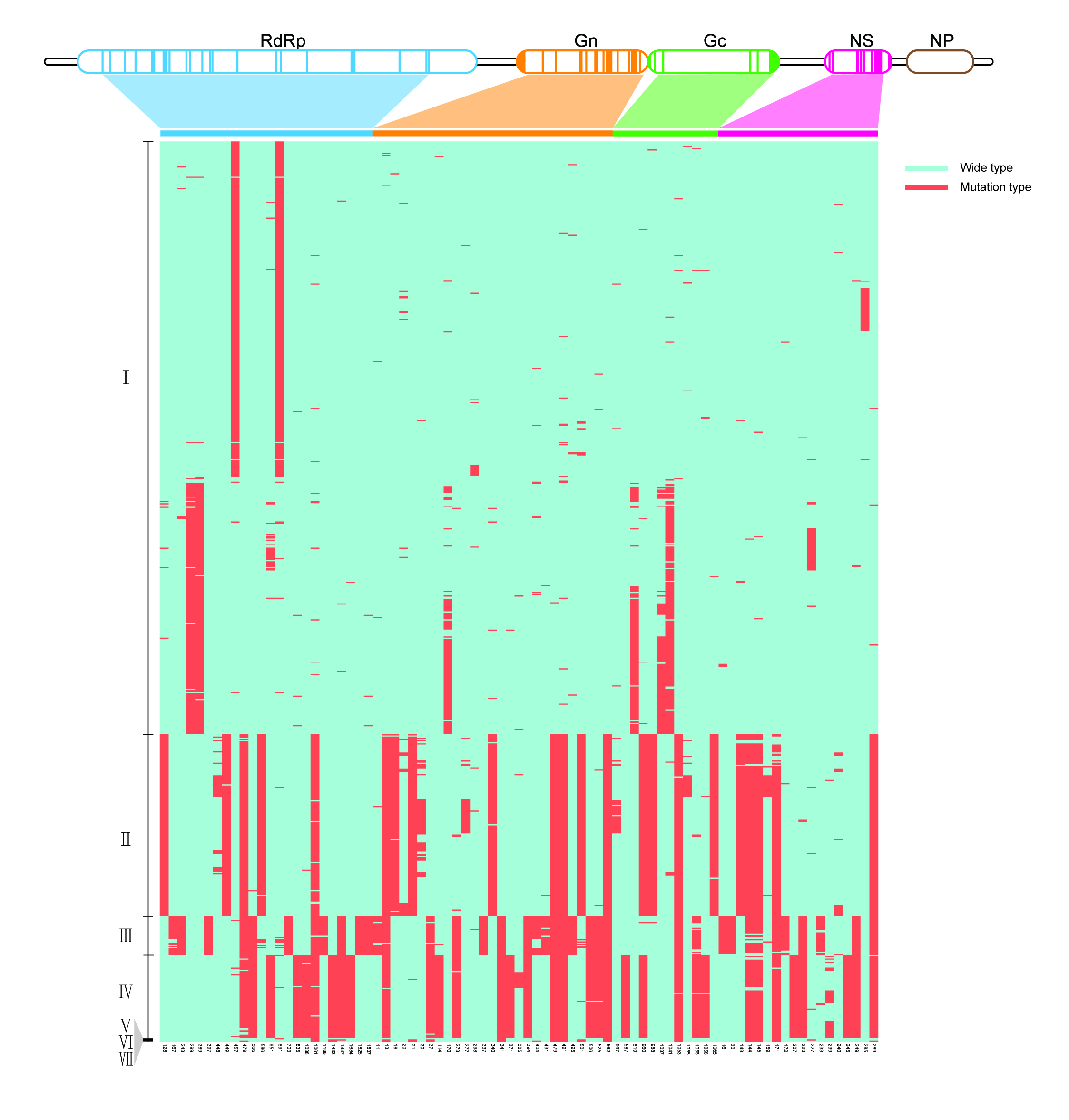


**Appendix Figure 8. Levels of immune mediators in serum from patients infected with SFTSV.**

Comparison of serum concentrations of CXCL9, IL-10, IL-6, IP-10, M-CSF, and IL-1β, among patients infected with wide type virus, patients infected with co-mutation pattern II virus, and HC (A). with Detailed information about the patients infected with wide type virus or co-mutation pattern II virus was senn in Table S17. Datapoints show exact values; horizontal lines show median values; error bars show interquartile-range. Comparison of serum concentrations of the six immune mediators tested on admission and during hospitalization in patients infected with wide type and co-mutation pattern II virus (B). Datapoints show median values; error bars show interquartile-range. *p<0.05, **p<0.01, ***p<0.001. HC=healthy control. ns=no significance.





**Appendix Figure 9. Phylogenetic analysis and geographic distribution of available SFTSV S segments from human patients in the GenBank.**

The phylogenetic tree was constructed using nucleotide sequences of 424 SFTSV S segments from Chinese human patients available in GenBank (A), with detailed information about sequences shown in Table S3. A total of 10 clades, including seven same clades (I to VII) as identified in Xinyang city in the current study and three new clades (VIII to X), were identified. The evolutionary branches of each genotype were collapsed, and the number of sequences was indicated in parentheses. Clade X represents the Genotype B-2 reported by Yun et al [3]. Geographic distribution of different SFTSV clades was shown at the province level (B). Pie charts indicate the proportion of different SFTSV clades in each province.





**Appendix Figure 10. Geographic distribution of patients infected with SFTSV clade IV.**

Geographic distribution of patients infected with SFTSV clade IV residing in Xinyang city, was shown with colored points representing different years of disease onset. Three dashed ellipses indicate the hotspot regions of patients located.





**Appendix Figure 11. Phylogenetic analysis of SFTSV S segment sequences from Japan and South Korea.**

The phylogenetic tree was constructed based on SFTS S segment (1656 bp) using the Maximum-likelihood model. Sequence from Heartland virus was used as outgroup. The confidence of the phylogenetic tree was tested using 1000 bootstrap replications. The colored labels indicate the sequences from China, and the black labels indicate the sequences from Japan and South Korea. Clade J1 represents the Genotype B-2 reported by Yun et al [3].





**Appendix Table 1. Primers used for nested RT-PCR assays.**

| Segment | First round | |  | Second round | |
| --- | --- | --- | --- | --- | --- |
|  | Primer name | Primer sequence (5’-3’) |  | Primer name | Primer sequence (5’-3’) |
| L | L1F | ACACAAAGACGCCCAGATGA |  | LN-1F | ACGCCCAGATGAACTTGGAA |
|  | L1R | GACATCTAGAAGGCACTTTGC |  | LN-1R | AGCCTGAGTCGGTCTTGATGT |
|  | L2F | AGGAGCAACAAGCAAACATCAT |  | LN-2F | GAATCGAGGGACAGTCAAAC |
|  | L2R | CAATGTACCAGCTGTTCACCA |  | LN-2R | TTGTGGACCGGCCTGAGATG |
|  | L3F | TGGATTGCATGGTGCGAATTG |  | LN-3F | GAACATTCCATGCCATCTCAG |
|  | L3R | GATCAGATGACCTAGACTCAG |  | LN-3R | GTGAGCTAAAAACCTTAGGTC |
|  | L4F | CATGCCAGCYAAATTCCACAG |  | LN-4F | TTGGGCTGCCATTTCCATGTT |
|  | L4R | TTCTTGTTGGCAGCTCTCCTG |  | LN-4R | GGACTCCAGGATTCTCATCT |
|  | L5F | GGAACTCTCAGCCACTCTGTT |  | LN-5F | TGAACAGGATGGGCCTTCCTG |
|  | L5R | GTAGAGAAGGCCTCTATGATC |  | LN-5R | CTTGTACTCCTCAGTGTATGG |
|  | L6F | TAGCCTAGAAGCTGAGAAGAG |  | LN-6F | TATCTCCATCCTCAAGCATGT |
|  | L6R | CCTTGGGTCTTCCTATCATTT |  | LN-6R | AGGAGAACTGAGGCATGTGA |
|  | L7F | ACACTGATATATCAGAGTCAGC |  | LN-7F | CAATCGAGACCTCTTCTCCT |
|  | L7R | ACACAAAGACCGCCCAGATC |  | LN-7R | ACCGCCCAGATCTTAAGGAA |
| M | M1F | ACACAAAGACGGCCAACAATG |  | MN-1F | ACGGCCAACAATGATGAAAGTC |
|  | M1R | GTCTGGGAATTCACTTTGGC |  | MN-1R | TCTTCAGCTCCAGAAATGTC |
|  | M2F | GATAGGGTTCTCTGGATAGG |  | MN-2F | AGGTGATGTTGCTTGTCAGC |
|  | M2R | ATCCCTCCATATGACACAACG |  | MN-2R | TGCACAAGTGAGCATCTACAC |
|  | M3F | GTRCAAGAGAGCTCATCCAAG |  | MN-3F | TGTGCTAYAAGGAAGGGACTG |
|  | M3R | TTCTTTGCAGGGTAGCACTG |  | MN-3R | GGATTTTTTAGAAACTCACGAC |
|  | M4F | GAATTCACATTTGAGGGTAGTT |  | MN-4F | AGTTGCATGTTCCCAGATGG |
|  | M4R | ACCGGGCATCAGGAACAAAAT |  | MN-4R | TGAGACATTTTGAGTCYGGAC |
|  | M5F | TGTGATGAGATGGTCCATGCT |  | MN-5F | AAGGGAGCGGAAATATGAAGG |
|  | M5R | ACCTCCATATCTGAGCCCAA |  | MN-5R | ACACTCACACCCTTGAAGAC |
|  | M6F | AGAGCTAACAATGCCCTCAG |  | MN-6F | GAGGACATTCCACCCCATGA |
|  | M6R | AGACTTTGGCGCCTGTCATGC |  | MN-6R | ACATTTGTCACYTCCCCTGTG |
|  | M7F | CCCCCTGGACATCACAGCTAT |  | MN-7F | CTGTYAATTATAGAGGCCTTCG |
|  | M7R | CCCATTGCCAAACAAGGCAT |  | MN-7R | CTTGGGATATTTGCCCCTGT |
|  | M8F | ACTGAACTGTGGGGGACATG |  | MN-8F | TTGTGGATGGCAGCTACATG |
|  | M8R | ACACAAAGACCGGCCAACACT |  | MN-8R | AACACTTCAAYRGAACCTCCAT |
| S | S1F | ACACAAAGAACCCCCAAAAAAGG |  | SN-1F | AACCCCCAAAAAAGGAAAGACG |
|  | S1R | CCCTTGGCCTTCAGCCACTT |  | SN-1R | AAGACAGAGTTCACAGCAGC |
|  | S2F | GCAAGATGCCTTCACCAAGA |  | SN-2F | GAGCCAGCAAGACAGAAGTT |
|  | S2R | ACAGTGTCTTGGATGAGGATG |  | SN-2R | CCAYATCTGATGGCACACTAT |
|  | S3F | GACAAAATTAGACCTCCTTCG |  | SN-3F | ACCAATGGCTGGCCAATCTCT |
|  | S3R | TGTACTACAAGGACATGAGG |  | SN-3R | TCAAGAACAGCTGGGCAATG |
|  | S4F | TATCATGTCCCCTTCAAAAAG |  | SN-4F | GGCCCCGCCAGTTCTCTCT |
|  | S4R | ACACAAAGAACCCCCTTCATTT |  | SN-4R | AACCCCCTTCATTTGGAAACCA |

**Appendix Table 2. SFTSV S segment sequences determined in the current study with accession number, source, location, name of isolate, and viral genotype.**

| Segment | Accession No. | Host | Location | Year | Isolate | Genotype |
| --- | --- | --- | --- | --- | --- | --- |
| S | MN510176 | Homo sapiens | China: Henan | 2011 | 11-China_Henan-1 | Ⅰ |
|  | MN510177 | Homo sapiens | China: Henan | 2011 | 11-China_Henan-13 | Ⅰ |
|  | OM451560 | Homo sapiens | China: Henan | 2011 | 11-China_Henan-2 | Ⅳ |
|  | MN510181 | Homo sapiens | China: Henan | 2011 | 11-China_Henan-266 | Ⅱ |
|  | MN510182 | Homo sapiens | China: Henan | 2011 | 11-China_Henan-273 | Ⅰ |
|  | MN510194 | Homo sapiens | China: Henan | 2012 | 12-China_Henan-103 | Ⅰ |
|  | OM451561 | Homo sapiens | China: Henan | 2012 | 12-China_Henan-108 | Ⅱ |
|  | OM451562 | Homo sapiens | China: Henan | 2012 | 12-China_Henan-109 | Ⅳ |
|  | MN510195 | Homo sapiens | China: Henan | 2012 | 12-China_Henan-110 | Ⅰ |
|  | OM451563 | Homo sapiens | China: Henan | 2012 | 12-China_Henan-114 | Ⅳ |
|  | MN510196 | Homo sapiens | China: Henan | 2012 | 12-China_Henan-127 | Ⅱ |
|  | OM451564 | Homo sapiens | China: Henan | 2012 | 12-China_Henan-13 | Ⅰ |
|  | MN510198 | Homo sapiens | China: Anhui | 2012 | 12-China_Anhui-154 | Ⅰ |
|  | OM451565 | Homo sapiens | China: Henan | 2012 | 12-China_Henan-194 | Ⅰ |
|  | OM451566 | Homo sapiens | China: Hubei | 2012 | 12-China_Hubei-21 | Ⅱ |
|  | OM451567 | Homo sapiens | China: Henan | 2012 | 12-China_Henan-220 | Ⅰ |
|  | MN510199 | Homo sapiens | China: Henan | 2012 | 12-China_Henan-224 | Ⅰ |
|  | OM451568 | Homo sapiens | China: Henan | 2012 | 12-China_Henan-23 | Ⅰ |
|  | OM451569 | Homo sapiens | China: Henan | 2012 | 12-China_Henan-234 | Ⅰ |
|  | OM451570 | Homo sapiens | China: Henan | 2012 | 12-China_Henan-237 | Ⅰ |
|  | OM451571 | Homo sapiens | China: Hebei | 2012 | 12-China_Hebei-24 | Ⅰ |
|  | MN510200 | Homo sapiens | China: Hubei | 2012 | 12-China_Hubei-242 | Ⅰ |
|  | MN510202 | Homo sapiens | China: Henan | 2012 | 12-China_Henan-270 | Ⅰ |
|  | MN510203 | Homo sapiens | China: Henan | 2012 | 12-China_Henan-271 | Ⅰ |
|  | MN510204 | Homo sapiens | China: Henan | 2012 | 12-China_Henan-279 | Ⅱ |
|  | OM451572 | Homo sapiens | China: Henan | 2012 | 12-China_Henan-30 | Ⅱ |
|  | OM451573 | Homo sapiens | China: Henan | 2012 | 12-China_Henan-32 | Ⅰ |
|  | MN510206 | Homo sapiens | China: Henan | 2012 | 12-China_Henan-324 | Ⅱ |
|  | OM451574 | Homo sapiens | China: Henan | 2012 | 12-China_Henan-36 | Ⅰ |
|  | OM451575 | Homo sapiens | China: Henan | 2012 | 12-China_Henan-37 | Ⅳ |
|  | OM451576 | Homo sapiens | China: Henan | 2012 | 12-China_Henan-40 | Ⅳ |
|  | OM451577 | Homo sapiens | China: Henan | 2012 | 12-China_Henan-41 | Ⅰ |
|  | MN510186 | Homo sapiens | China: Henan | 2012 | 12-China_Henan-48 | Ⅰ |
|  | OM451578 | Homo sapiens | China: Henan | 2012 | 12-China_Henan-5 | Ⅱ |
|  | OM451579 | Homo sapiens | China: Henan | 2012 | 12-China_Henan-54 | Ⅰ |
|  | OM451580 | Homo sapiens | China: Henan | 2012 | 12-China_Henan-56 | Ⅱ |
|  | OM451581 | Homo sapiens | China: Henan | 2012 | 12-China_Henan-64 | Ⅰ |
|  | OM451582 | Homo sapiens | China: Henan | 2012 | 12-China_Henan-65 | Ⅰ |
|  | MN510184 | Homo sapiens | China: Henan | 2012 | 12-China_Henan-7 | Ⅰ |
|  | OM451583 | Homo sapiens | China: Henan | 2012 | 12-China_Henan-9 | Ⅰ |
|  | OM451584 | Homo sapiens | China: Henan | 2012 | 12-China_Henan-90 | Ⅶ |
|  | OM451585 | Homo sapiens | China: Henan | 2013 | 13-China_Henan-100 | Ⅲ |
|  | OM451586 | Homo sapiens | China: Henan | 2013 | 13-China_Henan-101 | Ⅰ |
|  | OM451587 | Homo sapiens | China: Henan | 2013 | 13-China_Henan-110 | Ⅰ |
|  | OM451588 | Homo sapiens | China: Henan | 2013 | 13-China_Henan-112 | Ⅱ |
|  | MN510209 | Homo sapiens | China: Hubei | 2013 | 13-China_Hubei-113 | Ⅰ |
|  | OM451589 | Homo sapiens | China: Henan | 2013 | 13-China_Henan-116 | Ⅰ |
|  | OM451590 | Homo sapiens | China: Henan | 2013 | 13-China_Henan-118 | Ⅰ |
|  | OM451591 | Homo sapiens | China: Henan | 2013 | 13-China_Henan-119 | Ⅰ |
|  | OM451592 | Homo sapiens | China: Henan | 2013 | 13-China_Henan-120 | Ⅰ |
|  | OM451593 | Homo sapiens | China: Henan | 2013 | 13-China_Henan-122 | Ⅱ |
|  | MN510210 | Homo sapiens | China: Hubei | 2013 | 13-China_Hubei-124 | Ⅰ |
|  | OM451594 | Homo sapiens | China: Henan | 2013 | 13-China_Henan-126 | Ⅳ |
|  | OM451595 | Homo sapiens | China: Henan | 2013 | 13-China_Henan-128 | Ⅰ |
|  | OM451596 | Homo sapiens | China: Henan | 2013 | 13-China_Henan-129 | Ⅰ |
|  | OM451597 | Homo sapiens | China: Henan | 2013 | 13-China_Henan-130 | Ⅰ |
|  | OM451598 | Homo sapiens | China: Henan | 2013 | 13-China_Henan-132 | Ⅰ |
|  | OM451599 | Homo sapiens | China: Henan | 2013 | 13-China_Henan-139 | Ⅰ |
|  | MN510211 | Homo sapiens | China: Henan | 2013 | 13-China_Henan-141 | Ⅱ |
|  | OM451600 | Homo sapiens | China: Henan | 2013 | 13-China_Henan-142 | Ⅰ |
|  | OM451601 | Homo sapiens | China: Henan | 2013 | 13-China_Henan-144 | Ⅰ |
|  | OM451602 | Homo sapiens | China: Henan | 2013 | 13-China_Henan-147 | Ⅰ |
|  | MN510212 | Homo sapiens | China: Henan | 2013 | 13-China_Henan-154 | Ⅱ |
|  | OM451603 | Homo sapiens | China: Henan | 2013 | 13-China_Henan-155 | Ⅰ |
|  | OM451604 | Homo sapiens | China: Henan | 2013 | 13-China_Henan-160 | Ⅰ |
|  | MN510214 | Homo sapiens | China: Henan | 2013 | 13-China_Henan-166 | Ⅰ |
|  | OM451605 | Homo sapiens | China: Henan | 2013 | 13-China_Henan-170 | Ⅰ |
|  | OM451606 | Homo sapiens | China: Henan | 2013 | 13-China_Henan-177 | Ⅰ |
|  | OM451607 | Homo sapiens | China: Henan | 2013 | 13-China_Henan-178 | Ⅰ |
|  | OM451608 | Homo sapiens | China: Henan | 2013 | 13-China_Henan-181 | Ⅰ |
|  | OM451609 | Homo sapiens | China: Henan | 2013 | 13-China_Henan-192 | Ⅰ |
|  | OM451610 | Homo sapiens | China: Henan | 2013 | 13-China_Henan-195 | Ⅱ |
|  | OM451611 | Homo sapiens | China: Henan | 2013 | 13-China_Henan-198 | Ⅲ |
|  | OM451612 | Homo sapiens | China: Henan | 2013 | 13-China_Henan-200 | Ⅰ |
|  | OM451613 | Homo sapiens | China: Henan | 2013 | 13-China_Henan-201 | Ⅰ |
|  | OM451614 | Homo sapiens | China: Henan | 2013 | 13-China_Henan-212 | Ⅰ |
|  | OM451615 | Homo sapiens | China: Henan | 2013 | 13-China_Henan-213 | Ⅳ |
|  | OM451616 | Homo sapiens | China: Henan | 2013 | 13-China_Henan-219 | Ⅰ |
|  | OM451617 | Homo sapiens | China: Henan | 2013 | 13-China_Henan-221 | Ⅰ |
|  | OM451618 | Homo sapiens | China: Henan | 2013 | 13-China_Henan-224 | Ⅰ |
|  | OM451619 | Homo sapiens | China: Henan | 2013 | 13-China_Henan-229 | Ⅱ |
|  | OM451620 | Homo sapiens | China: Henan | 2013 | 13-China_Henan-238 | Ⅱ |
|  | OM451621 | Homo sapiens | China: Henan | 2013 | 13-China_Henan-24 | Ⅰ |
|  | OM451622 | Homo sapiens | China: Henan | 2013 | 13-China_Henan-240 | Ⅰ |
|  | OM451623 | Homo sapiens | China: Henan | 2013 | 13-China_Henan-246 | Ⅱ |
|  | OM451624 | Homo sapiens | China: Hubei | 2013 | 13-China_Hubei-252 | Ⅰ |
|  | OM451625 | Homo sapiens | China: Henan | 2013 | 13-China_Henan-254 | Ⅰ |
|  | OM451626 | Homo sapiens | China: Henan | 2013 | 13-China_Henan-255 | Ⅰ |
|  | OM451627 | Homo sapiens | China: Hubei | 2013 | 13-China_Hubei-256 | Ⅲ |
|  | OM451628 | Homo sapiens | China: Henan | 2013 | 13-China_Henan-258 | Ⅰ |
|  | OM451629 | Homo sapiens | China: Henan | 2013 | 13-China_Henan-262 | Ⅳ |
|  | MN510216 | Homo sapiens | China: Henan | 2013 | 13-China_Henan-263 | Ⅰ |
|  | MN510217 | Homo sapiens | China: Henan | 2013 | 13-China_Henan-264 | Ⅰ |
|  | OM451630 | Homo sapiens | China: Henan | 2013 | 13-China_Henan-266 | Ⅳ |
|  | OM451631 | Homo sapiens | China: Henan | 2013 | 13-China_Henan-271 | Ⅰ |
|  | MN510218 | Homo sapiens | China: Henan | 2013 | 13-China_Henan-275 | Ⅱ |
|  | OM451632 | Homo sapiens | China: Henan | 2013 | 13-China_Henan-279 | Ⅳ |
|  | OM451633 | Homo sapiens | China: Henan | 2013 | 13-China_Henan-280 | Ⅰ |
|  | MN510219 | Homo sapiens | China: Henan | 2013 | 13-China_Henan-288 | Ⅰ |
|  | OM451634 | Homo sapiens | China: Henan | 2013 | 13-China_Henan-29 | Ⅰ |
|  | MN510220 | Homo sapiens | China: Henan | 2013 | 13-China_Henan-292 | Ⅱ |
|  | OM451635 | Homo sapiens | China: Henan | 2013 | 13-China_Henan-293 | Ⅱ |
|  | OM451636 | Homo sapiens | China: Henan | 2013 | 13-China_Henan-294 | Ⅰ |
|  | MN510221 | Homo sapiens | China: Henan | 2013 | 13-China_Henan-295 | Ⅱ |
|  | OM451637 | Homo sapiens | China: Henan | 2013 | 13-China_Henan-297 | Ⅳ |
|  | OM451638 | Homo sapiens | China: Henan | 2013 | 13-China_Henan-299 | Ⅳ |
|  | OM451639 | Homo sapiens | China: Henan | 2013 | 13-China_Henan-30 | Ⅰ |
|  | OM451640 | Homo sapiens | China: Henan | 2013 | 13-China_Henan-300 | Ⅱ |
|  | OM451641 | Homo sapiens | China: Henan | 2013 | 13-China_Henan-302 | Ⅰ |
|  | OM451642 | Homo sapiens | China: Henan | 2013 | 13-China_Henan-304 | Ⅰ |
|  | OM451643 | Homo sapiens | China: Henan | 2013 | 13-China_Henan-31 | Ⅱ |
|  | OM451644 | Homo sapiens | China: Henan | 2013 | 13-China_Henan-310 | Ⅰ |
|  | OM451645 | Homo sapiens | China: Henan | 2013 | 13-China_Henan-311 | Ⅰ |
|  | OM451646 | Homo sapiens | China: Henan | 2013 | 13-China_Henan-313 | Ⅱ |
|  | OM451647 | Homo sapiens | China: Henan | 2013 | 13-China_Henan-320 | Ⅰ |
|  | MN510222 | Homo sapiens | China: Hubei | 2013 | 13-China_Hubei-321 | Ⅰ |
|  | OM451648 | Homo sapiens | China: Henan | 2013 | 13-China_Henan-324 | Ⅱ |
|  | OM451649 | Homo sapiens | China: Henan | 2013 | 13-China_Henan-357 | Ⅰ |
|  | OM451650 | Homo sapiens | China: Henan | 2013 | 13-China_Henan-364 | Ⅲ |
|  | OM451651 | Homo sapiens | China: Henan | 2013 | 13-China_Henan-368 | Ⅰ |
|  | OM451652 | Homo sapiens | China: Henan | 2013 | 13-China_Henan-369 | Ⅰ |
|  | OM451653 | Homo sapiens | China: Henan | 2013 | 13-China_Henan-37 | Ⅰ |
|  | OM451654 | Homo sapiens | China: Henan | 2013 | 13-China_Henan-371 | Ⅳ |
|  | OM451655 | Homo sapiens | China: Henan | 2013 | 13-China_Henan-38 | Ⅱ |
|  | OM451656 | Homo sapiens | China: Henan | 2013 | 13-China_Henan-382 | Ⅳ |
|  | OM451657 | Homo sapiens | China: Henan | 2013 | 13-China_Henan-39 | Ⅰ |
|  | OM451658 | Homo sapiens | China: Henan | 2013 | 13-China_Henan-392 | Ⅳ |
|  | MN510223 | Homo sapiens | China: Henan | 2013 | 13-China_Henan-398 | Ⅱ |
|  | OM451659 | Homo sapiens | China: Henan | 2013 | 13-China_Henan-40 | Ⅰ |
|  | OM451660 | Homo sapiens | China: Henan | 2013 | 13-China_Henan-408 | Ⅰ |
|  | OM451661 | Homo sapiens | China: Henan | 2013 | 13-China_Henan-420 | Ⅰ |
|  | OM451662 | Homo sapiens | China: Henan | 2013 | 13-China_Henan-423 | Ⅳ |
|  | OM451663 | Homo sapiens | China: Henan | 2013 | 13-China_Henan-424 | Ⅰ |
|  | MN510224 | Homo sapiens | China: Henan | 2013 | 13-China_Henan-425 | Ⅰ |
|  | OM451664 | Homo sapiens | China: Henan | 2013 | 13-China_Henan-428 | Ⅰ |
|  | MN510225 | Homo sapiens | China: Henan | 2013 | 13-China_Henan-429 | Ⅱ |
|  | OM451665 | Homo sapiens | China: Henan | 2013 | 13-China_Henan-44 | Ⅰ |
|  | OM451666 | Homo sapiens | China: Henan | 2013 | 13-China_Henan-45 | Ⅱ |
|  | OM451667 | Homo sapiens | China: Henan | 2013 | 13-China_Henan-48 | Ⅰ |
|  | OM451668 | Homo sapiens | China: Henan | 2013 | 13-China_Henan-50 | Ⅰ |
|  | OM451669 | Homo sapiens | China: Henan | 2013 | 13-China_Henan-52 | Ⅰ |
|  | OM451670 | Homo sapiens | China: Henan | 2013 | 13-China_Henan-53 | Ⅰ |
|  | OM451671 | Homo sapiens | China: Henan | 2013 | 13-China_Henan-54 | Ⅱ |
|  | OM451672 | Homo sapiens | China: Henan | 2013 | 13-China_Henan-55 | Ⅱ |
|  | OM451673 | Homo sapiens | China: Henan | 2013 | 13-China_Henan-60 | Ⅱ |
|  | OM451674 | Homo sapiens | China: Henan | 2013 | 13-China_Henan-62 | Ⅰ |
|  | MN510207 | Homo sapiens | China: Henan | 2013 | 13-China_Henan-65 | Ⅰ |
|  | OM451675 | Homo sapiens | China: Henan | 2013 | 13-China_Henan-67 | Ⅰ |
|  | OM451676 | Homo sapiens | China: Henan | 2013 | 13-China_Henan-68 | Ⅰ |
|  | OM451677 | Homo sapiens | China: Henan | 2013 | 13-China_Henan-77 | Ⅰ |
|  | OM451678 | Homo sapiens | China: Henan | 2013 | 13-China_Henan-78 | Ⅰ |
|  | OM451679 | Homo sapiens | China: Hubei | 2013 | 13-China_Hubei-79 | Ⅲ |
|  | OM451680 | Homo sapiens | China: Henan | 2013 | 13-China_Henan-86 | Ⅱ |
|  | OM451681 | Homo sapiens | China: Henan | 2013 | 13-China_Henan-87 | Ⅱ |
|  | OM451682 | Homo sapiens | China: Henan | 2013 | 13-China_Henan-91 | Ⅱ |
|  | OM451683 | Homo sapiens | China: Henan | 2013 | 13-China_Henan-93 | Ⅰ |
|  | MN510208 | Homo sapiens | China: Henan | 2013 | 13-China_Henan-94 | Ⅱ |
|  | OM451684 | Homo sapiens | China: Henan | 2014 | 14-China_Henan-107 | Ⅰ |
|  | OM451685 | Homo sapiens | China: Henan | 2014 | 14-China_Henan-109 | Ⅱ |
|  | OM451686 | Homo sapiens | China: Henan | 2014 | 14-China_Henan-11 | Ⅰ |
|  | OM451687 | Homo sapiens | China: Henan | 2014 | 14-China_Henan-118 | Ⅰ |
|  | OM451688 | Homo sapiens | China: Henan | 2014 | 14-China_Henan-129 | Ⅱ |
|  | OM451689 | Homo sapiens | China: Henan | 2014 | 14-China_Henan-13 | Ⅰ |
|  | OM451690 | Homo sapiens | China: Henan | 2014 | 14-China_Henan-134 | Ⅳ |
|  | OM451691 | Homo sapiens | China: Henan | 2014 | 14-China_Henan-143 | Ⅰ |
|  | OM451692 | Homo sapiens | China: Henan | 2014 | 14-China_Henan-148 | Ⅰ |
|  | OM451693 | Homo sapiens | China: Henan | 2014 | 14-China_Henan-149 | Ⅰ |
|  | OM451694 | Homo sapiens | China: Henan | 2014 | 14-China_Henan-15 | Ⅱ |
|  | MN510227 | Homo sapiens | China: Henan | 2014 | 14-China_Henan-151 | Ⅰ |
|  | OM451695 | Homo sapiens | China: Henan | 2014 | 14-China_Henan-152 | Ⅰ |
|  | OM451696 | Homo sapiens | China: Henan | 2014 | 14-China_Henan-172 | Ⅱ |
|  | OM451697 | Homo sapiens | China: Henan | 2014 | 14-China_Henan-174 | Ⅱ |
|  | OM451698 | Homo sapiens | China: Henan | 2014 | 14-China_Henan-175 | Ⅰ |
|  | OM451699 | Homo sapiens | China: Henan | 2014 | 14-China_Henan-177 | Ⅰ |
|  | OM451700 | Homo sapiens | China: Henan | 2014 | 14-China_Henan-178 | Ⅰ |
|  | OM451701 | Homo sapiens | China: Henan | 2014 | 14-China_Henan-189 | Ⅰ |
|  | MN510228 | Homo sapiens | China: Henan | 2014 | 14-China_Henan-191 | Ⅱ |
|  | OM451702 | Homo sapiens | China: Henan | 2014 | 14-China_Henan-193 | Ⅰ |
|  | OM451703 | Homo sapiens | China: Henan | 2014 | 14-China_Henan-20 | Ⅰ |
|  | OM451704 | Homo sapiens | China: Henan | 2014 | 14-China_Henan-22 | Ⅱ |
|  | MN510229 | Homo sapiens | China: Henan | 2014 | 14-China_Henan-222 | Ⅰ |
|  | MN510230 | Homo sapiens | China: Henan | 2014 | 14-China_Henan-236 | Ⅱ |
|  | OM451705 | Homo sapiens | China: Henan | 2014 | 14-China_Henan-26 | Ⅰ |
|  | OM451706 | Homo sapiens | China: Henan | 2014 | 14-China_Henan-3 | Ⅰ |
|  | OM451707 | Homo sapiens | China: Henan | 2014 | 14-China_Henan-307 | Ⅳ |
|  | OM451708 | Homo sapiens | China: Henan | 2014 | 14-China_Henan-311 | Ⅱ |
|  | OM451709 | Homo sapiens | China: Henan | 2014 | 14-China_Henan-312 | Ⅳ |
|  | MN510232 | Homo sapiens | China: Henan | 2014 | 14-China_Henan-314 | Ⅰ |
|  | OM451710 | Homo sapiens | China: Henan | 2014 | 14-China_Henan-316 | Ⅰ |
|  | OM451711 | Homo sapiens | China: Henan | 2014 | 14-China_Henan-320 | Ⅱ |
|  | OM451712 | Homo sapiens | China: Henan | 2014 | 14-China_Henan-326 | Ⅱ |
|  | MN510233 | Homo sapiens | China: Henan | 2014 | 14-China_Henan-337 | Ⅰ |
|  | OM451713 | Homo sapiens | China: Henan | 2014 | 14-China_Henan-346 | Ⅱ |
|  | OM451714 | Homo sapiens | China: Henan | 2014 | 14-China_Henan-35 | Ⅰ |
|  | OM451715 | Homo sapiens | China: Henan | 2014 | 14-China_Henan-403 | Ⅱ |
|  | OM451716 | Homo sapiens | China: Henan | 2014 | 14-China_Henan-422 | Ⅰ |
|  | OM451717 | Homo sapiens | China: Henan | 2014 | 14-China_Henan-424 | Ⅲ |
|  | OM451718 | Homo sapiens | China: Henan | 2014 | 14-China_Henan-436 | Ⅰ |
|  | OM451719 | Homo sapiens | China: Henan | 2014 | 14-China_Henan-438 | Ⅱ |
|  | OM451720 | Homo sapiens | China: Henan | 2014 | 14-China_Henan-440 | Ⅱ |
|  | OM451721 | Homo sapiens | China: Hubei | 2014 | 14-China_Hubei-444 | Ⅰ |
|  | OM451722 | Homo sapiens | China: Henan | 2014 | 14-China_Henan-446 | Ⅰ |
|  | OM451723 | Homo sapiens | China: Henan | 2014 | 14-China_Henan-452 | Ⅰ |
|  | OM451724 | Homo sapiens | China: Henan | 2014 | 14-China_Henan-453 | Ⅰ |
|  | OM451725 | Homo sapiens | China: Henan | 2014 | 14-China_Henan-463 | Ⅰ |
|  | OM451726 | Homo sapiens | China: Henan | 2014 | 14-China_Henan-474 | Ⅱ |
|  | OM451727 | Homo sapiens | China: Henan | 2014 | 14-China_Henan-484 | Ⅰ |
|  | OM451728 | Homo sapiens | China: Henan | 2014 | 14-China_Henan-485 | Ⅳ |
|  | OM451729 | Homo sapiens | China: Henan | 2014 | 14-China_Henan-488 | Ⅰ |
|  | OM451730 | Homo sapiens | China: Henan | 2014 | 14-China_Henan-49 | Ⅰ |
|  | MN510235 | Homo sapiens | China: Hubei | 2014 | 14-China_Hubei-492 | Ⅰ |
|  | OM451731 | Homo sapiens | China: Henan | 2014 | 14-China_Henan-494 | Ⅱ |
|  | OM451732 | Homo sapiens | China: Henan | 2014 | 14-China_Henan-499 | Ⅰ |
|  | OM451733 | Homo sapiens | China: Henan | 2014 | 14-China_Henan-5 | Ⅰ |
|  | OM451734 | Homo sapiens | China: Henan | 2014 | 14-China_Henan-501 | Ⅲ |
|  | OM451735 | Homo sapiens | China: Hubei | 2014 | 14-China_Hubei-512 | Ⅲ |
|  | MN510236 | Homo sapiens | China: Henan | 2014 | 14-China_Henan-523 | Ⅱ |
|  | OM451736 | Homo sapiens | China: Henan | 2014 | 14-China_Henan-529 | Ⅰ |
|  | OM451737 | Homo sapiens | China: Henan | 2014 | 14-China_Henan-54 | Ⅱ |
|  | OM451738 | Homo sapiens | China: Henan | 2014 | 14-China_Henan-540 | Ⅱ |
|  | OM451739 | Homo sapiens | China: Henan | 2014 | 14-China_Henan-542 | Ⅳ |
|  | OM451740 | Homo sapiens | China: Henan | 2014 | 14-China_Henan-546 | Ⅳ |
|  | OM451741 | Homo sapiens | China: Hubei | 2014 | 14-China_Hubei-548 | Ⅰ |
|  | OM451742 | Homo sapiens | China: Henan | 2014 | 14-China_Henan-55 | Ⅰ |
|  | OM451743 | Homo sapiens | China: Henan | 2014 | 14-China_Henan-555 | Ⅱ |
|  | OM451744 | Homo sapiens | China: Henan | 2014 | 14-China_Henan-559 | Ⅱ |
|  | OM451745 | Homo sapiens | China: Henan | 2014 | 14-China_Henan-561 | Ⅱ |
|  | OM451746 | Homo sapiens | China: Henan | 2014 | 14-China_Henan-562 | Ⅳ |
|  | OM451747 | Homo sapiens | China: Henan | 2014 | 14-China_Henan-563 | Ⅲ |
|  | OM451748 | Homo sapiens | China: Henan | 2014 | 14-China_Henan-564 | Ⅰ |
|  | OM451749 | Homo sapiens | China: Henan | 2014 | 14-China_Henan-567 | Ⅲ |
|  | OM451750 | Homo sapiens | China: Henan | 2014 | 14-China_Henan-568 | Ⅰ |
|  | OM451751 | Homo sapiens | China: Henan | 2014 | 14-China_Henan-570 | Ⅰ |
|  | OM451752 | Homo sapiens | China: Henan | 2014 | 14-China_Henan-571 | Ⅰ |
|  | OM451753 | Homo sapiens | China: Henan | 2014 | 14-China_Henan-573 | Ⅰ |
|  | OM451754 | Homo sapiens | China: Henan | 2014 | 14-China_Henan-574 | Ⅳ |
|  | OM451755 | Homo sapiens | China: Henan | 2014 | 14-China_Henan-578 | Ⅰ |
|  | MN510237 | Homo sapiens | China: Henan | 2014 | 14-China_Henan-579 | Ⅱ |
|  | OM451756 | Homo sapiens | China: Henan | 2014 | 14-China_Henan-58 | Ⅰ |
|  | OM451757 | Homo sapiens | China: Henan | 2014 | 14-China_Henan-581 | Ⅰ |
|  | OM451758 | Homo sapiens | China: Henan | 2014 | 14-China_Henan-582 | Ⅰ |
|  | OM451759 | Homo sapiens | China: Henan | 2014 | 14-China_Henan-586 | Ⅱ |
|  | OM451760 | Homo sapiens | China: Henan | 2014 | 14-China_Henan-592 | Ⅰ |
|  | OM451761 | Homo sapiens | China: Henan | 2014 | 14-China_Henan-593 | Ⅰ |
|  | OM451762 | Homo sapiens | China: Henan | 2014 | 14-China_Henan-600 | Ⅳ |
|  | OM451763 | Homo sapiens | China: Henan | 2014 | 14-China_Henan-601 | Ⅰ |
|  | OM451764 | Homo sapiens | China: Henan | 2014 | 14-China_Henan-604 | Ⅰ |
|  | OM451765 | Homo sapiens | China: Henan | 2014 | 14-China_Henan-605 | Ⅰ |
|  | OM451766 | Homo sapiens | China: Henan | 2014 | 14-China_Henan-606 | Ⅰ |
|  | MN510238 | Homo sapiens | China: Henan | 2014 | 14-China_Henan-607 | Ⅰ |
|  | OM451767 | Homo sapiens | China: Henan | 2014 | 14-China_Henan-61 | Ⅰ |
|  | OM451768 | Homo sapiens | China: Henan | 2014 | 14-China_Henan-610 | Ⅰ |
|  | MN510239 | Homo sapiens | China: Henan | 2014 | 14-China_Henan-613 | Ⅰ |
|  | OM451769 | Homo sapiens | China: Henan | 2014 | 14-China_Henan-617 | Ⅰ |
|  | OM451770 | Homo sapiens | China: Henan | 2014 | 14-China_Henan-62 | Ⅱ |
|  | OM451771 | Homo sapiens | China: Henan | 2014 | 14-China_Henan-63 | Ⅰ |
|  | OM451772 | Homo sapiens | China: Henan | 2014 | 14-China_Henan-66 | Ⅰ |
|  | OM451773 | Homo sapiens | China: Henan | 2014 | 14-China_Henan-68 | Ⅰ |
|  | OM451774 | Homo sapiens | China: Henan | 2014 | 14-China_Henan-69 | Ⅰ |
|  | OM451775 | Homo sapiens | China: Henan | 2014 | 14-China_Henan-70 | Ⅰ |
|  | OM451776 | Homo sapiens | China: Henan | 2014 | 14-China_Henan-71 | Ⅱ |
|  | OM451777 | Homo sapiens | China: Henan | 2014 | 14-China_Henan-74 | Ⅰ |
|  | OM451778 | Homo sapiens | China: Henan | 2014 | 14-China_Henan-76 | Ⅰ |
|  | MN510226 | Homo sapiens | China: Henan | 2014 | 14-China_Henan-79 | Ⅰ |
|  | OM451779 | Homo sapiens | China: Henan | 2014 | 14-China_Henan-84 | Ⅱ |
|  | OM451780 | Homo sapiens | China: Henan | 2014 | 14-China_Henan-86 | Ⅰ |
|  | OM451781 | Homo sapiens | China: Henan | 2014 | 14-China_Henan-9 | Ⅰ |
|  | OM451782 | Homo sapiens | China: Henan | 2016 | 16-China_Henan-110 | Ⅳ |
|  | OM451783 | Homo sapiens | China: Henan | 2015 | 15-China_Henan-113 | Ⅰ |
|  | OM451784 | Homo sapiens | China: Henan | 2015 | 15-China_Henan-117 | Ⅰ |
|  | OM451785 | Homo sapiens | China: Henan | 2015 | 15-China_Henan-123 | Ⅰ |
|  | OM451786 | Homo sapiens | China: Henan | 2015 | 15-China_Henan-127 | Ⅰ |
|  | OM451787 | Homo sapiens | China: Henan | 2015 | 15-China_Henan-130 | Ⅲ |
|  | OM451788 | Homo sapiens | China: Henan | 2015 | 15-China_Henan-132 | Ⅰ |
|  | OM451789 | Homo sapiens | China: Henan | 2015 | 15-China_Henan-134 | Ⅰ |
|  | OM451790 | Homo sapiens | China: Henan | 2016 | 16-China_Henan-136 | Ⅳ |
|  | OM451791 | Homo sapiens | China: Henan | 2015 | 15-China_Henan-141 | Ⅱ |
|  | OM451792 | Homo sapiens | China: Henan | 2015 | 15-China_Henan-147 | Ⅱ |
|  | OM451793 | Homo sapiens | China: Henan | 2015 | 15-China_Henan-15 | Ⅰ |
|  | MN510243 | Homo sapiens | China: Henan | 2015 | 15-China_Henan-150 | Ⅰ |
|  | OM451794 | Homo sapiens | China: Henan | 2015 | 15-China_Henan-151 | Ⅰ |
|  | OM451795 | Homo sapiens | China: Henan | 2015 | 15-China_Henan-153 | Ⅰ |
|  | MN510244 | Homo sapiens | China: Hubei | 2015 | 15-China_Hubei-154 | Ⅰ |
|  | OM451796 | Homo sapiens | China: Henan | 2015 | 15-China_Henan-155 | Ⅱ |
|  | OM451797 | Homo sapiens | China: Henan | 2015 | 15-China_Henan-16 | Ⅳ |
|  | OM451798 | Homo sapiens | China: Henan | 2015 | 15-China_Henan-160 | Ⅰ |
|  | OM451799 | Homo sapiens | China: Henan | 2015 | 15-China_Henan-165 | Ⅱ |
|  | OM451800 | Homo sapiens | China: Henan | 2015 | 15-China_Henan-168 | Ⅱ |
|  | OM451801 | Homo sapiens | China: Henan | 2015 | 15-China_Henan-170 | Ⅰ |
|  | OM451802 | Homo sapiens | China: Henan | 2015 | 15-China_Henan-174 | Ⅰ |
|  | OM451803 | Homo sapiens | China: Henan | 2015 | 15-China_Henan-176 | Ⅰ |
|  | OM451804 | Homo sapiens | China: Henan | 2015 | 15-China_Henan-178 | Ⅰ |
|  | OM451805 | Homo sapiens | China: Henan | 2015 | 15-China_Henan-179 | Ⅱ |
|  | OM451806 | Homo sapiens | China: Henan | 2015 | 15-China_Henan-2 | Ⅰ |
|  | OM451807 | Homo sapiens | China: Henan | 2015 | 15-China_Henan-200 | Ⅰ |
|  | OM451808 | Homo sapiens | China: Henan | 2015 | 15-China_Henan-201 | Ⅰ |
|  | OM451809 | Homo sapiens | China: Henan | 2015 | 15-China_Henan-204 | Ⅳ |
|  | OM451810 | Homo sapiens | China: Henan | 2015 | 15-China_Henan-206 | Ⅱ |
|  | OM451811 | Homo sapiens | China: Henan | 2015 | 15-China_Henan-211 | Ⅰ |
|  | OM451812 | Homo sapiens | China: Henan | 2015 | 15-China_Henan-213 | Ⅳ |
|  | OM451813 | Homo sapiens | China: Henan | 2015 | 15-China_Henan-217 | Ⅲ |
|  | OM451814 | Homo sapiens | China: Henan | 2015 | 15-China_Henan-221 | Ⅰ |
|  | OM451815 | Homo sapiens | China: Henan | 2015 | 15-China_Henan-224 | Ⅳ |
|  | OM451816 | Homo sapiens | China: Henan | 2015 | 15-China_Henan-225 | Ⅰ |
|  | OM451817 | Homo sapiens | China: Henan | 2015 | 15-China_Henan-226 | Ⅱ |
|  | OM451818 | Homo sapiens | China: Henan | 2015 | 15-China_Henan-227 | Ⅰ |
|  | OM451819 | Homo sapiens | China: Henan | 2015 | 15-China_Henan-228 | Ⅱ |
|  | OM451820 | Homo sapiens | China: Henan | 2015 | 15-China_Henan-229 | Ⅰ |
|  | OM451821 | Homo sapiens | China: Henan | 2015 | 15-China_Henan-23 | Ⅳ |
|  | OM451822 | Homo sapiens | China: Henan | 2015 | 15-China_Henan-232 | Ⅰ |
|  | OM451823 | Homo sapiens | China: Hubei | 2015 | 15-China_Hubei-234 | Ⅲ |
|  | OM451824 | Homo sapiens | China: Henan | 2015 | 15-China_Henan-238 | Ⅰ |
|  | OM451825 | Homo sapiens | China: Henan | 2015 | 15-China_Henan-249 | Ⅰ |
|  | OM451826 | Homo sapiens | China: Henan | 2015 | 15-China_Henan-250 | Ⅰ |
|  | MN510245 | Homo sapiens | China: Henan | 2015 | 15-China_Henan-256 | Ⅰ |
|  | OM451827 | Homo sapiens | China: Henan | 2015 | 15-China_Henan-257 | Ⅰ |
|  | OM451828 | Homo sapiens | China: Henan | 2015 | 15-China_Henan-258 | Ⅰ |
|  | MN510246 | Homo sapiens | China: Hubei | 2015 | 15-China_Hubei-259 | Ⅰ |
|  | OM451829 | Homo sapiens | China: Henan | 2015 | 15-China_Henan-260 | Ⅲ |
|  | OM451830 | Homo sapiens | China: Henan | 2015 | 15-China_Henan-266 | Ⅰ |
|  | OM451831 | Homo sapiens | China: Henan | 2015 | 15-China_Henan-29 | Ⅳ |
|  | OM451832 | Homo sapiens | China: Henan | 2015 | 15-China_Henan-30 | Ⅰ |
|  | OM451833 | Homo sapiens | China: Henan | 2015 | 15-China_Henan-316 | Ⅰ |
|  | MN510247 | Homo sapiens | China: Henan | 2015 | 15-China_Henan-321 | Ⅱ |
|  | OM451834 | Homo sapiens | China: Henan | 2015 | 15-China_Henan-322 | Ⅰ |
|  | OM451835 | Homo sapiens | China: Henan | 2015 | 15-China_Henan-323 | Ⅰ |
|  | OM451836 | Homo sapiens | China: Henan | 2015 | 15-China_Henan-326 | Ⅰ |
|  | OM451837 | Homo sapiens | China: Henan | 2015 | 15-China_Henan-338 | Ⅰ |
|  | MN510240 | Homo sapiens | China: Henan | 2015 | 15-China_Henan-34 | Ⅰ |
|  | OM451838 | Homo sapiens | China: Henan | 2015 | 15-China_Henan-343 | Ⅰ |
|  | MN510241 | Homo sapiens | China: Henan | 2015 | 15-China_Henan-35 | Ⅰ |
|  | MN510249 | Homo sapiens | China: Henan | 2015 | 15-China_Henan-351 | Ⅱ |
|  | OM451839 | Homo sapiens | China: Henan | 2015 | 15-China_Henan-353 | Ⅳ |
|  | MN510250 | Homo sapiens | China: Henan | 2015 | 15-China_Henan-356 | Ⅰ |
|  | OM451840 | Homo sapiens | China: Henan | 2015 | 15-China_Henan-363 | Ⅰ |
|  | OM451841 | Homo sapiens | China: Henan | 2016 | 16-China_Henan-364 | Ⅳ |
|  | OM451842 | Homo sapiens | China: Henan | 2015 | 15-China_Henan-365 | Ⅰ |
|  | OM451843 | Homo sapiens | China: Henan | 2015 | 15-China_Henan-374 | Ⅱ |
|  | MN510251 | Homo sapiens | China: Henan | 2015 | 15-China_Henan-380 | Ⅰ |
|  | OM451844 | Homo sapiens | China: Henan | 2015 | 15-China_Henan-391 | Ⅰ |
|  | OM451845 | Homo sapiens | China: Henan | 2015 | 15-China_Henan-393 | Ⅰ |
|  | OM451846 | Homo sapiens | China: Henan | 2015 | 15-China_Henan-398 | Ⅳ |
|  | OM451847 | Homo sapiens | China: Henan | 2015 | 15-China_Henan-4 | Ⅰ |
|  | OM451848 | Homo sapiens | China: Henan | 2015 | 15-China_Henan-40 | Ⅰ |
|  | MN510252 | Homo sapiens | China: Henan | 2015 | 15-China_Henan-411 | Ⅱ |
|  | OM451849 | Homo sapiens | China: Henan | 2015 | 15-China_Henan-413 | Ⅰ |
|  | OM451850 | Homo sapiens | China: Henan | 2015 | 15-China_Henan-414 | Ⅰ |
|  | OM451851 | Homo sapiens | China: Henan | 2015 | 15-China_Henan-415 | Ⅰ |
|  | OM451852 | Homo sapiens | China: Henan | 2015 | 15-China_Henan-55 | Ⅳ |
|  | OM451853 | Homo sapiens | China: Henan | 2015 | 15-China_Henan-58 | Ⅱ |
|  | OM451854 | Homo sapiens | China: Henan | 2015 | 15-China_Henan-61 | Ⅰ |
|  | OM451855 | Homo sapiens | China: Henan | 2015 | 15-China_Henan-62 | Ⅰ |
|  | OM451856 | Homo sapiens | China: Henan | 2015 | 15-China_Henan-68 | Ⅰ |
|  | OM451857 | Homo sapiens | China: Henan | 2015 | 15-China_Henan-7 | Ⅰ |
|  | OM451858 | Homo sapiens | China: Henan | 2015 | 15-China_Henan-73 | Ⅰ |
|  | OM451859 | Homo sapiens | China: Henan | 2015 | 15-China_Henan-75 | Ⅰ |
|  | OM451860 | Homo sapiens | China: Henan | 2015 | 15-China_Henan-78 | Ⅳ |
|  | OM451861 | Homo sapiens | China: Henan | 2015 | 15-China_Henan-79 | Ⅰ |
|  | OM451862 | Homo sapiens | China: Henan | 2015 | 15-China_Henan-8 | Ⅰ |
|  | OM451863 | Homo sapiens | China: Henan | 2015 | 15-China_Henan-82 | Ⅰ |
|  | OM451864 | Homo sapiens | China: Henan | 2015 | 15-China_Henan-9 | Ⅰ |
|  | OM451865 | Homo sapiens | China: Henan | 2015 | 15-China_Henan-91 | Ⅱ |
|  | OM451866 | Homo sapiens | China: Henan | 2015 | 15-China_Henan-95 | Ⅰ |
|  | OM451867 | Homo sapiens | China: Henan | 2016 | 16-China_Henan-115 | Ⅱ |
|  | OM451868 | Homo sapiens | China: Henan | 2016 | 16-China_Henan-118 | Ⅰ |
|  | OM451869 | Homo sapiens | China: Henan | 2016 | 16-China_Henan-122 | Ⅰ |
|  | OM451870 | Homo sapiens | China: Henan | 2016 | 16-China_Henan-123 | Ⅰ |
|  | OM451871 | Homo sapiens | China: Henan | 2016 | 16-China_Henan-128 | Ⅰ |
|  | MN510256 | Homo sapiens | China: Henan | 2016 | 16-China_Henan-135 | Ⅰ |
|  | MN510257 | Homo sapiens | China: Henan | 2016 | 16-China_Henan-138 | Ⅰ |
|  | MN510258 | Homo sapiens | China: Henan | 2016 | 16-China_Henan-149 | Ⅰ |
|  | OM451872 | Homo sapiens | China: Henan | 2016 | 16-China_Henan-16 | Ⅱ |
|  | OM451873 | Homo sapiens | China: Henan | 2016 | 16-China_Henan-164 | Ⅰ |
|  | MN510259 | Homo sapiens | China: Henan | 2016 | 16-China_Henan-167 | Ⅱ |
|  | MN510255 | Homo sapiens | China: Henan | 2016 | 16-China_Henan-17 | Ⅰ |
|  | MN510260 | Homo sapiens | China: Henan | 2016 | 16-China_Henan-176 | Ⅰ |
|  | MN510261 | Homo sapiens | China: Henan | 2016 | 16-China_Henan-179 | Ⅱ |
|  | OM451874 | Homo sapiens | China: Henan | 2016 | 16-China_Henan-18 | Ⅰ |
|  | OM451875 | Homo sapiens | China: Henan | 2016 | 16-China_Henan-181 | Ⅰ |
|  | OM451876 | Homo sapiens | China: Henan | 2016 | 16-China_Henan-187 | Ⅰ |
|  | OM451877 | Homo sapiens | China: Henan | 2016 | 16-China_Henan-188 | Ⅱ |
|  | OM451878 | Homo sapiens | China: Henan | 2016 | 16-China_Henan-19 | Ⅰ |
|  | MN510262 | Homo sapiens | China: Henan | 2016 | 16-China_Henan-196 | Ⅱ |
|  | OM451879 | Homo sapiens | China: Henan | 2016 | 16-China_Henan-198 | Ⅰ |
|  | MN510263 | Homo sapiens | China: Hubei | 2016 | 16-China_Hubei-203 | Ⅰ |
|  | OM451880 | Homo sapiens | China: Henan | 2016 | 16-China_Henan-212 | Ⅳ |
|  | MN510264 | Homo sapiens | China: Henan | 2016 | 16-China_Henan-245 | Ⅰ |
|  | OM451881 | Homo sapiens | China: Henan | 2016 | 16-China_Henan-251 | Ⅱ |
|  | OM451882 | Homo sapiens | China: Henan | 2016 | 16-China_Henan-279 | Ⅰ |
|  | OM451883 | Homo sapiens | China: Henan | 2016 | 16-China_Henan-283 | Ⅰ |
|  | OM451884 | Homo sapiens | China: Henan | 2016 | 16-China_Henan-286 | Ⅰ |
|  | OM451885 | Homo sapiens | China: Henan | 2016 | 16-China_Henan-29 | Ⅰ |
|  | MN510265 | Homo sapiens | China: Henan | 2016 | 16-China_Henan-305 | Ⅱ |
|  | OM451886 | Homo sapiens | China: Henan | 2016 | 16-China_Henan-32 | Ⅰ |
|  | MN510266 | Homo sapiens | China: Henan | 2016 | 16-China_Henan-335 | Ⅱ |
|  | MN510267 | Homo sapiens | China: Henan | 2016 | 16-China_Henan-341 | Ⅱ |
|  | OM451887 | Homo sapiens | China: Henan | 2016 | 16-China_Henan-370 | Ⅴ |
|  | OM451888 | Homo sapiens | China: Henan | 2016 | 16-China_Henan-39 | Ⅲ |
|  | MN510268 | Homo sapiens | China: Henan | 2016 | 16-China_Henan-397 | Ⅱ |
|  | OM451889 | Homo sapiens | China: Henan | 2016 | 16-China_Henan-401 | Ⅱ |
|  | MN510269 | Homo sapiens | China: Henan | 2016 | 16-China_Henan-402 | Ⅰ |
|  | OM451890 | Homo sapiens | China: Henan | 2016 | 16-China_Henan-405 | Ⅱ |
|  | MN510270 | Homo sapiens | China: Henan | 2016 | 16-China_Henan-412 | Ⅰ |
|  | MN510271 | Homo sapiens | China: Henan | 2016 | 16-China_Henan-418 | Ⅰ |
|  | OM451891 | Homo sapiens | China: Henan | 2016 | 16-China_Henan-421 | Ⅰ |
|  | OM451892 | Homo sapiens | China: Henan | 2016 | 16-China_Henan-428 | Ⅰ |
|  | MN510272 | Homo sapiens | China: Henan | 2016 | 16-China_Henan-431 | Ⅰ |
|  | OM451893 | Homo sapiens | China: Henan | 2016 | 16-China_Henan-439 | Ⅰ |
|  | MN510273 | Homo sapiens | China: Henan | 2016 | 16-China_Henan-444 | Ⅰ |
|  | MN510274 | Homo sapiens | China: Henan | 2016 | 16-China_Henan-452 | Ⅱ |
|  | OM451894 | Homo sapiens | China: Henan | 2016 | 16-China_Henan-453 | Ⅲ |
|  | MN510275 | Homo sapiens | China: Henan | 2016 | 16-China_Henan-456 | Ⅰ |
|  | OM451895 | Homo sapiens | China: Hubei | 2016 | 16-China_Hubei-471 | Ⅰ |
|  | MN510276 | Homo sapiens | China: Henan | 2016 | 16-China_Henan-504 | Ⅱ |
|  | OM451896 | Homo sapiens | China: Henan | 2016 | 16-China_Henan-532 | Ⅰ |
|  | MN510277 | Homo sapiens | China: Henan | 2016 | 16-China_Henan-590 | Ⅰ |
|  | MN510278 | Homo sapiens | China: Henan | 2016 | 16-China_Henan-595 | Ⅱ |
|  | OM451897 | Homo sapiens | China: Henan | 2016 | 16-China_Henan-598 | Ⅲ |
|  | OM451898 | Homo sapiens | China: Henan | 2016 | 16-China_Henan-605 | Ⅰ |
|  | OM451899 | Homo sapiens | China: Henan | 2016 | 16-China_Henan-614 | Ⅰ |
|  | OM451900 | Homo sapiens | China: Henan | 2016 | 16-China_Henan-64 | Ⅰ |
|  | OM451901 | Homo sapiens | China: Henan | 2016 | 16-China_Henan-74 | Ⅱ |
|  | OM451902 | Homo sapiens | China: Henan | 2016 | 16-China_Henan-81 | Ⅳ |
|  | MN510285 | Homo sapiens | China: Henan | 2017 | 17-China_Henan-110 | Ⅰ |
|  | MN510286 | Homo sapiens | China: Henan | 2017 | 17-China_Henan-112 | Ⅰ |
|  | MN510287 | Homo sapiens | China: Henan | 2017 | 17-China_Henan-116 | Ⅰ |
|  | MN510289 | Homo sapiens | China: Henan | 2017 | 17-China_Henan-138 | Ⅱ |
|  | MN510290 | Homo sapiens | China: Henan | 2017 | 17-China_Henan-142 | Ⅰ |
|  | MN510291 | Homo sapiens | China: Henan | 2017 | 17-China_Henan-143 | Ⅰ |
|  | MN510292 | Homo sapiens | China: Henan | 2017 | 17-China_Henan-154 | Ⅰ |
|  | MN510293 | Homo sapiens | China: Henan | 2017 | 17-China_Henan-178 | Ⅰ |
|  | MN510294 | Homo sapiens | China: Henan | 2017 | 17-China_Henan-215 | Ⅰ |
|  | OM451903 | Homo sapiens | China: Henan | 2017 | 17-China_Henan-218 | Ⅳ |
|  | MN510295 | Homo sapiens | China: Henan | 2017 | 17-China_Henan-242 | Ⅱ |
|  | MN510279 | Homo sapiens | China: Hubei | 2017 | 17-China_Hubei-25 | Ⅲ |
|  | OM451904 | Homo sapiens | China: Henan | 2017 | 17-China_Henan-257 | Ⅳ |
|  | MN510296 | Homo sapiens | China: Henan | 2017 | 17-China_Henan-290 | Ⅰ |
|  | MN510297 | Homo sapiens | China: Henan | 2017 | 17-China_Henan-317 | Ⅰ |
|  | MN510298 | Homo sapiens | China: Henan | 2017 | 17-China_Henan-320 | Ⅱ |
|  | MN510299 | Homo sapiens | China: Henan | 2017 | 17-China_Henan-337 | Ⅰ |
|  | MN510280 | Homo sapiens | China: Henan | 2017 | 17-China_Henan-35 | Ⅰ |
|  | MN510300 | Homo sapiens | China: Henan | 2017 | 17-China_Henan-356 | Ⅰ |
|  | MN510301 | Homo sapiens | China: Henan | 2017 | 17-China_Henan-365 | Ⅰ |
|  | MN510281 | Homo sapiens | China: Henan | 2017 | 17-China_Henan-51 | Ⅰ |
|  | MN510282 | Homo sapiens | China: Henan | 2017 | 17-China_Henan-64 | Ⅰ |
|  | OM451905 | Homo sapiens | China: Henan | 2017 | 17-China_Henan-76 | Ⅲ |
|  | OM451906 | Homo sapiens | China: Henan | 2017 | 17-China_Henan-84 | Ⅲ |
|  | MN510283 | Homo sapiens | China: Henan | 2017 | 17-China_Henan-91 | Ⅱ |
|  | MN510284 | Homo sapiens | China: Henan | 2017 | 17-China_Henan-94 | Ⅰ |
|  | OM451907 | Homo sapiens | China: Henan | 2017 | 17-China_Henan-96 | Ⅲ |
|  | OM451908 | Homo sapiens | China: Henan | 2017 | 17-China_Henan-101 | Ⅰ |
|  | OM451909 | Homo sapiens | China: Hubei | 2017 | 17-China_Hubei-102 | Ⅰ |
|  | OM451910 | Homo sapiens | China: Henan | 2018 | 18-China_Henan-103 | Ⅰ |
|  | OM451911 | Homo sapiens | China: Henan | 2018 | 18-China_Henan-105 | Ⅱ |
|  | OM451912 | Homo sapiens | China: Henan | 2017 | 17-China_Henan-107 | Ⅰ |
|  | MN510310 | Homo sapiens | China: Henan | 2018 | 18-China_Henan-109 | Ⅰ |
|  | OM451913 | Homo sapiens | China: Henan | 2018 | 18-China_Henan-110 | Ⅱ |
|  | OM451914 | Homo sapiens | China: Henan | 2017 | 17-China_Henan-114 | Ⅰ |
|  | OM451915 | Homo sapiens | China: Henan | 2017 | 17-China_Henan-113 | Ⅰ |
|  | OM451916 | Homo sapiens | China: Henan | 2018 | 18-China_Henan-114 | Ⅳ |
|  | OM451917 | Homo sapiens | China: Henan | 2018 | 18-China_Henan-115 | Ⅱ |
|  | OM451918 | Homo sapiens | China: Hubei | 2018 | 18-China_Hubei-118 | Ⅰ |
|  | OM451919 | Homo sapiens | China: Hubei | 2017 | 17-China_Hubei-120 | Ⅰ |
|  | OM451920 | Homo sapiens | China: Hubei | 2018 | 18-China_Hubei-123 | Ⅲ |
|  | OM451921 | Homo sapiens | China: Henan | 2017 | 17-China_Henan-128 | Ⅰ |
|  | OM451922 | Homo sapiens | China: Henan | 2018 | 18-China_Henan-129 | Ⅱ |
|  | OM451923 | Homo sapiens | China: Henan | 2017 | 17-China_Henan-13 | Ⅰ |
|  | OM451924 | Homo sapiens | China: Henan | 2018 | 18-China_Henan-130 | Ⅰ |
|  | OM451925 | Homo sapiens | China: Henan | 2017 | 17-China_Henan-136 | Ⅱ |
|  | OM451926 | Homo sapiens | China: Henan | 2017 | 17-China_Henan-139 | Ⅰ |
|  | OM451927 | Homo sapiens | China: Henan | 2018 | 18-China_Henan-139 | Ⅰ |
|  | OM451928 | Homo sapiens | China: Henan | 2018 | 18-China_Henan-141 | Ⅰ |
|  | OM451929 | Homo sapiens | China: Henan | 2018 | 18-China_Henan-143 | Ⅰ |
|  | OM451930 | Homo sapiens | China: Hubei | 2017 | 17-China_Hubei-144 | Ⅰ |
|  | OM451931 | Homo sapiens | China: Henan | 2017 | 17-China_Henan-147 | Ⅰ |
|  | OM451932 | Homo sapiens | China: Henan | 2017 | 17-China_Henan-148 | Ⅰ |
|  | OM451933 | Homo sapiens | China: Henan | 2018 | 18-China_Henan-149 | Ⅰ |
|  | OM451934 | Homo sapiens | China: Henan | 2018 | 18-China_Henan-150 | Ⅰ |
|  | MN510311 | Homo sapiens | China: Henan | 2018 | 18-China_Henan-152 | Ⅱ |
|  | OM451935 | Homo sapiens | China: Henan | 2018 | 18-China_Henan-153 | Ⅳ |
|  | OM451936 | Homo sapiens | China: Henan | 2018 | 18-China_Henan-154 | Ⅰ |
|  | OM451937 | Homo sapiens | China: Henan | 2018 | 18-China_Henan-155 | Ⅱ |
|  | OM451938 | Homo sapiens | China: Henan | 2018 | 18-China_Henan-158 | Ⅱ |
|  | OM451939 | Homo sapiens | China: Henan | 2018 | 18-China_Henan-159 | Ⅰ |
|  | OM451940 | Homo sapiens | China: Henan | 2018 | 18-China_Henan-160 | Ⅰ |
|  | OM451941 | Homo sapiens | China: Henan | 2018 | 18-China_Henan-161 | Ⅰ |
|  | OM451942 | Homo sapiens | China: Henan | 2017 | 17-China_Henan-163 | Ⅰ |
|  | OM451943 | Homo sapiens | China: Henan | 2018 | 18-China_Henan-164 | Ⅳ |
|  | OM451944 | Homo sapiens | China: Henan | 2017 | 17-China_Henan-165 | Ⅳ |
|  | OM451945 | Homo sapiens | China: Hubei | 2018 | 18-China_Hubei-167 | Ⅰ |
|  | OM451946 | Homo sapiens | China: Henan | 2018 | 18-China_Henan-169 | Ⅰ |
|  | OM451947 | Homo sapiens | China: Henan | 2018 | 18-China_Henan-170 | Ⅰ |
|  | OM451948 | Homo sapiens | China: Henan | 2018 | 18-China_Henan-172 | Ⅳ |
|  | OM451949 | Homo sapiens | China: Henan | 2017 | 17-China_Henan-177 | Ⅰ |
|  | OM451950 | Homo sapiens | China: Henan | 2018 | 18-China_Henan-178 | Ⅰ |
|  | OM451951 | Homo sapiens | China: Henan | 2018 | 18-China_Henan-180 | Ⅰ |
|  | MN510312 | Homo sapiens | China: Henan | 2018 | 18-China_Henan-184 | Ⅰ |
|  | OM451952 | Homo sapiens | China: Hubei | 2018 | 18-China_Hubei-186 | Ⅲ |
|  | OM451953 | Homo sapiens | China: Henan | 2018 | 18-China_Henan-187 | Ⅰ |
|  | MN510313 | Homo sapiens | China: Henan | 2018 | 18-China_Henan-189 | Ⅰ |
|  | OM451954 | Homo sapiens | China: Henan | 2017 | 17-China_Henan-190 | Ⅳ |
|  | OM451955 | Homo sapiens | China: Henan | 2018 | 18-China_Henan-191 | Ⅰ |
|  | OM451956 | Homo sapiens | China: Henan | 2018 | 18-China_Henan-193 | Ⅰ |
|  | OM451957 | Homo sapiens | China: Henan | 2017 | 17-China_Henan-194 | Ⅱ |
|  | OM451958 | Homo sapiens | China: Hubei | 2018 | 18-China_Hubei-199 | Ⅰ |
|  | OM451959 | Homo sapiens | China: Henan | 2018 | 18-China_Henan-2 | Ⅲ |
|  | MN510314 | Homo sapiens | China: Henan | 2018 | 18-China_Henan-200 | Ⅱ |
|  | OM451960 | Homo sapiens | China: Henan | 2018 | 18-China_Henan-207 | Ⅰ |
|  | OM451961 | Homo sapiens | China: Henan | 2018 | 18-China_Henan-208 | Ⅳ |
|  | OM451962 | Homo sapiens | China: Henan | 2018 | 18-China_Henan-209 | Ⅰ |
|  | OM451963 | Homo sapiens | China: Henan | 2018 | 18-China_Henan-210 | Ⅰ |
|  | MN510315 | Homo sapiens | China: Henan | 2018 | 18-China_Henan-213 | Ⅰ |
|  | OM451964 | Homo sapiens | China: Henan | 2018 | 18-China_Henan-217 | Ⅰ |
|  | OM451965 | Homo sapiens | China: Henan | 2018 | 18-China_Henan-22 | Ⅱ |
|  | OM451966 | Homo sapiens | China: Henan | 2018 | 18-China_Henan-222 | Ⅳ |
|  | OM451967 | Homo sapiens | China: Henan | 2018 | 18-China_Henan-223 | Ⅰ |
|  | OM451968 | Homo sapiens | China: Henan | 2018 | 18-China_Henan-225 | Ⅰ |
|  | MN510316 | Homo sapiens | China: Henan | 2018 | 18-China_Henan-226 | Ⅰ |
|  | OM451969 | Homo sapiens | China: Henan | 2018 | 18-China_Henan-227 | Ⅳ |
|  | OM451970 | Homo sapiens | China: Henan | 2018 | 18-China_Henan-228 | Ⅰ |
|  | OM451971 | Homo sapiens | China: Henan | 2018 | 18-China_Henan-233 | Ⅳ |
|  | OM451972 | Homo sapiens | China: Henan | 2017 | 17-China_Henan-241 | Ⅱ |
|  | OM451973 | Homo sapiens | China: Henan | 2018 | 18-China_Henan-243 | Ⅱ |
|  | OM451974 | Homo sapiens | China: Henan | 2018 | 18-China_Henan-251 | Ⅰ |
|  | OM451975 | Homo sapiens | China: Henan | 2018 | 18-China_Henan-254 | Ⅰ |
|  | OM451976 | Homo sapiens | China: Henan | 2017 | 17-China_Henan-255 | Ⅰ |
|  | OM451977 | Homo sapiens | China: Henan | 2018 | 18-China_Henan-257 | Ⅰ |
|  | OM451978 | Homo sapiens | China: Henan | 2018 | 18-China_Henan-258 | Ⅰ |
|  | OM451979 | Homo sapiens | China: Henan | 2017 | 17-China_Henan-261 | Ⅰ |
|  | OM451980 | Homo sapiens | China: Henan | 2018 | 18-China_Henan-263 | Ⅰ |
|  | OM451981 | Homo sapiens | China: Henan | 2017 | 17-China_Henan-264 | Ⅱ |
|  | OM451982 | Homo sapiens | China: Henan | 2018 | 18-China_Henan-268 | Ⅳ |
|  | OM451983 | Homo sapiens | China: Henan | 2017 | 17-China_Henan-269 | Ⅰ |
|  | OM451984 | Homo sapiens | China: Henan | 2018 | 18-China_Henan-27 | Ⅰ |
|  | OM451985 | Homo sapiens | China: Henan | 2018 | 18-China_Henan-271 | Ⅲ |
|  | OM451986 | Homo sapiens | China: Henan | 2018 | 18-China_Henan-273 | Ⅰ |
|  | MN510317 | Homo sapiens | China: Henan | 2018 | 18-China_Henan-278 | Ⅱ |
|  | MN510318 | Homo sapiens | China: Henan | 2018 | 18-China_Henan-279 | Ⅰ |
|  | OM451987 | Homo sapiens | China: Henan | 2018 | 18-China_Henan-28 | Ⅱ |
|  | OM451988 | Homo sapiens | China: Henan | 2017 | 17-China_Henan-281 | Ⅰ |
|  | OM451989 | Homo sapiens | China: Henan | 2017 | 17-China_Henan-285 | Ⅰ |
|  | OM451990 | Homo sapiens | China: Henan | 2017 | 17-China_Henan-286 | Ⅰ |
|  | MN510319 | Homo sapiens | China: Henan | 2018 | 18-China_Henan-287 | Ⅱ |
|  | OM451991 | Homo sapiens | China: Henan | 2017 | 17-China_Henan-289 | Ⅳ |
|  | OM451992 | Homo sapiens | China: Henan | 2018 | 18-China_Henan-291 | Ⅳ |
|  | OM451993 | Homo sapiens | China: Henan | 2018 | 18-China_Henan-292 | Ⅱ |
|  | OM451994 | Homo sapiens | China: Henan | 2017 | 17-China_Henan-293 | Ⅰ |
|  | OM451995 | Homo sapiens | China: Henan | 2017 | 17-China_Henan-299 | Ⅱ |
|  | OM451996 | Homo sapiens | China: Henan | 2017 | 17-China_Henan-304 | Ⅱ |
|  | OM451997 | Homo sapiens | China: Henan | 2018 | 18-China_Henan-309 | Ⅰ |
|  | OM451998 | Homo sapiens | China: Henan | 2018 | 18-China_Henan-312 | Ⅰ |
|  | OM451999 | Homo sapiens | China: Henan | 2017 | 17-China_Henan-322 | Ⅳ |
|  | OM452000 | Homo sapiens | China: Henan | 2017 | 17-China_Henan-321 | Ⅰ |
|  | MN510321 | Homo sapiens | China: Henan | 2018 | 18-China_Henan-323 | Ⅰ |
|  | OM452001 | Homo sapiens | China: Henan | 2017 | 17-China_Henan-325 | Ⅰ |
|  | OM452002 | Homo sapiens | China: Henan | 2018 | 18-China_Henan-326 | Ⅰ |
|  | OM452003 | Homo sapiens | China: Henan | 2017 | 17-China_Henan-33 | Ⅰ |
|  | MN510323 | Homo sapiens | China: Henan | 2018 | 18-China_Henan-334 | Ⅰ |
|  | OM452004 | Homo sapiens | China: Henan | 2018 | 18-China_Henan-337 | Ⅰ |
|  | OM452005 | Homo sapiens | China: Henan | 2018 | 18-China_Henan-338 | Ⅰ |
|  | OM452006 | Homo sapiens | China: Henan | 2018 | 18-China_Henan-342 | Ⅰ |
|  | OM452007 | Homo sapiens | China: Henan | 2017 | 17-China_Henan-343 | Ⅰ |
|  | OM452008 | Homo sapiens | China: Henan | 2018 | 18-China_Henan-348 | Ⅰ |
|  | OM452009 | Homo sapiens | China: Henan | 2018 | 18-China_Henan-349 | Ⅰ |
|  | OM452010 | Homo sapiens | China: Henan | 2017 | 17-China_Henan-36 | Ⅰ |
|  | OM452011 | Homo sapiens | China: Henan | 2018 | 18-China_Henan-353 | Ⅰ |
|  | OM452012 | Homo sapiens | China: Henan | 2017 | 17-China_Henan-354 | Ⅰ |
|  | OM452013 | Homo sapiens | China: Henan | 2018 | 18-China_Henan-356 | Ⅰ |
|  | OM452014 | Homo sapiens | China: Henan | 2018 | 18-China_Henan-357 | Ⅲ |
|  | OM452015 | Homo sapiens | China: Hubei | 2018 | 18-China_Hubei-358 | Ⅰ |
|  | OM452016 | Homo sapiens | China: Henan | 2018 | 18-China_Henan-362 | Ⅰ |
|  | OM452017 | Homo sapiens | China: Henan | 2018 | 18-China_Henan-365 | Ⅰ |
|  | MN510325 | Homo sapiens | China: Henan | 2018 | 18-China_Henan-366 | Ⅰ |
|  | OM452018 | Homo sapiens | China: Henan | 2017 | 17-China_Henan-367 | Ⅰ |
|  | OM452019 | Homo sapiens | China: Hubei | 2018 | 18-China_Hubei-368 | Ⅰ |
|  | OM452020 | Homo sapiens | China: Henan | 2018 | 18-China_Henan-37 | Ⅰ |
|  | OM452021 | Homo sapiens | China: Henan | 2018 | 18-China_Henan-40 | Ⅰ |
|  | OM452022 | Homo sapiens | China: Henan | 2017 | 17-China_Henan-42 | Ⅱ |
|  | OM452023 | Homo sapiens | China: Henan | 2018 | 18-China_Henan-43 | Ⅱ |
|  | OM452024 | Homo sapiens | China: Henan | 2018 | 18-China_Henan-44 | Ⅱ |
|  | OM452025 | Homo sapiens | China: Henan | 2017 | 17-China_Henan-47 | Ⅱ |
|  | MN510304 | Homo sapiens | China: Henan | 2018 | 18-China_Henan-50 | Ⅱ |
|  | OM452026 | Homo sapiens | China: Henan | 2018 | 18-China_Henan-51 | Ⅰ |
|  | OM452027 | Homo sapiens | China: Henan | 2018 | 18-China_Henan-53 | Ⅰ |
|  | OM452028 | Homo sapiens | China: Henan | 2017 | 17-China_Henan-54 | Ⅰ |
|  | OM452029 | Homo sapiens | China: Hubei | 2018 | 18-China_Hubei-58 | Ⅲ |
|  | OM452030 | Homo sapiens | China: Henan | 2018 | 18-China_Henan-6 | Ⅰ |
|  | MN510305 | Homo sapiens | China: Henan | 2018 | 18-China_Henan-60 | Ⅰ |
|  | OM452031 | Homo sapiens | China: Henan | 2018 | 18-China_Henan-61 | Ⅲ |
|  | MN510306 | Homo sapiens | China: Henan | 2018 | 18-China_Henan-64 | Ⅰ |
|  | OM452032 | Homo sapiens | China: Henan | 2018 | 18-China_Henan-65 | Ⅲ |
|  | OM452033 | Homo sapiens | China: Henan | 2017 | 17-China_Henan-67 | Ⅰ |
|  | MN510303 | Homo sapiens | China: Henan | 2018 | 18-China_Henan-7 | Ⅱ |
|  | OM452034 | Homo sapiens | China: Henan | 2018 | 18-China_Henan-70 | Ⅰ |
|  | OM452035 | Homo sapiens | China: Henan | 2018 | 18-China_Henan-71 | Ⅰ |
|  | OM452036 | Homo sapiens | China: Henan | 2017 | 17-China_Henan-72 | Ⅰ |
|  | OM452037 | Homo sapiens | China: Henan | 2017 | 17-China_Henan-79 | Ⅰ |
|  | OM452038 | Homo sapiens | China: Henan | 2018 | 18-China_Henan-80 | Ⅰ |
|  | OM452039 | Homo sapiens | China: Henan | 2018 | 18-China_Henan-81 | Ⅳ |
|  | OM452040 | Homo sapiens | China: Henan | 2018 | 18-China_Henan-84 | Ⅰ |
|  | OM452041 | Homo sapiens | China: Henan | 2017 | 17-China_Henan-87 | Ⅱ |
|  | OM452042 | Homo sapiens | China: Henan | 2018 | 18-China_Henan-89 | Ⅲ |
|  | MN510308 | Homo sapiens | China: Henan | 2018 | 18-China_Henan-93 | Ⅱ |
|  | MN510309 | Homo sapiens | China: Henan | 2018 | 18-China_Henan-94 | Ⅰ |
|  | OM452043 | Homo sapiens | China: Henan | 2017 | 17-China_Henan-98 | Ⅰ |
|  | OM452044 | Homo sapiens | China: Henan | 2017 | 17-China_Henan-99 | Ⅰ |
|  | OM452045 | Homo sapiens | China: Henan | 2019 | 19-China_Henan-12 | Ⅰ |
|  | OM452046 | Homo sapiens | China: Hubei | 2019 | 19-China_Hubei-144 | Ⅰ |
|  | OM452047 | Homo sapiens | China: Henan | 2019 | 19-China_Henan-162 | Ⅳ |
|  | OM452048 | Homo sapiens | China: Hubei | 2019 | 19-China_Hubei-167 | Ⅰ |
|  | OM452049 | Homo sapiens | China: Henan | 2019 | 19-China_Henan-186 | Ⅰ |
|  | OM452050 | Homo sapiens | China: Henan | 2019 | 19-China_Henan-19 | Ⅳ |
|  | OM452051 | Homo sapiens | China: Henan | 2019 | 19-China_Henan-193 | Ⅰ |
|  | OM452052 | Homo sapiens | China: Henan | 2019 | 19-China_Henan-231 | Ⅰ |
|  | OM452053 | Homo sapiens | China: Henan | 2019 | 19-China_Henan-233 | Ⅳ |
|  | OM452054 | Homo sapiens | China: Henan | 2019 | 19-China_Henan-234 | Ⅱ |
|  | OM452055 | Homo sapiens | China: Henan | 2019 | 19-China_Henan-237 | Ⅰ |
|  | OM452056 | Homo sapiens | China: Hubei | 2019 | 19-China_Hubei-238 | Ⅲ |
|  | OM452057 | Homo sapiens | China: Henan | 2019 | 19-China_Henan-248 | Ⅰ |
|  | OM452058 | Homo sapiens | China: Henan | 2019 | 19-China_Henan-271 | Ⅱ |
|  | OM452059 | Homo sapiens | China: Henan | 2019 | 19-China_Henan-287 | Ⅱ |
|  | OM452060 | Homo sapiens | China: Henan | 2019 | 19-China_Henan-3 | Ⅰ |
|  | OM452061 | Homo sapiens | China: Henan | 2019 | 19-China_Henan-309 | Ⅰ |
|  | OM452062 | Homo sapiens | China: Henan | 2019 | 19-China_Henan-313 | Ⅰ |
|  | OM452063 | Homo sapiens | China: Henan | 2019 | 19-China_Henan-316 | Ⅱ |
|  | OM452064 | Homo sapiens | China: Henan | 2019 | 19-China_Henan-317 | Ⅰ |
|  | OM452065 | Homo sapiens | China: Henan | 2019 | 19-China_Henan-330 | Ⅰ |
|  | OM452066 | Homo sapiens | China: Hubei | 2019 | 19-China_Hubei-7 | Ⅰ |
|  | OM452067 | Homo sapiens | China: Henan | 2019 | 19-China_Henan-94 | Ⅰ |
|  | OM452068 | Homo sapiens | China: Henan | 2020 | 20-China_Henan-1 | Ⅰ |
|  | OM452069 | Homo sapiens | China: Henan | 2020 | 20-China_Henan-100 | Ⅱ |
|  | OM452070 | Homo sapiens | China: Henan | 2020 | 20-China_Henan-102 | Ⅱ |
|  | OM452071 | Homo sapiens | China: Jiangxi | 2020 | 20-China_Jiangxi-104 | Ⅰ |
|  | OM452072 | Homo sapiens | China: Henan | 2020 | 20-China_Henan-108 | Ⅰ |
|  | OM452073 | Homo sapiens | China: Henan | 2020 | 20-China_Henan-111 | Ⅰ |
|  | OM452074 | Homo sapiens | China: Henan | 2020 | 20-China_Henan-112 | Ⅰ |
|  | OM452075 | Homo sapiens | China: Henan | 2020 | 20-China_Henan-113 | Ⅰ |
|  | OM452076 | Homo sapiens | China: Henan | 2020 | 20-China_Henan-114 | Ⅰ |
|  | OM452077 | Homo sapiens | China: Hubei | 2020 | 20-China_Hubei-116 | Ⅰ |
|  | OM452078 | Homo sapiens | China: Henan | 2020 | 20-China_Henan-118 | Ⅰ |
|  | OM452079 | Homo sapiens | China: Henan | 2020 | 20-China_Henan-120 | Ⅰ |
|  | OM452080 | Homo sapiens | China: Henan | 2020 | 20-China_Henan-122 | Ⅱ |
|  | OM452081 | Homo sapiens | China: Henan | 2020 | 20-China_Henan-126 | Ⅰ |
|  | OM452082 | Homo sapiens | China: Henan | 2020 | 20-China_Henan-13 | Ⅰ |
|  | OM452083 | Homo sapiens | China: Henan | 2020 | 20-China_Henan-130 | Ⅰ |
|  | OM452084 | Homo sapiens | China: Henan | 2020 | 20-China_Henan-133 | Ⅰ |
|  | OM452085 | Homo sapiens | China: Henan | 2020 | 20-China_Henan-134 | Ⅰ |
|  | OM452086 | Homo sapiens | China: Henan | 2020 | 20-China_Henan-137 | Ⅰ |
|  | OM452087 | Homo sapiens | China: Henan | 2020 | 20-China_Henan-138 | Ⅰ |
|  | OM452088 | Homo sapiens | China: Henan | 2020 | 20-China_Henan-14 | Ⅵ |
|  | OM452089 | Homo sapiens | China: Henan | 2020 | 20-China_Henan-140 | Ⅳ |
|  | OM452090 | Homo sapiens | China: Henan | 2020 | 20-China_Henan-142 | Ⅱ |
|  | OM452091 | Homo sapiens | China: Hubei | 2020 | 20-China_Hubei-143 | Ⅰ |
|  | OM452092 | Homo sapiens | China: Henan | 2020 | 20-China_Henan-144 | Ⅱ |
|  | OM452093 | Homo sapiens | China: Henan | 2020 | 20-China_Henan-145 | Ⅱ |
|  | OM452094 | Homo sapiens | China: Henan | 2020 | 20-China_Henan-149 | Ⅳ |
|  | OM452095 | Homo sapiens | China: Henan | 2020 | 20-China_Henan-15 | Ⅰ |
|  | OM452096 | Homo sapiens | China: Henan | 2020 | 20-China_Henan-153 | Ⅰ |
|  | OM452097 | Homo sapiens | China: Henan | 2020 | 20-China_Henan-156 | Ⅱ |
|  | OM452098 | Homo sapiens | China: Henan | 2020 | 20-China_Henan-157 | Ⅰ |
|  | OM452099 | Homo sapiens | China: Henan | 2020 | 20-China_Henan-158 | Ⅱ |
|  | OM452100 | Homo sapiens | China: Henan | 2020 | 20-China_Henan-168 | Ⅰ |
|  | OM452101 | Homo sapiens | China: Henan | 2020 | 20-China_Henan-175 | Ⅰ |
|  | OM452102 | Homo sapiens | China: Henan | 2020 | 20-China_Henan-177 | Ⅱ |
|  | OM452103 | Homo sapiens | China: Henan | 2020 | 20-China_Henan-178 | Ⅱ |
|  | OM452104 | Homo sapiens | China: Henan | 2020 | 20-China_Henan-180 | Ⅱ |
|  | OM452105 | Homo sapiens | China: Henan | 2020 | 20-China_Henan-181 | Ⅰ |
|  | OM452106 | Homo sapiens | China: Henan | 2020 | 20-China_Henan-182 | Ⅰ |
|  | OM452107 | Homo sapiens | China: Henan | 2020 | 20-China_Henan-183 | Ⅰ |
|  | OM452108 | Homo sapiens | China: Henan | 2020 | 20-China_Henan-189 | Ⅰ |
|  | OM452109 | Homo sapiens | China: Henan | 2020 | 20-China_Henan-190 | Ⅱ |
|  | OM452110 | Homo sapiens | China: Hubei | 2020 | 20-China_Hubei-191 | Ⅲ |
|  | OM452111 | Homo sapiens | China: Henan | 2020 | 20-China_Henan-192 | Ⅰ |
|  | OM452112 | Homo sapiens | China: Henan | 2020 | 20-China_Henan-198 | Ⅰ |
|  | OM452113 | Homo sapiens | China: Henan | 2020 | 20-China_Henan-20 | Ⅱ |
|  | OM452114 | Homo sapiens | China: Henan | 2020 | 20-China_Henan-200 | Ⅰ |
|  | OM452115 | Homo sapiens | China: Hubei | 2020 | 20-China_Hubei-201 | Ⅰ |
|  | OM452116 | Homo sapiens | China: Henan | 2020 | 20-China_Henan-203 | Ⅱ |
|  | OM452117 | Homo sapiens | China: Henan | 2020 | 20-China_Henan-209 | Ⅰ |
|  | OM452118 | Homo sapiens | China: Henan | 2020 | 20-China_Henan-210 | Ⅱ |
|  | OM452119 | Homo sapiens | China: Henan | 2020 | 20-China_Henan-211 | Ⅰ |
|  | OM452120 | Homo sapiens | China: Henan | 2020 | 20-China_Henan-215 | Ⅳ |
|  | OM452121 | Homo sapiens | China: Henan | 2020 | 20-China_Henan-219 | Ⅰ |
|  | OM452122 | Homo sapiens | China: Henan | 2020 | 20-China_Henan-222 | Ⅰ |
|  | OM452123 | Homo sapiens | China: Henan | 2020 | 20-China_Henan-223 | Ⅱ |
|  | OM452124 | Homo sapiens | China: Hubei | 2020 | 20-China_Hubei-228 | Ⅰ |
|  | OM452125 | Homo sapiens | China: Henan | 2020 | 20-China_Henan-230 | Ⅱ |
|  | OM452126 | Homo sapiens | China: Hubei | 2020 | 20-China_Hubei-233 | Ⅰ |
|  | OM452127 | Homo sapiens | China: Henan | 2020 | 20-China_Henan-234 | Ⅰ |
|  | OM452128 | Homo sapiens | China: Henan | 2020 | 20-China_Henan-236 | Ⅰ |
|  | OM452129 | Homo sapiens | China: Henan | 2020 | 20-China_Henan-238 | Ⅱ |
|  | OM452130 | Homo sapiens | China: Henan | 2020 | 20-China_Henan-24 | Ⅰ |
|  | OM452131 | Homo sapiens | China: Hubei | 2020 | 20-China_Hubei-240 | Ⅰ |
|  | OM452132 | Homo sapiens | China: Henan | 2020 | 20-China_Henan-244 | Ⅰ |
|  | OM452133 | Homo sapiens | China: Henan | 2020 | 20-China_Henan-246 | Ⅰ |
|  | OM452134 | Homo sapiens | China: Henan | 2020 | 20-China_Henan-249 | Ⅰ |
|  | OM452135 | Homo sapiens | China: Henan | 2020 | 20-China_Henan-252 | Ⅱ |
|  | OM452136 | Homo sapiens | China: Henan | 2020 | 20-China_Henan-254 | Ⅲ |
|  | OM452137 | Homo sapiens | China: Henan | 2020 | 20-China_Henan-255 | Ⅲ |
|  | OM452138 | Homo sapiens | China: Henan | 2020 | 20-China_Henan-28 | Ⅳ |
|  | OM452139 | Homo sapiens | China: Henan | 2020 | 20-China_Henan-3 | Ⅰ |
|  | OM452140 | Homo sapiens | China: Henan | 2020 | 20-China_Henan-37 | Ⅰ |
|  | OM452141 | Homo sapiens | China: Henan | 2020 | 20-China_Henan-4 | Ⅰ |
|  | OM452142 | Homo sapiens | China: Henan | 2020 | 20-China_Henan-40 | Ⅰ |
|  | OM452143 | Homo sapiens | China: Henan | 2020 | 20-China_Henan-41 | Ⅰ |
|  | OM452144 | Homo sapiens | China: Henan | 2020 | 20-China_Henan-42 | Ⅰ |
|  | OM452145 | Homo sapiens | China: Henan | 2020 | 20-China_Henan-43 | Ⅰ |
|  | OM452146 | Homo sapiens | China: Henan | 2020 | 20-China_Henan-48 | Ⅰ |
|  | OM452147 | Homo sapiens | China: Henan | 2020 | 20-China_Henan-49 | Ⅰ |
|  | OM452148 | Homo sapiens | China: Henan | 2020 | 20-China_Henan-51 | Ⅰ |
|  | OM452149 | Homo sapiens | China: Hubei | 2020 | 20-China_Hubei-54 | Ⅰ |
|  | OM452150 | Homo sapiens | China: Henan | 2020 | 20-China_Henan-59 | Ⅰ |
|  | OM452151 | Homo sapiens | China: Henan | 2020 | 20-China_Henan-61 | Ⅳ |
|  | OM452152 | Homo sapiens | China: Henan | 2020 | 20-China_Henan-65 | Ⅰ |
|  | OM452153 | Homo sapiens | China: Henan | 2020 | 20-China_Henan-67 | Ⅰ |
|  | OM452154 | Homo sapiens | China: Henan | 2020 | 20-China_Henan-73 | Ⅰ |
|  | OM452155 | Homo sapiens | China: Henan | 2020 | 20-China_Henan-75 | Ⅳ |
|  | OM452156 | Homo sapiens | China: Henan | 2020 | 20-China_Henan-77 | Ⅰ |
|  | OM452157 | Homo sapiens | China: Henan | 2020 | 20-China_Henan-78 | Ⅰ |
|  | OM452158 | Homo sapiens | China: Henan | 2020 | 20-China_Henan-81 | Ⅱ |
|  | OM452159 | Homo sapiens | China: Henan | 2020 | 20-China_Henan-86 | Ⅰ |
|  | OM452160 | Homo sapiens | China: Henan | 2020 | 20-China_Henan-88 | Ⅰ |
|  | OM452161 | Homo sapiens | China: Henan | 2020 | 20-China_Henan-90 | Ⅰ |
|  | OM452162 | Homo sapiens | China: Hubei | 2020 | 20-China_Hubei-92 | Ⅰ |
|  | OM452163 | Homo sapiens | China: Henan | 2020 | 20-China_Henan-93 | Ⅰ |
|  | OM452164 | Homo sapiens | China: Henan | 2020 | 20-China_Henan-99 | Ⅱ |
|  | OM452165 | Homo sapiens | China: Henan | 2019 | 19-China_Henan-126 | Ⅳ |
|  | OM452166 | Homo sapiens | China: Henan | 2019 | 19-China_Henan-129 | Ⅱ |
|  | OM452167 | Homo sapiens | China: Henan | 2019 | 19-China_Henan-132 | Ⅰ |
|  | OM452168 | Homo sapiens | China: Henan | 2019 | 19-China_Henan-133 | Ⅰ |
|  | OM452169 | Homo sapiens | China: Henan | 2019 | 19-China_Henan-137 | Ⅰ |
|  | OM452170 | Homo sapiens | China: Henan | 2019 | 19-China_Henan-139 | Ⅰ |
|  | OM452171 | Homo sapiens | China: Henan | 2019 | 19-China_Henan-148 | Ⅰ |
|  | OM452172 | Homo sapiens | China: Henan | 2019 | 19-China_Henan-156 | Ⅱ |
|  | OM452173 | Homo sapiens | China: Henan | 2019 | 19-China_Henan-160 | Ⅰ |
|  | OM452174 | Homo sapiens | China: Hubei | 2019 | 19-China_Hubei-166 | Ⅲ |
|  | OM452175 | Homo sapiens | China: Henan | 2019 | 19-China_Henan-173 | Ⅱ |
|  | OM452176 | Homo sapiens | China: Hubei | 2019 | 19-China_Hubei-174 | Ⅰ |
|  | OM452177 | Homo sapiens | China: Henan | 2019 | 19-China_Henan-175 | Ⅰ |
|  | OM452178 | Homo sapiens | China: Henan | 2019 | 19-China_Henan-177 | Ⅰ |
|  | OM452179 | Homo sapiens | China: Henan | 2019 | 19-China_Henan-179 | Ⅱ |
|  | OM452180 | Homo sapiens | China: Henan | 2019 | 19-China_Henan-183 | Ⅰ |
|  | OM452181 | Homo sapiens | China: Hubei | 2019 | 19-China_Hubei-185 | Ⅰ |
|  | OM452182 | Homo sapiens | China: Henan | 2019 | 19-China_Henan-189 | Ⅰ |
|  | OM452183 | Homo sapiens | China: Hubei | 2019 | 19-China_Hubei-195 | Ⅰ |
|  | OM452184 | Homo sapiens | China: Henan | 2019 | 19-China_Henan-197 | Ⅰ |
|  | OM452185 | Homo sapiens | China: Henan | 2019 | 19-China_Henan-198 | Ⅰ |
|  | OM452186 | Homo sapiens | China: Henan | 2019 | 19-China_Henan-199 | Ⅰ |
|  | OM452187 | Homo sapiens | China: Henan | 2019 | 19-China_Henan-201 | Ⅰ |
|  | OM452188 | Homo sapiens | China: Henan | 2019 | 19-China_Henan-208 | Ⅰ |
|  | OM452189 | Homo sapiens | China: Henan | 2019 | 19-China_Henan-214 | Ⅰ |
|  | OM452190 | Homo sapiens | China: Henan | 2019 | 19-China_Henan-215 | Ⅱ |
|  | OM452191 | Homo sapiens | China: Henan | 2019 | 19-China_Henan-217 | Ⅰ |
|  | OM452192 | Homo sapiens | China: Henan | 2019 | 19-China_Henan-223 | Ⅱ |
|  | OM452193 | Homo sapiens | China: Henan | 2019 | 19-China_Henan-227 | Ⅱ |
|  | OM452194 | Homo sapiens | China: Henan | 2019 | 19-China_Henan-232 | Ⅰ |
|  | OM452195 | Homo sapiens | China: Henan | 2019 | 19-China_Henan-235 | Ⅰ |
|  | OM452196 | Homo sapiens | China: Henan | 2019 | 19-China_Henan-236 | Ⅰ |
|  | OM452197 | Homo sapiens | China: Hubei | 2019 | 19-China_Hubei-240 | Ⅰ |
|  | OM452198 | Homo sapiens | China: Henan | 2019 | 19-China_Henan-241 | Ⅰ |
|  | OM452199 | Homo sapiens | China: Henan | 2019 | 19-China_Henan-242 | Ⅰ |
|  | OM452200 | Homo sapiens | China: Henan | 2019 | 19-China_Henan-243 | Ⅰ |
|  | OM452201 | Homo sapiens | China: Hubei | 2019 | 19-China_Hubei-249 | Ⅰ |
|  | OM452202 | Homo sapiens | China: Henan | 2019 | 19-China_Henan-253 | Ⅰ |
|  | OM452203 | Homo sapiens | China: Henan | 2019 | 19-China_Henan-254 | Ⅰ |
|  | OM452204 | Homo sapiens | China: Henan | 2019 | 19-China_Henan-259 | Ⅰ |
|  | OM452205 | Homo sapiens | China: Henan | 2019 | 19-China_Henan-260 | Ⅰ |
|  | OM452206 | Homo sapiens | China: Henan | 2019 | 19-China_Henan-261 | Ⅰ |
|  | OM452207 | Homo sapiens | China: Henan | 2019 | 19-China_Henan-262 | Ⅰ |
|  | OM452208 | Homo sapiens | China: Hubei | 2019 | 19-China_Hubei-263 | Ⅰ |
|  | OM452209 | Homo sapiens | China: Henan | 2019 | 19-China_Henan-264 | Ⅰ |
|  | OM452210 | Homo sapiens | China: Henan | 2019 | 19-China_Henan-266 | Ⅰ |
|  | OM452211 | Homo sapiens | China: Henan | 2019 | 19-China_Henan-268 | Ⅰ |
|  | OM452212 | Homo sapiens | China: Henan | 2019 | 19-China_Henan-272 | Ⅰ |
|  | OM452213 | Homo sapiens | China: Henan | 2019 | 19-China_Henan-273 | Ⅰ |
|  | OM452214 | Homo sapiens | China: Henan | 2019 | 19-China_Henan-274 | Ⅳ |
|  | OM452215 | Homo sapiens | China: Henan | 2019 | 19-China_Henan-278 | Ⅰ |
|  | OM452216 | Homo sapiens | China: Hubei | 2019 | 19-China_Hubei-279 | Ⅰ |
|  | OM452217 | Homo sapiens | China: Henan | 2019 | 19-China_Henan-280 | Ⅱ |
|  | OM452218 | Homo sapiens | China: Henan | 2019 | 19-China_Henan-285 | Ⅰ |
|  | OM452219 | Homo sapiens | China: Hubei | 2019 | 19-China_Hubei-291 | Ⅲ |
|  | OM452220 | Homo sapiens | China: Henan | 2019 | 19-China_Henan-295 | Ⅳ |
|  | OM452221 | Homo sapiens | China: Hubei | 2019 | 19-China_Hubei-297 | Ⅰ |
|  | OM452222 | Homo sapiens | China: Henan | 2019 | 19-China_Henan-298 | Ⅰ |
|  | OM452223 | Homo sapiens | China: Hubei | 2019 | 19-China_Hubei-299 | Ⅰ |
|  | OM452224 | Homo sapiens | China: Henan | 2019 | 19-China_Henan-301 | Ⅰ |
|  | OM452225 | Homo sapiens | China: Henan | 2019 | 19-China_Henan-302 | Ⅰ |
|  | OM452226 | Homo sapiens | China: Henan | 2019 | 19-China_Henan-304 | Ⅰ |
|  | OM452227 | Homo sapiens | China: Henan | 2019 | 19-China_Henan-305 | Ⅰ |
|  | OM452228 | Homo sapiens | China: Hubei | 2019 | 19-China_Hubei-306 | Ⅰ |
|  | OM452229 | Homo sapiens | China: Henan | 2019 | 19-China_Henan-308 | Ⅱ |
|  | OM452230 | Homo sapiens | China: Hubei | 2019 | 19-China_Hubei-310 | Ⅰ |
|  | OM452231 | Homo sapiens | China: Hubei | 2019 | 19-China_Hubei-314 | Ⅰ |
|  | OM452232 | Homo sapiens | China: Henan | 2019 | 19-China_Henan-321 | Ⅱ |
|  | OM452233 | Homo sapiens | China: Henan | 2019 | 19-China_Henan-322 | Ⅰ |
|  | OM452234 | Homo sapiens | China: Henan | 2019 | 19-China_Henan-323 | Ⅰ |
|  | OM452235 | Homo sapiens | China: Henan | 2019 | 19-China_Henan-324 | Ⅳ |
|  | OM452236 | Homo sapiens | China: Hubei | 2019 | 19-China_Hubei-327 | Ⅲ |
|  | OM452237 | Homo sapiens | China: Henan | 2019 | 19-China_Henan-358 | Ⅰ |
|  | OM452238 | Homo sapiens | China: Hubei | 2019 | 19-China_Hubei-377 | Ⅰ |
|  | OM452239 | Homo sapiens | China: Henan | 2019 | 19-China_Henan-382 | Ⅳ |
|  | OM452240 | Homo sapiens | China: Henan | 2019 | 19-China_Henan-388 | Ⅰ |
|  | OM452241 | Homo sapiens | China: Henan | 2020 | 20-China_Henan-12 | Ⅳ |
|  | OM452242 | Homo sapiens | China: Henan | 2020 | 20-China_Henan-115 | Ⅳ |
|  | OM452243 | Homo sapiens | China: Henan | 2020 | 20-China_Henan-139 | Ⅳ |
| M | OM452244 | Homo sapiens | China: Henan | 2011 | 11-China_Henan-1 | Ⅰ |
|  | MN510019 | Homo sapiens | China: Henan | 2011 | 11-China_Henan-13 | Ⅰ |
|  | OM452245 | Homo sapiens | China: Henan | 2011 | 11-China_Henan-2 | Ⅳ |
|  | OM452246 | Homo sapiens | China: Henan | 2011 | 11-China_Henan-266 | Ⅱ |
|  | MN510023 | Homo sapiens | China: Henan | 2011 | 11-China_Henan-273 | Ⅰ |
|  | MN510026 | Homo sapiens | China: Henan | 2012 | 12-China_Henan-103 | Ⅰ |
|  | OM452247 | Homo sapiens | China: Henan | 2012 | 12-China_Henan-108 | Ⅱ |
|  | MN510027 | Homo sapiens | China: Henan | 2012 | 12-China_Henan-109 | Ⅳ |
|  | MN510028 | Homo sapiens | China: Henan | 2012 | 12-China_Henan-110 | Ⅰ |
|  | MN510029 | Homo sapiens | China: Henan | 2012 | 12-China_Henan-114 | Ⅳ |
|  | OM452248 | Homo sapiens | China: Henan | 2012 | 12-China_Henan-127 | Ⅱ |
|  | MN510030 | Homo sapiens | China: Henan | 2012 | 12-China_Henan-13 | Ⅰ |
|  | MN510025 | Homo sapiens | China: Anhui | 2012 | 12-China_Anhui-154 | Ⅰ |
|  | MN510034 | Homo sapiens | China: Henan | 2012 | 12-China_Henan-194 | Ⅰ |
|  | OM452249 | Homo sapiens | China: Hubei | 2012 | 12-China_Hubei-21 | Ⅱ |
|  | OM452250 | Homo sapiens | China: Henan | 2012 | 12-China_Henan-220 | Ⅰ |
|  | OM452251 | Homo sapiens | China: Henan | 2012 | 12-China_Henan-224 | Ⅰ |
|  | MN510035 | Homo sapiens | China: Henan | 2012 | 12-China_Henan-23 | Ⅰ |
|  | MN510036 | Homo sapiens | China: Henan | 2012 | 12-China_Henan-234 | Ⅰ |
|  | OM452252 | Homo sapiens | China: Henan | 2012 | 12-China_Henan-237 | Ⅰ |
|  | OM452253 | Homo sapiens | China: Hebei | 2012 | 12-China_Hebei-24 | Ⅰ |
|  | MN510051 | Homo sapiens | China: Hubei | 2012 | 12-China_Hubei-242 | Ⅰ |
|  | MN510038 | Homo sapiens | China: Henan | 2012 | 12-China_Henan-270 | Ⅰ |
|  | OM452254 | Homo sapiens | China: Henan | 2012 | 12-China_Henan-271 | Ⅰ |
|  | OM452255 | Homo sapiens | China: Henan | 2012 | 12-China_Henan-279 | Ⅱ |
|  | OM452256 | Homo sapiens | China: Henan | 2012 | 12-China_Henan-30 | Ⅱ |
|  | OM452257 | Homo sapiens | China: Henan | 2012 | 12-China_Henan-32 | Ⅰ |
|  | OM452258 | Homo sapiens | China: Henan | 2012 | 12-China_Henan-324 | Ⅱ |
|  | MN510040 | Homo sapiens | China: Henan | 2012 | 12-China_Henan-36 | Ⅰ |
|  | MN510041 | Homo sapiens | China: Henan | 2012 | 12-China_Henan-37 | Ⅳ |
|  | OM452259 | Homo sapiens | China: Henan | 2012 | 12-China_Henan-40 | Ⅳ |
|  | OM452260 | Homo sapiens | China: Henan | 2012 | 12-China_Henan-41 | Ⅰ |
|  | MN510043 | Homo sapiens | China: Henan | 2012 | 12-China_Henan-48 | Ⅰ |
|  | OM452261 | Homo sapiens | China: Henan | 2012 | 12-China_Henan-5 | Ⅱ |
|  | OM452262 | Homo sapiens | China: Henan | 2012 | 12-China_Henan-54 | Ⅰ |
|  | OM452263 | Homo sapiens | China: Henan | 2012 | 12-China_Henan-56 | Ⅱ |
|  | OM452264 | Homo sapiens | China: Henan | 2012 | 12-China_Henan-64 | Ⅰ |
|  | OM452265 | Homo sapiens | China: Henan | 2012 | 12-China_Henan-65 | Ⅰ |
|  | OM452266 | Homo sapiens | China: Henan | 2012 | 12-China_Henan-7 | Ⅰ |
|  | MN510050 | Homo sapiens | China: Henan | 2012 | 12-China_Henan-9 | Ⅰ |
|  | OM452267 | Homo sapiens | China: Henan | 2012 | 12-China_Henan-90 | Ⅶ |
|  | MN510053 | Homo sapiens | China: Henan | 2013 | 13-China_Henan-100 | Ⅲ |
|  | OM452268 | Homo sapiens | China: Henan | 2013 | 13-China_Henan-101 | Ⅰ |
|  | MN510054 | Homo sapiens | China: Henan | 2013 | 13-China_Henan-110 | Ⅰ |
|  | OM452269 | Homo sapiens | China: Henan | 2013 | 13-China_Henan-112 | Ⅱ |
|  | MN510069 | Homo sapiens | China: Hubei | 2013 | 13-China_Hubei-113 | Ⅰ |
|  | OM452270 | Homo sapiens | China: Henan | 2013 | 13-China_Henan-116 | Ⅰ |
|  | OM452271 | Homo sapiens | China: Henan | 2013 | 13-China_Henan-118 | Ⅰ |
|  | OM452272 | Homo sapiens | China: Henan | 2013 | 13-China_Henan-119 | Ⅰ |
|  | OM452273 | Homo sapiens | China: Henan | 2013 | 13-China_Henan-120 | Ⅰ |
|  | OM452274 | Homo sapiens | China: Henan | 2013 | 13-China_Henan-122 | Ⅱ |
|  | MN510070 | Homo sapiens | China: Hubei | 2013 | 13-China_Hubei-124 | Ⅰ |
|  | MN510055 | Homo sapiens | China: Henan | 2013 | 13-China_Henan-126 | Ⅳ |
|  | MN510056 | Homo sapiens | China: Henan | 2013 | 13-China_Henan-128 | Ⅰ |
|  | OM452275 | Homo sapiens | China: Henan | 2013 | 13-China_Henan-129 | Ⅰ |
|  | OM452276 | Homo sapiens | China: Henan | 2013 | 13-China_Henan-130 | Ⅰ |
|  | OM452277 | Homo sapiens | China: Henan | 2013 | 13-China_Henan-132 | Ⅰ |
|  | OM452278 | Homo sapiens | China: Henan | 2013 | 13-China_Henan-139 | Ⅰ |
|  | OM452279 | Homo sapiens | China: Henan | 2013 | 13-China_Henan-141 | Ⅱ |
|  | OM452280 | Homo sapiens | China: Henan | 2013 | 13-China_Henan-142 | Ⅰ |
|  | OM452281 | Homo sapiens | China: Henan | 2013 | 13-China_Henan-144 | Ⅰ |
|  | OM452282 | Homo sapiens | China: Henan | 2013 | 13-China_Henan-147 | Ⅰ |
|  | OM452283 | Homo sapiens | China: Henan | 2013 | 13-China_Henan-154 | Ⅱ |
|  | OM452284 | Homo sapiens | China: Henan | 2013 | 13-China_Henan-155 | Ⅰ |
|  | OM452285 | Homo sapiens | China: Henan | 2013 | 13-China_Henan-160 | Ⅰ |
|  | OM452286 | Homo sapiens | China: Henan | 2013 | 13-China_Henan-166 | Ⅰ |
|  | OM452287 | Homo sapiens | China: Henan | 2013 | 13-China_Henan-170 | Ⅰ |
|  | OM452288 | Homo sapiens | China: Henan | 2013 | 13-China_Henan-177 | Ⅰ |
|  | OM452289 | Homo sapiens | China: Henan | 2013 | 13-China_Henan-178 | Ⅰ |
|  | OM452290 | Homo sapiens | China: Henan | 2013 | 13-China_Henan-181 | Ⅰ |
|  | OM452291 | Homo sapiens | China: Henan | 2013 | 13-China_Henan-192 | Ⅰ |
|  | OM452292 | Homo sapiens | China: Henan | 2013 | 13-China_Henan-195 | Ⅱ |
|  | MN510058 | Homo sapiens | China: Henan | 2013 | 13-China_Henan-198 | Ⅲ |
|  | OM452293 | Homo sapiens | China: Henan | 2013 | 13-China_Henan-200 | Ⅰ |
|  | OM452294 | Homo sapiens | China: Henan | 2013 | 13-China_Henan-201 | Ⅰ |
|  | OM452295 | Homo sapiens | China: Henan | 2013 | 13-China_Henan-212 | Ⅰ |
|  | OM452296 | Homo sapiens | China: Henan | 2013 | 13-China_Henan-213 | Ⅳ |
|  | OM452297 | Homo sapiens | China: Henan | 2013 | 13-China_Henan-219 | Ⅰ |
|  | OM452298 | Homo sapiens | China: Henan | 2013 | 13-China_Henan-221 | Ⅰ |
|  | OM452299 | Homo sapiens | China: Henan | 2013 | 13-China_Henan-224 | Ⅰ |
|  | OM452300 | Homo sapiens | China: Henan | 2013 | 13-China_Henan-229 | Ⅱ |
|  | OM452301 | Homo sapiens | China: Henan | 2013 | 13-China_Henan-238 | Ⅱ |
|  | MN510060 | Homo sapiens | China: Henan | 2013 | 13-China_Henan-24 | Ⅰ |
|  | OM452302 | Homo sapiens | China: Henan | 2013 | 13-China_Henan-240 | Ⅰ |
|  | OM452303 | Homo sapiens | China: Henan | 2013 | 13-China_Henan-246 | Ⅱ |
|  | OM452304 | Homo sapiens | China: Hubei | 2013 | 13-China_Hubei-252 | Ⅰ |
|  | OM452305 | Homo sapiens | China: Henan | 2013 | 13-China_Henan-254 | Ⅰ |
|  | OM452306 | Homo sapiens | China: Henan | 2013 | 13-China_Henan-255 | Ⅰ |
|  | MN510071 | Homo sapiens | China: Hubei | 2013 | 13-China_Hubei-256 | Ⅲ |
|  | OM452307 | Homo sapiens | China: Henan | 2013 | 13-China_Henan-258 | Ⅰ |
|  | OM452308 | Homo sapiens | China: Henan | 2013 | 13-China_Henan-262 | Ⅳ |
|  | OM452309 | Homo sapiens | China: Henan | 2013 | 13-China_Henan-263 | Ⅰ |
|  | OM452310 | Homo sapiens | China: Henan | 2013 | 13-China_Henan-264 | Ⅰ |
|  | OM452311 | Homo sapiens | China: Henan | 2013 | 13-China_Henan-266 | Ⅳ |
|  | OM452312 | Homo sapiens | China: Henan | 2013 | 13-China_Henan-271 | Ⅰ |
|  | OM452313 | Homo sapiens | China: Henan | 2013 | 13-China_Henan-275 | Ⅱ |
|  | OM452314 | Homo sapiens | China: Henan | 2013 | 13-China_Henan-279 | Ⅳ |
|  | OM452315 | Homo sapiens | China: Henan | 2013 | 13-China_Henan-280 | Ⅰ |
|  | MN510061 | Homo sapiens | China: Henan | 2013 | 13-China_Henan-288 | Ⅰ |
|  | OM452316 | Homo sapiens | China: Henan | 2013 | 13-China_Henan-29 | Ⅰ |
|  | OM452317 | Homo sapiens | China: Henan | 2013 | 13-China_Henan-292 | Ⅱ |
|  | OM452318 | Homo sapiens | China: Henan | 2013 | 13-China_Henan-293 | Ⅱ |
|  | OM452319 | Homo sapiens | China: Henan | 2013 | 13-China_Henan-294 | Ⅰ |
|  | OM452320 | Homo sapiens | China: Henan | 2013 | 13-China_Henan-295 | Ⅱ |
|  | MN510062 | Homo sapiens | China: Henan | 2013 | 13-China_Henan-297 | Ⅳ |
|  | OM452321 | Homo sapiens | China: Henan | 2013 | 13-China_Henan-299 | Ⅳ |
|  | OM452322 | Homo sapiens | China: Henan | 2013 | 13-China_Henan-30 | Ⅰ |
|  | OM452323 | Homo sapiens | China: Henan | 2013 | 13-China_Henan-300 | Ⅱ |
|  | OM452324 | Homo sapiens | China: Henan | 2013 | 13-China_Henan-302 | Ⅰ |
|  | OM452325 | Homo sapiens | China: Henan | 2013 | 13-China_Henan-304 | Ⅰ |
|  | OM452326 | Homo sapiens | China: Henan | 2013 | 13-China_Henan-31 | Ⅱ |
|  | OM452327 | Homo sapiens | China: Henan | 2013 | 13-China_Henan-310 | Ⅰ |
|  | OM452328 | Homo sapiens | China: Henan | 2013 | 13-China_Henan-311 | Ⅰ |
|  | OM452329 | Homo sapiens | China: Henan | 2013 | 13-China_Henan-313 | Ⅱ |
|  | OM452330 | Homo sapiens | China: Henan | 2013 | 13-China_Henan-320 | Ⅰ |
|  | MN510073 | Homo sapiens | China: Hubei | 2013 | 13-China_Hubei-321 | Ⅰ |
|  | OM452331 | Homo sapiens | China: Henan | 2013 | 13-China_Henan-324 | Ⅱ |
|  | MN510063 | Homo sapiens | China: Henan | 2013 | 13-China_Henan-357 | Ⅰ |
|  | OM452332 | Homo sapiens | China: Henan | 2013 | 13-China_Henan-364 | Ⅲ |
|  | OM452333 | Homo sapiens | China: Henan | 2013 | 13-China_Henan-368 | Ⅰ |
|  | OM452334 | Homo sapiens | China: Henan | 2013 | 13-China_Henan-369 | Ⅰ |
|  | OM452335 | Homo sapiens | China: Henan | 2013 | 13-China_Henan-37 | Ⅰ |
|  | OM452336 | Homo sapiens | China: Henan | 2013 | 13-China_Henan-371 | Ⅳ |
|  | OM452337 | Homo sapiens | China: Henan | 2013 | 13-China_Henan-38 | Ⅱ |
|  | OM452338 | Homo sapiens | China: Henan | 2013 | 13-China_Henan-382 | Ⅳ |
|  | OM452339 | Homo sapiens | China: Henan | 2013 | 13-China_Henan-39 | Ⅰ |
|  | MN510066 | Homo sapiens | China: Henan | 2013 | 13-China_Henan-392 | Ⅳ |
|  | OM452340 | Homo sapiens | China: Henan | 2013 | 13-China_Henan-398 | Ⅱ |
|  | OM452341 | Homo sapiens | China: Henan | 2013 | 13-China_Henan-40 | Ⅰ |
|  | OM452342 | Homo sapiens | China: Henan | 2013 | 13-China_Henan-408 | Ⅰ |
|  | OM452343 | Homo sapiens | China: Henan | 2013 | 13-China_Henan-420 | Ⅰ |
|  | MN510067 | Homo sapiens | China: Henan | 2013 | 13-China_Henan-423 | Ⅳ |
|  | OM452344 | Homo sapiens | China: Henan | 2013 | 13-China_Henan-424 | Ⅰ |
|  | OM452345 | Homo sapiens | China: Henan | 2013 | 13-China_Henan-425 | Ⅰ |
|  | OM452346 | Homo sapiens | China: Henan | 2013 | 13-China_Henan-428 | Ⅰ |
|  | OM452347 | Homo sapiens | China: Henan | 2013 | 13-China_Henan-429 | Ⅱ |
|  | OM452348 | Homo sapiens | China: Henan | 2013 | 13-China_Henan-44 | Ⅰ |
|  | OM452349 | Homo sapiens | China: Henan | 2013 | 13-China_Henan-45 | Ⅱ |
|  | MN510068 | Homo sapiens | China: Henan | 2013 | 13-China_Henan-48 | Ⅰ |
|  | OM452350 | Homo sapiens | China: Henan | 2013 | 13-China_Henan-50 | Ⅰ |
|  | OM452351 | Homo sapiens | China: Henan | 2013 | 13-China_Henan-52 | Ⅰ |
|  | OM452352 | Homo sapiens | China: Henan | 2013 | 13-China_Henan-53 | Ⅰ |
|  | OM452353 | Homo sapiens | China: Henan | 2013 | 13-China_Henan-54 | Ⅱ |
|  | OM452354 | Homo sapiens | China: Henan | 2013 | 13-China_Henan-55 | Ⅱ |
|  | OM452355 | Homo sapiens | China: Henan | 2013 | 13-China_Henan-60 | Ⅱ |
|  | OM452356 | Homo sapiens | China: Henan | 2013 | 13-China_Henan-62 | Ⅰ |
|  | OM452357 | Homo sapiens | China: Henan | 2013 | 13-China_Henan-65 | Ⅰ |
|  | OM452358 | Homo sapiens | China: Henan | 2013 | 13-China_Henan-67 | Ⅰ |
|  | OM452359 | Homo sapiens | China: Henan | 2013 | 13-China_Henan-68 | Ⅰ |
|  | OM452360 | Homo sapiens | China: Henan | 2013 | 13-China_Henan-77 | Ⅰ |
|  | OM452361 | Homo sapiens | China: Henan | 2013 | 13-China_Henan-78 | Ⅰ |
|  | MN510075 | Homo sapiens | China: Hubei | 2013 | 13-China_Hubei-79 | Ⅲ |
|  | OM452362 | Homo sapiens | China: Henan | 2013 | 13-China_Henan-86 | Ⅱ |
|  | OM452363 | Homo sapiens | China: Henan | 2013 | 13-China_Henan-87 | Ⅱ |
|  | OM452364 | Homo sapiens | China: Henan | 2013 | 13-China_Henan-91 | Ⅱ |
|  | OM452365 | Homo sapiens | China: Henan | 2013 | 13-China_Henan-93 | Ⅰ |
|  | OM452366 | Homo sapiens | China: Henan | 2013 | 13-China_Henan-94 | Ⅱ |
|  | OM452367 | Homo sapiens | China: Henan | 2014 | 14-China_Henan-107 | Ⅰ |
|  | OM452368 | Homo sapiens | China: Henan | 2014 | 14-China_Henan-109 | Ⅱ |
|  | OM452369 | Homo sapiens | China: Henan | 2014 | 14-China_Henan-11 | Ⅰ |
|  | OM452370 | Homo sapiens | China: Henan | 2014 | 14-China_Henan-118 | Ⅰ |
|  | OM452371 | Homo sapiens | China: Henan | 2014 | 14-China_Henan-129 | Ⅱ |
|  | OM452372 | Homo sapiens | China: Henan | 2014 | 14-China_Henan-13 | Ⅰ |
|  | OM452373 | Homo sapiens | China: Henan | 2014 | 14-China_Henan-134 | Ⅳ |
|  | OM452374 | Homo sapiens | China: Henan | 2014 | 14-China_Henan-143 | Ⅰ |
|  | OM452375 | Homo sapiens | China: Henan | 2014 | 14-China_Henan-148 | Ⅰ |
|  | OM452376 | Homo sapiens | China: Henan | 2014 | 14-China_Henan-149 | Ⅰ |
|  | OM452377 | Homo sapiens | China: Henan | 2014 | 14-China_Henan-15 | Ⅱ |
|  | OM452378 | Homo sapiens | China: Henan | 2014 | 14-China_Henan-151 | Ⅰ |
|  | OM452379 | Homo sapiens | China: Henan | 2014 | 14-China_Henan-152 | Ⅰ |
|  | OM452380 | Homo sapiens | China: Henan | 2014 | 14-China_Henan-172 | Ⅱ |
|  | OM452381 | Homo sapiens | China: Henan | 2014 | 14-China_Henan-174 | Ⅱ |
|  | OM452382 | Homo sapiens | China: Henan | 2014 | 14-China_Henan-175 | Ⅰ |
|  | OM452383 | Homo sapiens | China: Henan | 2014 | 14-China_Henan-177 | Ⅰ |
|  | OM452384 | Homo sapiens | China: Henan | 2014 | 14-China_Henan-178 | Ⅰ |
|  | OM452385 | Homo sapiens | China: Henan | 2014 | 14-China_Henan-189 | Ⅰ |
|  | OM452386 | Homo sapiens | China: Henan | 2014 | 14-China_Henan-191 | Ⅱ |
|  | OM452387 | Homo sapiens | China: Henan | 2014 | 14-China_Henan-193 | Ⅰ |
|  | OM452388 | Homo sapiens | China: Henan | 2014 | 14-China_Henan-20 | Ⅰ |
|  | OM452389 | Homo sapiens | China: Henan | 2014 | 14-China_Henan-22 | Ⅱ |
|  | OM452390 | Homo sapiens | China: Henan | 2014 | 14-China_Henan-222 | Ⅰ |
|  | OM452391 | Homo sapiens | China: Henan | 2014 | 14-China_Henan-236 | Ⅱ |
|  | OM452392 | Homo sapiens | China: Henan | 2014 | 14-China_Henan-26 | Ⅰ |
|  | OM452393 | Homo sapiens | China: Henan | 2014 | 14-China_Henan-3 | Ⅰ |
|  | OM452394 | Homo sapiens | China: Henan | 2014 | 14-China_Henan-307 | Ⅳ |
|  | OM452395 | Homo sapiens | China: Henan | 2014 | 14-China_Henan-311 | Ⅱ |
|  | OM452396 | Homo sapiens | China: Henan | 2014 | 14-China_Henan-312 | Ⅳ |
|  | OM452397 | Homo sapiens | China: Henan | 2014 | 14-China_Henan-314 | Ⅰ |
|  | OM452398 | Homo sapiens | China: Henan | 2014 | 14-China_Henan-316 | Ⅰ |
|  | OM452399 | Homo sapiens | China: Henan | 2014 | 14-China_Henan-320 | Ⅱ |
|  | OM452400 | Homo sapiens | China: Henan | 2014 | 14-China_Henan-326 | Ⅱ |
|  | OM452401 | Homo sapiens | China: Henan | 2014 | 14-China_Henan-337 | Ⅰ |
|  | OM452402 | Homo sapiens | China: Henan | 2014 | 14-China_Henan-346 | Ⅱ |
|  | OM452403 | Homo sapiens | China: Henan | 2014 | 14-China_Henan-35 | Ⅰ |
|  | OM452404 | Homo sapiens | China: Henan | 2014 | 14-China_Henan-403 | Ⅱ |
|  | OM452405 | Homo sapiens | China: Henan | 2014 | 14-China_Henan-422 | Ⅰ |
|  | OM452406 | Homo sapiens | China: Henan | 2014 | 14-China_Henan-424 | Ⅲ |
|  | OM452407 | Homo sapiens | China: Henan | 2014 | 14-China_Henan-436 | Ⅰ |
|  | OM452408 | Homo sapiens | China: Henan | 2014 | 14-China_Henan-438 | Ⅱ |
|  | OM452409 | Homo sapiens | China: Henan | 2014 | 14-China_Henan-440 | Ⅱ |
|  | OM452410 | Homo sapiens | China: Hubei | 2014 | 14-China_Hubei-444 | Ⅰ |
|  | OM452411 | Homo sapiens | China: Henan | 2014 | 14-China_Henan-446 | Ⅰ |
|  | OM452412 | Homo sapiens | China: Henan | 2014 | 14-China_Henan-452 | Ⅰ |
|  | OM452413 | Homo sapiens | China: Henan | 2014 | 14-China_Henan-453 | Ⅰ |
|  | OM452414 | Homo sapiens | China: Henan | 2014 | 14-China_Henan-463 | Ⅰ |
|  | OM452415 | Homo sapiens | China: Henan | 2014 | 14-China_Henan-474 | Ⅱ |
|  | OM452416 | Homo sapiens | China: Henan | 2014 | 14-China_Henan-484 | Ⅰ |
|  | OM452417 | Homo sapiens | China: Henan | 2014 | 14-China_Henan-485 | Ⅳ |
|  | OM452418 | Homo sapiens | China: Henan | 2014 | 14-China_Henan-488 | Ⅰ |
|  | OM452419 | Homo sapiens | China: Henan | 2014 | 14-China_Henan-49 | Ⅰ |
|  | OM452420 | Homo sapiens | China: Hubei | 2014 | 14-China_Hubei-492 | Ⅰ |
|  | OM452421 | Homo sapiens | China: Henan | 2014 | 14-China_Henan-494 | Ⅱ |
|  | OM452422 | Homo sapiens | China: Henan | 2014 | 14-China_Henan-499 | Ⅰ |
|  | OM452423 | Homo sapiens | China: Henan | 2014 | 14-China_Henan-5 | Ⅰ |
|  | OM452424 | Homo sapiens | China: Henan | 2014 | 14-China_Henan-501 | Ⅲ |
|  | OM452425 | Homo sapiens | China: Hubei | 2014 | 14-China_Hubei-512 | Ⅲ |
|  | OM452426 | Homo sapiens | China: Henan | 2014 | 14-China_Henan-523 | Ⅱ |
|  | OM452427 | Homo sapiens | China: Henan | 2014 | 14-China_Henan-529 | Ⅰ |
|  | OM452428 | Homo sapiens | China: Henan | 2014 | 14-China_Henan-54 | Ⅱ |
|  | OM452429 | Homo sapiens | China: Henan | 2014 | 14-China_Henan-540 | Ⅱ |
|  | OM452430 | Homo sapiens | China: Henan | 2014 | 14-China_Henan-542 | Ⅳ |
|  | OM452431 | Homo sapiens | China: Henan | 2014 | 14-China_Henan-546 | Ⅳ |
|  | OM452432 | Homo sapiens | China: Hubei | 2014 | 14-China_Hubei-548 | Ⅰ |
|  | OM452433 | Homo sapiens | China: Henan | 2014 | 14-China_Henan-55 | Ⅰ |
|  | OM452434 | Homo sapiens | China: Henan | 2014 | 14-China_Henan-555 | Ⅱ |
|  | OM452435 | Homo sapiens | China: Henan | 2014 | 14-China_Henan-559 | Ⅱ |
|  | OM452436 | Homo sapiens | China: Henan | 2014 | 14-China_Henan-561 | Ⅱ |
|  | OM452437 | Homo sapiens | China: Henan | 2014 | 14-China_Henan-562 | Ⅳ |
|  | OM452438 | Homo sapiens | China: Henan | 2014 | 14-China_Henan-563 | Ⅲ |
|  | OM452439 | Homo sapiens | China: Henan | 2014 | 14-China_Henan-564 | Ⅰ |
|  | OM452440 | Homo sapiens | China: Henan | 2014 | 14-China_Henan-567 | Ⅲ |
|  | OM452441 | Homo sapiens | China: Henan | 2014 | 14-China_Henan-568 | Ⅰ |
|  | MN510077 | Homo sapiens | China: Henan | 2014 | 14-China_Henan-570 | Ⅰ |
|  | OM452442 | Homo sapiens | China: Henan | 2014 | 14-China_Henan-571 | Ⅰ |
|  | OM452443 | Homo sapiens | China: Henan | 2014 | 14-China_Henan-573 | Ⅰ |
|  | OM452444 | Homo sapiens | China: Henan | 2014 | 14-China_Henan-574 | Ⅳ |
|  | MN510078 | Homo sapiens | China: Henan | 2014 | 14-China_Henan-578 | Ⅰ |
|  | OM452445 | Homo sapiens | China: Henan | 2014 | 14-China_Henan-579 | Ⅱ |
|  | OM452446 | Homo sapiens | China: Henan | 2014 | 14-China_Henan-58 | Ⅰ |
|  | OM452447 | Homo sapiens | China: Henan | 2014 | 14-China_Henan-581 | Ⅰ |
|  | OM452448 | Homo sapiens | China: Henan | 2014 | 14-China_Henan-582 | Ⅰ |
|  | OM452449 | Homo sapiens | China: Henan | 2014 | 14-China_Henan-586 | Ⅱ |
|  | OM452450 | Homo sapiens | China: Henan | 2014 | 14-China_Henan-592 | Ⅰ |
|  | OM452451 | Homo sapiens | China: Henan | 2014 | 14-China_Henan-593 | Ⅰ |
|  | OM452452 | Homo sapiens | China: Henan | 2014 | 14-China_Henan-600 | Ⅳ |
|  | OM452453 | Homo sapiens | China: Henan | 2014 | 14-China_Henan-601 | Ⅰ |
|  | OM452454 | Homo sapiens | China: Henan | 2014 | 14-China_Henan-604 | Ⅰ |
|  | OM452455 | Homo sapiens | China: Henan | 2014 | 14-China_Henan-605 | Ⅰ |
|  | OM452456 | Homo sapiens | China: Henan | 2014 | 14-China_Henan-606 | Ⅰ |
|  | OM452457 | Homo sapiens | China: Henan | 2014 | 14-China_Henan-607 | Ⅰ |
|  | OM452458 | Homo sapiens | China: Henan | 2014 | 14-China_Henan-61 | Ⅰ |
|  | OM452459 | Homo sapiens | China: Henan | 2014 | 14-China_Henan-610 | Ⅰ |
|  | OM452460 | Homo sapiens | China: Henan | 2014 | 14-China_Henan-613 | Ⅰ |
|  | OM452461 | Homo sapiens | China: Henan | 2014 | 14-China_Henan-617 | Ⅰ |
|  | OM452462 | Homo sapiens | China: Henan | 2014 | 14-China_Henan-62 | Ⅱ |
|  | OM452463 | Homo sapiens | China: Henan | 2014 | 14-China_Henan-63 | Ⅰ |
|  | OM452464 | Homo sapiens | China: Henan | 2014 | 14-China_Henan-66 | Ⅰ |
|  | OM452465 | Homo sapiens | China: Henan | 2014 | 14-China_Henan-68 | Ⅰ |
|  | OM452466 | Homo sapiens | China: Henan | 2014 | 14-China_Henan-69 | Ⅰ |
|  | OM452467 | Homo sapiens | China: Henan | 2014 | 14-China_Henan-70 | Ⅰ |
|  | OM452468 | Homo sapiens | China: Henan | 2014 | 14-China_Henan-71 | Ⅱ |
|  | OM452469 | Homo sapiens | China: Henan | 2014 | 14-China_Henan-74 | Ⅰ |
|  | OM452470 | Homo sapiens | China: Henan | 2014 | 14-China_Henan-76 | Ⅰ |
|  | OM452471 | Homo sapiens | China: Henan | 2014 | 14-China_Henan-79 | Ⅰ |
|  | OM452472 | Homo sapiens | China: Henan | 2014 | 14-China_Henan-84 | Ⅱ |
|  | OM452473 | Homo sapiens | China: Henan | 2014 | 14-China_Henan-86 | Ⅰ |
|  | OM452474 | Homo sapiens | China: Henan | 2014 | 14-China_Henan-9 | Ⅰ |
|  | OM452475 | Homo sapiens | China: Henan | 2016 | 16-China_Henan-110 | Ⅳ |
|  | OM452476 | Homo sapiens | China: Henan | 2015 | 15-China_Henan-113 | Ⅰ |
|  | OM452477 | Homo sapiens | China: Henan | 2015 | 15-China_Henan-117 | Ⅰ |
|  | OM452478 | Homo sapiens | China: Henan | 2015 | 15-China_Henan-123 | Ⅰ |
|  | OM452479 | Homo sapiens | China: Henan | 2015 | 15-China_Henan-127 | Ⅰ |
|  | OM452480 | Homo sapiens | China: Henan | 2015 | 15-China_Henan-130 | Ⅲ |
|  | OM452481 | Homo sapiens | China: Henan | 2015 | 15-China_Henan-132 | Ⅰ |
|  | OM452482 | Homo sapiens | China: Henan | 2015 | 15-China_Henan-134 | Ⅰ |
|  | OM452483 | Homo sapiens | China: Henan | 2016 | 16-China_Henan-136 | Ⅳ |
|  | OM452484 | Homo sapiens | China: Henan | 2015 | 15-China_Henan-141 | Ⅱ |
|  | OM452485 | Homo sapiens | China: Henan | 2015 | 15-China_Henan-147 | Ⅱ |
|  | OM452486 | Homo sapiens | China: Henan | 2015 | 15-China_Henan-15 | Ⅰ |
|  | OM452487 | Homo sapiens | China: Henan | 2015 | 15-China_Henan-150 | Ⅰ |
|  | OM452488 | Homo sapiens | China: Henan | 2015 | 15-China_Henan-151 | Ⅰ |
|  | OM452489 | Homo sapiens | China: Henan | 2015 | 15-China_Henan-153 | Ⅰ |
|  | MN510102 | Homo sapiens | China: Hubei | 2015 | 15-China_Hubei-154 | Ⅰ |
|  | OM452490 | Homo sapiens | China: Henan | 2015 | 15-China_Henan-155 | Ⅱ |
|  | OM452491 | Homo sapiens | China: Henan | 2015 | 15-China_Henan-16 | Ⅳ |
|  | OM452492 | Homo sapiens | China: Henan | 2015 | 15-China_Henan-160 | Ⅰ |
|  | OM452493 | Homo sapiens | China: Henan | 2015 | 15-China_Henan-165 | Ⅱ |
|  | OM452494 | Homo sapiens | China: Henan | 2015 | 15-China_Henan-168 | Ⅱ |
|  | OM452495 | Homo sapiens | China: Henan | 2015 | 15-China_Henan-170 | Ⅰ |
|  | OM452496 | Homo sapiens | China: Henan | 2015 | 15-China_Henan-174 | Ⅰ |
|  | OM452497 | Homo sapiens | China: Henan | 2015 | 15-China_Henan-176 | Ⅰ |
|  | OM452498 | Homo sapiens | China: Henan | 2015 | 15-China_Henan-178 | Ⅰ |
|  | OM452499 | Homo sapiens | China: Henan | 2015 | 15-China_Henan-179 | Ⅱ |
|  | OM452500 | Homo sapiens | China: Henan | 2015 | 15-China_Henan-2 | Ⅰ |
|  | OM452501 | Homo sapiens | China: Henan | 2015 | 15-China_Henan-200 | Ⅰ |
|  | MN510081 | Homo sapiens | China: Henan | 2015 | 15-China_Henan-201 | Ⅰ |
|  | MN510082 | Homo sapiens | China: Henan | 2015 | 15-China_Henan-204 | Ⅳ |
|  | OM452502 | Homo sapiens | China: Henan | 2015 | 15-China_Henan-206 | Ⅱ |
|  | OM452503 | Homo sapiens | China: Henan | 2015 | 15-China_Henan-211 | Ⅰ |
|  | OM452504 | Homo sapiens | China: Henan | 2015 | 15-China_Henan-213 | Ⅳ |
|  | MN510083 | Homo sapiens | China: Henan | 2015 | 15-China_Henan-217 | Ⅲ |
|  | OM452505 | Homo sapiens | China: Henan | 2015 | 15-China_Henan-221 | Ⅰ |
|  | OM452506 | Homo sapiens | China: Henan | 2015 | 15-China_Henan-224 | Ⅳ |
|  | OM452507 | Homo sapiens | China: Henan | 2015 | 15-China_Henan-225 | Ⅰ |
|  | MN510084 | Homo sapiens | China: Henan | 2015 | 15-China_Henan-226 | Ⅱ |
|  | OM452508 | Homo sapiens | China: Henan | 2015 | 15-China_Henan-227 | Ⅰ |
|  | OM452509 | Homo sapiens | China: Henan | 2015 | 15-China_Henan-228 | Ⅱ |
|  | OM452510 | Homo sapiens | China: Henan | 2015 | 15-China_Henan-229 | Ⅰ |
|  | MN510085 | Homo sapiens | China: Henan | 2015 | 15-China_Henan-23 | Ⅳ |
|  | OM452511 | Homo sapiens | China: Henan | 2015 | 15-China_Henan-232 | Ⅰ |
|  | MN510103 | Homo sapiens | China: Hubei | 2015 | 15-China_Hubei-234 | Ⅲ |
|  | OM452512 | Homo sapiens | China: Henan | 2015 | 15-China_Henan-238 | Ⅰ |
|  | OM452513 | Homo sapiens | China: Henan | 2015 | 15-China_Henan-249 | Ⅰ |
|  | OM452514 | Homo sapiens | China: Henan | 2015 | 15-China_Henan-250 | Ⅰ |
|  | MN510086 | Homo sapiens | China: Henan | 2015 | 15-China_Henan-256 | Ⅰ |
|  | OM452515 | Homo sapiens | China: Henan | 2015 | 15-China_Henan-257 | Ⅰ |
|  | OM452516 | Homo sapiens | China: Henan | 2015 | 15-China_Henan-258 | Ⅰ |
|  | MN510105 | Homo sapiens | China: Hubei | 2015 | 15-China_Hubei-259 | Ⅰ |
|  | MN510087 | Homo sapiens | China: Henan | 2015 | 15-China_Henan-260 | Ⅲ |
|  | OM452517 | Homo sapiens | China: Henan | 2015 | 15-China_Henan-266 | Ⅰ |
|  | OM452518 | Homo sapiens | China: Henan | 2015 | 15-China_Henan-29 | Ⅳ |
|  | OM452519 | Homo sapiens | China: Henan | 2015 | 15-China_Henan-30 | Ⅰ |
|  | OM452520 | Homo sapiens | China: Henan | 2015 | 15-China_Henan-316 | Ⅰ |
|  | OM452521 | Homo sapiens | China: Henan | 2015 | 15-China_Henan-321 | Ⅱ |
|  | MN510088 | Homo sapiens | China: Henan | 2015 | 15-China_Henan-322 | Ⅰ |
|  | OM452522 | Homo sapiens | China: Henan | 2015 | 15-China_Henan-323 | Ⅰ |
|  | OM452523 | Homo sapiens | China: Henan | 2015 | 15-China_Henan-326 | Ⅰ |
|  | MN510090 | Homo sapiens | China: Henan | 2015 | 15-China_Henan-338 | Ⅰ |
|  | OM452524 | Homo sapiens | China: Henan | 2015 | 15-China_Henan-34 | Ⅰ |
|  | OM452525 | Homo sapiens | China: Henan | 2015 | 15-China_Henan-343 | Ⅰ |
|  | OM452526 | Homo sapiens | China: Henan | 2015 | 15-China_Henan-35 | Ⅰ |
|  | OM452527 | Homo sapiens | China: Henan | 2015 | 15-China_Henan-351 | Ⅱ |
|  | OM452528 | Homo sapiens | China: Henan | 2015 | 15-China_Henan-353 | Ⅳ |
|  | MN510091 | Homo sapiens | China: Henan | 2015 | 15-China_Henan-356 | Ⅰ |
|  | OM452529 | Homo sapiens | China: Henan | 2015 | 15-China_Henan-363 | Ⅰ |
|  | OM452530 | Homo sapiens | China: Henan | 2016 | 16-China_Henan-364 | Ⅳ |
|  | OM452531 | Homo sapiens | China: Henan | 2015 | 15-China_Henan-365 | Ⅰ |
|  | OM452532 | Homo sapiens | China: Henan | 2015 | 15-China_Henan-374 | Ⅱ |
|  | MN510092 | Homo sapiens | China: Henan | 2015 | 15-China_Henan-380 | Ⅰ |
|  | OM452533 | Homo sapiens | China: Henan | 2015 | 15-China_Henan-391 | Ⅰ |
|  | MN510094 | Homo sapiens | China: Henan | 2015 | 15-China_Henan-393 | Ⅰ |
|  | MN510095 | Homo sapiens | China: Henan | 2015 | 15-China_Henan-398 | Ⅳ |
|  | OM452534 | Homo sapiens | China: Henan | 2015 | 15-China_Henan-4 | Ⅰ |
|  | OM452535 | Homo sapiens | China: Henan | 2015 | 15-China_Henan-40 | Ⅰ |
|  | OM452536 | Homo sapiens | China: Henan | 2015 | 15-China_Henan-411 | Ⅱ |
|  | OM452537 | Homo sapiens | China: Henan | 2015 | 15-China_Henan-413 | Ⅰ |
|  | OM452538 | Homo sapiens | China: Henan | 2015 | 15-China_Henan-414 | Ⅰ |
|  | OM452539 | Homo sapiens | China: Henan | 2015 | 15-China_Henan-415 | Ⅰ |
|  | MN510107 | Homo sapiens | China: Henan | 2015 | 15-China_Henan-55 | Ⅳ |
|  | OM452540 | Homo sapiens | China: Henan | 2015 | 15-China_Henan-58 | Ⅱ |
|  | OM452541 | Homo sapiens | China: Henan | 2015 | 15-China_Henan-61 | Ⅰ |
|  | OM452542 | Homo sapiens | China: Henan | 2015 | 15-China_Henan-62 | Ⅰ |
|  | OM452543 | Homo sapiens | China: Henan | 2015 | 15-China_Henan-68 | Ⅰ |
|  | OM452544 | Homo sapiens | China: Henan | 2015 | 15-China_Henan-7 | Ⅰ |
|  | OM452545 | Homo sapiens | China: Henan | 2015 | 15-China_Henan-73 | Ⅰ |
|  | OM452546 | Homo sapiens | China: Henan | 2015 | 15-China_Henan-75 | Ⅰ |
|  | OM452547 | Homo sapiens | China: Henan | 2015 | 15-China_Henan-78 | Ⅳ |
|  | OM452548 | Homo sapiens | China: Henan | 2015 | 15-China_Henan-79 | Ⅰ |
|  | OM452549 | Homo sapiens | China: Henan | 2015 | 15-China_Henan-8 | Ⅰ |
|  | OM452550 | Homo sapiens | China: Henan | 2015 | 15-China_Henan-82 | Ⅰ |
|  | OM452551 | Homo sapiens | China: Henan | 2015 | 15-China_Henan-9 | Ⅰ |
|  | OM452552 | Homo sapiens | China: Henan | 2015 | 15-China_Henan-91 | Ⅱ |
|  | OM452553 | Homo sapiens | China: Henan | 2015 | 15-China_Henan-95 | Ⅰ |
|  | OM452554 | Homo sapiens | China: Henan | 2016 | 16-China_Henan-115 | Ⅱ |
|  | OM452555 | Homo sapiens | China: Henan | 2016 | 16-China_Henan-118 | Ⅰ |
|  | OM452556 | Homo sapiens | China: Henan | 2016 | 16-China_Henan-122 | Ⅰ |
|  | OM452557 | Homo sapiens | China: Henan | 2016 | 16-China_Henan-123 | Ⅰ |
|  | OM452558 | Homo sapiens | China: Henan | 2016 | 16-China_Henan-128 | Ⅰ |
|  | MN510108 | Homo sapiens | China: Henan | 2016 | 16-China_Henan-135 | Ⅰ |
|  | MN510109 | Homo sapiens | China: Henan | 2016 | 16-China_Henan-138 | Ⅰ |
|  | MN510110 | Homo sapiens | China: Henan | 2016 | 16-China_Henan-149 | Ⅰ |
|  | OM452559 | Homo sapiens | China: Henan | 2016 | 16-China_Henan-16 | Ⅱ |
|  | OM452560 | Homo sapiens | China: Henan | 2016 | 16-China_Henan-164 | Ⅰ |
|  | OM452561 | Homo sapiens | China: Henan | 2016 | 16-China_Henan-167 | Ⅱ |
|  | MN510111 | Homo sapiens | China: Henan | 2016 | 16-China_Henan-17 | Ⅰ |
|  | MN510112 | Homo sapiens | China: Henan | 2016 | 16-China_Henan-176 | Ⅰ |
|  | OM452562 | Homo sapiens | China: Henan | 2016 | 16-China_Henan-179 | Ⅱ |
|  | OM452563 | Homo sapiens | China: Henan | 2016 | 16-China_Henan-18 | Ⅰ |
|  | OM452564 | Homo sapiens | China: Henan | 2016 | 16-China_Henan-181 | Ⅰ |
|  | OM452565 | Homo sapiens | China: Henan | 2016 | 16-China_Henan-187 | Ⅰ |
|  | OM452566 | Homo sapiens | China: Henan | 2016 | 16-China_Henan-188 | Ⅱ |
|  | OM452567 | Homo sapiens | China: Henan | 2016 | 16-China_Henan-19 | Ⅰ |
|  | OM452568 | Homo sapiens | China: Henan | 2016 | 16-China_Henan-196 | Ⅱ |
|  | OM452569 | Homo sapiens | China: Henan | 2016 | 16-China_Henan-198 | Ⅰ |
|  | MN510128 | Homo sapiens | China: Hubei | 2016 | 16-China_Hubei-203 | Ⅰ |
|  | OM452570 | Homo sapiens | China: Henan | 2016 | 16-China_Henan-212 | Ⅳ |
|  | MN510113 | Homo sapiens | China: Henan | 2016 | 16-China_Henan-245 | Ⅰ |
|  | OM452571 | Homo sapiens | China: Henan | 2016 | 16-China_Henan-251 | Ⅱ |
|  | OM452572 | Homo sapiens | China: Henan | 2016 | 16-China_Henan-279 | Ⅰ |
|  | OM452573 | Homo sapiens | China: Henan | 2016 | 16-China_Henan-283 | Ⅰ |
|  | OM452574 | Homo sapiens | China: Henan | 2016 | 16-China_Henan-286 | Ⅰ |
|  | OM452575 | Homo sapiens | China: Henan | 2016 | 16-China_Henan-29 | Ⅰ |
|  | OM452576 | Homo sapiens | China: Henan | 2016 | 16-China_Henan-305 | Ⅱ |
|  | OM452577 | Homo sapiens | China: Henan | 2016 | 16-China_Henan-32 | Ⅰ |
|  | OM452578 | Homo sapiens | China: Henan | 2016 | 16-China_Henan-335 | Ⅱ |
|  | OM452579 | Homo sapiens | China: Henan | 2016 | 16-China_Henan-341 | Ⅱ |
|  | MN510115 | Homo sapiens | China: Henan | 2016 | 16-China_Henan-370 | Ⅴ |
|  | OM452580 | Homo sapiens | China: Henan | 2016 | 16-China_Henan-39 | Ⅲ |
|  | OM452581 | Homo sapiens | China: Henan | 2016 | 16-China_Henan-397 | Ⅱ |
|  | OM452582 | Homo sapiens | China: Henan | 2016 | 16-China_Henan-401 | Ⅱ |
|  | MN510116 | Homo sapiens | China: Henan | 2016 | 16-China_Henan-402 | Ⅰ |
|  | OM452583 | Homo sapiens | China: Henan | 2016 | 16-China_Henan-405 | Ⅱ |
|  | MN510117 | Homo sapiens | China: Henan | 2016 | 16-China_Henan-412 | Ⅰ |
|  | MN510118 | Homo sapiens | China: Henan | 2016 | 16-China_Henan-418 | Ⅰ |
|  | MN510119 | Homo sapiens | China: Henan | 2016 | 16-China_Henan-421 | Ⅰ |
|  | MN510120 | Homo sapiens | China: Henan | 2016 | 16-China_Henan-428 | Ⅰ |
|  | MN510121 | Homo sapiens | China: Henan | 2016 | 16-China_Henan-431 | Ⅰ |
|  | MN510122 | Homo sapiens | China: Henan | 2016 | 16-China_Henan-439 | Ⅰ |
|  | MN510123 | Homo sapiens | China: Henan | 2016 | 16-China_Henan-444 | Ⅰ |
|  | OM452584 | Homo sapiens | China: Henan | 2016 | 16-China_Henan-452 | Ⅱ |
|  | MN510124 | Homo sapiens | China: Henan | 2016 | 16-China_Henan-453 | Ⅲ |
|  | MN510125 | Homo sapiens | China: Henan | 2016 | 16-China_Henan-456 | Ⅰ |
|  | OM452585 | Homo sapiens | China: Hubei | 2016 | 16-China_Hubei-471 | Ⅰ |
|  | OM452586 | Homo sapiens | China: Henan | 2016 | 16-China_Henan-504 | Ⅱ |
|  | OM452587 | Homo sapiens | China: Henan | 2016 | 16-China_Henan-532 | Ⅰ |
|  | MN510126 | Homo sapiens | China: Henan | 2016 | 16-China_Henan-590 | Ⅰ |
|  | OM452588 | Homo sapiens | China: Henan | 2016 | 16-China_Henan-595 | Ⅱ |
|  | OM452589 | Homo sapiens | China: Henan | 2016 | 16-China_Henan-598 | Ⅲ |
|  | OM452590 | Homo sapiens | China: Henan | 2016 | 16-China_Henan-605 | Ⅰ |
|  | OM452591 | Homo sapiens | China: Henan | 2016 | 16-China_Henan-614 | Ⅰ |
|  | OM452592 | Homo sapiens | China: Henan | 2016 | 16-China_Henan-64 | Ⅰ |
|  | OM452593 | Homo sapiens | China: Henan | 2016 | 16-China_Henan-74 | Ⅱ |
|  | OM452594 | Homo sapiens | China: Henan | 2016 | 16-China_Henan-81 | Ⅳ |
|  | MN510129 | Homo sapiens | China: Henan | 2017 | 17-China_Henan-110 | Ⅰ |
|  | MN510130 | Homo sapiens | China: Henan | 2017 | 17-China_Henan-112 | Ⅰ |
|  | MN510131 | Homo sapiens | China: Henan | 2017 | 17-China_Henan-116 | Ⅰ |
|  | OM452595 | Homo sapiens | China: Henan | 2017 | 17-China_Henan-138 | Ⅱ |
|  | MN510132 | Homo sapiens | China: Henan | 2017 | 17-China_Henan-142 | Ⅰ |
|  | MN510133 | Homo sapiens | China: Henan | 2017 | 17-China_Henan-143 | Ⅰ |
|  | OM452596 | Homo sapiens | China: Henan | 2017 | 17-China_Henan-154 | Ⅰ |
|  | OM452597 | Homo sapiens | China: Henan | 2017 | 17-China_Henan-178 | Ⅰ |
|  | MN510134 | Homo sapiens | China: Henan | 2017 | 17-China_Henan-215 | Ⅰ |
|  | MN510135 | Homo sapiens | China: Henan | 2017 | 17-China_Henan-218 | Ⅳ |
|  | OM452598 | Homo sapiens | China: Henan | 2017 | 17-China_Henan-242 | Ⅱ |
|  | MN510150 | Homo sapiens | China: Hubei | 2017 | 17-China_Hubei-25 | Ⅲ |
|  | MN510136 | Homo sapiens | China: Henan | 2017 | 17-China_Henan-257 | Ⅳ |
|  | MN510137 | Homo sapiens | China: Henan | 2017 | 17-China_Henan-290 | Ⅰ |
|  | OM452599 | Homo sapiens | China: Henan | 2017 | 17-China_Henan-317 | Ⅰ |
|  | OM452600 | Homo sapiens | China: Henan | 2017 | 17-China_Henan-320 | Ⅱ |
|  | MN510138 | Homo sapiens | China: Henan | 2017 | 17-China_Henan-337 | Ⅰ |
|  | MN510139 | Homo sapiens | China: Henan | 2017 | 17-China_Henan-35 | Ⅰ |
|  | MN510140 | Homo sapiens | China: Henan | 2017 | 17-China_Henan-356 | Ⅰ |
|  | MN510141 | Homo sapiens | China: Henan | 2017 | 17-China_Henan-365 | Ⅰ |
|  | MN510142 | Homo sapiens | China: Henan | 2017 | 17-China_Henan-51 | Ⅰ |
|  | MN510143 | Homo sapiens | China: Henan | 2017 | 17-China_Henan-64 | Ⅰ |
|  | MN510145 | Homo sapiens | China: Henan | 2017 | 17-China_Henan-76 | Ⅲ |
|  | MN510146 | Homo sapiens | China: Henan | 2017 | 17-China_Henan-84 | Ⅲ |
|  | OM452601 | Homo sapiens | China: Henan | 2017 | 17-China_Henan-91 | Ⅱ |
|  | MN510147 | Homo sapiens | China: Henan | 2017 | 17-China_Henan-94 | Ⅰ |
|  | MN510148 | Homo sapiens | China: Henan | 2017 | 17-China_Henan-96 | Ⅲ |
|  | OM452602 | Homo sapiens | China: Henan | 2017 | 17-China_Henan-101 | Ⅰ |
|  | OM452603 | Homo sapiens | China: Hubei | 2017 | 17-China_Hubei-102 | Ⅰ |
|  | MN510152 | Homo sapiens | China: Henan | 2018 | 18-China_Henan-103 | Ⅰ |
|  | OM452604 | Homo sapiens | China: Henan | 2018 | 18-China_Henan-105 | Ⅱ |
|  | OM452605 | Homo sapiens | China: Henan | 2017 | 17-China_Henan-107 | Ⅰ |
|  | OM452606 | Homo sapiens | China: Henan | 2018 | 18-China_Henan-109 | Ⅰ |
|  | OM452607 | Homo sapiens | China: Henan | 2018 | 18-China_Henan-110 | Ⅱ |
|  | OM452608 | Homo sapiens | China: Henan | 2017 | 17-China_Henan-114 | Ⅰ |
|  | OM452609 | Homo sapiens | China: Henan | 2017 | 17-China_Henan-113 | Ⅰ |
|  | OM452610 | Homo sapiens | China: Henan | 2018 | 18-China_Henan-114 | Ⅳ |
|  | OM452611 | Homo sapiens | China: Henan | 2018 | 18-China_Henan-115 | Ⅱ |
|  | MN510153 | Homo sapiens | China: Hubei | 2018 | 18-China_Hubei-118 | Ⅰ |
|  | OM452612 | Homo sapiens | China: Hubei | 2017 | 17-China_Hubei-120 | Ⅰ |
|  | OM452613 | Homo sapiens | China: Hubei | 2018 | 18-China_Hubei-123 | Ⅲ |
|  | OM452614 | Homo sapiens | China: Henan | 2017 | 17-China_Henan-128 | Ⅰ |
|  | OM452615 | Homo sapiens | China: Henan | 2018 | 18-China_Henan-129 | Ⅱ |
|  | OM452616 | Homo sapiens | China: Henan | 2017 | 17-China_Henan-13 | Ⅰ |
|  | OM452617 | Homo sapiens | China: Henan | 2018 | 18-China_Henan-130 | Ⅰ |
|  | OM452618 | Homo sapiens | China: Henan | 2017 | 17-China_Henan-136 | Ⅱ |
|  | OM452619 | Homo sapiens | China: Henan | 2017 | 17-China_Henan-139 | Ⅰ |
|  | OM452620 | Homo sapiens | China: Henan | 2018 | 18-China_Henan-139 | Ⅰ |
|  | OM452621 | Homo sapiens | China: Henan | 2018 | 18-China_Henan-141 | Ⅰ |
|  | OM452622 | Homo sapiens | China: Henan | 2018 | 18-China_Henan-143 | Ⅰ |
|  | OM452623 | Homo sapiens | China: Hubei | 2017 | 17-China_Hubei-144 | Ⅰ |
|  | OM452624 | Homo sapiens | China: Henan | 2017 | 17-China_Henan-147 | Ⅰ |
|  | OM452625 | Homo sapiens | China: Henan | 2017 | 17-China_Henan-148 | Ⅰ |
|  | OM452626 | Homo sapiens | China: Henan | 2018 | 18-China_Henan-149 | Ⅰ |
|  | OM452627 | Homo sapiens | China: Henan | 2018 | 18-China_Henan-150 | Ⅰ |
|  | OM452628 | Homo sapiens | China: Henan | 2018 | 18-China_Henan-152 | Ⅱ |
|  | MN510155 | Homo sapiens | China: Henan | 2018 | 18-China_Henan-153 | Ⅳ |
|  | OM452629 | Homo sapiens | China: Henan | 2018 | 18-China_Henan-154 | Ⅰ |
|  | OM452630 | Homo sapiens | China: Henan | 2018 | 18-China_Henan-155 | Ⅱ |
|  | OM452631 | Homo sapiens | China: Henan | 2018 | 18-China_Henan-158 | Ⅱ |
|  | OM452632 | Homo sapiens | China: Henan | 2018 | 18-China_Henan-159 | Ⅰ |
|  | OM452633 | Homo sapiens | China: Henan | 2018 | 18-China_Henan-160 | Ⅰ |
|  | OM452634 | Homo sapiens | China: Henan | 2018 | 18-China_Henan-161 | Ⅰ |
|  | OM452635 | Homo sapiens | China: Henan | 2017 | 17-China_Henan-163 | Ⅰ |
|  | OM452636 | Homo sapiens | China: Henan | 2018 | 18-China_Henan-164 | Ⅳ |
|  | OM452637 | Homo sapiens | China: Henan | 2017 | 17-China_Henan-165 | Ⅳ |
|  | OM452638 | Homo sapiens | China: Hubei | 2018 | 18-China_Hubei-167 | Ⅰ |
|  | MN510156 | Homo sapiens | China: Henan | 2018 | 18-China_Henan-169 | Ⅰ |
|  | OM452639 | Homo sapiens | China: Henan | 2018 | 18-China_Henan-170 | Ⅰ |
|  | MN510157 | Homo sapiens | China: Henan | 2018 | 18-China_Henan-172 | Ⅳ |
|  | OM452640 | Homo sapiens | China: Henan | 2017 | 17-China_Henan-177 | Ⅰ |
|  | MN510159 | Homo sapiens | China: Henan | 2018 | 18-China_Henan-178 | Ⅰ |
|  | OM452641 | Homo sapiens | China: Henan | 2018 | 18-China_Henan-180 | Ⅰ |
|  | OM452642 | Homo sapiens | China: Henan | 2018 | 18-China_Henan-184 | Ⅰ |
|  | OM452643 | Homo sapiens | China: Hubei | 2018 | 18-China_Hubei-186 | Ⅲ |
|  | MN510160 | Homo sapiens | China: Henan | 2018 | 18-China_Henan-187 | Ⅰ |
|  | OM452644 | Homo sapiens | China: Henan | 2018 | 18-China_Henan-189 | Ⅰ |
|  | OM452645 | Homo sapiens | China: Henan | 2017 | 17-China_Henan-190 | Ⅳ |
|  | OM452646 | Homo sapiens | China: Henan | 2018 | 18-China_Henan-191 | Ⅰ |
|  | OM452647 | Homo sapiens | China: Henan | 2018 | 18-China_Henan-193 | Ⅰ |
|  | OM452648 | Homo sapiens | China: Henan | 2017 | 17-China_Henan-194 | Ⅱ |
|  | OM452649 | Homo sapiens | China: Hubei | 2018 | 18-China_Hubei-199 | Ⅰ |
|  | OM452650 | Homo sapiens | China: Henan | 2018 | 18-China_Henan-2 | Ⅲ |
|  | OM452651 | Homo sapiens | China: Henan | 2018 | 18-China_Henan-200 | Ⅱ |
|  | OM452652 | Homo sapiens | China: Henan | 2018 | 18-China_Henan-207 | Ⅰ |
|  | MN510162 | Homo sapiens | China: Henan | 2018 | 18-China_Henan-208 | Ⅳ |
|  | OM452653 | Homo sapiens | China: Henan | 2018 | 18-China_Henan-209 | Ⅰ |
|  | OM452654 | Homo sapiens | China: Henan | 2018 | 18-China_Henan-210 | Ⅰ |
|  | OM452655 | Homo sapiens | China: Henan | 2018 | 18-China_Henan-213 | Ⅰ |
|  | OM452656 | Homo sapiens | China: Henan | 2018 | 18-China_Henan-217 | Ⅰ |
|  | OM452657 | Homo sapiens | China: Henan | 2018 | 18-China_Henan-22 | Ⅱ |
|  | MN510163 | Homo sapiens | China: Henan | 2018 | 18-China_Henan-222 | Ⅳ |
|  | OM452658 | Homo sapiens | China: Henan | 2018 | 18-China_Henan-223 | Ⅰ |
|  | OM452659 | Homo sapiens | China: Henan | 2018 | 18-China_Henan-225 | Ⅰ |
|  | OM452660 | Homo sapiens | China: Henan | 2018 | 18-China_Henan-226 | Ⅰ |
|  | MN510164 | Homo sapiens | China: Henan | 2018 | 18-China_Henan-227 | Ⅳ |
|  | OM452661 | Homo sapiens | China: Henan | 2018 | 18-China_Henan-228 | Ⅰ |
|  | OM452662 | Homo sapiens | China: Henan | 2018 | 18-China_Henan-233 | Ⅳ |
|  | OM452663 | Homo sapiens | China: Henan | 2017 | 17-China_Henan-241 | Ⅱ |
|  | OM452664 | Homo sapiens | China: Henan | 2018 | 18-China_Henan-243 | Ⅱ |
|  | OM452665 | Homo sapiens | China: Henan | 2018 | 18-China_Henan-251 | Ⅰ |
|  | OM452666 | Homo sapiens | China: Henan | 2018 | 18-China_Henan-254 | Ⅰ |
|  | OM452667 | Homo sapiens | China: Henan | 2017 | 17-China_Henan-255 | Ⅰ |
|  | OM452668 | Homo sapiens | China: Henan | 2018 | 18-China_Henan-257 | Ⅰ |
|  | OM452669 | Homo sapiens | China: Henan | 2018 | 18-China_Henan-258 | Ⅰ |
|  | OM452670 | Homo sapiens | China: Henan | 2017 | 17-China_Henan-261 | Ⅰ |
|  | OM452671 | Homo sapiens | China: Henan | 2018 | 18-China_Henan-263 | Ⅰ |
|  | OM452672 | Homo sapiens | China: Henan | 2017 | 17-China_Henan-264 | Ⅱ |
|  | OM452673 | Homo sapiens | China: Henan | 2018 | 18-China_Henan-268 | Ⅳ |
|  | OM452674 | Homo sapiens | China: Henan | 2017 | 17-China_Henan-269 | Ⅰ |
|  | OM452675 | Homo sapiens | China: Henan | 2018 | 18-China_Henan-27 | Ⅰ |
|  | OM452676 | Homo sapiens | China: Henan | 2018 | 18-China_Henan-271 | Ⅲ |
|  | OM452677 | Homo sapiens | China: Henan | 2018 | 18-China_Henan-273 | Ⅰ |
|  | OM452678 | Homo sapiens | China: Henan | 2018 | 18-China_Henan-278 | Ⅱ |
|  | OM452679 | Homo sapiens | China: Henan | 2018 | 18-China_Henan-279 | Ⅰ |
|  | OM452680 | Homo sapiens | China: Henan | 2018 | 18-China_Henan-28 | Ⅱ |
|  | OM452681 | Homo sapiens | China: Henan | 2017 | 17-China_Henan-281 | Ⅰ |
|  | OM452682 | Homo sapiens | China: Henan | 2017 | 17-China_Henan-285 | Ⅰ |
|  | OM452683 | Homo sapiens | China: Henan | 2017 | 17-China_Henan-286 | Ⅰ |
|  | OM452684 | Homo sapiens | China: Henan | 2018 | 18-China_Henan-287 | Ⅱ |
|  | OM452685 | Homo sapiens | China: Henan | 2017 | 17-China_Henan-289 | Ⅳ |
|  | MN510168 | Homo sapiens | China: Henan | 2018 | 18-China_Henan-291 | Ⅳ |
|  | OM452686 | Homo sapiens | China: Henan | 2018 | 18-China_Henan-292 | Ⅱ |
|  | OM452687 | Homo sapiens | China: Henan | 2017 | 17-China_Henan-293 | Ⅰ |
|  | OM452688 | Homo sapiens | China: Henan | 2017 | 17-China_Henan-299 | Ⅱ |
|  | OM452689 | Homo sapiens | China: Henan | 2017 | 17-China_Henan-304 | Ⅱ |
|  | OM452690 | Homo sapiens | China: Henan | 2018 | 18-China_Henan-309 | Ⅰ |
|  | OM452691 | Homo sapiens | China: Henan | 2018 | 18-China_Henan-312 | Ⅰ |
|  | OM452692 | Homo sapiens | China: Henan | 2017 | 17-China_Henan-322 | Ⅳ |
|  | OM452693 | Homo sapiens | China: Henan | 2017 | 17-China_Henan-321 | Ⅰ |
|  | MN510170 | Homo sapiens | China: Henan | 2018 | 18-China_Henan-323 | Ⅰ |
|  | OM452694 | Homo sapiens | China: Henan | 2017 | 17-China_Henan-325 | Ⅰ |
|  | OM452695 | Homo sapiens | China: Henan | 2018 | 18-China_Henan-326 | Ⅰ |
|  | OM452696 | Homo sapiens | China: Henan | 2017 | 17-China_Henan-33 | Ⅰ |
|  | OM452697 | Homo sapiens | China: Henan | 2018 | 18-China_Henan-334 | Ⅰ |
|  | OM452698 | Homo sapiens | China: Henan | 2018 | 18-China_Henan-337 | Ⅰ |
|  | OM452699 | Homo sapiens | China: Henan | 2018 | 18-China_Henan-338 | Ⅰ |
|  | OM452700 | Homo sapiens | China: Henan | 2018 | 18-China_Henan-342 | Ⅰ |
|  | OM452701 | Homo sapiens | China: Henan | 2017 | 17-China_Henan-343 | Ⅰ |
|  | OM452702 | Homo sapiens | China: Henan | 2018 | 18-China_Henan-348 | Ⅰ |
|  | OM452703 | Homo sapiens | China: Henan | 2018 | 18-China_Henan-349 | Ⅰ |
|  | OM452704 | Homo sapiens | China: Henan | 2017 | 17-China_Henan-36 | Ⅰ |
|  | OM452705 | Homo sapiens | China: Henan | 2018 | 18-China_Henan-353 | Ⅰ |
|  | OM452706 | Homo sapiens | China: Henan | 2017 | 17-China_Henan-354 | Ⅰ |
|  | OM452707 | Homo sapiens | China: Henan | 2018 | 18-China_Henan-356 | Ⅰ |
|  | OM452708 | Homo sapiens | China: Henan | 2018 | 18-China_Henan-357 | Ⅲ |
|  | OM452709 | Homo sapiens | China: Hubei | 2018 | 18-China_Hubei-358 | Ⅰ |
|  | OM452710 | Homo sapiens | China: Henan | 2018 | 18-China_Henan-362 | Ⅰ |
|  | OM452711 | Homo sapiens | China: Henan | 2018 | 18-China_Henan-365 | Ⅰ |
|  | OM452712 | Homo sapiens | China: Henan | 2018 | 18-China_Henan-366 | Ⅰ |
|  | OM452713 | Homo sapiens | China: Henan | 2017 | 17-China_Henan-367 | Ⅰ |
|  | OM452714 | Homo sapiens | China: Hubei | 2018 | 18-China_Hubei-368 | Ⅰ |
|  | OM452715 | Homo sapiens | China: Henan | 2018 | 18-China_Henan-37 | Ⅰ |
|  | OM452716 | Homo sapiens | China: Henan | 2018 | 18-China_Henan-40 | Ⅰ |
|  | OM452717 | Homo sapiens | China: Henan | 2017 | 17-China_Henan-42 | Ⅱ |
|  | OM452718 | Homo sapiens | China: Henan | 2018 | 18-China_Henan-43 | Ⅱ |
|  | OM452719 | Homo sapiens | China: Henan | 2018 | 18-China_Henan-44 | Ⅱ |
|  | OM452720 | Homo sapiens | China: Henan | 2017 | 17-China_Henan-47 | Ⅱ |
|  | OM452721 | Homo sapiens | China: Henan | 2018 | 18-China_Henan-50 | Ⅱ |
|  | OM452722 | Homo sapiens | China: Henan | 2018 | 18-China_Henan-51 | Ⅰ |
|  | OM452723 | Homo sapiens | China: Henan | 2018 | 18-China_Henan-53 | Ⅰ |
|  | OM452724 | Homo sapiens | China: Henan | 2017 | 17-China_Henan-54 | Ⅰ |
|  | MN510174 | Homo sapiens | China: Hubei | 2018 | 18-China_Hubei-58 | Ⅲ |
|  | OM452725 | Homo sapiens | China: Henan | 2018 | 18-China_Henan-6 | Ⅰ |
|  | MN510175 | Homo sapiens | China: Henan | 2018 | 18-China_Henan-60 | Ⅰ |
|  | OM452726 | Homo sapiens | China: Henan | 2018 | 18-China_Henan-61 | Ⅲ |
|  | OM452727 | Homo sapiens | China: Henan | 2018 | 18-China_Henan-64 | Ⅰ |
|  | OM452728 | Homo sapiens | China: Henan | 2018 | 18-China_Henan-65 | Ⅲ |
|  | OM452729 | Homo sapiens | China: Henan | 2017 | 17-China_Henan-67 | Ⅰ |
|  | OM452730 | Homo sapiens | China: Henan | 2018 | 18-China_Henan-7 | Ⅱ |
|  | OM452731 | Homo sapiens | China: Henan | 2018 | 18-China_Henan-70 | Ⅰ |
|  | OM452732 | Homo sapiens | China: Henan | 2018 | 18-China_Henan-71 | Ⅰ |
|  | OM452733 | Homo sapiens | China: Henan | 2017 | 17-China_Henan-72 | Ⅰ |
|  | OM452734 | Homo sapiens | China: Henan | 2017 | 17-China_Henan-79 | Ⅰ |
|  | OM452735 | Homo sapiens | China: Henan | 2018 | 18-China_Henan-80 | Ⅰ |
|  | OM452736 | Homo sapiens | China: Henan | 2018 | 18-China_Henan-81 | Ⅳ |
|  | OM452737 | Homo sapiens | China: Henan | 2018 | 18-China_Henan-84 | Ⅰ |
|  | OM452738 | Homo sapiens | China: Henan | 2017 | 17-China_Henan-87 | Ⅱ |
|  | OM452739 | Homo sapiens | China: Henan | 2018 | 18-China_Henan-89 | Ⅲ |
|  | OM452740 | Homo sapiens | China: Henan | 2018 | 18-China_Henan-93 | Ⅱ |
|  | OM452741 | Homo sapiens | China: Henan | 2018 | 18-China_Henan-94 | Ⅰ |
|  | OM452742 | Homo sapiens | China: Henan | 2017 | 17-China_Henan-98 | Ⅰ |
|  | OM452743 | Homo sapiens | China: Henan | 2017 | 17-China_Henan-99 | Ⅰ |
|  | OM452744 | Homo sapiens | China: Henan | 2019 | 19-China_Henan-12 | Ⅰ |
|  | OM452745 | Homo sapiens | China: Hubei | 2019 | 19-China_Hubei-144 | Ⅰ |
|  | OM452746 | Homo sapiens | China: Henan | 2019 | 19-China_Henan-162 | Ⅳ |
|  | OM452747 | Homo sapiens | China: Hubei | 2019 | 19-China_Hubei-167 | Ⅰ |
|  | OM452748 | Homo sapiens | China: Henan | 2019 | 19-China_Henan-186 | Ⅰ |
|  | OM452749 | Homo sapiens | China: Henan | 2019 | 19-China_Henan-19 | Ⅳ |
|  | OM452750 | Homo sapiens | China: Henan | 2019 | 19-China_Henan-193 | Ⅰ |
|  | OM452751 | Homo sapiens | China: Henan | 2019 | 19-China_Henan-231 | Ⅰ |
|  | OM452752 | Homo sapiens | China: Henan | 2019 | 19-China_Henan-233 | Ⅳ |
|  | OM452753 | Homo sapiens | China: Henan | 2019 | 19-China_Henan-234 | Ⅱ |
|  | OM452754 | Homo sapiens | China: Henan | 2019 | 19-China_Henan-237 | Ⅰ |
|  | OM452755 | Homo sapiens | China: Hubei | 2019 | 19-China_Hubei-238 | Ⅲ |
|  | OM452756 | Homo sapiens | China: Henan | 2019 | 19-China_Henan-248 | Ⅰ |
|  | OM452757 | Homo sapiens | China: Henan | 2019 | 19-China_Henan-271 | Ⅱ |
|  | OM452758 | Homo sapiens | China: Henan | 2019 | 19-China_Henan-287 | Ⅱ |
|  | OM452759 | Homo sapiens | China: Henan | 2019 | 19-China_Henan-3 | Ⅰ |
|  | OM452760 | Homo sapiens | China: Henan | 2019 | 19-China_Henan-309 | Ⅰ |
|  | OM452761 | Homo sapiens | China: Henan | 2019 | 19-China_Henan-313 | Ⅰ |
|  | OM452762 | Homo sapiens | China: Henan | 2019 | 19-China_Henan-316 | Ⅱ |
|  | OM452763 | Homo sapiens | China: Henan | 2019 | 19-China_Henan-317 | Ⅰ |
|  | OM452764 | Homo sapiens | China: Henan | 2019 | 19-China_Henan-330 | Ⅰ |
|  | OM452765 | Homo sapiens | China: Hubei | 2019 | 19-China_Hubei-7 | Ⅰ |
|  | OM452766 | Homo sapiens | China: Henan | 2019 | 19-China_Henan-94 | Ⅰ |
|  | OM452767 | Homo sapiens | China: Henan | 2020 | 20-China_Henan-1 | Ⅰ |
|  | OM452768 | Homo sapiens | China: Henan | 2020 | 20-China_Henan-100 | Ⅱ |
|  | OM452769 | Homo sapiens | China: Henan | 2020 | 20-China_Henan-102 | Ⅱ |
|  | OM452770 | Homo sapiens | China: Jiangxi | 2020 | 20-China_Jiangxi-104 | Ⅰ |
|  | OM452771 | Homo sapiens | China: Henan | 2020 | 20-China_Henan-108 | Ⅰ |
|  | OM452772 | Homo sapiens | China: Henan | 2020 | 20-China_Henan-111 | Ⅰ |
|  | OM452773 | Homo sapiens | China: Henan | 2020 | 20-China_Henan-112 | Ⅰ |
|  | OM452774 | Homo sapiens | China: Henan | 2020 | 20-China_Henan-113 | Ⅰ |
|  | OM452775 | Homo sapiens | China: Henan | 2020 | 20-China_Henan-114 | Ⅰ |
|  | OM452776 | Homo sapiens | China: Hubei | 2020 | 20-China_Hubei-116 | Ⅰ |
|  | OM452777 | Homo sapiens | China: Henan | 2020 | 20-China_Henan-118 | Ⅰ |
|  | OM452778 | Homo sapiens | China: Henan | 2020 | 20-China_Henan-120 | Ⅰ |
|  | OM452779 | Homo sapiens | China: Henan | 2020 | 20-China_Henan-122 | Ⅱ |
|  | OM452780 | Homo sapiens | China: Henan | 2020 | 20-China_Henan-126 | Ⅰ |
|  | OM452781 | Homo sapiens | China: Henan | 2020 | 20-China_Henan-13 | Ⅰ |
|  | OM452782 | Homo sapiens | China: Henan | 2020 | 20-China_Henan-130 | Ⅰ |
|  | OM452783 | Homo sapiens | China: Henan | 2020 | 20-China_Henan-133 | Ⅰ |
|  | OM452784 | Homo sapiens | China: Henan | 2020 | 20-China_Henan-134 | Ⅰ |
|  | OM452785 | Homo sapiens | China: Henan | 2020 | 20-China_Henan-137 | Ⅰ |
|  | OM452786 | Homo sapiens | China: Henan | 2020 | 20-China_Henan-138 | Ⅰ |
|  | OM452787 | Homo sapiens | China: Henan | 2020 | 20-China_Henan-14 | Ⅵ |
|  | OM452788 | Homo sapiens | China: Henan | 2020 | 20-China_Henan-140 | Ⅳ |
|  | OM452789 | Homo sapiens | China: Henan | 2020 | 20-China_Henan-142 | Ⅱ |
|  | OM452790 | Homo sapiens | China: Hubei | 2020 | 20-China_Hubei-143 | Ⅰ |
|  | OM452791 | Homo sapiens | China: Henan | 2020 | 20-China_Henan-144 | Ⅱ |
|  | OM452792 | Homo sapiens | China: Henan | 2020 | 20-China_Henan-145 | Ⅱ |
|  | OM452793 | Homo sapiens | China: Henan | 2020 | 20-China_Henan-149 | Ⅳ |
|  | OM452794 | Homo sapiens | China: Henan | 2020 | 20-China_Henan-15 | Ⅰ |
|  | OM452795 | Homo sapiens | China: Henan | 2020 | 20-China_Henan-153 | Ⅰ |
|  | OM452796 | Homo sapiens | China: Henan | 2020 | 20-China_Henan-156 | Ⅱ |
|  | OM452797 | Homo sapiens | China: Henan | 2020 | 20-China_Henan-157 | Ⅰ |
|  | OM452798 | Homo sapiens | China: Henan | 2020 | 20-China_Henan-158 | Ⅱ |
|  | OM452799 | Homo sapiens | China: Henan | 2020 | 20-China_Henan-168 | Ⅰ |
|  | OM452800 | Homo sapiens | China: Henan | 2020 | 20-China_Henan-175 | Ⅰ |
|  | OM452801 | Homo sapiens | China: Henan | 2020 | 20-China_Henan-177 | Ⅱ |
|  | OM452802 | Homo sapiens | China: Henan | 2020 | 20-China_Henan-178 | Ⅱ |
|  | OM452803 | Homo sapiens | China: Henan | 2020 | 20-China_Henan-180 | Ⅱ |
|  | OM452804 | Homo sapiens | China: Henan | 2020 | 20-China_Henan-181 | Ⅰ |
|  | OM452805 | Homo sapiens | China: Henan | 2020 | 20-China_Henan-182 | Ⅰ |
|  | OM452806 | Homo sapiens | China: Henan | 2020 | 20-China_Henan-183 | Ⅰ |
|  | OM452807 | Homo sapiens | China: Henan | 2020 | 20-China_Henan-189 | Ⅰ |
|  | OM452808 | Homo sapiens | China: Henan | 2020 | 20-China_Henan-190 | Ⅱ |
|  | OM452809 | Homo sapiens | China: Hubei | 2020 | 20-China_Hubei-191 | Ⅲ |
|  | OM452810 | Homo sapiens | China: Henan | 2020 | 20-China_Henan-192 | Ⅰ |
|  | OM452811 | Homo sapiens | China: Henan | 2020 | 20-China_Henan-198 | Ⅰ |
|  | OM452812 | Homo sapiens | China: Henan | 2020 | 20-China_Henan-20 | Ⅱ |
|  | OM452813 | Homo sapiens | China: Henan | 2020 | 20-China_Henan-200 | Ⅰ |
|  | OM452814 | Homo sapiens | China: Hubei | 2020 | 20-China_Hubei-201 | Ⅰ |
|  | OM452815 | Homo sapiens | China: Henan | 2020 | 20-China_Henan-203 | Ⅱ |
|  | OM452816 | Homo sapiens | China: Henan | 2020 | 20-China_Henan-209 | Ⅰ |
|  | OM452817 | Homo sapiens | China: Henan | 2020 | 20-China_Henan-210 | Ⅱ |
|  | OM452818 | Homo sapiens | China: Henan | 2020 | 20-China_Henan-211 | Ⅰ |
|  | OM452819 | Homo sapiens | China: Henan | 2020 | 20-China_Henan-215 | Ⅳ |
|  | OM452820 | Homo sapiens | China: Henan | 2020 | 20-China_Henan-219 | Ⅰ |
|  | OM452821 | Homo sapiens | China: Henan | 2020 | 20-China_Henan-222 | Ⅰ |
|  | OM452822 | Homo sapiens | China: Henan | 2020 | 20-China_Henan-223 | Ⅱ |
|  | OM452823 | Homo sapiens | China: Hubei | 2020 | 20-China_Hubei-228 | Ⅰ |
|  | OM452824 | Homo sapiens | China: Henan | 2020 | 20-China_Henan-230 | Ⅱ |
|  | OM452825 | Homo sapiens | China: Hubei | 2020 | 20-China_Hubei-233 | Ⅱ |
|  | OM452826 | Homo sapiens | China: Henan | 2020 | 20-China_Henan-234 | Ⅰ |
|  | OM452827 | Homo sapiens | China: Henan | 2020 | 20-China_Henan-236 | Ⅰ |
|  | OM452828 | Homo sapiens | China: Henan | 2020 | 20-China_Henan-238 | Ⅱ |
|  | OM452829 | Homo sapiens | China: Henan | 2020 | 20-China_Henan-24 | Ⅰ |
|  | OM452830 | Homo sapiens | China: Hubei | 2020 | 20-China_Hubei-240 | Ⅰ |
|  | OM452831 | Homo sapiens | China: Henan | 2020 | 20-China_Henan-244 | Ⅰ |
|  | OM452832 | Homo sapiens | China: Henan | 2020 | 20-China_Henan-246 | Ⅰ |
|  | OM452833 | Homo sapiens | China: Henan | 2020 | 20-China_Henan-249 | Ⅰ |
|  | OM452834 | Homo sapiens | China: Henan | 2020 | 20-China_Henan-252 | Ⅱ |
|  | OM452835 | Homo sapiens | China: Henan | 2020 | 20-China_Henan-254 | Ⅲ |
|  | OM452836 | Homo sapiens | China: Henan | 2020 | 20-China_Henan-255 | Ⅲ |
|  | OM452837 | Homo sapiens | China: Henan | 2020 | 20-China_Henan-28 | Ⅳ |
|  | OM452838 | Homo sapiens | China: Henan | 2020 | 20-China_Henan-3 | Ⅰ |
|  | OM452839 | Homo sapiens | China: Henan | 2020 | 20-China_Henan-37 | Ⅰ |
|  | OM452840 | Homo sapiens | China: Henan | 2020 | 20-China_Henan-4 | Ⅰ |
|  | OM452841 | Homo sapiens | China: Henan | 2020 | 20-China_Henan-40 | Ⅰ |
|  | OM452842 | Homo sapiens | China: Henan | 2020 | 20-China_Henan-41 | Ⅰ |
|  | OM452843 | Homo sapiens | China: Henan | 2020 | 20-China_Henan-42 | Ⅰ |
|  | OM452844 | Homo sapiens | China: Henan | 2020 | 20-China_Henan-43 | Ⅰ |
|  | OM452845 | Homo sapiens | China: Henan | 2020 | 20-China_Henan-48 | Ⅰ |
|  | OM452846 | Homo sapiens | China: Henan | 2020 | 20-China_Henan-49 | Ⅰ |
|  | OM452847 | Homo sapiens | China: Henan | 2020 | 20-China_Henan-51 | Ⅰ |
|  | OM452848 | Homo sapiens | China: Hubei | 2020 | 20-China_Hubei-54 | Ⅰ |
|  | OM452849 | Homo sapiens | China: Henan | 2020 | 20-China_Henan-59 | Ⅰ |
|  | OM452850 | Homo sapiens | China: Henan | 2020 | 20-China_Henan-61 | Ⅳ |
|  | OM452851 | Homo sapiens | China: Henan | 2020 | 20-China_Henan-65 | Ⅰ |
|  | OM452852 | Homo sapiens | China: Henan | 2020 | 20-China_Henan-67 | Ⅰ |
|  | OM452853 | Homo sapiens | China: Henan | 2020 | 20-China_Henan-73 | Ⅰ |
|  | OM452854 | Homo sapiens | China: Henan | 2020 | 20-China_Henan-75 | Ⅳ |
|  | OM452855 | Homo sapiens | China: Henan | 2020 | 20-China_Henan-77 | Ⅰ |
|  | OM452856 | Homo sapiens | China: Henan | 2020 | 20-China_Henan-78 | Ⅰ |
|  | OM452857 | Homo sapiens | China: Henan | 2020 | 20-China_Henan-81 | Ⅱ |
|  | OM452858 | Homo sapiens | China: Henan | 2020 | 20-China_Henan-86 | Ⅰ |
|  | OM452859 | Homo sapiens | China: Henan | 2020 | 20-China_Henan-88 | Ⅰ |
|  | OM452860 | Homo sapiens | China: Henan | 2020 | 20-China_Henan-90 | Ⅰ |
|  | OM452861 | Homo sapiens | China: Hubei | 2020 | 20-China_Hubei-92 | Ⅰ |
|  | OM452862 | Homo sapiens | China: Henan | 2020 | 20-China_Henan-93 | Ⅰ |
|  | OM452863 | Homo sapiens | China: Henan | 2020 | 20-China_Henan-99 | Ⅱ |
|  | OM452864 | Homo sapiens | China: Henan | 2019 | 19-China_Henan-126 | Ⅳ |
|  | OM452865 | Homo sapiens | China: Henan | 2019 | 19-China_Henan-129 | Ⅱ |
|  | OM452866 | Homo sapiens | China: Henan | 2019 | 19-China_Henan-132 | Ⅰ |
|  | OM452867 | Homo sapiens | China: Henan | 2019 | 19-China_Henan-133 | Ⅰ |
|  | OM452868 | Homo sapiens | China: Henan | 2019 | 19-China_Henan-137 | Ⅰ |
|  | OM452869 | Homo sapiens | China: Henan | 2019 | 19-China_Henan-139 | Ⅰ |
|  | OM452870 | Homo sapiens | China: Henan | 2019 | 19-China_Henan-148 | Ⅰ |
|  | OM452871 | Homo sapiens | China: Henan | 2019 | 19-China_Henan-156 | Ⅱ |
|  | OM452872 | Homo sapiens | China: Henan | 2019 | 19-China_Henan-160 | Ⅰ |
|  | OM452873 | Homo sapiens | China: Hubei | 2019 | 19-China_Hubei-166 | Ⅲ |
|  | OM452874 | Homo sapiens | China: Henan | 2019 | 19-China_Henan-173 | Ⅱ |
|  | OM452875 | Homo sapiens | China: Hubei | 2019 | 19-China_Hubei-174 | Ⅰ |
|  | OM452876 | Homo sapiens | China: Henan | 2019 | 19-China_Henan-175 | Ⅰ |
|  | OM452877 | Homo sapiens | China: Henan | 2019 | 19-China_Henan-177 | Ⅰ |
|  | OM452878 | Homo sapiens | China: Henan | 2019 | 19-China_Henan-179 | Ⅱ |
|  | OM452879 | Homo sapiens | China: Henan | 2019 | 19-China_Henan-183 | Ⅰ |
|  | OM452880 | Homo sapiens | China: Hubei | 2019 | 19-China_Hubei-185 | Ⅰ |
|  | OM452881 | Homo sapiens | China: Henan | 2019 | 19-China_Henan-189 | Ⅰ |
|  | OM452882 | Homo sapiens | China: Hubei | 2019 | 19-China_Hubei-195 | Ⅰ |
|  | OM452883 | Homo sapiens | China: Henan | 2019 | 19-China_Henan-197 | Ⅰ |
|  | OM452884 | Homo sapiens | China: Henan | 2019 | 19-China_Henan-198 | Ⅰ |
|  | OM452885 | Homo sapiens | China: Henan | 2019 | 19-China_Henan-199 | Ⅰ |
|  | OM452886 | Homo sapiens | China: Henan | 2019 | 19-China_Henan-201 | Ⅰ |
|  | OM452887 | Homo sapiens | China: Henan | 2019 | 19-China_Henan-208 | Ⅰ |
|  | OM452888 | Homo sapiens | China: Henan | 2019 | 19-China_Henan-214 | Ⅰ |
|  | OM452889 | Homo sapiens | China: Henan | 2019 | 19-China_Henan-215 | Ⅱ |
|  | OM452890 | Homo sapiens | China: Henan | 2019 | 19-China_Henan-217 | Ⅰ |
|  | OM452891 | Homo sapiens | China: Henan | 2019 | 19-China_Henan-223 | Ⅱ |
|  | OM452892 | Homo sapiens | China: Henan | 2019 | 19-China_Henan-227 | Ⅱ |
|  | OM452893 | Homo sapiens | China: Henan | 2019 | 19-China_Henan-232 | Ⅰ |
|  | OM452894 | Homo sapiens | China: Henan | 2019 | 19-China_Henan-235 | Ⅰ |
|  | OM452895 | Homo sapiens | China: Henan | 2019 | 19-China_Henan-236 | Ⅰ |
|  | OM452896 | Homo sapiens | China: Hubei | 2019 | 19-China_Hubei-240 | Ⅰ |
|  | OM452897 | Homo sapiens | China: Henan | 2019 | 19-China_Henan-241 | Ⅰ |
|  | OM452898 | Homo sapiens | China: Henan | 2019 | 19-China_Henan-242 | Ⅰ |
|  | OM452899 | Homo sapiens | China: Henan | 2019 | 19-China_Henan-243 | Ⅰ |
|  | OM452900 | Homo sapiens | China: Hubei | 2019 | 19-China_Hubei-249 | Ⅰ |
|  | OM452901 | Homo sapiens | China: Henan | 2019 | 19-China_Henan-253 | Ⅰ |
|  | OM452902 | Homo sapiens | China: Henan | 2019 | 19-China_Henan-254 | Ⅰ |
|  | OM452903 | Homo sapiens | China: Henan | 2019 | 19-China_Henan-259 | Ⅰ |
|  | OM452904 | Homo sapiens | China: Henan | 2019 | 19-China_Henan-260 | Ⅰ |
|  | OM452905 | Homo sapiens | China: Henan | 2019 | 19-China_Henan-261 | Ⅰ |
|  | OM452906 | Homo sapiens | China: Henan | 2019 | 19-China_Henan-262 | Ⅰ |
|  | OM452907 | Homo sapiens | China: Hubei | 2019 | 19-China_Hubei-263 | Ⅰ |
|  | OM452908 | Homo sapiens | China: Henan | 2019 | 19-China_Henan-264 | Ⅰ |
|  | OM452909 | Homo sapiens | China: Henan | 2019 | 19-China_Henan-266 | Ⅰ |
|  | OM452910 | Homo sapiens | China: Henan | 2019 | 19-China_Henan-268 | Ⅰ |
|  | OM452911 | Homo sapiens | China: Henan | 2019 | 19-China_Henan-272 | Ⅰ |
|  | OM452912 | Homo sapiens | China: Henan | 2019 | 19-China_Henan-273 | Ⅰ |
|  | OM452913 | Homo sapiens | China: Henan | 2019 | 19-China_Henan-274 | Ⅳ |
|  | OM452914 | Homo sapiens | China: Henan | 2019 | 19-China_Henan-278 | Ⅰ |
|  | OM452915 | Homo sapiens | China: Hubei | 2019 | 19-China_Hubei-279 | Ⅰ |
|  | OM452916 | Homo sapiens | China: Henan | 2019 | 19-China_Henan-280 | Ⅱ |
|  | OM452917 | Homo sapiens | China: Henan | 2019 | 19-China_Henan-285 | Ⅰ |
|  | OM452918 | Homo sapiens | China: Hubei | 2019 | 19-China_Hubei-291 | Ⅲ |
|  | OM452919 | Homo sapiens | China: Henan | 2019 | 19-China_Henan-295 | Ⅳ |
|  | OM452920 | Homo sapiens | China: Hubei | 2019 | 19-China_Hubei-297 | Ⅰ |
|  | OM452921 | Homo sapiens | China: Henan | 2019 | 19-China_Henan-298 | Ⅰ |
|  | OM452922 | Homo sapiens | China: Hubei | 2019 | 19-China_Hubei-299 | Ⅰ |
|  | OM452923 | Homo sapiens | China: Henan | 2019 | 19-China_Henan-301 | Ⅰ |
|  | OM452924 | Homo sapiens | China: Henan | 2019 | 19-China_Henan-302 | Ⅰ |
|  | OM452925 | Homo sapiens | China: Henan | 2019 | 19-China_Henan-304 | Ⅰ |
|  | OM452926 | Homo sapiens | China: Henan | 2019 | 19-China_Henan-305 | Ⅰ |
|  | OM452927 | Homo sapiens | China: Hubei | 2019 | 19-China_Hubei-306 | Ⅰ |
|  | OM452928 | Homo sapiens | China: Henan | 2019 | 19-China_Henan-308 | Ⅱ |
|  | OM452929 | Homo sapiens | China: Hubei | 2019 | 19-China_Hubei-310 | Ⅰ |
|  | OM452930 | Homo sapiens | China: Hubei | 2019 | 19-China_Hubei-314 | Ⅰ |
|  | OM452931 | Homo sapiens | China: Henan | 2019 | 19-China_Henan-321 | Ⅱ |
|  | OM452932 | Homo sapiens | China: Henan | 2019 | 19-China_Henan-322 | Ⅰ |
|  | OM452933 | Homo sapiens | China: Henan | 2019 | 19-China_Henan-323 | Ⅰ |
|  | OM452934 | Homo sapiens | China: Henan | 2019 | 19-China_Henan-324 | Ⅳ |
|  | OM452935 | Homo sapiens | China: Hubei | 2019 | 19-China_Hubei-327 | Ⅲ |
|  | OM452936 | Homo sapiens | China: Henan | 2019 | 19-China_Henan-358 | Ⅰ |
|  | OM452937 | Homo sapiens | China: Hubei | 2019 | 19-China_Hubei-377 | Ⅰ |
|  | OM452938 | Homo sapiens | China: Henan | 2019 | 19-China_Henan-382 | Ⅳ |
|  | OM452939 | Homo sapiens | China: Henan | 2019 | 19-China_Henan-388 | Ⅰ |
|  | OM452940 | Homo sapiens | China: Henan | 2020 | 20-China_Henan-12 | Ⅳ |
|  | OM452941 | Homo sapiens | China: Henan | 2020 | 20-China_Henan-115 | Ⅳ |
|  | OM452942 | Homo sapiens | China: Henan | 2020 | 20-China_Henan-139 | Ⅳ |
| L | MN509835 | Homo sapiens | China: Henan | 2011 | 11-China_Henan-1 | Ⅰ |
|  | MN509837 | Homo sapiens | China: Henan | 2011 | 11-China_Henan-13 | Ⅰ |
|  | MN509836 | Homo sapiens | China: Henan | 2011 | 11-China_Henan-2 | Ⅳ |
|  | MN509848 | Homo sapiens | China: Henan | 2011 | 11-China_Henan-266 | Ⅱ |
|  | MN509849 | Homo sapiens | China: Henan | 2011 | 11-China_Henan-273 | Ⅰ |
|  | MN509858 | Homo sapiens | China: Henan | 2012 | 12-China_Henan-103 | Ⅰ |
|  | OM452943 | Homo sapiens | China: Henan | 2012 | 12-China_Henan-108 | Ⅱ |
|  | MN509859 | Homo sapiens | China: Henan | 2012 | 12-China_Henan-109 | Ⅳ |
|  | MN509860 | Homo sapiens | China: Henan | 2012 | 12-China_Henan-110 | Ⅰ |
|  | MN509861 | Homo sapiens | China: Henan | 2012 | 12-China_Henan-114 | Ⅳ |
|  | OM452944 | Homo sapiens | China: Henan | 2012 | 12-China_Henan-127 | Ⅱ |
|  | OM452945 | Homo sapiens | China: Henan | 2012 | 12-China_Henan-13 | Ⅰ |
|  | MN509863 | Homo sapiens | China: Anhui | 2012 | 12-China_Anhui-154 | Ⅰ |
|  | OM452946 | Homo sapiens | China: Henan | 2012 | 12-China_Henan-194 | Ⅰ |
|  | OM452947 | Homo sapiens | China: Hubei | 2012 | 12-China_Hubei-21 | Ⅱ |
|  | OM452948 | Homo sapiens | China: Henan | 2012 | 12-China_Henan-220 | Ⅰ |
|  | OM452949 | Homo sapiens | China: Henan | 2012 | 12-China_Henan-224 | Ⅰ |
|  | MN509851 | Homo sapiens | China: Henan | 2012 | 12-China_Henan-23 | Ⅰ |
|  | MN509864 | Homo sapiens | China: Henan | 2012 | 12-China_Henan-234 | Ⅰ |
|  | OM452950 | Homo sapiens | China: Henan | 2012 | 12-China_Henan-237 | Ⅰ |
|  | OM452951 | Homo sapiens | China: Hebei | 2012 | 12-China_Hebei-24 | Ⅰ |
|  | MN509865 | Homo sapiens | China: Hubei | 2012 | 12-China_Hubei-242 | Ⅰ |
|  | MN509867 | Homo sapiens | China: Henan | 2012 | 12-China_Henan-270 | Ⅰ |
|  | MN509868 | Homo sapiens | China: Henan | 2012 | 12-China_Henan-271 | Ⅰ |
|  | OM452952 | Homo sapiens | China: Henan | 2012 | 12-China_Henan-279 | Ⅱ |
|  | OM452953 | Homo sapiens | China: Henan | 2012 | 12-China_Henan-30 | Ⅱ |
|  | OM452954 | Homo sapiens | China: Henan | 2012 | 12-China_Henan-32 | Ⅰ |
|  | OM452955 | Homo sapiens | China: Henan | 2012 | 12-China_Henan-324 | Ⅱ |
|  | OM452956 | Homo sapiens | China: Henan | 2012 | 12-China_Henan-36 | Ⅰ |
|  | OM452957 | Homo sapiens | China: Henan | 2012 | 12-China_Henan-37 | Ⅳ |
|  | OM452958 | Homo sapiens | China: Henan | 2012 | 12-China_Henan-40 | Ⅳ |
|  | OM452959 | Homo sapiens | China: Henan | 2012 | 12-China_Henan-41 | Ⅰ |
|  | MN509852 | Homo sapiens | China: Henan | 2012 | 12-China_Henan-48 | Ⅰ |
|  | OM452960 | Homo sapiens | China: Henan | 2012 | 12-China_Henan-5 | Ⅱ |
|  | MN509855 | Homo sapiens | China: Henan | 2012 | 12-China_Henan-54 | Ⅰ |
|  | OM452961 | Homo sapiens | China: Henan | 2012 | 12-China_Henan-56 | Ⅱ |
|  | OM452962 | Homo sapiens | China: Henan | 2012 | 12-China_Henan-64 | Ⅰ |
|  | OM452963 | Homo sapiens | China: Henan | 2012 | 12-China_Henan-65 | Ⅰ |
|  | OM452964 | Homo sapiens | China: Henan | 2012 | 12-China_Henan-7 | Ⅰ |
|  | OM452965 | Homo sapiens | China: Henan | 2012 | 12-China_Henan-9 | Ⅰ |
|  | OM452966 | Homo sapiens | China: Henan | 2012 | 12-China_Henan-90 | Ⅶ |
|  | OM452967 | Homo sapiens | China: Henan | 2013 | 13-China_Henan-100 | Ⅰ |
|  | OM452968 | Homo sapiens | China: Henan | 2013 | 13-China_Henan-101 | Ⅰ |
|  | MN509876 | Homo sapiens | China: Henan | 2013 | 13-China_Henan-110 | Ⅰ |
|  | OM452969 | Homo sapiens | China: Henan | 2013 | 13-China_Henan-112 | Ⅱ |
|  | MN509877 | Homo sapiens | China: Hubei | 2013 | 13-China_Hubei-113 | Ⅰ |
|  | OM452970 | Homo sapiens | China: Henan | 2013 | 13-China_Henan-116 | Ⅰ |
|  | OM452971 | Homo sapiens | China: Henan | 2013 | 13-China_Henan-118 | Ⅰ |
|  | OM452972 | Homo sapiens | China: Henan | 2013 | 13-China_Henan-119 | Ⅰ |
|  | OM452973 | Homo sapiens | China: Henan | 2013 | 13-China_Henan-120 | Ⅰ |
|  | OM452974 | Homo sapiens | China: Henan | 2013 | 13-China_Henan-122 | Ⅱ |
|  | MN509878 | Homo sapiens | China: Hubei | 2013 | 13-China_Hubei-124 | Ⅰ |
|  | OM452975 | Homo sapiens | China: Henan | 2013 | 13-China_Henan-126 | Ⅳ |
|  | MN509880 | Homo sapiens | China: Henan | 2013 | 13-China_Henan-128 | Ⅰ |
|  | OM452976 | Homo sapiens | China: Henan | 2013 | 13-China_Henan-129 | Ⅰ |
|  | OM452977 | Homo sapiens | China: Henan | 2013 | 13-China_Henan-130 | Ⅰ |
|  | OM452978 | Homo sapiens | China: Henan | 2013 | 13-China_Henan-132 | Ⅰ |
|  | OM452979 | Homo sapiens | China: Henan | 2013 | 13-China_Henan-139 | Ⅰ |
|  | OM452980 | Homo sapiens | China: Henan | 2013 | 13-China_Henan-141 | Ⅱ |
|  | OM452981 | Homo sapiens | China: Henan | 2013 | 13-China_Henan-142 | Ⅰ |
|  | OM452982 | Homo sapiens | China: Henan | 2013 | 13-China_Henan-144 | Ⅰ |
|  | MN509881 | Homo sapiens | China: Henan | 2013 | 13-China_Henan-147 | Ⅰ |
|  | OM452983 | Homo sapiens | China: Henan | 2013 | 13-China_Henan-154 | Ⅱ |
|  | OM452984 | Homo sapiens | China: Henan | 2013 | 13-China_Henan-155 | Ⅰ |
|  | MN509882 | Homo sapiens | China: Henan | 2013 | 13-China_Henan-160 | Ⅰ |
|  | OM452985 | Homo sapiens | China: Henan | 2013 | 13-China_Henan-166 | Ⅰ |
|  | OM452986 | Homo sapiens | China: Henan | 2013 | 13-China_Henan-170 | Ⅰ |
|  | OM452987 | Homo sapiens | China: Henan | 2013 | 13-China_Henan-177 | Ⅰ |
|  | MN509883 | Homo sapiens | China: Henan | 2013 | 13-China_Henan-178 | Ⅰ |
|  | OM452988 | Homo sapiens | China: Henan | 2013 | 13-China_Henan-181 | Ⅰ |
|  | OM452989 | Homo sapiens | China: Henan | 2013 | 13-China_Henan-192 | Ⅰ |
|  | OM452990 | Homo sapiens | China: Henan | 2013 | 13-China_Henan-195 | Ⅱ |
|  | OM452991 | Homo sapiens | China: Henan | 2013 | 13-China_Henan-198 | Ⅲ |
|  | OM452992 | Homo sapiens | China: Henan | 2013 | 13-China_Henan-200 | Ⅰ |
|  | OM452993 | Homo sapiens | China: Henan | 2013 | 13-China_Henan-201 | Ⅰ |
|  | MN509885 | Homo sapiens | China: Henan | 2013 | 13-China_Henan-212 | Ⅰ |
|  | OM452994 | Homo sapiens | China: Henan | 2013 | 13-China_Henan-213 | Ⅳ |
|  | OM452995 | Homo sapiens | China: Henan | 2013 | 13-China_Henan-219 | Ⅰ |
|  | OM452996 | Homo sapiens | China: Henan | 2013 | 13-China_Henan-221 | Ⅰ |
|  | OM452997 | Homo sapiens | China: Henan | 2013 | 13-China_Henan-224 | Ⅰ |
|  | OM452998 | Homo sapiens | China: Henan | 2013 | 13-China_Henan-229 | Ⅱ |
|  | OM452999 | Homo sapiens | China: Henan | 2013 | 13-China_Henan-238 | Ⅱ |
|  | OM453000 | Homo sapiens | China: Henan | 2013 | 13-China_Henan-24 | Ⅰ |
|  | OM453001 | Homo sapiens | China: Henan | 2013 | 13-China_Henan-240 | Ⅰ |
|  | OM453002 | Homo sapiens | China: Henan | 2013 | 13-China_Henan-246 | Ⅱ |
|  | MN509887 | Homo sapiens | China: Hubei | 2013 | 13-China_Hubei-252 | Ⅰ |
|  | OM453003 | Homo sapiens | China: Henan | 2013 | 13-China_Henan-254 | Ⅰ |
|  | MN509888 | Homo sapiens | China: Henan | 2013 | 13-China_Henan-255 | Ⅰ |
|  | MN509889 | Homo sapiens | China: Hubei | 2013 | 13-China_Hubei-256 | Ⅲ |
|  | OM453004 | Homo sapiens | China: Henan | 2013 | 13-China_Henan-258 | Ⅰ |
|  | OM453005 | Homo sapiens | China: Henan | 2013 | 13-China_Henan-262 | Ⅳ |
|  | MN509890 | Homo sapiens | China: Henan | 2013 | 13-China_Henan-263 | Ⅰ |
|  | OM453006 | Homo sapiens | China: Henan | 2013 | 13-China_Henan-264 | Ⅰ |
|  | OM453007 | Homo sapiens | China: Henan | 2013 | 13-China_Henan-266 | Ⅳ |
|  | OM453008 | Homo sapiens | China: Henan | 2013 | 13-China_Henan-271 | Ⅰ |
|  | OM453009 | Homo sapiens | China: Henan | 2013 | 13-China_Henan-275 | Ⅱ |
|  | OM453010 | Homo sapiens | China: Henan | 2013 | 13-China_Henan-279 | Ⅳ |
|  | OM453011 | Homo sapiens | China: Henan | 2013 | 13-China_Henan-280 | Ⅰ |
|  | MN509891 | Homo sapiens | China: Henan | 2013 | 13-China_Henan-288 | Ⅰ |
|  | OM453012 | Homo sapiens | China: Henan | 2013 | 13-China_Henan-29 | Ⅰ |
|  | OM453013 | Homo sapiens | China: Henan | 2013 | 13-China_Henan-292 | Ⅱ |
|  | OM453014 | Homo sapiens | China: Henan | 2013 | 13-China_Henan-293 | Ⅱ |
|  | OM453015 | Homo sapiens | China: Henan | 2013 | 13-China_Henan-294 | Ⅰ |
|  | OM453016 | Homo sapiens | China: Henan | 2013 | 13-China_Henan-295 | Ⅱ |
|  | OM453017 | Homo sapiens | China: Henan | 2013 | 13-China_Henan-297 | Ⅳ |
|  | OM453018 | Homo sapiens | China: Henan | 2013 | 13-China_Henan-299 | Ⅳ |
|  | OM453019 | Homo sapiens | China: Henan | 2013 | 13-China_Henan-30 | Ⅰ |
|  | OM453020 | Homo sapiens | China: Henan | 2013 | 13-China_Henan-300 | Ⅱ |
|  | MN509892 | Homo sapiens | China: Henan | 2013 | 13-China_Henan-302 | Ⅰ |
|  | OM453021 | Homo sapiens | China: Henan | 2013 | 13-China_Henan-304 | Ⅰ |
|  | OM453022 | Homo sapiens | China: Henan | 2013 | 13-China_Henan-31 | Ⅱ |
|  | OM453023 | Homo sapiens | China: Henan | 2013 | 13-China_Henan-310 | Ⅰ |
|  | OM453024 | Homo sapiens | China: Henan | 2013 | 13-China_Henan-311 | Ⅰ |
|  | OM453025 | Homo sapiens | China: Henan | 2013 | 13-China_Henan-313 | Ⅱ |
|  | OM453026 | Homo sapiens | China: Henan | 2013 | 13-China_Henan-320 | Ⅰ |
|  | MN509894 | Homo sapiens | China: Hubei | 2013 | 13-China_Hubei-321 | Ⅰ |
|  | OM453027 | Homo sapiens | China: Henan | 2013 | 13-China_Henan-324 | Ⅱ |
|  | OM453028 | Homo sapiens | China: Henan | 2013 | 13-China_Henan-357 | Ⅰ |
|  | OM453029 | Homo sapiens | China: Henan | 2013 | 13-China_Henan-364 | Ⅲ |
|  | OM453030 | Homo sapiens | China: Henan | 2013 | 13-China_Henan-368 | Ⅰ |
|  | MN509895 | Homo sapiens | China: Henan | 2013 | 13-China_Henan-369 | Ⅰ |
|  | OM453031 | Homo sapiens | China: Henan | 2013 | 13-China_Henan-37 | Ⅰ |
|  | OM453032 | Homo sapiens | China: Henan | 2013 | 13-China_Henan-371 | Ⅳ |
|  | OM453033 | Homo sapiens | China: Henan | 2013 | 13-China_Henan-38 | Ⅱ |
|  | MN509896 | Homo sapiens | China: Henan | 2013 | 13-China_Henan-382 | Ⅳ |
|  | MN509871 | Homo sapiens | China: Henan | 2013 | 13-China_Henan-39 | Ⅰ |
|  | MN509898 | Homo sapiens | China: Henan | 2013 | 13-China_Henan-392 | Ⅳ |
|  | OM453034 | Homo sapiens | China: Henan | 2013 | 13-China_Henan-398 | Ⅱ |
|  | MN509872 | Homo sapiens | China: Henan | 2013 | 13-China_Henan-40 | Ⅰ |
|  | OM453035 | Homo sapiens | China: Henan | 2013 | 13-China_Henan-408 | Ⅰ |
|  | OM453036 | Homo sapiens | China: Henan | 2013 | 13-China_Henan-420 | Ⅰ |
|  | OM453037 | Homo sapiens | China: Henan | 2013 | 13-China_Henan-423 | Ⅳ |
|  | OM453038 | Homo sapiens | China: Henan | 2013 | 13-China_Henan-424 | Ⅰ |
|  | OM453039 | Homo sapiens | China: Henan | 2013 | 13-China_Henan-425 | Ⅰ |
|  | OM453040 | Homo sapiens | China: Henan | 2013 | 13-China_Henan-428 | Ⅰ |
|  | OM453041 | Homo sapiens | China: Henan | 2013 | 13-China_Henan-429 | Ⅱ |
|  | OM453042 | Homo sapiens | China: Henan | 2013 | 13-China_Henan-44 | Ⅰ |
|  | OM453043 | Homo sapiens | China: Henan | 2013 | 13-China_Henan-45 | Ⅱ |
|  | MN509873 | Homo sapiens | China: Henan | 2013 | 13-China_Henan-48 | Ⅰ |
|  | OM453044 | Homo sapiens | China: Henan | 2013 | 13-China_Henan-50 | Ⅰ |
|  | OM453045 | Homo sapiens | China: Henan | 2013 | 13-China_Henan-52 | Ⅰ |
|  | OM453046 | Homo sapiens | China: Henan | 2013 | 13-China_Henan-53 | Ⅰ |
|  | OM453047 | Homo sapiens | China: Henan | 2013 | 13-China_Henan-54 | Ⅱ |
|  | OM453048 | Homo sapiens | China: Henan | 2013 | 13-China_Henan-55 | Ⅱ |
|  | OM453049 | Homo sapiens | China: Henan | 2013 | 13-China_Henan-60 | Ⅱ |
|  | OM453050 | Homo sapiens | China: Henan | 2013 | 13-China_Henan-62 | Ⅰ |
|  | OM453051 | Homo sapiens | China: Henan | 2013 | 13-China_Henan-65 | Ⅰ |
|  | OM453052 | Homo sapiens | China: Henan | 2013 | 13-China_Henan-67 | Ⅰ |
|  | OM453053 | Homo sapiens | China: Henan | 2013 | 13-China_Henan-68 | Ⅰ |
|  | OM453054 | Homo sapiens | China: Henan | 2013 | 13-China_Henan-77 | Ⅰ |
|  | OM453055 | Homo sapiens | China: Henan | 2013 | 13-China_Henan-78 | Ⅰ |
|  | MN509874 | Homo sapiens | China: Hubei | 2013 | 13-China_Hubei-79 | Ⅲ |
|  | OM453056 | Homo sapiens | China: Henan | 2013 | 13-China_Henan-86 | Ⅱ |
|  | OM453057 | Homo sapiens | China: Henan | 2013 | 13-China_Henan-87 | Ⅱ |
|  | OM453058 | Homo sapiens | China: Henan | 2013 | 13-China_Henan-91 | Ⅱ |
|  | OM453059 | Homo sapiens | China: Henan | 2013 | 13-China_Henan-93 | Ⅰ |
|  | OM453060 | Homo sapiens | China: Henan | 2013 | 13-China_Henan-94 | Ⅱ |
|  | OM453061 | Homo sapiens | China: Henan | 2014 | 14-China_Henan-107 | Ⅰ |
|  | OM453062 | Homo sapiens | China: Henan | 2014 | 14-China_Henan-109 | Ⅱ |
|  | OM453063 | Homo sapiens | China: Henan | 2014 | 14-China_Henan-11 | Ⅰ |
|  | OM453064 | Homo sapiens | China: Henan | 2014 | 14-China_Henan-118 | Ⅰ |
|  | OM453065 | Homo sapiens | China: Henan | 2014 | 14-China_Henan-129 | Ⅱ |
|  | OM453066 | Homo sapiens | China: Henan | 2014 | 14-China_Henan-13 | Ⅰ |
|  | OM453067 | Homo sapiens | China: Henan | 2014 | 14-China_Henan-134 | Ⅳ |
|  | OM453068 | Homo sapiens | China: Henan | 2014 | 14-China_Henan-143 | Ⅰ |
|  | OM453069 | Homo sapiens | China: Henan | 2014 | 14-China_Henan-148 | Ⅰ |
|  | OM453070 | Homo sapiens | China: Henan | 2014 | 14-China_Henan-149 | Ⅰ |
|  | OM453071 | Homo sapiens | China: Henan | 2014 | 14-China_Henan-15 | Ⅱ |
|  | OM453072 | Homo sapiens | China: Henan | 2014 | 14-China_Henan-151 | Ⅰ |
|  | OM453073 | Homo sapiens | China: Henan | 2014 | 14-China_Henan-152 | Ⅰ |
|  | OM453074 | Homo sapiens | China: Henan | 2014 | 14-China_Henan-172 | Ⅱ |
|  | OM453075 | Homo sapiens | China: Henan | 2014 | 14-China_Henan-174 | Ⅱ |
|  | OM453076 | Homo sapiens | China: Henan | 2014 | 14-China_Henan-175 | Ⅰ |
|  | OM453077 | Homo sapiens | China: Henan | 2014 | 14-China_Henan-177 | Ⅰ |
|  | OM453078 | Homo sapiens | China: Henan | 2014 | 14-China_Henan-178 | Ⅰ |
|  | OM453079 | Homo sapiens | China: Henan | 2014 | 14-China_Henan-189 | Ⅰ |
|  | OM453080 | Homo sapiens | China: Henan | 2014 | 14-China_Henan-191 | Ⅱ |
|  | OM453081 | Homo sapiens | China: Henan | 2014 | 14-China_Henan-193 | Ⅰ |
|  | OM453082 | Homo sapiens | China: Henan | 2014 | 14-China_Henan-20 | Ⅰ |
|  | OM453083 | Homo sapiens | China: Henan | 2014 | 14-China_Henan-22 | Ⅱ |
|  | OM453084 | Homo sapiens | China: Henan | 2014 | 14-China_Henan-222 | Ⅰ |
|  | OM453085 | Homo sapiens | China: Henan | 2014 | 14-China_Henan-236 | Ⅱ |
|  | OM453086 | Homo sapiens | China: Henan | 2014 | 14-China_Henan-26 | Ⅰ |
|  | OM453087 | Homo sapiens | China: Henan | 2014 | 14-China_Henan-3 | Ⅰ |
|  | OM453088 | Homo sapiens | China: Henan | 2014 | 14-China_Henan-307 | Ⅳ |
|  | OM453089 | Homo sapiens | China: Henan | 2014 | 14-China_Henan-311 | Ⅱ |
|  | OM453090 | Homo sapiens | China: Henan | 2014 | 14-China_Henan-312 | Ⅳ |
|  | MN509905 | Homo sapiens | China: Henan | 2014 | 14-China_Henan-314 | Ⅰ |
|  | OM453091 | Homo sapiens | China: Henan | 2014 | 14-China_Henan-316 | Ⅰ |
|  | OM453092 | Homo sapiens | China: Henan | 2014 | 14-China_Henan-320 | Ⅱ |
|  | OM453093 | Homo sapiens | China: Henan | 2014 | 14-China_Henan-326 | Ⅱ |
|  | OM453094 | Homo sapiens | China: Henan | 2014 | 14-China_Henan-337 | Ⅰ |
|  | OM453095 | Homo sapiens | China: Henan | 2014 | 14-China_Henan-346 | Ⅱ |
|  | OM453096 | Homo sapiens | China: Henan | 2014 | 14-China_Henan-35 | Ⅰ |
|  | OM453097 | Homo sapiens | China: Henan | 2014 | 14-China_Henan-403 | Ⅱ |
|  | OM453098 | Homo sapiens | China: Henan | 2014 | 14-China_Henan-422 | Ⅰ |
|  | MN509909 | Homo sapiens | China: Henan | 2014 | 14-China_Henan-424 | Ⅲ |
|  | OM453099 | Homo sapiens | China: Henan | 2014 | 14-China_Henan-436 | Ⅰ |
|  | OM453100 | Homo sapiens | China: Henan | 2014 | 14-China_Henan-438 | Ⅰ |
|  | OM453101 | Homo sapiens | China: Henan | 2014 | 14-China_Henan-440 | Ⅱ |
|  | MN509910 | Homo sapiens | China: Hubei | 2014 | 14-China_Hubei-444 | Ⅰ |
|  | OM453102 | Homo sapiens | China: Henan | 2014 | 14-China_Henan-446 | Ⅰ |
|  | MN509911 | Homo sapiens | China: Henan | 2014 | 14-China_Henan-452 | Ⅰ |
|  | OM453103 | Homo sapiens | China: Henan | 2014 | 14-China_Henan-453 | Ⅰ |
|  | OM453104 | Homo sapiens | China: Henan | 2014 | 14-China_Henan-463 | Ⅰ |
|  | OM453105 | Homo sapiens | China: Henan | 2014 | 14-China_Henan-474 | Ⅱ |
|  | OM453106 | Homo sapiens | China: Henan | 2014 | 14-China_Henan-484 | Ⅰ |
|  | MN509912 | Homo sapiens | China: Henan | 2014 | 14-China_Henan-485 | Ⅳ |
|  | OM453107 | Homo sapiens | China: Henan | 2014 | 14-China_Henan-488 | Ⅰ |
|  | OM453108 | Homo sapiens | China: Henan | 2014 | 14-China_Henan-49 | Ⅰ |
|  | MN509913 | Homo sapiens | China: Hubei | 2014 | 14-China_Hubei-492 | Ⅰ |
|  | OM453109 | Homo sapiens | China: Henan | 2014 | 14-China_Henan-494 | Ⅱ |
|  | OM453110 | Homo sapiens | China: Henan | 2014 | 14-China_Henan-499 | Ⅰ |
|  | OM453111 | Homo sapiens | China: Henan | 2014 | 14-China_Henan-5 | Ⅰ |
|  | MN509914 | Homo sapiens | China: Henan | 2014 | 14-China_Henan-501 | Ⅲ |
|  | MN509915 | Homo sapiens | China: Hubei | 2014 | 14-China_Hubei-512 | Ⅲ |
|  | OM453112 | Homo sapiens | China: Henan | 2014 | 14-China_Henan-523 | Ⅱ |
|  | OM453113 | Homo sapiens | China: Henan | 2014 | 14-China_Henan-529 | Ⅰ |
|  | OM453114 | Homo sapiens | China: Henan | 2014 | 14-China_Henan-54 | Ⅱ |
|  | OM453115 | Homo sapiens | China: Henan | 2014 | 14-China_Henan-540 | Ⅱ |
|  | OM453116 | Homo sapiens | China: Henan | 2014 | 14-China_Henan-542 | Ⅳ |
|  | OM453117 | Homo sapiens | China: Henan | 2014 | 14-China_Henan-546 | Ⅳ |
|  | MN509916 | Homo sapiens | China: Hubei | 2014 | 14-China_Hubei-548 | Ⅰ |
|  | OM453118 | Homo sapiens | China: Henan | 2014 | 14-China_Henan-55 | Ⅰ |
|  | OM453119 | Homo sapiens | China: Henan | 2014 | 14-China_Henan-555 | Ⅱ |
|  | OM453120 | Homo sapiens | China: Henan | 2014 | 14-China_Henan-559 | Ⅱ |
|  | OM453121 | Homo sapiens | China: Henan | 2014 | 14-China_Henan-561 | Ⅱ |
|  | OM453122 | Homo sapiens | China: Henan | 2014 | 14-China_Henan-562 | Ⅳ |
|  | MN509917 | Homo sapiens | China: Henan | 2014 | 14-China_Henan-563 | Ⅲ |
|  | OM453123 | Homo sapiens | China: Henan | 2014 | 14-China_Henan-564 | Ⅰ |
|  | MN509918 | Homo sapiens | China: Henan | 2014 | 14-China_Henan-567 | Ⅲ |
|  | OM453124 | Homo sapiens | China: Henan | 2014 | 14-China_Henan-568 | Ⅰ |
|  | OM453125 | Homo sapiens | China: Henan | 2014 | 14-China_Henan-570 | Ⅰ |
|  | MN509919 | Homo sapiens | China: Henan | 2014 | 14-China_Henan-571 | Ⅰ |
|  | OM453126 | Homo sapiens | China: Henan | 2014 | 14-China_Henan-573 | Ⅰ |
|  | OM453127 | Homo sapiens | China: Henan | 2014 | 14-China_Henan-574 | Ⅳ |
|  | OM453128 | Homo sapiens | China: Henan | 2014 | 14-China_Henan-578 | Ⅰ |
|  | OM453129 | Homo sapiens | China: Henan | 2014 | 14-China_Henan-579 | Ⅱ |
|  | OM453130 | Homo sapiens | China: Henan | 2014 | 14-China_Henan-58 | Ⅰ |
|  | OM453131 | Homo sapiens | China: Henan | 2014 | 14-China_Henan-581 | Ⅰ |
|  | OM453132 | Homo sapiens | China: Henan | 2014 | 14-China_Henan-582 | Ⅰ |
|  | OM453133 | Homo sapiens | China: Henan | 2014 | 14-China_Henan-586 | Ⅱ |
|  | OM453134 | Homo sapiens | China: Henan | 2014 | 14-China_Henan-592 | Ⅰ |
|  | OM453135 | Homo sapiens | China: Henan | 2014 | 14-China_Henan-593 | Ⅰ |
|  | MN509920 | Homo sapiens | China: Henan | 2014 | 14-China_Henan-600 | Ⅳ |
|  | OM453136 | Homo sapiens | China: Henan | 2014 | 14-China_Henan-601 | Ⅰ |
|  | OM453137 | Homo sapiens | China: Henan | 2014 | 14-China_Henan-604 | Ⅰ |
|  | MN509921 | Homo sapiens | China: Henan | 2014 | 14-China_Henan-605 | Ⅰ |
|  | OM453138 | Homo sapiens | China: Henan | 2014 | 14-China_Henan-606 | Ⅰ |
|  | OM453139 | Homo sapiens | China: Henan | 2014 | 14-China_Henan-607 | Ⅰ |
|  | OM453140 | Homo sapiens | China: Henan | 2014 | 14-China_Henan-61 | Ⅰ |
|  | OM453141 | Homo sapiens | China: Henan | 2014 | 14-China_Henan-610 | Ⅰ |
|  | MN509922 | Homo sapiens | China: Henan | 2014 | 14-China_Henan-613 | Ⅰ |
|  | MN509923 | Homo sapiens | China: Henan | 2014 | 14-China_Henan-617 | Ⅰ |
|  | OM453142 | Homo sapiens | China: Henan | 2014 | 14-China_Henan-62 | Ⅱ |
|  | OM453143 | Homo sapiens | China: Henan | 2014 | 14-China_Henan-63 | Ⅰ |
|  | OM453144 | Homo sapiens | China: Henan | 2014 | 14-China_Henan-66 | Ⅰ |
|  | OM453145 | Homo sapiens | China: Henan | 2014 | 14-China_Henan-68 | Ⅰ |
|  | OM453146 | Homo sapiens | China: Henan | 2014 | 14-China_Henan-69 | Ⅰ |
|  | OM453147 | Homo sapiens | China: Henan | 2014 | 14-China_Henan-70 | Ⅰ |
|  | MN509899 | Homo sapiens | China: Henan | 2014 | 14-China_Henan-71 | Ⅱ |
|  | OM453148 | Homo sapiens | China: Henan | 2014 | 14-China_Henan-74 | Ⅰ |
|  | OM453149 | Homo sapiens | China: Henan | 2014 | 14-China_Henan-76 | Ⅰ |
|  | OM453150 | Homo sapiens | China: Henan | 2014 | 14-China_Henan-79 | Ⅰ |
|  | OM453151 | Homo sapiens | China: Henan | 2014 | 14-China_Henan-84 | Ⅱ |
|  | OM453152 | Homo sapiens | China: Henan | 2014 | 14-China_Henan-86 | Ⅰ |
|  | OM453153 | Homo sapiens | China: Henan | 2014 | 14-China_Henan-9 | Ⅰ |
|  | OM453154 | Homo sapiens | China: Henan | 2016 | 16-China_Henan-110 | Ⅳ |
|  | OM453155 | Homo sapiens | China: Henan | 2015 | 15-China_Henan-113 | Ⅰ |
|  | OM453156 | Homo sapiens | China: Henan | 2015 | 15-China_Henan-117 | Ⅰ |
|  | OM453157 | Homo sapiens | China: Henan | 2015 | 15-China_Henan-123 | Ⅰ |
|  | OM453158 | Homo sapiens | China: Henan | 2015 | 15-China_Henan-127 | Ⅰ |
|  | MN509927 | Homo sapiens | China: Henan | 2015 | 15-China_Henan-130 | Ⅲ |
|  | OM453159 | Homo sapiens | China: Henan | 2015 | 15-China_Henan-132 | Ⅰ |
|  | OM453160 | Homo sapiens | China: Henan | 2015 | 15-China_Henan-134 | Ⅰ |
|  | OM453161 | Homo sapiens | China: Henan | 2016 | 16-China_Henan-136 | Ⅳ |
|  | MN509929 | Homo sapiens | China: Henan | 2015 | 15-China_Henan-141 | Ⅱ |
|  | OM453162 | Homo sapiens | China: Henan | 2015 | 15-China_Henan-147 | Ⅱ |
|  | OM453163 | Homo sapiens | China: Henan | 2015 | 15-China_Henan-15 | Ⅳ |
|  | OM453164 | Homo sapiens | China: Henan | 2015 | 15-China_Henan-150 | Ⅰ |
|  | OM453165 | Homo sapiens | China: Henan | 2015 | 15-China_Henan-151 | Ⅰ |
|  | OM453166 | Homo sapiens | China: Henan | 2015 | 15-China_Henan-153 | Ⅰ |
|  | MN509930 | Homo sapiens | China: Hubei | 2015 | 15-China_Hubei-154 | Ⅰ |
|  | OM453167 | Homo sapiens | China: Henan | 2015 | 15-China_Henan-155 | Ⅱ |
|  | OM453168 | Homo sapiens | China: Henan | 2015 | 15-China_Henan-16 | Ⅰ |
|  | OM453169 | Homo sapiens | China: Henan | 2015 | 15-China_Henan-160 | Ⅰ |
|  | OM453170 | Homo sapiens | China: Henan | 2015 | 15-China_Henan-165 | Ⅱ |
|  | OM453171 | Homo sapiens | China: Henan | 2015 | 15-China_Henan-168 | Ⅱ |
|  | OM453172 | Homo sapiens | China: Henan | 2015 | 15-China_Henan-170 | Ⅰ |
|  | OM453173 | Homo sapiens | China: Henan | 2015 | 15-China_Henan-174 | Ⅰ |
|  | OM453174 | Homo sapiens | China: Henan | 2015 | 15-China_Henan-176 | Ⅰ |
|  | OM453175 | Homo sapiens | China: Henan | 2015 | 15-China_Henan-178 | Ⅰ |
|  | OM453176 | Homo sapiens | China: Henan | 2015 | 15-China_Henan-179 | Ⅱ |
|  | OM453177 | Homo sapiens | China: Henan | 2015 | 15-China_Henan-2 | Ⅰ |
|  | OM453178 | Homo sapiens | China: Henan | 2015 | 15-China_Henan-200 | Ⅰ |
|  | MN509931 | Homo sapiens | China: Henan | 2015 | 15-China_Henan-201 | Ⅰ |
|  | OM453179 | Homo sapiens | China: Henan | 2015 | 15-China_Henan-204 | Ⅳ |
|  | OM453180 | Homo sapiens | China: Henan | 2015 | 15-China_Henan-206 | Ⅱ |
|  | MN509932 | Homo sapiens | China: Henan | 2015 | 15-China_Henan-211 | Ⅰ |
|  | OM453181 | Homo sapiens | China: Henan | 2015 | 15-China_Henan-213 | Ⅳ |
|  | MN509933 | Homo sapiens | China: Henan | 2015 | 15-China_Henan-217 | Ⅲ |
|  | MN509934 | Homo sapiens | China: Henan | 2015 | 15-China_Henan-221 | Ⅰ |
|  | OM453182 | Homo sapiens | China: Henan | 2015 | 15-China_Henan-224 | Ⅳ |
|  | OM453183 | Homo sapiens | China: Henan | 2015 | 15-China_Henan-225 | Ⅰ |
|  | OM453184 | Homo sapiens | China: Henan | 2015 | 15-China_Henan-226 | Ⅱ |
|  | OM453185 | Homo sapiens | China: Henan | 2015 | 15-China_Henan-227 | Ⅰ |
|  | OM453186 | Homo sapiens | China: Henan | 2015 | 15-China_Henan-228 | Ⅱ |
|  | OM453187 | Homo sapiens | China: Henan | 2015 | 15-China_Henan-229 | Ⅰ |
|  | OM453188 | Homo sapiens | China: Henan | 2015 | 15-China_Henan-23 | Ⅳ |
|  | OM453189 | Homo sapiens | China: Henan | 2015 | 15-China_Henan-232 | Ⅰ |
|  | MN509935 | Homo sapiens | China: Hubei | 2015 | 15-China_Hubei-234 | Ⅰ |
|  | OM453190 | Homo sapiens | China: Henan | 2015 | 15-China_Henan-238 | Ⅰ |
|  | OM453191 | Homo sapiens | China: Henan | 2015 | 15-China_Henan-249 | Ⅰ |
|  | OM453192 | Homo sapiens | China: Henan | 2015 | 15-China_Henan-250 | Ⅰ |
|  | MN509937 | Homo sapiens | China: Henan | 2015 | 15-China_Henan-256 | Ⅰ |
|  | OM453193 | Homo sapiens | China: Henan | 2015 | 15-China_Henan-257 | Ⅰ |
|  | OM453194 | Homo sapiens | China: Henan | 2015 | 15-China_Henan-258 | Ⅰ |
|  | MN509938 | Homo sapiens | China: Hubei | 2015 | 15-China_Hubei-259 | Ⅰ |
|  | MN509939 | Homo sapiens | China: Henan | 2015 | 15-China_Henan-260 | Ⅲ |
|  | OM453195 | Homo sapiens | China: Henan | 2015 | 15-China_Henan-266 | Ⅰ |
|  | OM453196 | Homo sapiens | China: Henan | 2015 | 15-China_Henan-29 | Ⅳ |
|  | OM453197 | Homo sapiens | China: Henan | 2015 | 15-China_Henan-30 | Ⅰ |
|  | MN509941 | Homo sapiens | China: Henan | 2015 | 15-China_Henan-316 | Ⅰ |
|  | OM453198 | Homo sapiens | China: Henan | 2015 | 15-China_Henan-321 | Ⅱ |
|  | MN509942 | Homo sapiens | China: Henan | 2015 | 15-China_Henan-322 | Ⅰ |
|  | OM453199 | Homo sapiens | China: Henan | 2015 | 15-China_Henan-323 | Ⅰ |
|  | OM453200 | Homo sapiens | China: Henan | 2015 | 15-China_Henan-326 | Ⅰ |
|  | OM453201 | Homo sapiens | China: Henan | 2015 | 15-China_Henan-338 | Ⅰ |
|  | MN509924 | Homo sapiens | China: Henan | 2015 | 15-China_Henan-34 | Ⅰ |
|  | OM453202 | Homo sapiens | China: Henan | 2015 | 15-China_Henan-343 | Ⅰ |
|  | OM453203 | Homo sapiens | China: Henan | 2015 | 15-China_Henan-35 | Ⅰ |
|  | OM453204 | Homo sapiens | China: Henan | 2015 | 15-China_Henan-351 | Ⅱ |
|  | OM453205 | Homo sapiens | China: Henan | 2015 | 15-China_Henan-353 | Ⅳ |
|  | OM453206 | Homo sapiens | China: Henan | 2015 | 15-China_Henan-356 | Ⅳ |
|  | MN509945 | Homo sapiens | China: Henan | 2015 | 15-China_Henan-363 | Ⅰ |
|  | OM453207 | Homo sapiens | China: Henan | 2016 | 16-China_Henan-364 | Ⅳ |
|  | OM453208 | Homo sapiens | China: Henan | 2015 | 15-China_Henan-365 | Ⅰ |
|  | OM453209 | Homo sapiens | China: Henan | 2015 | 15-China_Henan-374 | Ⅱ |
|  | MN509947 | Homo sapiens | China: Henan | 2015 | 15-China_Henan-380 | Ⅰ |
|  | OM453210 | Homo sapiens | China: Henan | 2015 | 15-China_Henan-391 | Ⅰ |
|  | OM453211 | Homo sapiens | China: Henan | 2015 | 15-China_Henan-393 | Ⅰ |
|  | OM453212 | Homo sapiens | China: Henan | 2015 | 15-China_Henan-398 | Ⅳ |
|  | OM453213 | Homo sapiens | China: Henan | 2015 | 15-China_Henan-4 | Ⅰ |
|  | OM453214 | Homo sapiens | China: Henan | 2015 | 15-China_Henan-40 | Ⅰ |
|  | OM453215 | Homo sapiens | China: Henan | 2015 | 15-China_Henan-411 | Ⅱ |
|  | OM453216 | Homo sapiens | China: Henan | 2015 | 15-China_Henan-413 | Ⅰ |
|  | MN509952 | Homo sapiens | China: Henan | 2015 | 15-China_Henan-414 | Ⅰ |
|  | MN509953 | Homo sapiens | China: Henan | 2015 | 15-China_Henan-415 | Ⅰ |
|  | MN509925 | Homo sapiens | China: Henan | 2015 | 15-China_Henan-55 | Ⅳ |
|  | OM453217 | Homo sapiens | China: Henan | 2015 | 15-China_Henan-58 | Ⅱ |
|  | OM453218 | Homo sapiens | China: Henan | 2015 | 15-China_Henan-61 | Ⅰ |
|  | OM453219 | Homo sapiens | China: Henan | 2015 | 15-China_Henan-62 | Ⅰ |
|  | OM453220 | Homo sapiens | China: Henan | 2015 | 15-China_Henan-68 | Ⅰ |
|  | OM453221 | Homo sapiens | China: Henan | 2015 | 15-China_Henan-7 | Ⅰ |
|  | OM453222 | Homo sapiens | China: Henan | 2015 | 15-China_Henan-73 | Ⅰ |
|  | OM453223 | Homo sapiens | China: Henan | 2015 | 15-China_Henan-75 | Ⅰ |
|  | OM453224 | Homo sapiens | China: Henan | 2015 | 15-China_Henan-78 | Ⅳ |
|  | OM453225 | Homo sapiens | China: Henan | 2015 | 15-China_Henan-79 | Ⅰ |
|  | OM453226 | Homo sapiens | China: Henan | 2015 | 15-China_Henan-8 | Ⅰ |
|  | OM453227 | Homo sapiens | China: Henan | 2015 | 15-China_Henan-82 | Ⅰ |
|  | OM453228 | Homo sapiens | China: Henan | 2015 | 15-China_Henan-9 | Ⅰ |
|  | OM453229 | Homo sapiens | China: Henan | 2015 | 15-China_Henan-91 | Ⅱ |
|  | OM453230 | Homo sapiens | China: Henan | 2015 | 15-China_Henan-95 | Ⅰ |
|  | OM453231 | Homo sapiens | China: Henan | 2016 | 16-China_Henan-115 | Ⅱ |
|  | OM453232 | Homo sapiens | China: Henan | 2016 | 16-China_Henan-118 | Ⅰ |
|  | OM453233 | Homo sapiens | China: Henan | 2016 | 16-China_Henan-122 | Ⅰ |
|  | OM453234 | Homo sapiens | China: Henan | 2016 | 16-China_Henan-123 | Ⅰ |
|  | OM453235 | Homo sapiens | China: Henan | 2016 | 16-China_Henan-128 | Ⅰ |
|  | MN509957 | Homo sapiens | China: Henan | 2016 | 16-China_Henan-135 | Ⅰ |
|  | MN509958 | Homo sapiens | China: Henan | 2016 | 16-China_Henan-138 | Ⅰ |
|  | MN509959 | Homo sapiens | China: Henan | 2016 | 16-China_Henan-149 | Ⅰ |
|  | OM453236 | Homo sapiens | China: Henan | 2016 | 16-China_Henan-16 | Ⅱ |
|  | OM453237 | Homo sapiens | China: Henan | 2016 | 16-China_Henan-164 | Ⅰ |
|  | OM453238 | Homo sapiens | China: Henan | 2016 | 16-China_Henan-167 | Ⅱ |
|  | MN509956 | Homo sapiens | China: Henan | 2016 | 16-China_Henan-17 | Ⅰ |
|  | MN509960 | Homo sapiens | China: Henan | 2016 | 16-China_Henan-176 | Ⅰ |
|  | OM453239 | Homo sapiens | China: Henan | 2016 | 16-China_Henan-179 | Ⅱ |
|  | OM453240 | Homo sapiens | China: Henan | 2016 | 16-China_Henan-18 | Ⅰ |
|  | OM453241 | Homo sapiens | China: Henan | 2016 | 16-China_Henan-181 | Ⅰ |
|  | OM453242 | Homo sapiens | China: Henan | 2016 | 16-China_Henan-187 | Ⅰ |
|  | OM453243 | Homo sapiens | China: Henan | 2016 | 16-China_Henan-188 | Ⅱ |
|  | OM453244 | Homo sapiens | China: Henan | 2016 | 16-China_Henan-19 | Ⅰ |
|  | OM453245 | Homo sapiens | China: Henan | 2016 | 16-China_Henan-196 | Ⅱ |
|  | MN509961 | Homo sapiens | China: Henan | 2016 | 16-China_Henan-198 | Ⅰ |
|  | MN509962 | Homo sapiens | China: Hubei | 2016 | 16-China_Hubei-203 | Ⅰ |
|  | OM453246 | Homo sapiens | China: Henan | 2016 | 16-China_Henan-212 | Ⅳ |
|  | MN509963 | Homo sapiens | China: Henan | 2016 | 16-China_Henan-245 | Ⅰ |
|  | OM453247 | Homo sapiens | China: Henan | 2016 | 16-China_Henan-251 | Ⅱ |
|  | OM453248 | Homo sapiens | China: Henan | 2016 | 16-China_Henan-279 | Ⅰ |
|  | OM453249 | Homo sapiens | China: Henan | 2016 | 16-China_Henan-283 | Ⅰ |
|  | OM453250 | Homo sapiens | China: Henan | 2016 | 16-China_Henan-286 | Ⅰ |
|  | OM453251 | Homo sapiens | China: Henan | 2016 | 16-China_Henan-29 | Ⅰ |
|  | OM453252 | Homo sapiens | China: Henan | 2016 | 16-China_Henan-305 | Ⅱ |
|  | OM453253 | Homo sapiens | China: Henan | 2016 | 16-China_Henan-32 | Ⅰ |
|  | OM453254 | Homo sapiens | China: Henan | 2016 | 16-China_Henan-335 | Ⅱ |
|  | OM453255 | Homo sapiens | China: Henan | 2016 | 16-China_Henan-341 | Ⅱ |
|  | MN509965 | Homo sapiens | China: Henan | 2016 | 16-China_Henan-370 | Ⅴ |
|  | OM453256 | Homo sapiens | China: Henan | 2016 | 16-China_Henan-39 | Ⅲ |
|  | OM453257 | Homo sapiens | China: Henan | 2016 | 16-China_Henan-397 | Ⅱ |
|  | OM453258 | Homo sapiens | China: Henan | 2016 | 16-China_Henan-401 | Ⅱ |
|  | MN509966 | Homo sapiens | China: Henan | 2016 | 16-China_Henan-402 | Ⅰ |
|  | OM453259 | Homo sapiens | China: Henan | 2016 | 16-China_Henan-405 | Ⅱ |
|  | MN509967 | Homo sapiens | China: Henan | 2016 | 16-China_Henan-412 | Ⅰ |
|  | MN509968 | Homo sapiens | China: Henan | 2016 | 16-China_Henan-418 | Ⅰ |
|  | MN509969 | Homo sapiens | China: Henan | 2016 | 16-China_Henan-421 | Ⅰ |
|  | MN509970 | Homo sapiens | China: Henan | 2016 | 16-China_Henan-428 | Ⅰ |
|  | MN509971 | Homo sapiens | China: Henan | 2016 | 16-China_Henan-431 | Ⅰ |
|  | MN509972 | Homo sapiens | China: Henan | 2016 | 16-China_Henan-439 | Ⅰ |
|  | MN509973 | Homo sapiens | China: Henan | 2016 | 16-China_Henan-444 | Ⅰ |
|  | OM453260 | Homo sapiens | China: Henan | 2016 | 16-China_Henan-452 | Ⅱ |
|  | MN509974 | Homo sapiens | China: Henan | 2016 | 16-China_Henan-453 | Ⅲ |
|  | MN509975 | Homo sapiens | China: Henan | 2016 | 16-China_Henan-456 | Ⅰ |
|  | OM453261 | Homo sapiens | China: Hubei | 2016 | 16-China_Hubei-471 | Ⅰ |
|  | OM453262 | Homo sapiens | China: Henan | 2016 | 16-China_Henan-504 | Ⅱ |
|  | OM453263 | Homo sapiens | China: Henan | 2016 | 16-China_Henan-532 | Ⅰ |
|  | OM453264 | Homo sapiens | China: Henan | 2016 | 16-China_Henan-590 | Ⅰ |
|  | OM453265 | Homo sapiens | China: Henan | 2016 | 16-China_Henan-595 | Ⅱ |
|  | OM453266 | Homo sapiens | China: Henan | 2016 | 16-China_Henan-598 | Ⅲ |
|  | OM453267 | Homo sapiens | China: Henan | 2016 | 16-China_Henan-605 | Ⅰ |
|  | OM453268 | Homo sapiens | China: Henan | 2016 | 16-China_Henan-614 | Ⅰ |
|  | OM453269 | Homo sapiens | China: Henan | 2016 | 16-China_Henan-64 | Ⅰ |
|  | OM453270 | Homo sapiens | China: Henan | 2016 | 16-China_Henan-74 | Ⅱ |
|  | OM453271 | Homo sapiens | China: Henan | 2016 | 16-China_Henan-81 | Ⅳ |
|  | MN509982 | Homo sapiens | China: Henan | 2017 | 17-China_Henan-110 | Ⅰ |
|  | MN509983 | Homo sapiens | China: Henan | 2017 | 17-China_Henan-112 | Ⅰ |
|  | MN509984 | Homo sapiens | China: Henan | 2017 | 17-China_Henan-116 | Ⅰ |
|  | OM453272 | Homo sapiens | China: Henan | 2017 | 17-China_Henan-138 | Ⅱ |
|  | MN509986 | Homo sapiens | China: Henan | 2017 | 17-China_Henan-142 | Ⅰ |
|  | MN509987 | Homo sapiens | China: Henan | 2017 | 17-China_Henan-143 | Ⅰ |
|  | MN509988 | Homo sapiens | China: Henan | 2017 | 17-China_Henan-154 | Ⅰ |
|  | MN509989 | Homo sapiens | China: Henan | 2017 | 17-China_Henan-178 | Ⅰ |
|  | MN509990 | Homo sapiens | China: Henan | 2017 | 17-China_Henan-215 | Ⅰ |
|  | OM453273 | Homo sapiens | China: Henan | 2017 | 17-China_Henan-218 | Ⅳ |
|  | MN509991 | Homo sapiens | China: Henan | 2017 | 17-China_Henan-242 | Ⅱ |
|  | OM453274 | Homo sapiens | China: Hubei | 2017 | 17-China_Hubei-25 | Ⅲ |
|  | OM453275 | Homo sapiens | China: Henan | 2017 | 17-China_Henan-257 | Ⅳ |
|  | MN509992 | Homo sapiens | China: Henan | 2017 | 17-China_Henan-290 | Ⅰ |
|  | MN509993 | Homo sapiens | China: Henan | 2017 | 17-China_Henan-317 | Ⅰ |
|  | OM453276 | Homo sapiens | China: Henan | 2017 | 17-China_Henan-320 | Ⅱ |
|  | MN509994 | Homo sapiens | China: Henan | 2017 | 17-China_Henan-337 | Ⅰ |
|  | MN509976 | Homo sapiens | China: Henan | 2017 | 17-China_Henan-35 | Ⅰ |
|  | MN509995 | Homo sapiens | China: Henan | 2017 | 17-China_Henan-356 | Ⅰ |
|  | MN509996 | Homo sapiens | China: Henan | 2017 | 17-China_Henan-365 | Ⅰ |
|  | MN509977 | Homo sapiens | China: Henan | 2017 | 17-China_Henan-51 | Ⅰ |
|  | MN509978 | Homo sapiens | China: Henan | 2017 | 17-China_Henan-64 | Ⅰ |
|  | OM453277 | Homo sapiens | China: Henan | 2017 | 17-China_Henan-76 | Ⅲ |
|  | OM453278 | Homo sapiens | China: Henan | 2017 | 17-China_Henan-84 | Ⅲ |
|  | OM453279 | Homo sapiens | China: Henan | 2017 | 17-China_Henan-91 | Ⅱ |
|  | MN509980 | Homo sapiens | China: Henan | 2017 | 17-China_Henan-94 | Ⅰ |
|  | MN509981 | Homo sapiens | China: Henan | 2017 | 17-China_Henan-96 | Ⅲ |
|  | OM453280 | Homo sapiens | China: Henan | 2017 | 17-China_Henan-101 | Ⅰ |
|  | OM453281 | Homo sapiens | China: Hubei | 2017 | 17-China_Hubei-102 | Ⅰ |
|  | OM453282 | Homo sapiens | China: Henan | 2018 | 18-China_Henan-103 | Ⅰ |
|  | OM453283 | Homo sapiens | China: Henan | 2018 | 18-China_Henan-105 | Ⅱ |
|  | OM453284 | Homo sapiens | China: Henan | 2017 | 17-China_Henan-107 | Ⅰ |
|  | OM453285 | Homo sapiens | China: Henan | 2018 | 18-China_Henan-109 | Ⅰ |
|  | OM453286 | Homo sapiens | China: Henan | 2018 | 18-China_Henan-110 | Ⅱ |
|  | OM453287 | Homo sapiens | China: Henan | 2017 | 17-China_Henan-114 | Ⅰ |
|  | OM453288 | Homo sapiens | China: Henan | 2017 | 17-China_Henan-113 | Ⅰ |
|  | OM453289 | Homo sapiens | China: Henan | 2018 | 18-China_Henan-114 | Ⅳ |
|  | OM453290 | Homo sapiens | China: Henan | 2018 | 18-China_Henan-115 | Ⅱ |
|  | OM453291 | Homo sapiens | China: Hubei | 2018 | 18-China_Hubei-118 | Ⅰ |
|  | OM453292 | Homo sapiens | China: Hubei | 2017 | 17-China_Hubei-120 | Ⅰ |
|  | OM453293 | Homo sapiens | China: Hubei | 2018 | 18-China_Hubei-123 | Ⅲ |
|  | OM453294 | Homo sapiens | China: Henan | 2017 | 17-China_Henan-128 | Ⅰ |
|  | OM453295 | Homo sapiens | China: Henan | 2018 | 18-China_Henan-129 | Ⅱ |
|  | OM453296 | Homo sapiens | China: Henan | 2017 | 17-China_Henan-13 | Ⅰ |
|  | OM453297 | Homo sapiens | China: Henan | 2018 | 18-China_Henan-130 | Ⅰ |
|  | OM453298 | Homo sapiens | China: Henan | 2017 | 17-China_Henan-136 | Ⅱ |
|  | OM453299 | Homo sapiens | China: Henan | 2017 | 17-China_Henan-139 | Ⅰ |
|  | OM453300 | Homo sapiens | China: Henan | 2018 | 18-China_Henan-139 | Ⅰ |
|  | OM453301 | Homo sapiens | China: Henan | 2018 | 18-China_Henan-141 | Ⅰ |
|  | OM453302 | Homo sapiens | China: Henan | 2018 | 18-China_Henan-143 | Ⅰ |
|  | OM453303 | Homo sapiens | China: Hubei | 2017 | 17-China_Hubei-144 | Ⅰ |
|  | OM453304 | Homo sapiens | China: Henan | 2017 | 17-China_Henan-147 | Ⅰ |
|  | OM453305 | Homo sapiens | China: Henan | 2017 | 17-China_Henan-148 | Ⅰ |
|  | OM453306 | Homo sapiens | China: Henan | 2018 | 18-China_Henan-149 | Ⅰ |
|  | OM453307 | Homo sapiens | China: Henan | 2018 | 18-China_Henan-150 | Ⅰ |
|  | OM453308 | Homo sapiens | China: Henan | 2018 | 18-China_Henan-152 | Ⅱ |
|  | OM453309 | Homo sapiens | China: Henan | 2018 | 18-China_Henan-153 | Ⅳ |
|  | OM453310 | Homo sapiens | China: Henan | 2018 | 18-China_Henan-154 | Ⅰ |
|  | OM453311 | Homo sapiens | China: Henan | 2018 | 18-China_Henan-155 | Ⅱ |
|  | OM453312 | Homo sapiens | China: Henan | 2018 | 18-China_Henan-158 | Ⅱ |
|  | OM453313 | Homo sapiens | China: Henan | 2018 | 18-China_Henan-159 | Ⅰ |
|  | OM453314 | Homo sapiens | China: Henan | 2018 | 18-China_Henan-160 | Ⅰ |
|  | OM453315 | Homo sapiens | China: Henan | 2018 | 18-China_Henan-161 | Ⅰ |
|  | OM453316 | Homo sapiens | China: Henan | 2017 | 17-China_Henan-163 | Ⅰ |
|  | OM453317 | Homo sapiens | China: Henan | 2018 | 18-China_Henan-164 | Ⅳ |
|  | OM453318 | Homo sapiens | China: Henan | 2017 | 17-China_Henan-165 | Ⅳ |
|  | OM453319 | Homo sapiens | China: Hubei | 2018 | 18-China_Hubei-167 | Ⅰ |
|  | OM453320 | Homo sapiens | China: Henan | 2018 | 18-China_Henan-169 | Ⅰ |
|  | OM453321 | Homo sapiens | China: Henan | 2018 | 18-China_Henan-170 | Ⅰ |
|  | OM453322 | Homo sapiens | China: Henan | 2018 | 18-China_Henan-172 | Ⅳ |
|  | OM453323 | Homo sapiens | China: Henan | 2017 | 17-China_Henan-177 | Ⅰ |
|  | OM453324 | Homo sapiens | China: Henan | 2018 | 18-China_Henan-178 | Ⅰ |
|  | OM453325 | Homo sapiens | China: Henan | 2018 | 18-China_Henan-180 | Ⅰ |
|  | OM453326 | Homo sapiens | China: Henan | 2018 | 18-China_Henan-184 | Ⅰ |
|  | OM453327 | Homo sapiens | China: Hubei | 2018 | 18-China_Hubei-186 | Ⅲ |
|  | OM453328 | Homo sapiens | China: Henan | 2018 | 18-China_Henan-187 | Ⅰ |
|  | OM453329 | Homo sapiens | China: Henan | 2018 | 18-China_Henan-189 | Ⅰ |
|  | OM453330 | Homo sapiens | China: Henan | 2017 | 17-China_Henan-190 | Ⅳ |
|  | OM453331 | Homo sapiens | China: Henan | 2018 | 18-China_Henan-191 | Ⅰ |
|  | OM453332 | Homo sapiens | China: Henan | 2018 | 18-China_Henan-193 | Ⅰ |
|  | OM453333 | Homo sapiens | China: Henan | 2017 | 17-China_Henan-194 | Ⅱ |
|  | MN510004 | Homo sapiens | China: Hubei | 2018 | 18-China_Hubei-199 | Ⅰ |
|  | OM453334 | Homo sapiens | China: Henan | 2018 | 18-China_Henan-2 | Ⅲ |
|  | OM453335 | Homo sapiens | China: Henan | 2018 | 18-China_Henan-200 | Ⅱ |
|  | OM453336 | Homo sapiens | China: Henan | 2018 | 18-China_Henan-207 | Ⅰ |
|  | MN510005 | Homo sapiens | China: Henan | 2018 | 18-China_Henan-208 | Ⅳ |
|  | MN510006 | Homo sapiens | China: Henan | 2018 | 18-China_Henan-209 | Ⅰ |
|  | OM453337 | Homo sapiens | China: Henan | 2018 | 18-China_Henan-210 | Ⅰ |
|  | OM453338 | Homo sapiens | China: Henan | 2018 | 18-China_Henan-213 | Ⅰ |
|  | OM453339 | Homo sapiens | China: Henan | 2018 | 18-China_Henan-217 | Ⅰ |
|  | OM453340 | Homo sapiens | China: Henan | 2018 | 18-China_Henan-22 | Ⅱ |
|  | OM453341 | Homo sapiens | China: Henan | 2018 | 18-China_Henan-222 | Ⅳ |
|  | OM453342 | Homo sapiens | China: Henan | 2018 | 18-China_Henan-223 | Ⅰ |
|  | OM453343 | Homo sapiens | China: Henan | 2018 | 18-China_Henan-225 | Ⅰ |
|  | OM453344 | Homo sapiens | China: Henan | 2018 | 18-China_Henan-226 | Ⅰ |
|  | MN510007 | Homo sapiens | China: Henan | 2018 | 18-China_Henan-227 | Ⅳ |
|  | OM453345 | Homo sapiens | China: Henan | 2018 | 18-China_Henan-228 | Ⅰ |
|  | MN510009 | Homo sapiens | China: Henan | 2018 | 18-China_Henan-233 | Ⅳ |
|  | OM453346 | Homo sapiens | China: Henan | 2017 | 17-China_Henan-241 | Ⅱ |
|  | OM453347 | Homo sapiens | China: Henan | 2018 | 18-China_Henan-243 | Ⅱ |
|  | OM453348 | Homo sapiens | China: Henan | 2018 | 18-China_Henan-251 | Ⅰ |
|  | MN510012 | Homo sapiens | China: Henan | 2018 | 18-China_Henan-254 | Ⅰ |
|  | OM453349 | Homo sapiens | China: Henan | 2017 | 17-China_Henan-255 | Ⅰ |
|  | OM453350 | Homo sapiens | China: Henan | 2018 | 18-China_Henan-257 | Ⅰ |
|  | OM453351 | Homo sapiens | China: Henan | 2018 | 18-China_Henan-258 | Ⅰ |
|  | OM453352 | Homo sapiens | China: Henan | 2017 | 17-China_Henan-261 | Ⅰ |
|  | OM453353 | Homo sapiens | China: Henan | 2018 | 18-China_Henan-263 | Ⅰ |
|  | OM453354 | Homo sapiens | China: Henan | 2017 | 17-China_Henan-264 | Ⅱ |
|  | OM453355 | Homo sapiens | China: Henan | 2018 | 18-China_Henan-268 | Ⅳ |
|  | OM453356 | Homo sapiens | China: Henan | 2017 | 17-China_Henan-269 | Ⅰ |
|  | OM453357 | Homo sapiens | China: Henan | 2018 | 18-China_Henan-27 | Ⅰ |
|  | OM453358 | Homo sapiens | China: Henan | 2018 | 18-China_Henan-271 | Ⅲ |
|  | OM453359 | Homo sapiens | China: Henan | 2018 | 18-China_Henan-273 | Ⅰ |
|  | OM453360 | Homo sapiens | China: Henan | 2018 | 18-China_Henan-278 | Ⅱ |
|  | OM453361 | Homo sapiens | China: Henan | 2018 | 18-China_Henan-279 | Ⅰ |
|  | OM453362 | Homo sapiens | China: Henan | 2018 | 18-China_Henan-28 | Ⅱ |
|  | OM453363 | Homo sapiens | China: Henan | 2017 | 17-China_Henan-281 | Ⅰ |
|  | OM453364 | Homo sapiens | China: Henan | 2017 | 17-China_Henan-285 | Ⅰ |
|  | OM453365 | Homo sapiens | China: Henan | 2017 | 17-China_Henan-286 | Ⅰ |
|  | OM453366 | Homo sapiens | China: Henan | 2018 | 18-China_Henan-287 | Ⅱ |
|  | OM453367 | Homo sapiens | China: Henan | 2017 | 17-China_Henan-289 | Ⅳ |
|  | OM453368 | Homo sapiens | China: Henan | 2018 | 18-China_Henan-291 | Ⅳ |
|  | OM453369 | Homo sapiens | China: Henan | 2018 | 18-China_Henan-292 | Ⅱ |
|  | OM453370 | Homo sapiens | China: Henan | 2017 | 17-China_Henan-293 | Ⅰ |
|  | OM453371 | Homo sapiens | China: Henan | 2017 | 17-China_Henan-299 | Ⅱ |
|  | OM453372 | Homo sapiens | China: Henan | 2017 | 17-China_Henan-304 | Ⅱ |
|  | OM453373 | Homo sapiens | China: Henan | 2018 | 18-China_Henan-309 | Ⅰ |
|  | OM453374 | Homo sapiens | China: Henan | 2018 | 18-China_Henan-312 | Ⅰ |
|  | OM453375 | Homo sapiens | China: Henan | 2017 | 17-China_Henan-322 | Ⅳ |
|  | OM453376 | Homo sapiens | China: Henan | 2017 | 17-China_Henan-321 | Ⅰ |
|  | MN510014 | Homo sapiens | China: Henan | 2018 | 18-China_Henan-323 | Ⅰ |
|  | OM453377 | Homo sapiens | China: Henan | 2017 | 17-China_Henan-325 | Ⅰ |
|  | OM453378 | Homo sapiens | China: Henan | 2018 | 18-China_Henan-326 | Ⅰ |
|  | OM453379 | Homo sapiens | China: Henan | 2017 | 17-China_Henan-33 | Ⅰ |
|  | OM453380 | Homo sapiens | China: Henan | 2018 | 18-China_Henan-334 | Ⅰ |
|  | MN510016 | Homo sapiens | China: Henan | 2018 | 18-China_Henan-337 | Ⅰ |
|  | OM453381 | Homo sapiens | China: Henan | 2018 | 18-China_Henan-338 | Ⅰ |
|  | OM453382 | Homo sapiens | China: Henan | 2018 | 18-China_Henan-342 | Ⅰ |
|  | OM453383 | Homo sapiens | China: Henan | 2017 | 17-China_Henan-343 | Ⅰ |
|  | OM453384 | Homo sapiens | China: Henan | 2018 | 18-China_Henan-348 | Ⅰ |
|  | OM453385 | Homo sapiens | China: Henan | 2018 | 18-China_Henan-349 | Ⅰ |
|  | OM453386 | Homo sapiens | China: Henan | 2017 | 17-China_Henan-36 | Ⅰ |
|  | OM453387 | Homo sapiens | China: Henan | 2018 | 18-China_Henan-353 | Ⅰ |
|  | OM453388 | Homo sapiens | China: Henan | 2017 | 17-China_Henan-354 | Ⅰ |
|  | OM453389 | Homo sapiens | China: Henan | 2018 | 18-China_Henan-356 | Ⅰ |
|  | OM453390 | Homo sapiens | China: Henan | 2018 | 18-China_Henan-357 | Ⅲ |
|  | OM453391 | Homo sapiens | China: Hubei | 2018 | 18-China_Hubei-358 | Ⅰ |
|  | OM453392 | Homo sapiens | China: Henan | 2018 | 18-China_Henan-362 | Ⅰ |
|  | OM453393 | Homo sapiens | China: Henan | 2018 | 18-China_Henan-365 | Ⅰ |
|  | MN510018 | Homo sapiens | China: Henan | 2018 | 18-China_Henan-366 | Ⅰ |
|  | OM453394 | Homo sapiens | China: Henan | 2017 | 17-China_Henan-367 | Ⅰ |
|  | OM453395 | Homo sapiens | China: Hubei | 2018 | 18-China_Hubei-368 | Ⅰ |
|  | OM453396 | Homo sapiens | China: Henan | 2018 | 18-China_Henan-37 | Ⅰ |
|  | OM453397 | Homo sapiens | China: Henan | 2018 | 18-China_Henan-40 | Ⅰ |
|  | OM453398 | Homo sapiens | China: Henan | 2017 | 17-China_Henan-42 | Ⅱ |
|  | OM453399 | Homo sapiens | China: Henan | 2018 | 18-China_Henan-43 | Ⅱ |
|  | OM453400 | Homo sapiens | China: Henan | 2018 | 18-China_Henan-44 | Ⅱ |
|  | OM453401 | Homo sapiens | China: Henan | 2017 | 17-China_Henan-47 | Ⅱ |
|  | OM453402 | Homo sapiens | China: Henan | 2018 | 18-China_Henan-50 | Ⅱ |
|  | OM453403 | Homo sapiens | China: Henan | 2018 | 18-China_Henan-51 | Ⅰ |
|  | OM453404 | Homo sapiens | China: Henan | 2018 | 18-China_Henan-53 | Ⅰ |
|  | OM453405 | Homo sapiens | China: Henan | 2017 | 17-China_Henan-54 | Ⅰ |
|  | OM453406 | Homo sapiens | China: Hubei | 2018 | 18-China_Hubei-58 | Ⅲ |
|  | OM453407 | Homo sapiens | China: Henan | 2018 | 18-China_Henan-6 | Ⅰ |
|  | OM453408 | Homo sapiens | China: Henan | 2018 | 18-China_Henan-60 | Ⅰ |
|  | MN509998 | Homo sapiens | China: Henan | 2018 | 18-China_Henan-61 | Ⅲ |
|  | OM453409 | Homo sapiens | China: Henan | 2018 | 18-China_Henan-64 | Ⅰ |
|  | MN509999 | Homo sapiens | China: Henan | 2018 | 18-China_Henan-65 | Ⅲ |
|  | OM453410 | Homo sapiens | China: Henan | 2017 | 17-China_Henan-67 | Ⅰ |
|  | OM453411 | Homo sapiens | China: Henan | 2018 | 18-China_Henan-7 | Ⅱ |
|  | OM453412 | Homo sapiens | China: Henan | 2018 | 18-China_Henan-70 | Ⅰ |
|  | OM453413 | Homo sapiens | China: Henan | 2018 | 18-China_Henan-71 | Ⅰ |
|  | OM453414 | Homo sapiens | China: Henan | 2017 | 17-China_Henan-72 | Ⅰ |
|  | OM453415 | Homo sapiens | China: Henan | 2017 | 17-China_Henan-79 | Ⅰ |
|  | OM453416 | Homo sapiens | China: Henan | 2018 | 18-China_Henan-80 | Ⅰ |
|  | OM453417 | Homo sapiens | China: Henan | 2018 | 18-China_Henan-81 | Ⅳ |
|  | MN510000 | Homo sapiens | China: Henan | 2018 | 18-China_Henan-84 | Ⅰ |
|  | OM453418 | Homo sapiens | China: Henan | 2017 | 17-China_Henan-87 | Ⅱ |
|  | MN510001 | Homo sapiens | China: Henan | 2018 | 18-China_Henan-89 | Ⅲ |
|  | OM453419 | Homo sapiens | China: Henan | 2018 | 18-China_Henan-93 | Ⅱ |
|  | OM453420 | Homo sapiens | China: Henan | 2018 | 18-China_Henan-94 | Ⅰ |
|  | OM453421 | Homo sapiens | China: Henan | 2017 | 17-China_Henan-98 | Ⅰ |
|  | OM453422 | Homo sapiens | China: Henan | 2017 | 17-China_Henan-99 | Ⅰ |
|  | OM453423 | Homo sapiens | China: Henan | 2019 | 19-China_Henan-12 | Ⅰ |
|  | OM453424 | Homo sapiens | China: Hubei | 2019 | 19-China_Hubei-144 | Ⅰ |
|  | OM453425 | Homo sapiens | China: Henan | 2019 | 19-China_Henan-162 | Ⅳ |
|  | OM453426 | Homo sapiens | China: Hubei | 2019 | 19-China_Hubei-167 | Ⅰ |
|  | OM453427 | Homo sapiens | China: Henan | 2019 | 19-China_Henan-186 | Ⅰ |
|  | OM453428 | Homo sapiens | China: Henan | 2019 | 19-China_Henan-19 | Ⅳ |
|  | OM453429 | Homo sapiens | China: Henan | 2019 | 19-China_Henan-193 | Ⅰ |
|  | OM453430 | Homo sapiens | China: Henan | 2019 | 19-China_Henan-231 | Ⅰ |
|  | OM453431 | Homo sapiens | China: Henan | 2019 | 19-China_Henan-233 | Ⅳ |
|  | OM453432 | Homo sapiens | China: Henan | 2019 | 19-China_Henan-234 | Ⅱ |
|  | OM453433 | Homo sapiens | China: Henan | 2019 | 19-China_Henan-237 | Ⅰ |
|  | OM453434 | Homo sapiens | China: Hubei | 2019 | 19-China_Hubei-238 | Ⅲ |
|  | OM453435 | Homo sapiens | China: Henan | 2019 | 19-China_Henan-248 | Ⅰ |
|  | OM453436 | Homo sapiens | China: Henan | 2019 | 19-China_Henan-271 | Ⅱ |
|  | OM453437 | Homo sapiens | China: Henan | 2019 | 19-China_Henan-287 | Ⅱ |
|  | OM453438 | Homo sapiens | China: Henan | 2019 | 19-China_Henan-3 | Ⅰ |
|  | OM453439 | Homo sapiens | China: Henan | 2019 | 19-China_Henan-309 | Ⅰ |
|  | OM453440 | Homo sapiens | China: Henan | 2019 | 19-China_Henan-313 | Ⅰ |
|  | OM453441 | Homo sapiens | China: Henan | 2019 | 19-China_Henan-316 | Ⅱ |
|  | OM453442 | Homo sapiens | China: Henan | 2019 | 19-China_Henan-317 | Ⅰ |
|  | OM453443 | Homo sapiens | China: Henan | 2019 | 19-China_Henan-330 | Ⅰ |
|  | OM453444 | Homo sapiens | China: Hubei | 2019 | 19-China_Hubei-7 | Ⅰ |
|  | OM453445 | Homo sapiens | China: Henan | 2019 | 19-China_Henan-94 | Ⅰ |
|  | OM453446 | Homo sapiens | China: Henan | 2020 | 20-China_Henan-1 | Ⅰ |
|  | OM453447 | Homo sapiens | China: Henan | 2020 | 20-China_Henan-100 | Ⅱ |
|  | OM453448 | Homo sapiens | China: Henan | 2020 | 20-China_Henan-102 | Ⅱ |
|  | OM453449 | Homo sapiens | China: Jiangxi | 2020 | 20-China_Jiangxi-104 | Ⅱ |
|  | OM453450 | Homo sapiens | China: Henan | 2020 | 20-China_Henan-108 | Ⅰ |
|  | OM453451 | Homo sapiens | China: Henan | 2020 | 20-China_Henan-111 | Ⅲ |
|  | OM453452 | Homo sapiens | China: Henan | 2020 | 20-China_Henan-112 | Ⅰ |
|  | OM453453 | Homo sapiens | China: Henan | 2020 | 20-China_Henan-113 | Ⅰ |
|  | OM453454 | Homo sapiens | China: Henan | 2020 | 20-China_Henan-114 | Ⅰ |
|  | OM453455 | Homo sapiens | China: Hubei | 2020 | 20-China_Hubei-116 | Ⅰ |
|  | OM453456 | Homo sapiens | China: Henan | 2020 | 20-China_Henan-118 | Ⅰ |
|  | OM453457 | Homo sapiens | China: Henan | 2020 | 20-China_Henan-120 | Ⅰ |
|  | OM453458 | Homo sapiens | China: Henan | 2020 | 20-China_Henan-122 | Ⅱ |
|  | OM453459 | Homo sapiens | China: Henan | 2020 | 20-China_Henan-126 | Ⅰ |
|  | OM453460 | Homo sapiens | China: Henan | 2020 | 20-China_Henan-13 | Ⅰ |
|  | OM453461 | Homo sapiens | China: Henan | 2020 | 20-China_Henan-130 | Ⅰ |
|  | OM453462 | Homo sapiens | China: Henan | 2020 | 20-China_Henan-133 | Ⅱ |
|  | OM453463 | Homo sapiens | China: Henan | 2020 | 20-China_Henan-134 | Ⅰ |
|  | OM453464 | Homo sapiens | China: Henan | 2020 | 20-China_Henan-137 | Ⅰ |
|  | OM453465 | Homo sapiens | China: Henan | 2020 | 20-China_Henan-138 | Ⅰ |
|  | OM453466 | Homo sapiens | China: Henan | 2020 | 20-China_Henan-14 | Ⅵ |
|  | OM453467 | Homo sapiens | China: Henan | 2020 | 20-China_Henan-140 | Ⅳ |
|  | OM453468 | Homo sapiens | China: Henan | 2020 | 20-China_Henan-142 | Ⅱ |
|  | OM453469 | Homo sapiens | China: Hubei | 2020 | 20-China_Hubei-143 | Ⅰ |
|  | OM453470 | Homo sapiens | China: Henan | 2020 | 20-China_Henan-144 | Ⅱ |
|  | OM453471 | Homo sapiens | China: Henan | 2020 | 20-China_Henan-145 | Ⅱ |
|  | OM453472 | Homo sapiens | China: Henan | 2020 | 20-China_Henan-149 | Ⅳ |
|  | OM453473 | Homo sapiens | China: Henan | 2020 | 20-China_Henan-15 | Ⅰ |
|  | OM453474 | Homo sapiens | China: Henan | 2020 | 20-China_Henan-153 | Ⅰ |
|  | OM453475 | Homo sapiens | China: Henan | 2020 | 20-China_Henan-156 | Ⅱ |
|  | OM453476 | Homo sapiens | China: Henan | 2020 | 20-China_Henan-157 | Ⅰ |
|  | OM453477 | Homo sapiens | China: Henan | 2020 | 20-China_Henan-158 | Ⅱ |
|  | OM453478 | Homo sapiens | China: Henan | 2020 | 20-China_Henan-168 | Ⅰ |
|  | OM453479 | Homo sapiens | China: Henan | 2020 | 20-China_Henan-175 | Ⅰ |
|  | OM453480 | Homo sapiens | China: Henan | 2020 | 20-China_Henan-177 | Ⅱ |
|  | OM453481 | Homo sapiens | China: Henan | 2020 | 20-China_Henan-178 | Ⅱ |
|  | OM453482 | Homo sapiens | China: Henan | 2020 | 20-China_Henan-180 | Ⅰ |
|  | OM453483 | Homo sapiens | China: Henan | 2020 | 20-China_Henan-181 | Ⅰ |
|  | OM453484 | Homo sapiens | China: Henan | 2020 | 20-China_Henan-182 | Ⅰ |
|  | OM453485 | Homo sapiens | China: Henan | 2020 | 20-China_Henan-183 | Ⅰ |
|  | OM453486 | Homo sapiens | China: Henan | 2020 | 20-China_Henan-189 | Ⅰ |
|  | OM453487 | Homo sapiens | China: Henan | 2020 | 20-China_Henan-190 | Ⅰ |
|  | OM453488 | Homo sapiens | China: Hubei | 2020 | 20-China_Hubei-191 | Ⅰ |
|  | OM453489 | Homo sapiens | China: Henan | 2020 | 20-China_Henan-192 | Ⅰ |
|  | OM453490 | Homo sapiens | China: Henan | 2020 | 20-China_Henan-198 | Ⅰ |
|  | OM453491 | Homo sapiens | China: Henan | 2020 | 20-China_Henan-20 | Ⅱ |
|  | OM453492 | Homo sapiens | China: Henan | 2020 | 20-China_Henan-200 | Ⅰ |
|  | OM453493 | Homo sapiens | China: Hubei | 2020 | 20-China_Hubei-201 | Ⅰ |
|  | OM453494 | Homo sapiens | China: Henan | 2020 | 20-China_Henan-203 | Ⅱ |
|  | OM453495 | Homo sapiens | China: Henan | 2020 | 20-China_Henan-209 | Ⅰ |
|  | OM453496 | Homo sapiens | China: Henan | 2020 | 20-China_Henan-210 | Ⅱ |
|  | OM453497 | Homo sapiens | China: Henan | 2020 | 20-China_Henan-211 | Ⅰ |
|  | OM453498 | Homo sapiens | China: Henan | 2020 | 20-China_Henan-215 | Ⅳ |
|  | OM453499 | Homo sapiens | China: Henan | 2020 | 20-China_Henan-219 | Ⅰ |
|  | OM453500 | Homo sapiens | China: Henan | 2020 | 20-China_Henan-222 | Ⅰ |
|  | OM453501 | Homo sapiens | China: Henan | 2020 | 20-China_Henan-223 | Ⅱ |
|  | OM453502 | Homo sapiens | China: Hubei | 2020 | 20-China_Hubei-228 | Ⅰ |
|  | OM453503 | Homo sapiens | China: Henan | 2020 | 20-China_Henan-230 | Ⅱ |
|  | OM453504 | Homo sapiens | China: Hubei | 2020 | 20-China_Hubei-233 | Ⅰ |
|  | OM453505 | Homo sapiens | China: Henan | 2020 | 20-China_Henan-234 | Ⅰ |
|  | OM453506 | Homo sapiens | China: Henan | 2020 | 20-China_Henan-236 | Ⅰ |
|  | OM453507 | Homo sapiens | China: Henan | 2020 | 20-China_Henan-238 | Ⅱ |
|  | OM453508 | Homo sapiens | China: Henan | 2020 | 20-China_Henan-24 | Ⅱ |
|  | OM453509 | Homo sapiens | China: Hubei | 2020 | 20-China_Hubei-240 | Ⅰ |
|  | OM453510 | Homo sapiens | China: Henan | 2020 | 20-China_Henan-244 | Ⅰ |
|  | OM453511 | Homo sapiens | China: Henan | 2020 | 20-China_Henan-246 | Ⅰ |
|  | OM453512 | Homo sapiens | China: Henan | 2020 | 20-China_Henan-249 | Ⅰ |
|  | OM453513 | Homo sapiens | China: Henan | 2020 | 20-China_Henan-252 | Ⅱ |
|  | OM453514 | Homo sapiens | China: Henan | 2020 | 20-China_Henan-254 | Ⅲ |
|  | OM453515 | Homo sapiens | China: Henan | 2020 | 20-China_Henan-255 | Ⅲ |
|  | OM453516 | Homo sapiens | China: Henan | 2020 | 20-China_Henan-28 | Ⅳ |
|  | OM453517 | Homo sapiens | China: Henan | 2020 | 20-China_Henan-3 | Ⅰ |
|  | OM453518 | Homo sapiens | China: Henan | 2020 | 20-China_Henan-37 | Ⅰ |
|  | OM453519 | Homo sapiens | China: Henan | 2020 | 20-China_Henan-4 | Ⅰ |
|  | OM453520 | Homo sapiens | China: Henan | 2020 | 20-China_Henan-40 | Ⅰ |
|  | OM453521 | Homo sapiens | China: Henan | 2020 | 20-China_Henan-41 | Ⅰ |
|  | OM453522 | Homo sapiens | China: Henan | 2020 | 20-China_Henan-42 | Ⅰ |
|  | OM453523 | Homo sapiens | China: Henan | 2020 | 20-China_Henan-43 | Ⅰ |
|  | OM453524 | Homo sapiens | China: Henan | 2020 | 20-China_Henan-48 | Ⅰ |
|  | OM453525 | Homo sapiens | China: Henan | 2020 | 20-China_Henan-49 | Ⅰ |
|  | OM453526 | Homo sapiens | China: Henan | 2020 | 20-China_Henan-51 | Ⅰ |
|  | OM453527 | Homo sapiens | China: Hubei | 2020 | 20-China_Hubei-54 | Ⅰ |
|  | OM453528 | Homo sapiens | China: Henan | 2020 | 20-China_Henan-59 | Ⅰ |
|  | OM453529 | Homo sapiens | China: Henan | 2020 | 20-China_Henan-61 | Ⅳ |
|  | OM453530 | Homo sapiens | China: Henan | 2020 | 20-China_Henan-65 | Ⅰ |
|  | OM453531 | Homo sapiens | China: Henan | 2020 | 20-China_Henan-67 | Ⅰ |
|  | OM453532 | Homo sapiens | China: Henan | 2020 | 20-China_Henan-73 | Ⅰ |
|  | OM453533 | Homo sapiens | China: Henan | 2020 | 20-China_Henan-75 | Ⅳ |
|  | OM453534 | Homo sapiens | China: Henan | 2020 | 20-China_Henan-77 | Ⅰ |
|  | OM453535 | Homo sapiens | China: Henan | 2020 | 20-China_Henan-78 | Ⅰ |
|  | OM453536 | Homo sapiens | China: Henan | 2020 | 20-China_Henan-81 | Ⅱ |
|  | OM453537 | Homo sapiens | China: Henan | 2020 | 20-China_Henan-86 | Ⅰ |
|  | OM453538 | Homo sapiens | China: Henan | 2020 | 20-China_Henan-88 | Ⅰ |
|  | OM453539 | Homo sapiens | China: Henan | 2020 | 20-China_Henan-90 | Ⅰ |
|  | OM453540 | Homo sapiens | China: Hubei | 2020 | 20-China_Hubei-92 | Ⅰ |
|  | OM453541 | Homo sapiens | China: Henan | 2020 | 20-China_Henan-93 | Ⅰ |
|  | OM453542 | Homo sapiens | China: Henan | 2020 | 20-China_Henan-99 | Ⅱ |
|  | OM453543 | Homo sapiens | China: Henan | 2019 | 19-China_Henan-126 | Ⅳ |
|  | OM453544 | Homo sapiens | China: Henan | 2019 | 19-China_Henan-129 | Ⅱ |
|  | OM453545 | Homo sapiens | China: Henan | 2019 | 19-China_Henan-132 | Ⅰ |
|  | OM453546 | Homo sapiens | China: Henan | 2019 | 19-China_Henan-133 | Ⅰ |
|  | OM453547 | Homo sapiens | China: Henan | 2019 | 19-China_Henan-137 | Ⅰ |
|  | OM453548 | Homo sapiens | China: Henan | 2019 | 19-China_Henan-139 | Ⅰ |
|  | OM453549 | Homo sapiens | China: Henan | 2019 | 19-China_Henan-148 | Ⅰ |
|  | OM453550 | Homo sapiens | China: Henan | 2019 | 19-China_Henan-156 | Ⅱ |
|  | OM453551 | Homo sapiens | China: Henan | 2019 | 19-China_Henan-160 | Ⅰ |
|  | OM453552 | Homo sapiens | China: Hubei | 2019 | 19-China_Hubei-166 | Ⅲ |
|  | OM453553 | Homo sapiens | China: Henan | 2019 | 19-China_Henan-173 | Ⅱ |
|  | OM453554 | Homo sapiens | China: Hubei | 2019 | 19-China_Hubei-174 | Ⅰ |
|  | OM453555 | Homo sapiens | China: Henan | 2019 | 19-China_Henan-175 | Ⅰ |
|  | OM453556 | Homo sapiens | China: Henan | 2019 | 19-China_Henan-177 | Ⅰ |
|  | OM453557 | Homo sapiens | China: Henan | 2019 | 19-China_Henan-179 | Ⅱ |
|  | OM453558 | Homo sapiens | China: Henan | 2019 | 19-China_Henan-183 | Ⅰ |
|  | OM453559 | Homo sapiens | China: Hubei | 2019 | 19-China_Hubei-185 | Ⅰ |
|  | OM453560 | Homo sapiens | China: Henan | 2019 | 19-China_Henan-189 | Ⅰ |
|  | OM453561 | Homo sapiens | China: Hubei | 2019 | 19-China_Hubei-195 | Ⅰ |
|  | OM453562 | Homo sapiens | China: Henan | 2019 | 19-China_Henan-197 | Ⅰ |
|  | OM453563 | Homo sapiens | China: Henan | 2019 | 19-China_Henan-198 | Ⅰ |
|  | OM453564 | Homo sapiens | China: Henan | 2019 | 19-China_Henan-199 | Ⅰ |
|  | OM453565 | Homo sapiens | China: Henan | 2019 | 19-China_Henan-201 | Ⅰ |
|  | OM453566 | Homo sapiens | China: Henan | 2019 | 19-China_Henan-208 | Ⅰ |
|  | OM453567 | Homo sapiens | China: Henan | 2019 | 19-China_Henan-214 | Ⅰ |
|  | OM453568 | Homo sapiens | China: Henan | 2019 | 19-China_Henan-215 | Ⅱ |
|  | OM453569 | Homo sapiens | China: Henan | 2019 | 19-China_Henan-217 | Ⅰ |
|  | OM453570 | Homo sapiens | China: Henan | 2019 | 19-China_Henan-223 | Ⅱ |
|  | OM453571 | Homo sapiens | China: Henan | 2019 | 19-China_Henan-227 | Ⅱ |
|  | OM453572 | Homo sapiens | China: Henan | 2019 | 19-China_Henan-232 | Ⅰ |
|  | OM453573 | Homo sapiens | China: Henan | 2019 | 19-China_Henan-235 | Ⅰ |
|  | OM453574 | Homo sapiens | China: Henan | 2019 | 19-China_Henan-236 | Ⅰ |
|  | OM453575 | Homo sapiens | China: Hubei | 2019 | 19-China_Hubei-240 | Ⅰ |
|  | OM453576 | Homo sapiens | China: Henan | 2019 | 19-China_Henan-241 | Ⅰ |
|  | OM453577 | Homo sapiens | China: Henan | 2019 | 19-China_Henan-242 | Ⅰ |
|  | OM453578 | Homo sapiens | China: Henan | 2019 | 19-China_Henan-243 | Ⅰ |
|  | OM453579 | Homo sapiens | China: Hubei | 2019 | 19-China_Hubei-249 | Ⅰ |
|  | OM453580 | Homo sapiens | China: Henan | 2019 | 19-China_Henan-253 | Ⅰ |
|  | OM453581 | Homo sapiens | China: Henan | 2019 | 19-China_Henan-254 | Ⅰ |
|  | OM453582 | Homo sapiens | China: Henan | 2019 | 19-China_Henan-259 | Ⅰ |
|  | OM453583 | Homo sapiens | China: Henan | 2019 | 19-China_Henan-260 | Ⅰ |
|  | OM453584 | Homo sapiens | China: Henan | 2019 | 19-China_Henan-261 | Ⅰ |
|  | OM453585 | Homo sapiens | China: Henan | 2019 | 19-China_Henan-262 | Ⅰ |
|  | OM453586 | Homo sapiens | China: Hubei | 2019 | 19-China_Hubei-263 | Ⅰ |
|  | OM453587 | Homo sapiens | China: Henan | 2019 | 19-China_Henan-264 | Ⅰ |
|  | OM453588 | Homo sapiens | China: Henan | 2019 | 19-China_Henan-266 | Ⅰ |
|  | OM453589 | Homo sapiens | China: Henan | 2019 | 19-China_Henan-268 | Ⅰ |
|  | OM453590 | Homo sapiens | China: Henan | 2019 | 19-China_Henan-272 | Ⅰ |
|  | OM453591 | Homo sapiens | China: Henan | 2019 | 19-China_Henan-273 | Ⅰ |
|  | OM453592 | Homo sapiens | China: Henan | 2019 | 19-China_Henan-274 | Ⅳ |
|  | OM453593 | Homo sapiens | China: Henan | 2019 | 19-China_Henan-278 | Ⅰ |
|  | OM453594 | Homo sapiens | China: Hubei | 2019 | 19-China_Hubei-279 | Ⅰ |
|  | OM453595 | Homo sapiens | China: Henan | 2019 | 19-China_Henan-280 | Ⅱ |
|  | OM453596 | Homo sapiens | China: Henan | 2019 | 19-China_Henan-285 | Ⅰ |
|  | OM453597 | Homo sapiens | China: Hubei | 2019 | 19-China_Hubei-291 | Ⅲ |
|  | OM453598 | Homo sapiens | China: Henan | 2019 | 19-China_Henan-295 | Ⅳ |
|  | OM453599 | Homo sapiens | China: Hubei | 2019 | 19-China_Hubei-297 | Ⅰ |
|  | OM453600 | Homo sapiens | China: Henan | 2019 | 19-China_Henan-298 | Ⅰ |
|  | OM453601 | Homo sapiens | China: Hubei | 2019 | 19-China_Hubei-299 | Ⅰ |
|  | OM453602 | Homo sapiens | China: Henan | 2019 | 19-China_Henan-301 | Ⅰ |
|  | OM453603 | Homo sapiens | China: Henan | 2019 | 19-China_Henan-302 | Ⅰ |
|  | OM453604 | Homo sapiens | China: Henan | 2019 | 19-China_Henan-304 | Ⅰ |
|  | OM453605 | Homo sapiens | China: Henan | 2019 | 19-China_Henan-305 | Ⅰ |
|  | OM453606 | Homo sapiens | China: Hubei | 2019 | 19-China_Hubei-306 | Ⅰ |
|  | OM453607 | Homo sapiens | China: Henan | 2019 | 19-China_Henan-308 | Ⅱ |
|  | OM453608 | Homo sapiens | China: Hubei | 2019 | 19-China_Hubei-310 | Ⅰ |
|  | OM453609 | Homo sapiens | China: Hubei | 2019 | 19-China_Hubei-314 | Ⅰ |
|  | OM453610 | Homo sapiens | China: Henan | 2019 | 19-China_Henan-321 | Ⅱ |
|  | OM453611 | Homo sapiens | China: Henan | 2019 | 19-China_Henan-322 | Ⅰ |
|  | OM453612 | Homo sapiens | China: Henan | 2019 | 19-China_Henan-323 | Ⅰ |
|  | OM453613 | Homo sapiens | China: Henan | 2019 | 19-China_Henan-324 | Ⅳ |
|  | OM453614 | Homo sapiens | China: Hubei | 2019 | 19-China_Hubei-327 | Ⅲ |
|  | OM453615 | Homo sapiens | China: Henan | 2019 | 19-China_Henan-358 | Ⅰ |
|  | OM453616 | Homo sapiens | China: Hubei | 2019 | 19-China_Hubei-377 | Ⅰ |
|  | OM453617 | Homo sapiens | China: Henan | 2019 | 19-China_Henan-382 | Ⅳ |
|  | OM453618 | Homo sapiens | China: Henan | 2019 | 19-China_Henan-388 | Ⅰ |
|  | OM453619 | Homo sapiens | China: Henan | 2020 | 20-China_Henan-12 | Ⅳ |
|  | OM453620 | Homo sapiens | China: Henan | 2020 | 20-China_Henan-115 | Ⅳ |
|  | OM453621 | Homo sapiens | China: Henan | 2020 | 20-China_Henan-139 | Ⅳ |

**Appendix Table 3. SFTSV S segment sequences available from GenBank were listed with accession number, source, location, name of isolate, and viral genotype.**

| Accession No. | Host | Location | Isolate | Genotype |
| --- | --- | --- | --- | --- |
| MF045962 | Homo sapiens | Henan | BL282 | Ⅰ |
| MF045965 | Homo sapiens | Henan | SW249 | Ⅰ |
| MF045963 | Homo sapiens | Henan | SW131 | Ⅰ |
| MF045964 | Homo sapiens | Henan | SW228 | Ⅰ |
| MF045968 | Homo sapiens | Henan | BL136 | Ⅰ |
| KP339933 | Homo sapiens | Henan | 21S | Ⅰ |
| KP339907 | Homo sapiens | Henan | 123S | Ⅰ |
| KT721305 | Homo sapiens | Henan | YSH126 | Ⅰ |
| MN510178 | Homo sapiens | Henan | 11-China_Henan-42 | Ⅰ |
| KF917433 | Homo sapiens | Henan | 2013075S | Ⅰ |
| MN510205 | Homo sapiens | Henan | 12-China_Henan-289 | Ⅰ |
| KY965090 | Homo sapiens | Hubei | HB2016-100 | Ⅰ |
| KY965075 | Homo sapiens | Hubei | HB2016-010 | Ⅰ |
| KT736100 | Homo sapiens | Henan | 2015SH242S | Ⅰ |
| KT736099 | Homo sapiens | Henan | 2015SH239S | Ⅰ |
| KT721308 | Homo sapiens | Henan | YSH147 | Ⅰ |
| KT721307 | Homo sapiens | Henan | YSH127 | Ⅰ |
| MN510193 | Homo sapiens | Henan | 12-China_Henan-100 | Ⅰ |
| KF917432 | Homo sapiens | Henan | 2013048S | Ⅰ |
| KT380657 | Homo sapiens | Henan | 2015SYSH86 | Ⅰ |
| KC292283 | Homo sapiens | Henan | HNXY_31 | Ⅰ |
| KT721295 | Homo sapiens | Henan | YSH122 | Ⅰ |
| KF917430 | Homo sapiens | Henan | 2013041S | Ⅰ |
| MN510254 | Homo sapiens | Henan | 15-China_Henan-472 | Ⅰ |
| MN510197 | Homo sapiens | Henan | 12-China_Henan-143 | Ⅰ |
| MN510180 | Homo sapiens | Henan | 11-China_Henan-251 | Ⅰ |
| KP339909 | Homo sapiens | Henan | 127S | Ⅰ |
| KC292286 | Homo sapiens | Henan | HNXY_231 | Ⅰ |
| KT721310 | Homo sapiens | Henan | YSH136 | Ⅰ |
| KF917431 | Homo sapiens | Henan | 2013043S | Ⅰ |
| MN510179 | Homo sapiens | Henan | 11-China_Henan-211 | Ⅰ |
| MT114285 | Homo sapiens | Hubei | HB2012-196 | Ⅰ |
| KT380659 | Homo sapiens | Henan | 2015SYSH73 | Ⅰ |
| KT736091 | Homo sapiens | Henan | 2015PQ42S | Ⅰ |
| HQ171191 | Homo sapiens | Henan | S-WJ | Ⅰ |
| KF917429 | Homo sapiens | Henan | 2013067S | Ⅰ |
| MK524354 | Homo sapiens | Hubei | HB2017-09 | Ⅰ |
| KC292292 | Homo sapiens | Henan | HNXY_319 | Ⅰ |
| KT721294 | Homo sapiens | Henan | YSH98 | Ⅰ |
| KT721293 | Homo sapiens | Henan | YSH119 | Ⅰ |
| KP339894 | Homo sapiens | Henan | 79S | Ⅰ |
| KT380650 | Homo sapiens | Henan | 2015SYSH16 | Ⅰ |
| KT380646 | Homo sapiens | Henan | 2015SGS5 | Ⅰ |
| KP339941 | Homo sapiens | Henan | 41S | Ⅰ |
| KP339886 | Homo sapiens | Henan | 53S | Ⅰ |
| KR075922 | Homo sapiens | Henan | 2014181S | Ⅰ |
| KR075921 | Homo sapiens | Henan | 2014178S | Ⅰ |
| KR075920 | Homo sapiens | Henan | 2014175S | Ⅰ |
| KC292291 | Homo sapiens | Henan | HNXY_2 | Ⅰ |
| KP339898 | Homo sapiens | Henan | 93S | Ⅰ |
| KP339932 | Homo sapiens | Henan | 18S | Ⅰ |
| KP339930 | Homo sapiens | Henan | 16S | Ⅰ |
| KP339937 | Homo sapiens | Henan | 33S | Ⅰ |
| KP339910 | Homo sapiens | Henan | 129S | Ⅰ |
| KP339929 | Homo sapiens | Henan | 12S | Ⅰ |
| KT721304 | Homo sapiens | Henan | YSH123 | Ⅰ |
| KP339928 | Homo sapiens | Henan | 9S | Ⅰ |
| KP339904 | Homo sapiens | Henan | 111S | Ⅰ |
| KU361341 | Homo sapiens | Zhejiang | ZL13-32 | Ⅰ |
| KY933676 | Homo sapiens | Henan | HB2014-26 | Ⅰ |
| MN147876 | Homo sapiens | Henan | HNXY2017-66 | Ⅰ |
| KT736093 | Homo sapiens | Henan | 2015SH220S | Ⅰ |
| KP339896 | Homo sapiens | Henan | 82s | Ⅰ |
| KP339888 | Homo sapiens | Henan | 62S | Ⅰ |
| KP339921 | Homo sapiens | Henan | 151S | Ⅰ |
| KP339922 | Homo sapiens | Henan | 154S | Ⅰ |
| KF917434 | Homo sapiens | Henan | 2013040S | Ⅰ |
| KT721299 | Homo sapiens | Henan | YSH138 | Ⅰ |
| KT721296 | Homo sapiens | Henan | YSH137 | Ⅰ |
| KT721311 | Homo sapiens | Henan | YSH162 | Ⅰ |
| KT721315 | Homo sapiens | Henan | YSH159 | Ⅰ |
| KT721316 | Homo sapiens | Henan | YSH161 | Ⅰ |
| KT721318 | Homo sapiens | Henan | YSH165 | Ⅰ |
| KT721317 | Homo sapiens | Henan | YSH166 | Ⅰ |
| KT721314 | Homo sapiens | Henan | YSH139 | Ⅰ |
| KP339901 | Homo sapiens | Henan | 98S | Ⅰ |
| KT721306 | Homo sapiens | Henan | YSH145 | Ⅰ |
| KP339899 | Homo sapiens | Henan | 95S | Ⅰ |
| KR075919 | Homo sapiens | Henan | 2014101S | Ⅰ |
| MT320807 | Homo sapiens | Hubei | HBSZ52 | Ⅰ |
| MT320804 | Homo sapiens | Hubei | HBXG51 | Ⅰ |
| KT380658 | Homo sapiens | Henan | 2015SYSH82 | Ⅰ |
| KT380649 | Homo sapiens | Henan | 2015SYSH10 | Ⅰ |
| KP339906 | Homo sapiens | Henan | 121S | Ⅰ |
| KT736101 | Homo sapiens | Henan | 2015SH246S | Ⅰ |
| KT380651 | Homo sapiens | Henan | 2015SYSH25 | Ⅰ |
| KT721297 | Homo sapiens | Henan | YSH101 | Ⅰ |
| KT721309 | Homo sapiens | Henan | YSH143 | Ⅰ |
| KF917427 | Homo sapiens | Henan | 2013082S | Ⅰ |
| KF917428 | Homo sapiens | Henan | 2013066S | Ⅰ |
| KP339908 | Homo sapiens | Henan | 125S | Ⅰ |
| KC292284 | Homo sapiens | Henan | HNXY_93 | Ⅰ |
| KP339889 | Homo sapiens | Henan | 65S | Ⅰ |
| KC292287 | Homo sapiens | Henan | HNXY_164 | Ⅰ |
| KC292297 | Homo sapiens | Henan | HNXY_182 | Ⅰ |
| KT380654 | Homo sapiens | Henan | 2015SYSH41 | Ⅰ |
| MN510192 | Homo sapiens | Henan | 12-China_Henan-87 | Ⅰ |
| KT380652 | Homo sapiens | Henan | 2015SYSH30 | Ⅰ |
| HQ171192 | Homo sapiens | Henan | S-HGX | Ⅰ |
| MN510189 | Homo sapiens | Henan | 12-China_Henan-55 | Ⅰ |
| MN510324 | Homo sapiens | Henan | 18-China_Henan-340 | Ⅰ |
| KT721312 | Homo sapiens | Henan | YSH152 | Ⅰ |
| KT721298 | Homo sapiens | Henan | YSH113 | Ⅰ |
| KT380648 | Homo sapiens | Henan | 2015SYSH5 | Ⅰ |
| KP339887 | Homo sapiens | Henan | 61S | Ⅰ |
| KC292288 | Homo sapiens | Henan | HNXY_207 | Ⅰ |
| KP339934 | Homo sapiens | Henan | 28S | Ⅰ |
| KP339938 | Homo sapiens | Henan | 34S | Ⅰ |
| KP339890 | Homo sapiens | Henan | 67S | Ⅰ |
| MN510201 | Homo sapiens | Henan | 12-China_Henan-265 | Ⅰ |
| KC292285 | Homo sapiens | Henan | HNXY_115 | Ⅰ |
| KF917437 | Homo sapiens | Henan | 2013037S | Ⅰ |
| HQ141597 | Homo sapiens | Henan | HN6 | Ⅰ |
| KP339905 | Homo sapiens | Henan | 116S | Ⅰ |
| KP339895 | Homo sapiens | Henan | 81S | Ⅰ |
| MT320810 | Homo sapiens | Hubei | HBHG54 | Ⅰ |
| KU738907 | Homo sapiens | Hubei | HB2014-12 | Ⅰ |
| KF917436 | Homo sapiens | Henan | 2013051S | Ⅰ |
| KF917435 | Homo sapiens | Henan | 2013049S | Ⅰ |
| KC292289 | Homo sapiens | Henan | HNXY_130 | Ⅰ |
| KC292299 | Homo sapiens | Henan | HNXY_195 | Ⅰ |
| KC292290 | Homo sapiens | Henan | HNXY_224 | Ⅰ |
| KT736090 | Homo sapiens | Henan | 2015PQ36S | Ⅰ |
| JQ670933 | Homo sapiens | Anhui | AHZ/China/2011 | Ⅰ |
| MT114269 | Homo sapiens | Liaoning | LN2012-049 | Ⅰ |
| MN510307 | Homo sapiens | Henan | 18-China_Henan-74 | Ⅰ |
| KR075913 | Homo sapiens | Henan | 2014221S | Ⅰ |
| MN510242 | Homo sapiens | Henan | 15-China_Henan-119 | Ⅰ |
| KT721313 | Homo sapiens | Henan | YSH130 | Ⅰ |
| KR230803 | Homo sapiens | Jiangsu | JS2014-33 | Ⅰ |
| KR230792 | Homo sapiens | Jiangsu | JS2012-79 | Ⅰ |
| KY362328 | Homo sapiens | Jiangsu | JS2013-55 | Ⅰ |
| KR230793 | Homo sapiens | Jiangsu | JS2013-44 | Ⅰ |
| KY362330 | Homo sapiens | Jiangsu | JS2015-26 | Ⅰ |
| KR230796 | Homo sapiens | Jiangsu | JS2013-69 | Ⅰ |
| KY362331 | Homo sapiens | Jiangsu | JS2015-36 | Ⅰ |
| KC505128 | Homo sapiens | Jiangsu | JS2011-013-1 | Ⅰ |
| KR230802 | Homo sapiens | Jiangsu | JS2014-31 | Ⅰ |
| KY362339 | Homo sapiens | Jiangsu | JS2014-06 | Ⅰ |
| KY362337 | Homo sapiens | Jiangsu | JS2013-41 | Ⅰ |
| KY362338 | Homo sapiens | Jiangsu | JS2014-04 | Ⅰ |
| KR230798 | Homo sapiens | Jiangsu | JS2014-03 | Ⅰ |
| KC505134 | Homo sapiens | Jiangsu | JS2011-034 | Ⅰ |
| KY965091 | Homo sapiens | Hubei | HB2016-106 | Ⅰ |
| MT559337 | Homo sapiens | Anhui | AHZ/2020-01 | Ⅰ |
| KY362335 | Homo sapiens | Jiangsu | JS2013-32 | Ⅰ |
| KY362336 | Homo sapiens | Jiangsu | JS2013-38 | Ⅰ |
| KR230795 | Homo sapiens | Jiangsu | JS2013-52 | Ⅰ |
| KY362325 | Homo sapiens | Jiangsu | JS2011-98 | Ⅰ |
| KY362322 | Homo sapiens | Jiangsu | JS2011-70 | Ⅰ |
| KY362324 | Homo sapiens | Jiangsu | JS2011-92 | Ⅰ |
| KY362333 | Homo sapiens | Jiangsu | JS2015-78 | Ⅰ |
| HQ141591 | Homo sapiens | Anhui | AH12 | Ⅰ |
| KR017811 | Homo sapiens | Anhui | AH-YTY/China/05/2012 | Ⅰ |
| KY965077 | Homo sapiens | Hubei | HB2016-013 | Ⅰ |
| KU738908 | Homo sapiens | Hubei | HB2014-13 | Ⅰ |
| KY965080 | Homo sapiens | Hubei | HB2016-031 | Ⅰ |
| MT114277 | Homo sapiens | Hubei | HB2011-071 | Ⅰ |
| MK513916 | Homo sapiens | Hubei | HB2015-25 | Ⅰ |
| HQ171194 | Homo sapiens | Henan | S-WWG | Ⅰ |
| MT114274 | Homo sapiens | Liaoning | LN2013-047 | Ⅰ |
| MT114275 | Homo sapiens | Liaoning | LN2013-065 | Ⅰ |
| MT114260 | Homo sapiens | Liaoning | LN2010-001 | Ⅰ |
| MT114270 | Homo sapiens | Liaoning | LN2013-019 | Ⅰ |
| MF357020 | Homo sapiens | Liaoning | DanDong202 | Ⅰ |
| MT114266 | Homo sapiens | Liaoning | LN2012-030 | Ⅰ |
| MF357023 | Homo sapiens | Liaoning | DanDong292 | Ⅰ |
| KU738911 | Homo sapiens | Hubei | HB2014-21 | Ⅰ |
| MT114262 | Homo sapiens | Liaoning | LN2011-001 | Ⅰ |
| HQ141609 | Homo sapiens | Liaoning | LN2 | Ⅰ |
| MN510288 | Homo sapiens | Hubei | 17-China_Hubei-125 | Ⅰ |
| KF917442 | Homo sapiens | Henan | 2013079S | Ⅰ |
| KF917441 | Homo sapiens | Henan | 2013077S | Ⅰ |
| KC292279 | Homo sapiens | Henan | HNXY_174 | Ⅰ |
| KF917443 | Homo sapiens | Henan | 2013052S | Ⅰ |
| MK524360 | Homo sapiens | Hubei | HB2017-24 | Ⅰ |
| MK524366 | Homo sapiens | Hubei | HB2017-49 | Ⅰ |
| MT114279 | Homo sapiens | Hubei | HB2012-171 | Ⅰ |
| KY965079 | Homo sapiens | Hubei | HB2016-021 | Ⅰ |
| KF917444 | Homo sapiens | Henan | 2013063S | Ⅰ |
| KC292280 | Homo sapiens | Henan | HNXY_144 | Ⅰ |
| KU738909 | Homo sapiens | Hubei | HB2014-15 | Ⅰ |
| KY933677 | Homo sapiens | Hubei | HB2014-33 | Ⅰ |
| MK524372 | Homo sapiens | Hubei | HB2017-53 | Ⅰ |
| MN510302 | Homo sapiens | Hubei | 17-China_Hubei-385 | Ⅰ |
| KC292282 | Homo sapiens | Henan | HNXY_262 | Ⅰ |
| MN510213 | Homo sapiens | Henan | 13-China_Henan-161 | Ⅰ |
| KT721300 | Homo sapiens | Henan | YSH96 | Ⅰ |
| MN510248 | Homo sapiens | Henan | 15-China_Henan-327 | Ⅰ |
| KC292281 | Homo sapiens | Henan | HNXY_186 | Ⅰ |
| KY440772 | Homo sapiens | Hubei | HBGS13 | Ⅰ |
| KY965084 | Homo sapiens | Hubei | HB2016-052 | Ⅰ |
| KR075915 | Homo sapiens | Henan | 2014187S | Ⅰ |
| KC292298 | Homo sapiens | Henan | HNXY_202 | Ⅰ |
| KU738906 | Homo sapiens | Hubei | HB2014-10 | Ⅰ |
| MT114289 | Homo sapiens | Hubei | HB2014-013 | Ⅰ |
| KP339927 | Homo sapiens | Henan | 6S | Ⅰ |
| KP339923 | Homo sapiens | Henan | 156S | Ⅰ |
| KR075914 | Homo sapiens | Henan | 2014186S | Ⅰ |
| KF917440 | Homo sapiens | Henan | 2013045S | Ⅰ |
| KT736095 | Homo sapiens | Henan | 2015SH224S | Ⅰ |
| KT721319 | Homo sapiens | Henan | YSH60 | Ⅰ |
| KP339885 | Homo sapiens | Henan | 48S | Ⅰ |
| KC292293 | Homo sapiens | Henan | HNXY_245 | Ⅰ |
| MN510320 | Homo sapiens | Henan | 18-China_Henan-313 | Ⅰ |
| MN510231 | Homo sapiens | Henan | 14-China_Henan-244 | Ⅰ |
| KC292276 | Homo sapiens | Henan | HNXY_293 | Ⅰ |
| KT736094 | Homo sapiens | Henan | 2015SH221S | Ⅰ |
| KP339918 | Homo sapiens | Henan | 144S | Ⅰ |
| MN510253 | Homo sapiens | Henan | 15-China_Henan-430 | Ⅰ |
| KT380653 | Homo sapiens | Henan | 2015SYSH33 | Ⅰ |
| MN510188 | Homo sapiens | Henan | 12-China_Henan-53 | Ⅰ |
| KT380644 | Homo sapiens | Henan | 2015SYPQ3 | Ⅰ |
| KT721301 | Homo sapiens | Henan | YSH107 | Ⅰ |
| KT380655 | Homo sapiens | Henan | 2015SNiB1 | Ⅰ |
| KT380645 | Homo sapiens | Henan | 2015SYPQ26 | Ⅰ |
| KC292278 | Homo sapiens | Henan | HNXY_157 | Ⅰ |
| KC292277 | Homo sapiens | Henan | HNXY_170 | Ⅰ |
| KF917439 | Homo sapiens | Henan | 2013001S | Ⅰ |
| KP339920 | Homo sapiens | Henan | 149S | Ⅰ |
| KF917438 | Homo sapiens | Henan | 2013081S | Ⅰ |
| KP339931 | Homo sapiens | Henan | 17S | Ⅰ |
| KP339940 | Homo sapiens | Henan | 39S | Ⅰ |
| KP339902 | Homo sapiens | Henan | 105S | Ⅰ |
| HQ141606 | Homo sapiens | Jiangsu | JS4 | Ⅴ |
| MT114299 | Homo sapiens | Shandong | SD2011-040 | Ⅴ |
| MT114323 | Homo sapiens | Shandong | SD2014-157 | Ⅴ |
| HM802204 | Homo sapiens | Shandong | SD4 | Ⅴ |
| MT114308 | Homo sapiens | Shandong | SD2013-054 | Ⅴ |
| MT114290 | Homo sapiens | Shandong | SD2011-006 | Ⅴ |
| KY362327 | Homo sapiens | Jiangsu | JS2013-31 | Ⅴ |
| MT114303 | Homo sapiens | Shandong | SD2012-017 | Ⅴ |
| MT114320 | Homo sapiens | Shandong | SD2014-067 | Ⅴ |
| MK524369 | Homo sapiens | Hubei | HB2017-51 | Ⅴ |
| KY965085 | Homo sapiens | Hubei | HB2016-055 | Ⅴ |
| MK513919 | Homo sapiens | Hubei | HB2015-28 | Ⅴ |
| KY965087 | Homo sapiens | Hubei | HB2016-078 | Ⅴ |
| KY965082 | Homo sapiens | Hubei | HB2016-038 | Ⅴ |
| MK513928 | Homo sapiens | Hubei | HB2015-35 | Ⅴ |
| MK513907 | Homo sapiens | Hubei | HB2015-10 | Ⅴ |
| KT736102 | Homo sapiens | Henan | 2015SH171S | Ⅱ |
| KC292275 | Homo sapiens | Henan | HNXY_206 | Ⅱ |
| KP339917 | Homo sapiens | Henan | 143S | Ⅱ |
| KT721303 | Homo sapiens | Henan | YSH116 | Ⅱ |
| MT114283 | Homo sapiens | Hubei | HB2012-182 | Ⅱ |
| KP339903 | Homo sapiens | Henan | 109S | Ⅱ |
| KP339884 | Homo sapiens | Henan | 44S | Ⅱ |
| KY965083 | Homo sapiens | Hubei | HB2016-047 | Ⅱ |
| MT114284 | Homo sapiens | Hubei | HB2012-193 | Ⅱ |
| MT320798 | Homo sapiens | Hubei | HBHG36 | Ⅱ |
| MT114313 | Homo sapiens | Shandong | SD2013-076 | Ⅱ |
| KR075912 | Homo sapiens | Henan | 2014218S | Ⅱ |
| KR075911 | Homo sapiens | Henan | 2014203S | Ⅱ |
| KR075909 | Homo sapiens | Henan | 2014206S | Ⅱ |
| KP339926 | Homo sapiens | Henan | 165S | Ⅱ |
| KP339893 | Homo sapiens | Henan | 77S | Ⅱ |
| KR075918 | Homo sapiens | Henan | 2014073S | Ⅱ |
| MT114280 | Homo sapiens | Hubei | HB2012-175 | Ⅱ |
| MT114287 | Homo sapiens | Hubei | HB2013-050 | Ⅱ |
| KT736098 | Homo sapiens | Henan | 2015SH233S | Ⅱ |
| HQ171195 | Homo sapiens | Henan | S-WJQ | Ⅱ |
| MK513925 | Homo sapiens | Hubei | HB2015-34 | Ⅱ |
| KU738910 | Homo sapiens | Hubei | HB2014-20 | Ⅱ |
| KT736092 | Homo sapiens | Henan | 2015SH218S | Ⅱ |
| KP339939 | Homo sapiens | Henan | 38S | Ⅱ |
| KP339912 | Homo sapiens | Henan | 132S | Ⅱ |
| KP339913 | Homo sapiens | Henan | 133S | Ⅱ |
| KP339915 | Homo sapiens | Henan | 135S | Ⅱ |
| KR075924 | Homo sapiens | Henan | 2014083S | Ⅱ |
| KR075923 | Homo sapiens | Henan | 2014045S | Ⅱ |
| KP339911 | Homo sapiens | Henan | 131S | Ⅱ |
| KC292274 | Homo sapiens | Henan | HNXY_188 | Ⅱ |
| KF917446 | Homo sapiens | Henan | 2013070S | Ⅱ |
| MN510187 | Homo sapiens | Henan | 12-China_Henan-52 | Ⅱ |
| HQ141600 | Homo sapiens | Henan | HN13 | Ⅱ |
| KC292296 | Homo sapiens | Henan | HNXY_191 | Ⅱ |
| MN510190 | Homo sapiens | Henan | 12-China_Henan-75 | Ⅱ |
| KR075910 | Homo sapiens | Henan | 2014197S | Ⅱ |
| KP339900 | Homo sapiens | Henan | 96S | Ⅱ |
| KP339919 | Homo sapiens | Henan | 147S | Ⅱ |
| MK513922 | Homo sapiens | Hubei | HB2015-31 | Ⅱ |
| KP339936 | Homo sapiens | Henan | 32S | Ⅱ |
| KR075917 | Homo sapiens | Henan | 2014196S | Ⅱ |
| KR075916 | Homo sapiens | Henan | 2014193S | Ⅱ |
| KP339935 | Homo sapiens | Henan | 30S | Ⅱ |
| KF917445 | Homo sapiens | Henan | 2013038S | Ⅱ |
| KT380647 | Homo sapiens | Henan | 2015SGS21 | Ⅱ |
| KT721302 | Homo sapiens | Henan | YSH111 | Ⅱ |
| KP339914 | Homo sapiens | Henan | 134S | Ⅱ |
| MN510191 | Homo sapiens | Henan | 12-China_Henan-81 | Ⅱ |
| KC292295 | Homo sapiens | Henan | HNXY_278 | Ⅱ |
| KP339892 | Homo sapiens | Henan | 74S | Ⅱ |
| KP339924 | Homo sapiens | Henan | 157S | Ⅱ |
| MN510234 | Homo sapiens | Henan | 14-China_Henan-352 | Ⅱ |
| MZ561690 | Homo sapiens | Zhejiang | ZJ2018-01 | Ⅸ |
| KR230790 | Homo sapiens | Jiangsu | JS2011-13-2 | Ⅸ |
| HQ141603 | Homo sapiens | Jiangsu | JS3 | Ⅸ |
| KC505143 | Homo sapiens | Jiangsu | JS2012-020 | Ⅸ |
| KC505125 | Homo sapiens | Jiangsu | JS2011-004 | Ⅸ |
| KY362340 | Homo sapiens | Jiangsu | JS2015-01 | Ⅸ |
| KC505146 | Homo sapiens | Jiangsu | JS2012-035 | Ⅸ |
| KC505140 | Homo sapiens | Jiangsu | JS2011-109 | Ⅸ |
| MT114292 | Homo sapiens | Shandong | SD2011-028 | Ⅸ |
| KR230797 | Homo sapiens | Jiangsu | JS2013-71 | Ⅸ |
| KR230799 | Homo sapiens | Jiangsu | JS2014-15 | Ⅸ |
| MT114272 | Homo sapiens | Liaoning | LN2013-030 | Ⅸ |
| MT114264 | Homo sapiens | Liaoning | LN2011-xq | Ⅸ |
| HQ141612 | Homo sapiens | Liaoning | LN3 | Ⅸ |
| KY362321 | Homo sapiens | Jiangsu | JS2011-46 | Ⅸ |
| KY362329 | Homo sapiens | Jiangsu | JS2014-25 | Ⅸ |
| KR230804 | Homo sapiens | Jiangsu | JS2014-39 | Ⅸ |
| MT114310 | Homo sapiens | Shandong | SD2013-056 | Ⅸ |
| MT114307 | Homo sapiens | Shandong | SD2012-081 | Ⅸ |
| MT114309 | Homo sapiens | Shandong | SD2013-055 | Ⅸ |
| KY362320 | Homo sapiens | Jiangsu | JS2011-28 | Ⅸ |
| KY362318 | Homo sapiens | Jiangsu | JS2011-106 | Ⅸ |
| KY362323 | Homo sapiens | Jiangsu | JS2011-79 | Ⅸ |
| HQ141594 | Homo sapiens | Anhui | AH15 | Ⅸ |
| KC505131 | Homo sapiens | Jiangsu | JS2011-027 | Ⅸ |
| KY362319 | Homo sapiens | Jiangsu | JS2011-19 | Ⅸ |
| KY362326 | Homo sapiens | Jiangsu | JS2012-70 | Ⅸ |
| KR230794 | Homo sapiens | Jiangsu | JS2013-46 | Ⅸ |
| MT114324 | Homo sapiens | Shandong | SD2014-163 | Ⅷ |
| MT114311 | Homo sapiens | Shandong | SD2013-058 | Ⅷ |
| KC505137 | Homo sapiens | Jiangsu | JS2011-062 | Ⅷ |
| MF357022 | Homo sapiens | Liaoning | DanDong273 | Ⅷ |
| MT114267 | Homo sapiens | Liaoning | LN2012-038 | Ⅷ |
| MF357024 | Homo sapiens | Shandong | LN2012-038 | Ⅷ |
| KY362341 | Homo sapiens | Jiangsu | JS2015-32 | Ⅷ |
| MT114261 | Homo sapiens | Liaoning | LN2010-017 | Ⅷ |
| MT114273 | Homo sapiens | Liaoning | LN2013-035 | Ⅷ |
| MT114265 | Homo sapiens | Liaoning | LN2011-pc | Ⅷ |
| MT114263 | Homo sapiens | Liaoning | LN2011-037 | Ⅷ |
| MT114268 | Homo sapiens | Liaoning | LN2012-047 | Ⅷ |
| JQ670932 | Homo sapiens | Anhui | AHL/China/2011 | Ⅷ |
| KY965074 | Homo sapiens | Hubei | HB2016-003 | Ⅷ |
| MT114306 | Homo sapiens | Shandong | SD2012-076 | Ⅷ |
| KY965078 | Homo sapiens | Hubei | HB2016-016 | Ⅷ |
| KC292273 | Homo sapiens | Henan | HNXY_212 | Ⅲ |
| KC292294 | Homo sapiens | Henan | HNXY_327 | Ⅲ |
| KY440775 | Homo sapiens | Hubei | HBMC16 | Ⅲ |
| KY440769 | Homo sapiens | Hubei | HBMC5 | Ⅲ |
| MK513931 | Homo sapiens | Hubei | HB2015-39 | Ⅲ |
| MT114316 | Homo sapiens | Shandong | SD2013-081 | Ⅳ |
| MT114315 | Homo sapiens | Shandong | SD2013-080 | Ⅳ |
| MT114297 | Homo sapiens | Shandong | SD2011-038 | Ⅳ |
| MT114326 | Homo sapiens | Shandong | SD2014-209 | Ⅳ |
| MT114318 | Homo sapiens | Shandong | SD2013-094 | Ⅳ |
| MT114304 | Homo sapiens | Shandong | SD2012-050 | Ⅳ |
| MT114327 | Homo sapiens | Shandong | SD2015-064 | Ⅳ |
| MT114288 | Homo sapiens | Hubei | HB2013-048 | Ⅳ |
| MT114296 | Homo sapiens | Shandong | SD2011-036 | Ⅳ |
| KU239113 | Homo sapiens | Shandong | SDYY104/China/2011 | Ⅳ |
| MT114301 | Homo sapiens | Shandong | SD2011-054 | Ⅳ |
| MT114328 | Homo sapiens | Shandong | SD2015-086 | Ⅳ |
| KX641918 | Homo sapiens | Shandong | SDTA_2 | Ⅳ |
| MT114298 | Homo sapiens | Shandong | SD2011-039 | Ⅳ |
| MT114291 | Homo sapiens | Shandong | SD2011-027 | Ⅳ |
| MT114294 | Homo sapiens | Shandong | SD2011-032 | Ⅳ |
| MT114319 | Homo sapiens | Shandong | SD2014-059 | Ⅳ |
| MT114302 | Homo sapiens | Shandong | SD2011-057 | Ⅳ |
| MT114325 | Homo sapiens | Shandong | SD2014-19 | Ⅳ |
| MN147875 | Homo sapiens | Henan | HNXY2017-50 | Ⅳ |
| KP339925 | Homo sapiens | Henan | 164S | Ⅳ |
| KF917448 | Homo sapiens | Henan | 2013050S | Ⅳ |
| KP339916 | Homo sapiens | Henan | 142S | Ⅳ |
| HM802205 | Homo sapiens | Shandong | SD24 | Ⅳ |
| MT114305 | Homo sapiens | Shandong | SD2012-068 | Ⅳ |
| MT114295 | Homo sapiens | Shandong | SD2011-035 | Ⅳ |
| MT114329 | Homo sapiens | Shandong | SD2015-088 | Ⅳ |
| KX641920 | Homo sapiens | Shandong | SDTA_4 | Ⅳ |
| MT114331 | Homo sapiens | Shandong | SD2015-101 | Ⅳ |
| MT114314 | Homo sapiens | Shandong | SD2013-077 | Ⅳ |
| MT114322 | Homo sapiens | Shandong | SD2014-148 | Ⅳ |
| MT114282 | Homo sapiens | Hubei | HB2012-179 | Ⅳ |
| MK524363 | Homo sapiens | Hubei | HB2017-25 | Ⅳ |
| KT736097 | Homo sapiens | Henan | 2015SH232S | Ⅳ |
| KP339891 | Homo sapiens | Henan | 72S | Ⅳ |
| KF917447 | Homo sapiens | Henan | 2013039S | Ⅳ |
| MT114300 | Homo sapiens | Shandong | SD2011-049 | Ⅳ |
| MF357025 | Homo sapiens | Shandong | TaiAn036 | Ⅳ |
| MF357026 | Homo sapiens | Shandong | TaiAn038 | Ⅳ |
| MT114321 | Homo sapiens | Shandong | SD2014-146 | Ⅳ |
| MT114293 | Homo sapiens | Shandong | SD2011-031 | Ⅳ |
| KT721320 | Homo sapiens | Henan | YSH11 | Ⅳ |
| MT320819 | Homo sapiens | Hubei | HBSZ11 | Ⅳ |
| MT114281 | Homo sapiens | Hubei | HB2012-177 | Ⅳ |
| KU738905 | Homo sapiens | Hubei | HB2014-08 | Ⅳ |
| HM745932 | Homo sapiens | Hubei | HB29 | Ⅳ |
| KT736096 | Homo sapiens | Henan | 2015SH230S | Ⅳ |
| MT114312 | Homo sapiens | Shandong | SD2013-073 | Ⅳ |
| KX641919 | Homo sapiens | Shandong | SDTA_3 | Ⅳ |
| KX641917 | Homo sapiens | Shandong | SDTA_1 | Ⅳ |
| KP339897 | Homo sapiens | Henan | 87S | Ⅳ |
| MT114330 | Homo sapiens | Shandong | SD2015-089 | Ⅳ |
| MT114276 | Homo sapiens | Hubei | HB2011-004 | Ⅳ |
| KY965089 | Homo sapiens | Hubei | HB2016-090 | Ⅳ |
| MN245798 | Homo sapiens | Jiangxi | JX2013-01 | Ⅳ |
| KY848827 | Homo sapiens | Jiangxi | SFTSV_JX2013 | Ⅳ |
| KY965076 | Homo sapiens | Hubei | HB2016-011 | Ⅳ |
| KT380656 | Homo sapiens | Henan | 2015SYSH84 | Ⅳ |
| MT320813 | Homo sapiens | Hubei | HBSZ55 | Ⅳ |
| MT320789 | Homo sapiens | Hubei | HBHG29 | Ⅳ |
| MT320792 | Homo sapiens | Hubei | HBHG30 | Ⅳ |
| MT320816 | Homo sapiens | Hubei | HBHG8 | Ⅳ |
| MT320801 | Homo sapiens | Hubei | HBHG38 | Ⅳ |
| MT320795 | Homo sapiens | Hubei | HBHG35 | Ⅳ |
| MK513904 | Homo sapiens | Hubei | HB2015-09 | Ⅵ |
| MK524357 | Homo sapiens | Hubei | HB2017-17 | Ⅵ |
| KY933681 | Homo sapiens | Hubei | HB2014-37 | Ⅵ |
| KY933678 | Homo sapiens | Hubei | HB2014-31 | Ⅵ |
| KY362334 | Homo sapiens | Jiangsu | JS2011-13-2 | Ⅵ |
| KR230800 | Homo sapiens | Jiangsu | JS2014-16 | Ⅵ |
| KY362332 | Homo sapiens | Jiangsu | JS2015-69 | Ⅵ |
| KR230791 | Homo sapiens | Jiangsu | JS2011-69 | Ⅵ |
| MT114286 | Homo sapiens | Hubei | HB2012-197 | Ⅹ |
| MN480460 | Homo sapiens | Zhejiang | LS19-02 | Ⅹ |
| MN830173 | Homo sapiens | Taiwan | TP1910a | Ⅹ |
| KY965086 | Homo sapiens | Hubei | HB2016-073 | Ⅶ |
| MK513913 | Homo sapiens | Hubei | HB2015-23 | Ⅶ |
| KY965088 | Homo sapiens | Hubei | HB2016-080 | Ⅶ |
| KY933679 | Homo sapiens | Hubei | HB2014-35 | Ⅶ |
| KY933680 | Homo sapiens | Hubei | HB2014-36 | Ⅶ |
| MN510322 | Homo sapiens | Henan | 18-China_Henan-327 | Ⅶ |

^*^Genotype determined in this present study.

**Appendix Table 4. Epidemiology, clinical manifestations, and treatment outcome of SFTS patients who had viral genome sequenced.**

|  | **All tested patients (n=1149)** | **Patients with available complete genome (n=805)** | **p** |
| --- | --- | --- | --- |
| Sex, female | 700 (60.9) | 488 (60.6) | 0.893 |
| Age, years | 63.0±11.7 | 63.0±11.8 | 0.866 |
| Time from symptom onset to admission, days | 5 (4-7) | 5 (4-7) | 0.403 |
| Reported history of tick bite | 142 (12.4) | 98 (12.2) | 0.903 |
| Underlying condition |  |  |  |
| Hypertension | 126 (11.0) | 94 (11.7) | 0.625 |
| Diabetes | 69 (6.0) | 56 (7.0) | 0.398 |
| Cerebrovascular disease | 43 (3.7) | 29 (3.6) | 0.872 |
| Chronic heart disease | 35 (3.0) | 24 (3.0) | 0.933 |
| Chronic obstructive pulmonary disease | 103 (9.0) | 77 (9.6) | 0.652 |
| Hepatitis | 115 (10.0) | 75 (9.3) | 0.612 |
| Pulmonary tuberculosis | 11 (1.0) | 9 (1.1) | 0.728 |
| Cancer | 10 (0.9) | 8 (1.0) | 0.779 |
| Clinical manifestation |  |  |  |
| Fever | 1147 (99.8) | 804 (99.9) | 0.781 |
| Feeble | 1082 (94.2) | 759 (94.3) | 0.913 |
| Dizzy | 254 (22.1) | 179 (22.2) | 0.944 |
| Headache | 153 (13.3) | 112 (13.9) | 0.704 |
| Chills | 135 (11.7) | 95 (11.8) | 0.975 |
| Myalgias | 908 (79.0) | 625 (77.6) | 0.463 |
| Arthralgia | 36 (3.1) | 22 (2.7) | 0.608 |
| Lymphadenopathy | 642 (55.9) | 458 (56.9) | 0.655 |
| Cough | 595 (51.8) | 422 (52.4) | 0.781 |
| Sputum | 465 (40.5) | 329 (40.9) | 0.860 |
| Dyspnea | 97 (8.4) | 62 (7.7) | 0.556 |
| Anorexia | 913 (79.5) | 654 (81.2) | 0.331 |
| Nausea | 552 (48.0) | 396 (49.2) | 0.616 |
| Vomit | 250 (21.8) | 178 (22.1) | 0.852 |
| Abdominal pain | 112 (9.7) | 90 (11.2) | 0.306 |
| Diarrhea | 110 (9.6) | 76 (9.4) | 0.920 |
| Macroscopic hematuria | 1 (0.1) | 1 (0.1) | 0.800 |
| Ecchymosis at venipuncture site | 5 (0.4) | 4 (0.5) | 0.843 |
| Gingival bleeding | 2 (0.2) | 2 (0.2) | 0.721 |
| Convulsion | 3 (0.3) | 3 (0.4) | 0.661 |
| Confusion | 1 (0.1) | 1 (0.1) | 0.800 |
| Death | 199 (17.3) | 139 (17.3) | 0.975 |

Data are n (%), mean (SD), and median (IQR).

**Appendix Table 5. Laboratory parameters tested on admission of SFTS patients who had S, M, or L segments sequenced.**

| **Variables** | **All tested patients (n=1149)** | **Patients with available complete genome (n=805)** | **p** |
| --- | --- | --- | --- |
| PLT, 10^9^/L | 63.0 (44.0-84.0) | 62.0 (43.0-84.0) | 0.717 |
| WBC, 10^9^/L | 2.2 (1.6-3.3) | 2.1 (1.5-3.3) | 0.620 |
| NEU percentage, % | 69.8 (59.2-78.6) | 69.8 (58.6-78.6) | 0.995 |
| LYM percentage, % | 23.2 (16.3-32.7) | 23.3 (16.3-33.3) | 0.772 |
| RBC, 10^12^/L | 4.2 (3.9-4.6) | 4.2 (3.9-4.6) | 0.951 |
| HGB, g/L | 127.0 (116.0-138.0) | 127.0 (116.0-139.0) | 0.770 |
| AST, U/L | 94.0 (52.0-227.0) | 95.0 (53.0-227.0) | 0.795 |
| ALT, U/L | 43.0 (26.0-88.0) | 41.0 (26.0-84.3) | 0.633 |
| CK, U/L | 318.5 (145.8-705.0) | 325.5 (149.0-723.8) | 0.676 |
| LDH, U/L | 424.0 (277.0-718.5) | 420.5 (277.3-729.0) | 0.972 |
| ALB, g/L | 38.5 (±4.6) | 38.3 (±4.6) | 0.505 |
| TBIL, μmol/L | 9.6 (7.2-12.4) | 9.3 (6.9-12.3) | 0.220 |
| BUN, mmol/L | 6.2 (4.7-8.4) | 6.2 (4.7-8.3) | 0.857 |
| UA, μmol/L | 254.0 (195.0-331.9) | 253.2 (194.3-337.0) | 0.979 |
| CREA, μmol/L | 77.0 (63.0-96.8) | 78.0 (64.0-97.0) | 0.554 |
| PT, S | 11.5 (10.7-12.5) | 11.7 (10.7-12.6) | 0.221 |
| APTT, S | 38.8 (32.5-49.6) | 38.3 (32.3-47.6) | 0.474 |
| TT, S | 18.6 (16.3-21.8) | 18.6 (16.3-21.6) | 0.906 |
| FIB, g/L | 2.5 (2.1-3.0) | 2.4 (2.1-3.0) | 0.433 |
| INR | 1.0 (0.9-1.1) | 1.0 (0.9-1.1) | 0.388 |

Data are n (%), mean (SD), and median (IQR).

PLT, platelet; WBC, white blood cell; NEU, neutrophil; LYM, lymphocyte; RBC, red blood cell; HGB, hemoglobin; AST, aminotransferase; ALT, aspartate aminotransferase; CK, creatine kinase; LDH, lactate dehydrogenase; ALB, albumin; TBIL, total bilirubin; BUN, blood urea nitrogen; UA, uric acid; CREA, creatinine; PT, prothrombin time; APTT, activated partial thromboplastin time; TT, thrombin time; FIB, fibrinogen; INR, international normalized ratio. Normal ranges for each variable are 100-300 109/L for PLT, 4-10 109/L for WBC, 50-70% for NEU percentage, 20-40% for LYM percentage, 3.5-5.5 1012/L for RBC, 110-170 g/L for HGB, 0-40 U/L for AST, 0-40 U/L for ALT, 25-200 U/L for CK, 109-245 U/L for LDH, 35-55 g/L for ALB, 5.11-17.10 μmol/L for TBIL, 2.86-8.20 mmol/L for BUN, 210-430 μmol/L for UA, 40-79 μmol/L for CREA, 9.6-14.0 S for 23.0-35.0 S for APTT, PT, 14.0-21.0 S for TT, 2.0-4.0 g/L for FIB, and 0.8-1.2 for INR.

**Appendix Table 6. Confirmation of the SFTSV segment reassortment by seven methods using RDP packages.**

| Recombinant sequence  (S/M/L) | Minor parental sequence  (S/M/L) | Major parental sequence  (S/M/L) | Breakpoint positions | |  | Detection Methods | | | | | | |
| --- | --- | --- | --- | --- | --- | --- | --- | --- | --- | --- | --- | --- |
|  |  |  | Begin | End |  | RDP | GENECONV | Bootscan | Maxchi | Chimaera | SiSscan | 3Seq |
| 2020-104-F (Ⅰ/Ⅰ/Ⅱ) | 2019-253-F (Ⅰ/Ⅰ/Ⅰ) | 2017-242-F (Ⅱ/Ⅱ/Ⅱ) | 6350 | 11396 |  | *** | *** | *** | *** | NS | ** | *** |
| 2020-180-F (Ⅱ/Ⅱ/Ⅰ) | 2018-326-F (Ⅰ/Ⅰ/Ⅰ) | 2014-236-F (Ⅱ/Ⅱ/Ⅱ) | 11396 | 6367 |  | *** | *** | *** | *** | * | *** | *** |
| 2020-111-F (Ⅰ/Ⅰ/Ⅲ) | 2020-233-F (Ⅰ/Ⅱ/Ⅰ)^b^ | 2020-90-F (Ⅰ/Ⅰ/Ⅰ) | 11443 | 6406 |  | *** | *** | *** | *** | * | *** | *** |
| 2020-233-F (Ⅰ/Ⅱ/Ⅰ)^a^ | 2018-279-F (Ⅰ/Ⅰ/Ⅰ) | 2015-15-F (Ⅰ/Ⅰ/Ⅳ)^b^ | 9668 | 6324 |  | *** | *** | *** | *** | NS | *** | *** |
| 2020-191-F (Ⅲ/Ⅲ/Ⅰ) | 2016-598-F (Ⅲ/Ⅲ/Ⅲ) | 2012-64-F (Ⅰ/Ⅰ/Ⅰ) | 6394 | 11460 |  | *** | *** | *** | *** | ** | *** | *** |
| 2015-16-F (Ⅳ/Ⅳ/Ⅰ) | 2012-13-F (Ⅰ/Ⅰ/Ⅰ) | 2018-164-F (Ⅳ/Ⅳ/Ⅳ) | 11446 | 6376 |  | *** | *** | *** | *** | NS | NS | * |
| ^2015-234-F (Ⅲ/Ⅲ/Ⅰ) | 2015-15-F (Ⅰ/Ⅰ/Ⅳ)^b^ | 2014-307-F (Ⅳ/Ⅳ/Ⅳ) | 123 | 6420 |  | *** | *** | *** | *** | ** | *** | *** |
| 2015-15-F (Ⅰ/Ⅰ/Ⅳ) | 2014-546-F (Ⅳ/Ⅳ/Ⅳ) | 2019-322-F (Ⅰ/Ⅰ/Ⅰ) | 11460 | 6376 |  | *** | *** | *** | *** | NS | *** | * |
| 2015-40-F (Ⅰ/Ⅰ/Ⅰ) | 2016-135-F (Ⅰ/Ⅰ/Ⅰ) | 2018-365-F (Ⅰ/Ⅰ/Ⅰ) | 6307 | 11414 |  | * | * | NS | ** | ** | ** | *** |
| 2020-40-F (Ⅰ/Ⅰ/Ⅰ) | 2019-253-F (Ⅰ/Ⅰ/Ⅰ) | 2014-607-F (Ⅰ/Ⅰ/Ⅰ) | 6388 | 11307^c^ |  | * | * | NS | * | * | * | *** |
| 2019-208-F (Ⅰ/Ⅰ/Ⅰ) | 2019-377-F (Ⅰ/Ⅰ/Ⅰ) | 2019-177-F (Ⅰ/Ⅰ/Ⅰ) | 6388 | 9834 |  | * | * | NS | * | * | NS | ** |

***p<10^-20^; **p<10^-10^; *p<10^-3^; NS=not significant (p>0.05). Minor parent, parent contributing the smaller fraction of sequence. Major parent, parent contributing the larger fraction of sequence.

^a^The recombinant sequence may have been misidentified (one of the identified parents might be the recombinant).

^b^Only one parent and a recombinant need to be in the alignment for a recombination event to be detectable. The sequence listed was used to infer the existence of a missing parental sequence.

^c^The actual breakpoint position is undetermined (it was most likely overprinted by a subsequent recombination event).

**Appendix Table 7. Geographic distribution of SFTSV clades obtained from patients residing in Xinyang city.**

| **Region** | **Clade Ⅰ**  **(n=454)** | **Clade Ⅱ**  **(n=159)** | **Clade Ⅲ**  **(n=23)** | **Clade Ⅳ**  **(n=73)** | **CFR (%)** | **R^2^** | **p^*^** |
| --- | --- | --- | --- | --- | --- | --- | --- |
| 1 | 25 (100) | 0 | 0 | 0 | 12.0 | 0.405 | 0.056^a^ |
| 2 | 32 (94.1) | 0 | 2 (5.9) | 0 | 23.5 | 0.272 | 0.209^b^ |
| 3 | 27 (83.9) | 4 (12.9) | 1 (3.2) | 0 | 6.25 | 0.088 | 0.690^c^ |
| 4 | 47 (92.1) | 1 (2.0) | 3 (5.9) | 0 | 5.9 | **0.591** | **0.003^d^** |
| 5 | 20 (74.1) | 1 (3.7) | 3 (11.1) | 3 (11.1) | 11.1 |  |  |
| 6 | 8 (32.0) | 3 (12.0) | 5 (20.0) | 10 (40.0) | 23.1 |  |  |
| 7 | 5 (25.0) | 8 (40.0) | 7 (35.0) | 0 | 10.0 |  |  |
| 8 | 4 (18.2) | 17 (77.3) | 1 (4.5) | 0 | 18.2 |  |  |
| 9 | 0 | 7 (63.6) | 0 | 4 (36.4) | 36.4 |  |  |
| 10 | 15 (40.5) | 21 (56.8) | 0 | 1 (2.7) | 16.2 |  |  |
| 11 | 8 (16.0) | 33 (66.0) | 0 | 9 (18.0) | 20.0 |  |  |
| 12 | 36 (53.7) | 17 (25.4) | 1 (1.5) | 14 (20.9) | 19.1 |  |  |
| 13 | 2 (13.3) | 9 (60.0) | 0 | 4 (26.7) | 20.0 |  |  |
| 14 | 5 (18.5) | 16 (59.3) | 0 | 6 (22.2) | 50.0 |  |  |
| 15 | 2 (14.3) | 11 (78.6) | 0 | 1 (7.1) | 0 |  |  |
| 16 | 5 (71.4) | 2 (28.6) | 0 | 0 | 14.3 |  |  |
| 17 | 14 (82.3) | 1 (5.9) | 0 | 2 (11.8) | 23.5 |  |  |
| 18 | 45 (100) | 0 | 0 | 0 | 13.3 |  |  |
| 19 | 53 (96.4) | 0 | 0 | 2 (3.6) | 27.3 |  |  |
| 20 | 77 (82.8) | 3 (3.2) | 0 | 13 (14.0) | 21.5 |  |  |
| 21 | 23 (69.7) | 6 (18.2) | 0 | 2 (12.1) | 9.1 |  |  |
| 22 | 2 (100) | 0 | 0 | 0 | 0 |  |  |
| 23 | 1 (100) | 0 | 0 | 0 | 0 |  | 0.880 |

Data are number (proportion). CFR=case fatality rate.

A total of 709 patients with SFTS residing in Xinyang city were included for analysis.

^*^Correlation between the CFR and proportion of each viral clade was analyzed by Pearson correlation test.

^a^Correlation between the CFR and proportion of clade I.

^b^Correlation between the CFR and proportion of clade II.

^c^Correlation between the CFR and proportion of clade III.

^d^Correlation between the CFR and proportion of clade IV.

**Appendix Table 8. Comparison of case fatality rates among patients infected with four virus clades.**

|  | **No. of patients** | **No. of surviving patients (%)** | **No. of fatal patients (%)** | **p** |
| --- | --- | --- | --- | --- |
| Clade Ⅰ | 521 | 434 (83.3) | 87 (16.7) | 0.001* |
| Clade Ⅱ | 160 | 138 (86.2) | 22 (13.8) | 0.001* |
| Clade Ⅲ | 34 | 30 (88.2) | 4 (11.8) | 0.021* |
| Clade Ⅳ | 73 | 49 (67.1) | 24 (32.9) | 0.002# |

^*^Comparison of case fatality rates between clade Ⅳ and each of other thee clades by χ² test or Fisher’s exact test.

^#^Comparison of case fatality rates among four clades by χ² test.

**Appendix Table 9. Survival analysis of patients infected with four viral clades.**

|  | **Log rank test** |  |  | **Cox regression** |  | | **Cox regression adjusting for age, sex, and hospitalization delay** | | | |
| --- | --- | --- | --- | --- | --- | --- | --- | --- | --- | --- |
|  | **p** |  |  | **HR (95% CI)** | **p** |  |  | | **HR (95% CI)** | **p** |
| Clade Ⅳ vs. Clade Ⅰ | 0.001 |  |  | 1.292 (1.111-1.502) | 0.001 |  |  | | 1.327 (1.140-1.545) | <0.001 |
| Clade Ⅳ vs. Clade Ⅱ | <0.001 |  |  | 1.634 (1.224-2.183) | 0.001 |  |  | | 1.697 (1.265-2.276) | <0.001 |
| Clade Ⅳ vs. Clade Ⅲ | 0.026 |  |  | 3.091 (1.072-8.911) | 0.037 |  |  | 2.916 (1.002-8.481) | | 0.048 |

HR=hazard ratio; CI=confidence interval.

**Appendix Table 10. Underlying conditions and presence of severe clinical manifestations during hospitalization among SFTS patients infected with four viral clades.**

|  | **Clade Ⅰ**  **(n=521)** | **Clade Ⅱ**  **(n=160)** | **Clade Ⅲ**  **(n=34)** | **Clade Ⅳ**  **(n=73)** | **p** |
| --- | --- | --- | --- | --- | --- |
| Underlying condition |  |  |  |  |  |
| Hypertension | 63 (12.1) | 15 (9.4) | 4 (11.8) | 8 (11.0) | 0.822 |
| Diabetes | 34 (6.5) | 13 (8.1) | 1 (2.9) | 7 (9.6) | 0.550 |
| Cerebrovascular disease | 16 (3.1) | 8 (5.0) | 0 (0.0) | 2 (2.7) | 0.429 |
| Chronic heart disease | 13 (2.5) | 7 (4.4) | 0 (0.0) | 2 (2.7) | 0.453 |
| Chronic obstructive pulmonary disease | 55 (10.6) | 10 (6.3) | 2 (5.9) | 9 (12.3) | 0.288 |
| Hepatitis | 45 (8.6) | 20 (12.5) | 3 (8.8) | 5 (6.8) | 0.432 |
| Pulmonary tuberculosis | 5 (1.0) | 3 (1.9) | 0 (0.0) | 0 (0.0) | 0.514 |
| Cancer | 4 (0.8) | 1 (0.6) | 1 (2.9) | 2 (2.7) | 0.264 |
| Clinical manifestation | 63 (12.1) | 15 (9.4) | 4 (11.8) | 8 (11.0) | 0.822 |
| Dyspnea | 33 (6.3) | 13 (8.1) | 2 (5.9) | 13 (17.8) | **0.007** |
| DIC^*^ | 8 (4.4) | 3 (5.4) | 0 (0.0) | 5 (16.7) | **0.046** |
| Hemorrhagic sings | 220 (42.2) | 67 (41.9) | 10 (29.4) | 33 (45.2) | 0.470 |
| Macroscopic hematuria | 32 (6.1) | 13 (8.1) | 2 (5.9) | 4 (5.5) | 0.812 |
| Ecchymosis at venipuncture site | 123 (23.6) | 39 (24.4) | 4 (11.8) | 19 (26.0) | 0.401 |
| Petechia | 43 (8.3) | 8 (5.0) | 2 (5.9) | 5 (6.8) | 0.561 |
| Gingival bleeding | 58 (11.1) | 22 (13.8) | 2 (5.9) | 8 (11.0) | 0.583 |
| Hemoptysis | 23 (4.4) | 6 (3.8) | 1 (2.9) | 1 (1.4) | 0.639 |
| Melena | 34 (6.5) | 4 (2.5) | 3 (8.8) | 5 (6.8) | 0.223 |
| Epistaxis | 7 (1.3) | 1 (0.6) | 2 (5.9) | 1 (1.4) | 0.129 |
| Hematemesis | 7 (1.3) | 4 (2.5) | 0 (0.0) | 2 (2.7) | 0.548 |
| Ecchymosis | 25 (4.8) | 4 (2.5) | 2 (5.9) | 2 (2.7) | 0.523 |
| Ophthalmorrhagia | 2 (0.4) | 0 (0.0) | 0 (0.0) | 0 (0.0) | 0.794 |
| Neurological symptoms | 148 (28.4) | 35 (21.9) | 3 (8.8) | 33 (45.2) | **<0.001** |
| Convulsion | 51 (9.8) | 13 (8.1) | 0 (0.0) | 14 (19.2) | **0.010** |
| Dysphoria | 77 (14.8) | 18 (11.3) | 2 (5.9) | 16 (21.9) | 0.081 |
| Confusion | 90 (17.3) | 22 (13.8) | 3 (8.8) | 23 (31.5) | **0.004** |
| Lethargy | 19 (3.6) | 6 (3.8) | 0 (0.0) | 3 (4.1) | 0.717 |
| Coma | 46 (8.8) | 8 (5.0) | 2 (5.9) | 11 (15.1) | 0.074 |

^*^A total of 286 patients had available data, including 182 patients infected with clade I, 56 infected with clade II, 12 infected with clade III, and 30 infected with clade IV. DIC=disseminated intravascular coagulation.

**Appendix Table 11. Comparison of viral loads tested during hospitalization among patients infected with four virus clades.**

| **Days since symptom onset** | **Viral load (log_10_ copies/mL)** | | | | |
| --- | --- | --- | --- | --- | --- |
|  | **Clade Ⅰ**  **(n=521)** | **Clade Ⅱ**  **(n=160)** | **Clade Ⅲ**  **(n=34)** | **Clade Ⅳ**  **(n=73)** | **p^*^** |
| 3-4 | 6.12±0.99 (n=149) | 5.92±0.83 (n=33) | 5.63±0.95  (n=8) | 6.24±0.50 (n=19) | 0.296 |
| 5-6 | 6.37±1.02 (n=345) | 6.32±0.98 (n=93) | 6.14±0.94 (n=27) | 6.70±1.01 (n=66) | **0.040** |
| 7-8 | 6.45±1.37 (n=439) | 6.39±1.39 (n=127) | 6.17±1.32 (n=31) | 6.96±1.27 (n=64) | **0.017** |
| 9-10 | 5.89±1.75 (n=344) | 5.84±1.66 (n=89) | 6.03±1.65 (n=33) | 6.53±1.62 (n=54) | 0.076 |
| 11-12 | 5.28±1.79 (n=213) | 5.33±1.57 (n=68) | 5.47±2.08 (n=14) | 5.51±1.81 (n=26) | 0.919 |
| 13-14 | 4.51±1.42 (n=120) | 4.70±1.60 (n=46) | 4.31±1.21 (n=11) | 4.76±1.71 (n=19) | 0.754 |
| 15-16 | 4.13±1.09 (n=42) | 4.37±1.34 (n=18) | 4.58±1.95  (n=2) | 4.31±1.94  (n=6) | 0.880 |

Data are mean (SD), and exact number of patients.

^*^Comparison of viral loads among four clades by ANOVA test.

**Appendix Table 12. Nucleotide substitution rates of SFTSV S, M, and L genome segments.**

|  | **S segment** | **M segment** | **L segment** | **p** |
| --- | --- | --- | --- | --- |
| Total mutation rate | 0.093 | 0.080 | 0.074 | 0.030 |
| Transition rate |  |  |  |  |
| Total | 0.827 | 0.793 | 0.811 | 0.664 |
| A-G | 0.231 | 0.350 | 0.247 | 0.011 |
| G-A | 0.276 | 0.210 | 0.239 | 0.371 |
| C-T | 0.284 | 0.229 | 0.249 | 0.519 |
| T-C | 0.209 | 0.210 | 0.265 | 0.216 |
| Transversion rate |  |  |  |  |
| Total | 0.173 | 0.207 | 0.189 | 0.664 |
| A-C | 0.036 | 0.107 | 0.067 | 0.466 |
| A-T | 0.536 | 0.375 | 0.371 | 0.271 |
| C-A | 0.036 | 0.179 | 0.157 | 0.190 |
| C-G | 0.000 | 0.000 | 0.034 | 0.237 |
| G-C | 0.000 | 0.018 | 0.034 | 0.556 |
| G-T | 0.071 | 0.071 | 0.079 | 0.984 |
| T-A | 0.250 | 0.179 | 0.236 | 0.657 |
| T-G | 0.071 | 0.071 | 0.022 | 0.309 |

**Appendix Table 13. Nucleotide mutations related to fatal outcome of patients with SFTS.**

| **Nucleotide acid sites** | **Wide**  **type** | **Mutation type** |  | **Wild type** |  | **Mutation type** |  | **Univariate regression** | |  | **Multivariate regression^*^** | |
| --- | --- | --- | --- | --- | --- | --- | --- | --- | --- | --- | --- | --- |
|  |  |  |  | **Fatal/Total (%)** |  | **Fatal/Total (%)** |  | **OR (95% CI)** | **p** |  | **OR (95% CI)** | **p** |
| **S** |  |  |  |  |  |  |  |  |  |  |  |  |
| 127 | T | C |  | 114/726 (15.7) |  | 25/79 (31.6) |  | 2.485 (1.486-4.158) | <0.001 |  | 2.938 (1.688-5.112) | <0.001 |
| 145 | A | G |  | 115/722 (15.9) |  | 24/83 (28.9) |  | 2.147 (1.283-3.592) | 0.004 |  | 2.429 (1.403-4.205) | 0.002 |
| 190 | C | T |  | 59/428 (13.8) |  | 80/376 (21.3) |  | 1.690 (1.168-2.446) | 0.005 |  | 1.675 (1.135-2.472) | 0.009 |
| 205 | A | T |  | 114/723 (15.8) |  | 25/78 (32.1) |  | 2.520 (1.504-4.221) | <0.001 |  | 2.967 (1.701-5.177) | <0.001 |
| 208 | A | T |  | 109/689 (15.8) |  | 29/112 (25.9) |  | 1.859 (1.162-2.974) | 0.010 |  | 2.121 (1.282-3.508) | 0.003 |
| 214 | G | A |  | 110/693 (15.9) |  | 29/112 (25.9) |  | 1.852 (1.158-2.961) | 0.010 |  | 2.105 (1.274-3.480) | 0.004 |
| 226 | T | A |  | 54/377 (14.3) |  | 29/116 (25.0) |  | 1.994 (1.198-3.319) | 0.008 |  | 2.150 (1.254-3.688) | 0.005 |
| 253 | T | C |  | 110/692 (15.9) |  | 29/111 (26.1) |  | 1.871 (1.170-2.994) | 0.009 |  | 2.110 (1.276-3.489) | 0.004 |
| 266 | C | T |  | 110/693 (15.9) |  | 29/112 (25.9) |  | 1.852 (1.158-2.961) | 0.010 |  | 2.114 (1.278-3.495) | 0.004 |
| 325 | T | C |  | 110/689 (16.0) |  | 29/116 (25.0) |  | 1.755 (1.100-2.799) | 0.018 |  | 1.912 (1.160-3.151) | 0.011 |
| 340 | G | A |  | 109/691 (15.8) |  | 30/113 (26.5) |  | 1.930 (1.212-3.072) | 0.006 |  | 2.116 (1.286-3.482) | 0.003 |
| 343 | G | A |  | 109/689 (15.8) |  | 30/116 (25.9) |  | 1.856 (1.168-2.950) | 0.009 |  | 2.064 (1.258-3.388) | 0.004 |
| 373 | T | C |  | 110/695 (15.8) |  | 29/109 (26.6) |  | 1.928 (1.204-3.088) | 0.006 |  | 2.274 (1.372-3.770) | 0.001 |
| 448 | A | T |  | 115/731 (15.7) |  | 24/73 (32.9) |  | 2.624 (1.548-4.446) | <0.001 |  | 3.068 (1.744-5.396) | <0.001 |
| 460 | A | G |  | 111/707 (15.7) |  | 28/98 (28.6) |  | 2.148 (1.325-3.481) | 0.002 |  | 2.418 (1.439-4.062) | <0.001 |
| 466 | T | C |  | 111/701 (15.8) |  | 28/104 (26.9) |  | 1.958 (1.214-3.160) | 0.006 |  | 2.175 (1.303-3.630) | 0.003 |
| 508 | A | T |  | 113/715 (15.8) |  | 26/90 (28.9) |  | 2.164 (1.315-3.562) | 0.002 |  | 2.367 (1.387-4.039) | 0.002 |
| 637 | G | A |  | 110/692 (15.9) |  | 29/113 (25.7) |  | 1.827 (1.143-2.919) | 0.012 |  | 2.021 (1.224-3.337) | 0.006 |
| 647 | T | C |  | 115/731 (15.7) |  | 24/74 (32.4) |  | 2.571 (1.520-4.350) | <0.001 |  | 2.995 (1.704-5.264) | <0.001 |
| 667 | G | A |  | 110/691 (15.9) |  | 24/76 (31.6) |  | 2.438 (1.442-4.120) | <0.001 |  | 2.786 (1.587-4.891) | <0.001 |
| 670 | A | G |  | 70/469 (14.9) |  | 69/336 (20.5) |  | 1.473 (1.021-2.126) | 0.039 |  | 1.543 (1.047-2.274) | 0.028 |
| 695 | A | G |  | 110/693 (15.9) |  | 29/111 (26.1) |  | 1.874 (1.172-2.999) | 0.009 |  | 2.114 (1.278-3.496) | 0.004 |
| 721 | A | T |  | 110/692 (15.9) |  | 29/112 (25.9) |  | 1.849 (1.156-2.956) | 0.010 |  | 2.094 (1.267-3.459) | 0.004 |
| 745 | T | G |  | 128/773 (16.6) |  | 8/15 (53.3) |  | 5.759 (2.052-16.163) | <0.001 |  | 5.480 (1.834-16.376) | 0.002 |
| 754 | G | A |  | 113/718 (15.7) |  | 26/87 (29.9) |  | 2.282 (1.383-3.766) | 0.001 |  | 2.691 (1.570-4.612) | <0.001 |
| 763 | A | T |  | 93/569 (16.3) |  | 24/74 (32.4) |  | 2.457 (1.439-4.195) | <0.001 |  | 2.842 (1.601-5.046) | <0.001 |
| 773 | T | C |  | 110/691 (15.9) |  | 29/112 (25.9) |  | 1.845 (1.154-2.951) | 0.011 |  | 2.097 (1.269-3.466) | 0.004 |
| 805 | A | T |  | 110/694 (15.9) |  | 29/110 (26.4) |  | 1.901 (1.187-3.043) | 0.007 |  | 2.141 (1.294-3.543) | 0.003 |
| 1034 | T | C |  | 110/689 (16.0) |  | 29/116 (25.0) |  | 1.755 (1.100-2.799) | 0.018 |  | 1.939 (1.177-3.193) | 0.009 |
| 1055 | C | T |  | 110/694 (15.9) |  | 29/110 (26.4) |  | 1.901 (1.187-3.043) | 0.007 |  | 2.129 (1.287-3.522) | 0.003 |
| 1100 | G | A |  | 110/691 (15.9) |  | 29/111 (26.1) |  | 1.868 (1.168-2.989) | 0.009 |  | 2.118 (1.280-3.504) | 0.003 |
| 1196 | A | G |  | 108/687 (15.7) |  | 30/117 (25.6) |  | 1.849 (1.163-2.937) | 0.009 |  | 2.046 (1.248-3.356) | 0.005 |
| 1319 | C | T |  | 111/694 (16.0) |  | 28/109 (25.7) |  | 1.816 (1.129-2.920) | 0.014 |  | 2.018 (1.215-3.351) | 0.007 |
| 1339 | A | G |  | 110/693 (15.9) |  | 29/112 (25.9) |  | 1.852 (1.158-2.961) | 0.010 |  | 2.111 (1.277-3.491) | 0.004 |
| 1340 | G | A |  | 115/730 (15.8) |  | 24/75 (32.0) |  | 2.517 (1.490-4.252) | <0.001 |  | 2.945 (1.678-5.167) | <0.001 |
| 1349 | T | A |  | 109/690 (15.8) |  | 30/113 (26.5) |  | 1.927 (1.210-3.067) | 0.006 |  | 2.145 (1.303-3.529) | 0.003 |
| 1382 | T | A |  | 109/692 (15.8) |  | 29/110 (26.4) |  | 1.915 (1.196-3.067) | 0.007 |  | 2.172 (1.311-3.599) | 0.003 |
| 1463 | A | G |  | 55/383 (14.4) |  | 29/115 (25.2) |  | 2.011 (1.209-3.344) | 0.007 |  | 2.185 (1.273-3.750) | 0.005 |
| 1541 | A | T |  | 87/519 (16.8) |  | 30/115 (26.1) |  | 1.753 (1.089-2.820) | 0.021 |  | 1.999 (1.200-3.330) | 0.008 |
| 1601 | A | G |  | 108/687 (15.7) |  | 31/118 (26.3) |  | 1.910 (1.208-3.022) | 0.006 |  | 2.164 (1.322-3.542) | 0.002 |
| 1634 | A | G |  | 115/732 (15.7) |  | 24/73 (32.9) |  | 2.628 (1.551-4.453) | <0.001 |  | 3.216 (1.822-5.676) | <0.001 |
| 1697 | T | C |  | 112/710 (15.8) |  | 27/95 (28.4) |  | 2.120 (1.300-3.458) | 0.003 |  | 2.403 (1.419-4.071) | 0.001 |
| **M** |  |  |  |  |  |  |  |  |  |  |  |  |
| 128 | A | G |  | 110/697 (15.8) |  | 29/108 (26.9) |  | 1.959 (1.222-3.140) | 0.005 |  | 2.181 (1.316-3.613) | 0.002 |
| 204 | G | A |  | 84/409 (20.5) |  | 55/396 (13.9) |  | 0.624 (0.430-0.906) | 0.013 |  | 0.630 (0.426-0.933) | 0.021 |
| 213 | A | G |  | 109/696 (15.7) |  | 29/108 (26.9) |  | 1.977 (1.233-3.170) | 0.005 |  | 2.192 (1.324-3.630) | 0.002 |
| 222 | G | A |  | 110/695 (15.8) |  | 29/109 (26.6) |  | 1.928 (1.204-3.088) | 0.006 |  | 2.127 (1.286-3.518) | 0.003 |
| 231 | A | T |  | 114/724 (15.7) |  | 24/75 (32.0) |  | 2.518 (1.490-4.256) | <0.001 |  | 2.909 (1.659-5.101) | <0.001 |
| 258 | G | T |  | 114/728 (15.7) |  | 24/75 (32.0) |  | 2.535 (1.500-4.283) | <0.001 |  | 2.925 (1.668-5.129) | <0.001 |
| 276 | A | G |  | 110/696 (15.8) |  | 29/109 (26.6) |  | 1.931 (1.206-3.093) | 0.006 |  | 2.155 (1.302-3.567) | 0.003 |
| 288 | A | G |  | 110/695 (15.8) |  | 29/109 (26.6) |  | 1.928 (1.204-3.088) | 0.006 |  | 2.136 (1.291-3.533) | 0.003 |
| 300 | G | A |  | 109/695 (15.7) |  | 30/110 (27.3) |  | 2.016 (1.264-3.215) | 0.003 |  | 2.181 (1.324-3.592) | 0.002 |
| 359 | G | A |  | 115/729 (15.8) |  | 24/76 (31.6) |  | 2.464 (1.461-4.158) | <0.001 |  | 2.885 (1.647-5.054) | <0.001 |
| 504 | T | A |  | 115/728 (15.8) |  | 24/74 (32.4) |  | 2.559 (1.512-4.329) | <0.001 |  | 2.985 (1.698-5.248) | <0.001 |
| 636 | C | T |  | 108/693 (15.6) |  | 31/112 (27.7) |  | 2.073 (1.306-3.291) | 0.002 |  | 2.279 (1.389-3.739) | 0.001 |
| 729 | A | G |  | 115/730 (15.8) |  | 24/75 (32.0) |  | 2.517 (1.490-4.252) | <0.001 |  | 2.951 (1.681-5.180) | <0.001 |
| 810 | G | A |  | 110/694 (15.9) |  | 29/111 (26.1) |  | 1.878 (1.174-3.004) | 0.009 |  | 2.081 (1.260-3.436) | 0.004 |
| 835 | A | G |  | 110/692 (15.9) |  | 29/113 (25.7) |  | 1.827 (1.143-2.919) | 0.012 |  | 2.034 (1.233-3.356) | 0.005 |
| 840 | C | T |  | 112/713 (15.7) |  | 27/92 (29.3) |  | 2.229 (1.363-3.646) | 0.001 |  | 2.518 (1.487-4.266) | <0.001 |
| 861 | G | A |  | 115/731 (15.7) |  | 24/74 (32.4) |  | 2.571 (1.520-4.350) | <0.001 |  | 2.854 (1.627-5.006) | <0.001 |
| 894 | T | C |  | 115/731 (15.7) |  | 24/74 (32.4) |  | 2.571 (1.520-4.350) | <0.001 |  | 2.995 (1.704-5.264) | <0.001 |
| 903 | G | A |  | 115/729 (15.8) |  | 24/75 (32.0) |  | 2.513 (1.487-4.245) | <0.001 |  | 2.941 (1.675-5.166) | <0.001 |
| 906 | A | G |  | 110/694 (15.9) |  | 29/111 (26.1) |  | 1.878 (1.174-3.004) | 0.009 |  | 2.119 (1.282-3.504) | 0.003 |
| 918 | A | G |  | 115/728 (15.8) |  | 24/76 (31.6) |  | 2.460 (1.458-4.151) | <0.001 |  | 2.888 (1.649-5.058) | <0.001 |
| 951 | C | T |  | 86/526 (16.3) |  | 31/117 (26.5) |  | 1.844 (1.151-2.954) | 0.011 |  | 1.991 (1.203-3.294) | 0.007 |
| 1002 | T | C |  | 115/731 (15.7) |  | 24/74 (32.4) |  | 2.571 (1.520-4.350) | <0.001 |  | 2.995 (1.704-5.264) | <0.001 |
| 1040 | A | C |  | 109/691 (15.8) |  | 30/114 (26.3) |  | 1.907 (1.199-3.034) | 0.006 |  | 2.112 (1.284-3.472) | 0.003 |
| 1041 | A | T |  | 103/603 (17.1) |  | 11/32 (34.4) |  | 2.543 (1.190-5.436) | 0.016 |  | 2.524 (1.127-5.654) | 0.024 |
| 1130 | A | G |  | 115/730 (15.8) |  | 24/75 (32.0) |  | 2.517 (1.490-4.252) | <0.001 |  | 2.972 (1.692-5.220) | <0.001 |
| 1187 | G | A |  | 129/776 (16.6) |  | 10/29 (34.5) |  | 2.640 (1.200-5.809) | 0.016 |  | 2.625 (1.133-6.082) | 0.024 |
| 1200 | A | T |  | 110/692 (15.9) |  | 28/106 (26.4) |  | 1.899 (1.178-3.062) | 0.008 |  | 2.178 (1.307-3.629) | 0.003 |
| 1233 | A | T |  | 109/693 (15.7) |  | 29/110 (26.4) |  | 1.918 (1.198-3.072) | 0.007 |  | 2.141 (1.295-3.540) | 0.003 |
| 1239 | C | T |  | 109/685 (15.9) |  | 30/120 (25.0) |  | 1.761 (1.111-2.793) | 0.016 |  | 1.947 (1.192-3.178) | 0.008 |
| 1305 | T | A |  | 108/688 (15.7) |  | 29/111 (26.1) |  | 1.899 (1.186-3.041) | 0.008 |  | 2.131 (1.288-3.525) | 0.003 |
| 1353 | C | T |  | 110/689 (16.0) |  | 28/114 (24.6) |  | 1.714 (1.068-2.749) | 0.026 |  | 1.965 (1.186-3.258) | 0.009 |
| 1423 | C | T |  | 116/732 (15.8) |  | 23/73 (31.5) |  | 2.443 (1.435-4.159) | 0.001 |  | 2.828 (1.600-4.997) | <0.001 |
| 1455 | T | C |  | 110/691 (15.9) |  | 28/110 (25.5) |  | 1.804 (1.122-2.900) | 0.015 |  | 2.031 (1.222-3.375) | 0.006 |
| 1470 | C | A |  | 111/695 (16.0) |  | 22/72 (30.6) |  | 2.315 (1.348-3.976) | 0.002 |  | 2.674 (1.500-4.765) | <0.001 |
| 1534 | A | G |  | 111/695 (16.0) |  | 28/110 (25.5) |  | 1.797 (1.118-2.888) | 0.016 |  | 2.018 (1.216-3.351) | 0.007 |
| 1591 | A | G |  | 111/691 (16.1) |  | 28/114 (24.6) |  | 1.701 (1.061-2.728) | 0.027 |  | 1.944 (1.174-3.221) | 0.010 |
| 1593 | A | G |  | 111/695 (16.0) |  | 28/110 (25.5) |  | 1.797 (1.118-2.888) | 0.016 |  | 2.018 (1.216-3.351) | 0.007 |
| 1638 | A | G |  | 84/416 (20.2) |  | 55/389 (14.1) |  | 0.651 (0.448-0.944) | 0.024 |  | 0.661 (0.447-0.979) | 0.039 |
| 1677 | T | C |  | 111/689 (16.1) |  | 28/115 (24.3) |  | 1.676 (1.046-2.686) | 0.032 |  | 1.885 (1.140-3.117) | 0.013 |
| 1749 | G | A |  | 116/732 (15.8) |  | 23/73 (31.5) |  | 2.443 (1.435-4.159) | 0.001 |  | 2.828 (1.600-4.997) | <0.001 |
| 1776 | T | C |  | 110/694 (15.9) |  | 29/111 (26.1) |  | 1.878 (1.174-3.004) | 0.009 |  | 2.119 (1.282-3.504) | 0.003 |
| 1777 | G | A |  | 115/731 (15.7) |  | 24/74 (32.4) |  | 2.571 (1.520-4.350) | <0.001 |  | 2.995 (1.704-5.264) | <0.001 |
| 1842 | G | A |  | 114/716 (15.9) |  | 25/89 (28.1) |  | 2.063 (1.246-3.414) | 0.005 |  | 2.384 (1.395-4.074) | 0.001 |
| 1884 | T | C |  | 112/703 (15.9) |  | 25/99 (25.3) |  | 1.783 (1.085-2.929) | 0.022 |  | 1.941 (1.144-3.294) | 0.014 |
| 1977 | T | G |  | 86/516 (16.7) |  | 29/110 (26.4) |  | 1.790 (1.104-2.902) | 0.018 |  | 1.992 (1.186-3.344) | 0.009 |
| 2064 | A | T |  | 110/695 (15.8) |  | 29/110 (26.4) |  | 1.904 (1.189-3.048) | 0.007 |  | 2.151 (1.299-3.560) | 0.003 |
| 2154 | A | G |  | 110/694 (15.9) |  | 29/109 (26.6) |  | 1.925 (1.201-3.083) | 0.006 |  | 2.116 (1.278-3.502) | 0.004 |
| 2184 | A | G |  | 115/731 (15.7) |  | 24/74 (32.4) |  | 2.571 (1.520-4.350) | <0.001 |  | 2.995 (1.704-5.264) | <0.001 |
| 2193 | T | C |  | 110/696 (15.8) |  | 29/109 (26.6) |  | 1.931 (1.206-3.093) | 0.006 |  | 2.160 (1.305-3.577) | 0.003 |
| 2241 | G | T |  | 109/690 (15.8) |  | 29/110 (26.4) |  | 1.908 (1.192-3.056) | 0.007 |  | 2.157 (1.303-3.573) | 0.003 |
| 2283 | A | G |  | 110/693 (15.9) |  | 29/112 (25.9) |  | 1.852 (1.158-2.961) | 0.010 |  | 2.069 (1.253-3.416) | 0.004 |
| 2376 | G | A |  | 115/731 (15.7) |  | 24/74 (32.4) |  | 2.571 (1.520-4.350) | <0.001 |  | 2.995 (1.704-5.264) | <0.001 |
| 2457 | A | G |  | 109/691 (15.8) |  | 30/114 (26.3) |  | 1.907 (1.199-3.034) | 0.006 |  | 2.166 (1.317-3.561) | 0.002 |
| 2553 | C | T |  | 110/692 (15.9) |  | 29/111 (26.1) |  | 1.871 (1.170-2.994) | 0.009 |  | 2.112 (1.277-3.492) | 0.004 |
| 2685 | T | C |  | 108/689 (15.7) |  | 31/116 (26.7) |  | 1.962 (1.239-3.107) | 0.004 |  | 2.212 (1.351-3.619) | 0.002 |
| 2713 | C | T |  | 110/696 (15.8) |  | 29/109 (26.6) |  | 1.931 (1.206-3.093) | 0.006 |  | 2.176 (1.314-3.603) | 0.003 |
| 2715 | G | A |  | 115/721 (16.0) |  | 24/84 (28.6) |  | 2.108 (1.261-3.523) | 0.004 |  | 2.466 (1.424-4.271) | 0.001 |
| 2778 | T | C |  | 115/730 (15.8) |  | 24/75 (32.0) |  | 2.517 (1.490-4.252) | <0.001 |  | 2.967 (1.690-5.211) | <0.001 |
| 2841 | A | T |  | 110/692 (15.9) |  | 29/109 (26.6) |  | 1.918 (1.197-3.072) | 0.007 |  | 2.163 (1.305-3.585) | 0.003 |
| 2897 | C | T |  | 115/731 (15.7) |  | 24/74 (32.4) |  | 2.571 (1.520-4.350) | <0.001 |  | 2.995 (1.704-5.264) | <0.001 |
| 2904 | T | C |  | 109/693 (15.7) |  | 30/112 (26.8) |  | 1.960 (1.231-3.122) | 0.005 |  | 2.175 (1.321-3.583) | 0.002 |
| 2910 | T | C |  | 110/696 (15.8) |  | 29/109 (26.6) |  | 1.931 (1.206-3.093) | 0.006 |  | 2.177 (1.315-3.604) | 0.003 |
| 2937 | G | A |  | 115/731 (15.7) |  | 24/74 (32.4) |  | 2.571 (1.520-4.350) | <0.001 |  | 2.995 (1.704-5.264) | <0.001 |
| 2943 | A | G |  | 115/723 (15.9) |  | 24/81 (29.6) |  | 2.226 (1.328-3.733) | 0.002 |  | 2.544 (1.465-4.417) | <0.001 |
| 2979 | A | T |  | 90/561 (16.0) |  | 25/76 (32.9) |  | 2.565 (1.512-4.354) | <0.001 |  | 2.992 (1.696-5.279) | <0.001 |
| 3045 | T | C |  | 109/693 (15.7) |  | 30/112 (26.8) |  | 1.960 (1.231-3.122) | 0.005 |  | 2.133 (1.296-3.509) | 0.003 |
| 3057 | C | A |  | 116/727 (16.0) |  | 23/73 (31.5) |  | 2.423 (1.423-4.126) | 0.001 |  | 2.790 (1.578-4.930) | <0.001 |
| 3185 | C | T |  | 113/700 (16.1) |  | 26/104 (25.0) |  | 1.732 (1.064-2.819) | 0.027 |  | 2.003 (1.189-3.373) | 0.009 |
| 3190 | T | C |  | 116/728 (15.9) |  | 23/77 (29.9) |  | 2.247 (1.327-3.806) | 0.003 |  | 2.607 (1.483-4.583) | <0.001 |
| 3228 | G | A |  | 84/413 (20.3) |  | 55/392 (14.0) |  | 0.639 (0.440-0.928) | 0.018 |  | 0.636 (0.430-0.941) | 0.024 |
| 3261 | A | G |  | 64/445 (14.4) |  | 75/359 (20.9) |  | 1.572 (1.089-2.270) | 0.016 |  | 1.604 (1.088-2.363) | 0.017 |
| 3308 | C | T |  | 111/695 (16.0) |  | 28/110 (25.5) |  | 1.797 (1.118-2.888) | 0.016 |  | 1.990 (1.198-3.304) | 0.008 |
| 3309 | A | T |  | 112/696 (16.1) |  | 27/109 (24.8) |  | 1.717 (1.063-2.774) | 0.027 |  | 1.869 (1.121-3.115) | 0.016 |
| **L** |  |  |  |  |  |  |  |  |  |  |  |  |
| 103 | C | T |  | 112/697 (16.1) |  | 27/108 (25.0) |  | 1.741 (1.077-2.814) | 0.024 |  | 1.991 (1.192-3.326) | 0.009 |
| 115 | C | T |  | 90/538 (16.7) |  | 26/99 (26.3) |  | 1.773 (1.074-2.928) | 0.025 |  | 2.018 (1.177-3.459) | 0.011 |
| 145 | A | T |  | 117/733 (16.0) |  | 22/72 (30.6) |  | 2.317 (1.351-3.971) | 0.002 |  | 2.750 (1.543-4.899) | <0.001 |
| 151 | G | A |  | 113/698 (16.2) |  | 26/107 (24.3) |  | 1.662 (1.023-2.700) | 0.040 |  | 1.847 (1.102-3.097) | 0.020 |
| 191 | C | T |  | 112/697 (16.1) |  | 27/108 (25.0) |  | 1.741 (1.077-2.814) | 0.024 |  | 1.983 (1.187-3.312) | 0.009 |
| 196 | T | C |  | 112/696 (16.1) |  | 27/109 (24.8) |  | 1.717 (1.063-2.774) | 0.027 |  | 1.942 (1.164-3.239) | 0.011 |
| 208 | T | C |  | 116/733 (15.8) |  | 23/72 (31.9) |  | 2.497 (1.464-4.257) | <0.001 |  | 2.871 (1.621-5.084) | <0.001 |
| 235 | T | A |  | 115/725 (15.9) |  | 23/74 (31.1) |  | 2.392 (1.407-4.069) | 0.001 |  | 2.553 (1.445-4.509) | 0.001 |
| 238 | A | G |  | 112/698 (16.0) |  | 27/107 (25.2) |  | 1.766 (1.092-2.856) | 0.020 |  | 1.998 (1.196-3.339) | 0.008 |
| 364 | C | A |  | 116/727 (16.0) |  | 23/75 (30.7) |  | 2.330 (1.372-3.956) | 0.002 |  | 2.716 (1.541-4.786) | <0.001 |
| 481 | G | A |  | 116/729 (15.9) |  | 23/76 (30.3) |  | 2.293 (1.352-3.889) | 0.002 |  | 2.640 (1.501-4.645) | <0.001 |
| 691 | C | T |  | 116/729 (15.9) |  | 23/76 (30.3) |  | 2.293 (1.352-3.889) | 0.002 |  | 2.673 (1.518-4.705) | <0.001 |
| 713 | T | C |  | 112/695 (16.1) |  | 27/110 (24.5) |  | 1.693 (1.049-2.734) | 0.031 |  | 1.917 (1.150-3.194) | 0.013 |
| 742 | A | G |  | 111/693 (16.0) |  | 28/112 (25.0) |  | 1.748 (1.089-2.806) | 0.021 |  | 1.936 (1.168-3.209) | 0.010 |
| 754 | T | C |  | 104/658 (15.8) |  | 35/147 (23.8) |  | 1.665 (1.079-2.568) | 0.021 |  | 1.877 (1.183-2.980) | 0.008 |
| 760 | A | T |  | 116/731 (15.9) |  | 22/72 (30.6) |  | 2.333 (1.360-4.000) | 0.002 |  | 2.673 (1.504-4.750) | <0.001 |
| 862 | T | C |  | 112/694 (16.1) |  | 27/111 (24.3) |  | 1.670 (1.035-2.695) | 0.036 |  | 1.912 (1.148-3.184) | 0.013 |
| 868 | C | T |  | 112/695 (16.1) |  | 27/110 (24.5) |  | 1.693 (1.049-2.734) | 0.031 |  | 1.914 (1.149-3.188) | 0.013 |
| 892 | C | T |  | 112/695 (16.1) |  | 27/110 (24.5) |  | 1.693 (1.049-2.734) | 0.031 |  | 1.959 (1.174-3.268) | 0.010 |
| 931 | T | A |  | 112/694 (16.1) |  | 27/111 (24.3) |  | 1.670 (1.035-2.695) | 0.036 |  | 1.897 (1.139-3.158) | 0.014 |
| 949 | C | A |  | 112/694 (16.1) |  | 27/111 (24.3) |  | 1.670 (1.035-2.695) | 0.036 |  | 1.898 (1.140-3.160) | 0.014 |
| 967 | A | G |  | 112/697 (16.1) |  | 27/108 (25.0) |  | 1.741 (1.077-2.814) | 0.024 |  | 2.000 (1.197-3.342) | 0.008 |
| 1015 | G | A |  | 113/703 (16.1) |  | 26/102 (25.5) |  | 1.786 (1.096-2.912) | 0.020 |  | 2.057 (1.220-3.468) | 0.007 |
| 1042 | G | A |  | 121/748 (16.2) |  | 18/57 (31.6) |  | 2.392 (1.324-4.321) | 0.004 |  | 2.654 (1.410-4.993) | 0.002 |
| 1078 | C | T |  | 114/726 (15.7) |  | 25/79 (31.6) |  | 2.485 (1.486-4.158) | <0.001 |  | 2.878 (1.658-4.994) | <0.001 |
| 1081 | A | G |  | 110/691 (15.9) |  | 29/114 (25.4) |  | 1.802 (1.128-2.878) | 0.014 |  | 2.023 (1.226-3.335) | 0.006 |
| 1135 | G | A |  | 115/728 (15.8) |  | 24/76 (31.6) |  | 2.460 (1.458-4.151) | <0.001 |  | 2.816 (1.611-4.922) | <0.001 |
| 1177 | A | G |  | 111/691 (16.1) |  | 28/114 (24.6) |  | 1.701 (1.061-2.728) | 0.027 |  | 1.950 (1.178-3.228) | 0.009 |
| 1189 | C | T |  | 109/686 (15.9) |  | 28/110 (25.5) |  | 1.808 (1.124-2.908) | 0.015 |  | 2.127 (1.279-3.539) | 0.004 |
| 1312 | G | A |  | 113/727 (15.5) |  | 26/78 (33.3) |  | 2.717 (1.629-4.532) | <0.001 |  | 3.138 (1.814-5.429) | <0.001 |
| 1393 | A | T |  | 88/528 (16.7) |  | 29/114 (25.4) |  | 1.706 (1.056-2.756) | 0.029 |  | 1.898 (1.136-3.170) | 0.014 |
| 1426 | A | T |  | 111/695 (16.0) |  | 28/109 (25.7) |  | 1.819 (1.131-2.925) | 0.014 |  | 2.086 (1.254-3.467) | 0.005 |
| 1510 | C | T |  | 115/725 (15.9) |  | 24/80 (30.0) |  | 2.273 (1.354-3.816) | 0.002 |  | 2.586 (1.489-4.491) | <0.001 |
| 1525 | C | T |  | 109/689 (15.8) |  | 30/116 (25.9) |  | 1.856 (1.168-2.950) | 0.009 |  | 2.079 (1.268-3.410) | 0.004 |
| 1561 | T | C |  | 111/692 (16.0) |  | 28/111 (25.2) |  | 1.766 (1.099-2.837) | 0.019 |  | 1.988 (1.199-3.297) | 0.008 |
| 1713 | A | T |  | 111/694 (16.0) |  | 28/111 (25.2) |  | 1.772 (1.103-2.846) | 0.018 |  | 2.034 (1.226-3.376) | 0.006 |
| 1762 | A | G |  | 109/687 (15.9) |  | 30/118 (25.4) |  | 1.808 (1.139-2.870) | 0.012 |  | 1.998 (1.219-3.275) | 0.006 |
| 1850 | T | C |  | 111/694 (16.0) |  | 28/110 (25.5) |  | 1.793 (1.116-2.883) | 0.016 |  | 2.044 (1.231-3.394) | 0.006 |
| 1895 | C | T |  | 111/694 (16.0) |  | 28/111 (25.2) |  | 1.772 (1.103-2.846) | 0.018 |  | 2.001 (1.205-3.323) | 0.007 |
| 1942 | G | A |  | 110/691 (15.9) |  | 29/114 (25.4) |  | 1.802 (1.128-2.878) | 0.014 |  | 1.959 (1.190-3.227) | 0.008 |
| 1967 | A | G |  | 112/700 (16.0) |  | 27/105 (25.7) |  | 1.817 (1.122-2.943) | 0.015 |  | 2.098 (1.256-3.506) | 0.005 |
| 1987 | T | C |  | 110/692 (15.9) |  | 28/109 (25.7) |  | 1.829 (1.137-2.943) | 0.013 |  | 2.058 (1.239-3.419) | 0.005 |
| 2044 | T | C |  | 111/699 (15.9) |  | 27/105 (25.7) |  | 1.834 (1.132-2.970) | 0.014 |  | 2.034 (1.216-3.400) | 0.007 |
| 2131 | G | A |  | 114/730 (15.6) |  | 25/75 (33.3) |  | 2.702 (1.606-4.545) | <0.001 |  | 3.090 (1.772-5.388) | <0.001 |
| 2172 | G | A |  | 114/726 (15.7) |  | 25/79 (31.6) |  | 2.485 (1.486-4.158) | <0.001 |  | 2.758 (1.595-4.772) | <0.001 |
| 2174 | C | T |  | 112/699 (16.0) |  | 27/106 (25.5) |  | 1.791 (1.107-2.899) | 0.018 |  | 2.013 (1.204-3.366) | 0.008 |
| 2329 | T | C |  | 115/728 (15.8) |  | 24/77 (31.2) |  | 2.414 (1.433-4.067) | <0.001 |  | 2.726 (1.563-4.755) | <0.001 |
| 2365 | C | T |  | 115/724 (15.9) |  | 24/81 (29.6) |  | 2.230 (1.330-3.739) | 0.002 |  | 2.644 (1.520-4.600) | <0.001 |
| 2389 | G | A |  | 115/725 (15.9) |  | 24/80 (30.0) |  | 2.273 (1.354-3.816) | 0.002 |  | 2.705 (1.553-4.713) | <0.001 |
| 2398 | T | C |  | 114/725 (15.7) |  | 25/80 (31.3) |  | 2.436 (1.458-4.070) | <0.001 |  | 2.607 (1.507-4.513) | <0.001 |
| 2476 | A | G |  | 110/693 (15.9) |  | 29/112 (25.9) |  | 1.852 (1.158-2.961) | 0.010 |  | 2.016 (1.223-3.325) | 0.006 |
| 2499 | A | G |  | 119/744 (16.0) |  | 20/61 (32.8) |  | 2.562 (1.450-4.528) | 0.001 |  | 2.827 (1.542-5.185) | <0.001 |
| 2520 | G | A |  | 115/726 (15.8) |  | 24/79 (30.4) |  | 2.318 (1.379-3.897) | 0.002 |  | 2.731 (1.567-4.760) | <0.001 |
| 2563 | G | A |  | 111/695 (16.0) |  | 28/110 (25.5) |  | 1.797 (1.118-2.888) | 0.016 |  | 2.034 (1.226-3.377) | 0.006 |
| 2587 | C | T |  | 111/698 (15.9) |  | 28/107 (26.2) |  | 1.874 (1.164-3.018) | 0.010 |  | 2.087 (1.255-3.469) | 0.005 |
| 2719 | G | A |  | 83/416 (20.0) |  | 56/389 (14.4) |  | 0.675 (0.465-0.978) | 0.038 |  | 0.674 (0.456-0.997) | 0.048 |
| 2785 | C | T |  | 128/777 (16.5) |  | 11/28 (39.3) |  | 3.281 (1.501-7.169) | 0.003 |  | 2.726 (1.167-6.368) | 0.020 |
| 2797 | G | T |  | 111/693 (16.0) |  | 28/110 (25.5) |  | 1.790 (1.114-2.878) | 0.016 |  | 2.054 (1.237-3.412) | 0.005 |
| 2824 | T | C |  | 115/729 (15.8) |  | 24/75 (32.0) |  | 2.513 (1.487-4.245) | <0.001 |  | 2.896 (1.654-5.073) | <0.001 |
| 2836 | T | C |  | 115/730 (15.8) |  | 24/75 (32.0) |  | 2.517 (1.490-4.252) | <0.001 |  | 2.922 (1.666-5.122) | <0.001 |
| 2854 | G | A |  | 108/691 (15.6) |  | 31/114 (27.2) |  | 2.016 (1.272-3.196) | 0.003 |  | 2.264 (1.381-3.713) | 0.001 |
| 2896 | C | T |  | 110/693 (15.9) |  | 25/76 (32.9) |  | 2.598 (1.544-4.370) | <0.001 |  | 2.955 (1.696-5.147) | <0.001 |
| 2929 | T | C |  | 115/724 (15.9) |  | 24/81 (29.6) |  | 2.230 (1.330-3.739) | 0.002 |  | 2.586 (1.487-4.497) | <0.001 |
| 2950 | A | T |  | 111/696 (15.9) |  | 28/108 (25.9) |  | 1.845 (1.146-2.969) | 0.012 |  | 2.076 (1.249-3.449) | 0.005 |
| 3115 | A | G |  | 116/732 (15.8) |  | 23/73 (31.5) |  | 2.443 (1.435-4.159) | 0.001 |  | 2.962 (1.673-5.245) | <0.001 |
| 3128 | T | A |  | 116/731 (15.9) |  | 23/74 (31.1) |  | 2.391 (1.406-4.065) | 0.001 |  | 2.722 (1.545-4.795) | <0.001 |
| 3160 | T | C |  | 112/698 (16.0) |  | 27/107 (25.2) |  | 1.766 (1.092-2.856) | 0.020 |  | 2.040 (1.220-3.412) | 0.007 |
| 3271 | C | T |  | 110/695 (15.8) |  | 29/110 (26.4) |  | 1.904 (1.189-3.048) | 0.007 |  | 2.059 (1.246-3.404) | 0.005 |
| 3277 | C | T |  | 110/696 (15.8) |  | 29/109 (26.6) |  | 1.931 (1.206-3.093) | 0.006 |  | 2.128 (1.287-3.520) | 0.003 |
| 3316 | T | C |  | 116/730 (15.9) |  | 23/75 (30.7) |  | 2.341 (1.379-3.975) | 0.002 |  | 2.690 (1.528-4.735) | <0.001 |
| 3424 | T | C |  | 111/723 (15.4) |  | 27/78 (34.6) |  | 2.919 (1.756-4.853) | <0.001 |  | 3.265 (1.893-5.630) | <0.001 |
| 3517 | A | T |  | 115/732 (15.7) |  | 24/73 (32.9) |  | 2.628 (1.551-4.453) | <0.001 |  | 3.005 (1.709-5.283) | <0.001 |
| 3640 | T | A |  | 114/727 (15.7) |  | 25/78 (32.1) |  | 2.536 (1.514-4.249) | <0.001 |  | 2.955 (1.700-5.140) | <0.001 |
| 3664 | G | A |  | 111/697 (15.9) |  | 28/108 (25.9) |  | 1.848 (1.148-2.974) | 0.011 |  | 2.109 (1.268-3.508) | 0.004 |
| 3685 | C | G |  | 111/697 (15.9) |  | 28/104 (26.9) |  | 1.945 (1.205-3.138) | 0.006 |  | 2.174 (1.304-3.624) | 0.003 |
| 3787 | G | A |  | 111/687 (16.2) |  | 28/118 (23.7) |  | 1.614 (1.009-2.584) | 0.046 |  | 1.824 (1.106-3.008) | 0.019 |
| 3809 | C | T |  | 112/699 (16.0) |  | 27/106 (25.5) |  | 1.791 (1.107-2.899) | 0.018 |  | 2.024 (1.210-3.386) | 0.007 |
| 3874 | A | T |  | 115/726 (15.8) |  | 24/74 (32.4) |  | 2.550 (1.507-4.315) | <0.001 |  | 2.916 (1.661-5.117) | <0.001 |
| 3925 | G | A |  | 113/723 (15.6) |  | 24/78 (30.8) |  | 2.399 (1.425-4.040) | <0.001 |  | 2.762 (1.584-4.815) | <0.001 |
| 3937 | A | T |  | 111/695 (16.0) |  | 28/109 (25.7) |  | 1.819 (1.131-2.925) | 0.014 |  | 2.074 (1.248-3.446) | 0.005 |
| 3997 | C | T |  | 114/728 (15.7) |  | 25/77 (32.5) |  | 2.589 (1.544-4.343) | <0.001 |  | 2.850 (1.638-4.957) | <0.001 |
| 4069 | A | G |  | 112/703 (15.9) |  | 27/102 (26.5) |  | 1.900 (1.171-3.082) | 0.009 |  | 2.197 (1.308-3.690) | 0.003 |
| 4093 | A | G |  | 119/743 (16.0) |  | 19/61 (31.1) |  | 2.372 (1.333-4.221) | 0.003 |  | 2.763 (1.487-5.136) | 0.001 |
| 4162 | G | A |  | 110/690 (15.9) |  | 29/115 (25.2) |  | 1.778 (1.114-2.838) | 0.016 |  | 1.994 (1.210-3.286) | 0.007 |
| 4171 | G | A |  | 110/692 (15.9) |  | 29/113 (25.7) |  | 1.827 (1.143-2.919) | 0.012 |  | 2.043 (1.238-3.371) | 0.005 |
| 4192 | A | T |  | 111/693 (16.0) |  | 28/112 (25.0) |  | 1.748 (1.089-2.806) | 0.021 |  | 2.022 (1.219-3.354) | 0.006 |
| 4201 | G | A |  | 107/675 (15.9) |  | 32/130 (24.6) |  | 1.733 (1.106-2.717) | 0.016 |  | 1.776 (1.102-2.860) | 0.018 |
| 4261 | C | T |  | 110/691 (15.9) |  | 29/114 (25.4) |  | 1.802 (1.128-2.878) | 0.014 |  | 2.025 (1.228-3.340) | 0.006 |
| 4288 | T | C |  | 111/691 (16.1) |  | 28/114 (24.6) |  | 1.701 (1.061-2.728) | 0.027 |  | 1.942 (1.174-3.215) | 0.010 |
| 4297 | C | T |  | 111/694 (16.0) |  | 28/111 (25.2) |  | 1.772 (1.103-2.846) | 0.018 |  | 2.029 (1.223-3.367) | 0.006 |
| 4313 | A | G |  | 115/728 (15.8) |  | 24/77 (31.2) |  | 2.414 (1.433-4.067) | <0.001 |  | 2.783 (1.594-4.862) | <0.001 |
| 4342 | G | A |  | 115/729 (15.8) |  | 24/75 (32.0) |  | 2.513 (1.487-4.245) | <0.001 |  | 2.914 (1.662-5.107) | <0.001 |
| 4355 | A | G |  | 111/691 (16.1) |  | 28/114 (24.6) |  | 1.701 (1.061-2.728) | 0.027 |  | 1.902 (1.149-3.147) | 0.012 |
| 4531 | G | A |  | 115/729 (15.8) |  | 24/76 (31.6) |  | 2.464 (1.461-4.158) | <0.001 |  | 2.860 (1.635-5.002) | <0.001 |
| 4558 | A | G |  | 111/697 (15.9) |  | 28/108 (25.9) |  | 1.848 (1.148-2.974) | 0.011 |  | 2.064 (1.243-3.428) | 0.005 |
| 4645 | C | T |  | 111/693 (16.0) |  | 28/112 (25.0) |  | 1.748 (1.089-2.806) | 0.021 |  | 2.024 (1.220-3.358) | 0.006 |
| 4682 | T | C |  | 116/730 (15.9) |  | 23/75 (30.7) |  | 2.341 (1.379-3.975) | 0.002 |  | 2.733 (1.551-4.817) | <0.001 |
| 4768 | A | G |  | 111/692 (16.0) |  | 28/113 (24.8) |  | 1.724 (1.075-2.767) | 0.024 |  | 1.999 (1.206-3.314) | 0.007 |
| 4819 | C | T |  | 115/728 (15.8) |  | 24/75 (32.0) |  | 2.508 (1.485-4.238) | <0.001 |  | 2.908 (1.659-5.096) | <0.001 |
| 4870 | C | T |  | 113/698 (16.2) |  | 26/107 (24.3) |  | 1.662 (1.023-2.700) | 0.040 |  | 1.891 (1.127-3.174) | 0.016 |
| 5023 | A | T |  | 111/695 (16.0) |  | 28/110 (25.5) |  | 1.797 (1.118-2.888) | 0.016 |  | 2.049 (1.234-3.402) | 0.006 |
| 5035 | T | C |  | 111/694 (16.0) |  | 28/111 (25.2) |  | 1.772 (1.103-2.846) | 0.018 |  | 2.012 (1.213-3.337) | 0.007 |
| 5067 | G | A |  | 115/729 (15.8) |  | 24/76 (31.6) |  | 2.464 (1.461-4.158) | <0.001 |  | 2.908 (1.659-5.096) | <0.001 |
| 5110 | C | A |  | 111/691 (16.1) |  | 28/111 (25.2) |  | 1.763 (1.097-2.832) | 0.019 |  | 2.001 (1.207-3.318) | 0.007 |
| 5125 | C | T |  | 111/695 (16.0) |  | 28/110 (25.5) |  | 1.797 (1.118-2.888) | 0.016 |  | 2.049 (1.234-3.402) | 0.006 |
| 5173 | C | T |  | 111/695 (16.0) |  | 28/110 (25.5) |  | 1.797 (1.118-2.888) | 0.016 |  | 2.049 (1.234-3.402) | 0.006 |
| 5197 | A | G |  | 110/689 (16.0) |  | 28/114 (24.6) |  | 1.714 (1.068-2.749) | 0.026 |  | 1.894 (1.145-3.131) | 0.013 |
| 5425 | C | G |  | 108/689 (15.7) |  | 28/110 (25.5) |  | 1.837 (1.142-2.956) | 0.012 |  | 2.086 (1.256-3.466) | 0.005 |
| 5440 | T | C |  | 109/679 (16.1) |  | 30/123 (24.4) |  | 1.687 (1.065-2.671) | 0.026 |  | 1.891 (1.158-3.086) | 0.011 |
| 5476 | C | T |  | 115/725 (15.9) |  | 24/80 (30.0) |  | 2.273 (1.354-3.816) | 0.002 |  | 2.576 (1.483-4.473) | <0.001 |
| 5479 | A | G |  | 111/695 (16.0) |  | 28/110 (25.5) |  | 1.797 (1.118-2.888) | 0.016 |  | 2.049 (1.234-3.402) | 0.006 |
| 5530 | C | A |  | 86/551 (15.6) |  | 28/111 (25.2) |  | 1.824 (1.122-2.966) | 0.015 |  | 2.048 (1.220-3.438) | 0.007 |
| 5560 | A | G |  | 106/675 (15.7) |  | 33/130 (25.4) |  | 1.826 (1.169-2.853) | 0.008 |  | 2.128 (1.319-3.436) | 0.002 |
| 5572 | G | A |  | 111/691 (16.1) |  | 28/114 (24.6) |  | 1.701 (1.061-2.728) | 0.027 |  | 1.944 (1.175-3.216) | 0.010 |
| 5599 | C | T |  | 112/711 (15.8) |  | 26/91 (28.6) |  | 2.139 (1.301-3.518) | 0.003 |  | 2.415 (1.421-4.107) | 0.001 |
| 5617 | C | T |  | 115/732 (15.7) |  | 24/73 (32.9) |  | 2.628 (1.551-4.453) | <0.001 |  | 3.076 (1.747-5.414) | <0.001 |
| 5650 | G | A |  | 111/695 (16.0) |  | 28/110 (25.5) |  | 1.797 (1.118-2.888) | 0.016 |  | 2.049 (1.234-3.402) | 0.006 |
| 5674 | G | A |  | 111/695 (16.0) |  | 28/110 (25.5) |  | 1.797 (1.118-2.888) | 0.016 |  | 2.102 (1.265-3.493) | 0.004 |
| 5785 | C | T |  | 109/690 (15.8) |  | 30/115 (26.1) |  | 1.881 (1.183-2.991) | 0.008 |  | 2.139 (1.303-3.510) | 0.003 |
| 5788 | G | A |  | 111/696 (15.9) |  | 28/109 (25.7) |  | 1.822 (1.133-2.930) | 0.013 |  | 2.059 (1.240-3.419) | 0.005 |
| 5875 | T | C |  | 110/693 (15.9) |  | 29/110 (26.4) |  | 1.898 (1.185-3.038) | 0.008 |  | 2.110 (1.275-3.490) | 0.004 |
| 5923 | A | G |  | 109/682 (16.0) |  | 30/123 (24.4) |  | 1.696 (1.071-2.685) | 0.024 |  | 1.858 (1.138-3.033) | 0.013 |
| 5938 | A | G |  | 109/686 (15.9) |  | 30/118 (25.4) |  | 1.805 (1.137-2.865) | 0.012 |  | 1.961 (1.196-3.214) | 0.008 |
| 6118 | C | T |  | 114/729 (15.6) |  | 25/76 (32.9) |  | 2.644 (1.574-4.442) | <0.001 |  | 3.093 (1.774-5.391) | <0.001 |
| 6124 | T | C |  | 111/693 (16.0) |  | 28/109 (25.7) |  | 1.812 (1.127-2.915) | 0.014 |  | 2.052 (1.236-3.408) | 0.005 |
| 6187 | T | A |  | 115/728 (15.8) |  | 24/75 (32.0) |  | 2.508 (1.485-4.238) | <0.001 |  | 2.906 (1.658-5.093) | <0.001 |
| 6238 | T | C |  | 115/730 (15.8) |  | 24/75 (32.0) |  | 2.517 (1.490-4.252) | <0.001 |  | 2.916 (1.664-5.111) | <0.001 |
| 6276 | A | G |  | 113/723 (15.6) |  | 26/82 (31.7) |  | 2.506 (1.510-4.160) | <0.001 |  | 2.526 (1.468-4.347) | <0.001 |
| 6289 | C | A |  | 85/518 (16.4) |  | 27/109 (24.8) |  | 1.677 (1.024-2.747) | 0.040 |  | 1.907 (1.126-3.229) | 0.016 |
| 6314 | A | G |  | 111/699 (15.9) |  | 28/106 (26.4) |  | 1.902 (1.180-3.064) | 0.008 |  | 2.181 (1.308-3.636) | 0.003 |
| 6322 | A | G |  | 117/734 (15.9) |  | 22/69 (31.9) |  | 2.468 (1.433-4.251) | 0.001 |  | 3.004 (1.675-5.386) | <0.001 |
| 6333 | A | G |  | 130/786 (16.5) |  | 9/19 (47.4) |  | 4.542 (1.810-11.396) | 0.001 |  | 5.021 (1.889-13.342) | 0.001 |

OR=odds ratio. CI=confidence interval. The nucleotide mutations with a frequency less than 2% were not included in the association analysis.

*****Adjusting for age, sex, and hospitalization delay.

**Appendix Table 14. Number of co-mutation patterns located in four SFTSV viral clades.**

| Co-mutation pattern | Clade I | Clade II | Clade III | Clade IV | Total |
| --- | --- | --- | --- | --- | --- |
| I |  |  | 34 | 73 | 107 |
| II |  |  |  | 73 | 73 |
| III |  |  | 34 |  | 34 |

**Appendix Table 15. Co-mutation patterns related to fatal outcome of patients with SFTS.**

| **Co-mutation patter** |  | **Fatal/Total (%)** | | |  | **Multivariate regression^*^** | |  | **Multivariate regression^#^** | |
| --- | --- | --- | --- | --- | --- | --- | --- | --- | --- | --- |
|  |  | **Mutation type** | **Wild type** | **All others** |  | **OR (95% CI)** | **p** |  | **OR (95% CI)** | **p** |
| I |  | 28/107 (26.2) | 109/678 (16.1) | 109/681 (16.0) |  | 2.077 (1.250-3.453) | 0.004 |  | 2.089 (1.257-3.472) | 0.005 |
| II |  | 24/73 (32.9) | 89/549 (16.2) | 113/715 (15.8) |  | 2.886 (1.624-5.130) | <0.001 |  | 2.990 (1.702-5.254) | <0.001 |
| III |  | 4/34 (11.8) | 133/754 (17.6) | 133/754 (17.6) |  | 0.631 (0.208-1.916) | 0.417 |  | 0.631 (0.208-1.916) | 0.417 |

OR=odds ratio. CI=confidence interval.

**^*^**Wide type sequences setting as reference and adjusting for age, sex, and hospitalization delay.

**^#^**All other sequences setting as reference and adjusting for age, sex, and hospitalization delay.

**Appendix Table 16. Basic features of patients infected with four SFTSV clades included for analysis of serum cytokine levels.**

|  | Clade I | Clade II | Clade III | Clade IV | p |
| --- | --- | --- | --- | --- | --- |
| Age | 65 (57.5-72) | 63.5 (53-70) | 59 (54-73) | 63 (56.5-70) | 0.796 |
| Female, IQR | 26 (48.1) | 22 (68.8) | 10 (66.7) | 14 (56.0) | 0.249 |
| Delay | 5 (4-7) | 5 (4-6) | 5 (4-5) | 5 (4-6) | 0.706 |
| Viral load, (log_10_ copies/mL) | 6.20±1.00 | 6.19±1.09 | 5.98±1.00 | 6.14±0.92 | 0.906 |

IQR, interquartile-range.

**Appendix Table 17. Basic features of patients infected with wide type or co-mutation pattern II SFTSV included for analysis of serum cytokine levels.**

|  | Wide type | Co-mutation pattern II | p |
| --- | --- | --- | --- |
| Age | 64.5 (56-72) | 62 (55-68) | 0.414 |
| Female, IQR | 60 (55.6) | 12 (66.7) | 0.268 |
| Delay | 5 (4-7) | 5.5 (5-7) | 0.189 |
| Viral load, (log_10_ copies/mL) | 6.16±1.02 | 6.16±0.95 | 0.988 |

IQR, interquartile-range.

**References**

1. Li H, Li X, Lv S, et al. Single-cell landscape of peripheral immune responses to fatal SFTS. Cell Rep. 2021 Nov 23;37(8):110039.
2. Li H, Zhang LK, Li SF, et al. Calcium channel blockers reduce severe fever with thrombocytopenia syndrome virus (SFTSV) related fatality. Cell Res. 2019 Sep;29(9):739-753.
3. Yun SM, Park SJ, Kim YI, et al. Genetic and pathogenic diversity of severe fever with thrombocytopenia syndrome virus (SFTSV) in South Korea. JCI Insight. 2020 Jan 30;5(2).
